# Major Histocompatibility Complex Genomic Regions as Candidates for Reproductive Success in Cattle: From GWAS to Post-GWAS Analysis

**DOI:** 10.3390/genes17080895

**Published:** 2026-07-30

**Authors:** Louíse B. Faverzani, Andreia K. Zwirtes, Dinah P. A. Rodrigues, Thais F. Machado, Daniele A. Oliveira, Luiza C. Silveira, Nathalia P. Seixas, Evandro N. Silva, Danielly B. S. Silva, Ricardo Z. Vaz, Thaise P. Melo

**Affiliations:** 1 Animal Science Department, Federal University of Santa Maria, Santa Maria 97105900, Rio Grande do Sul, Brazil; louise.faverzani@acad.ufsm.br (L.B.F.); andreia.kaspary@acad.ufsm.br (A.K.Z.); rodrigues.dinah@acad.ufsm.br (D.P.A.R.); thais.machado@acad.ufsm.br (T.F.M.); daniele.alves@acad.ufsm.br (D.A.O.); luiza.comin@acad.ufsm.br (L.C.S.); nathaliaaseixasp@gmail.com (N.P.S.); ricardo.vaz@ufsm.br (R.Z.V.); 2 Department of Microbiology and Immunology, Federal University of Alfenas, Alfenas 37130001, Minas Gerais, Brazil; evandro.silva@sou.unifal-mg.edu.br; 3 Universidade José do Rosário Vellano, Câmpus Alfenas, Rodovia MG-179, Km 0, Câmpus Universitário, Loteamento Trevo, Alfenas 37132440, Minas Gerais, Brazil; daniellyberaldo@gmail.com

**Keywords:** bovine leukocyte antigen, gene regulatory networks, genetic selection, transcription factors

## Abstract

Background: The major histocompatibility complex (MHC) is a well-known gene complex that plays a fundamental role in immune recognition; however, its influence on reproductive processes in cattle is not yet fully established, particularly regarding how it modulates the female response to the embryo. Therefore, this study aimed to investigate the relationship between MHC genes and reproductive processes in cattle and to identify potential molecular targets involved in these mechanisms. Methods: Data from four studies identified through a systematic review were integrated. MHC genes associated with reproduction were identified, neighboring genomic regions were explored to detect candidate genes and transcription factors, and biological process networks were constructed using differentially expressed genes associated with cattle. Results: The integrated analysis led to the identification of six MHC genes associated with reproduction, along with 54 candidate genes from genome-wide association studies and 46 from transcriptomic studies located in neighboring regions. Among these, *BoLA-DQB* and *BoLA-DQA5* stood out in all analyses, while the transcription factors *FOXA1*, *FOXO3*, *ELF5*, and *STAT3* appeared across the different datasets. These genes and factors may represent potential targets for future functional validations and could be explored using genome editing technologies to better understand their roles in reproductive processes. The identified biological pathways were related to embryonic development and reproductive organ formation, providing a preliminary foundation for future investigations. Conclusions: This work integrates currently available evidence and highlights potential molecular targets that can be explored in cattle breeding and reproductive biology, associating MHC genes with these roles and advancing innovative perspectives in the field of molecular genetics.

## 1. Introduction

The major histocompatibility complex (MHC) is a group of genes encoding cell surface antigens that play an essential role in the immune response [1]. It is considered one of the most gene-dense regions in the mammalian genome [2]. Bovine MHC genes have been mapped to *Bos taurus* chromosome 23 (BTA23) [3,4], known as bovine leukocyte antigen (*BoLA*) [5], and are associated with both resistance and susceptibility to a variety of diseases. The *BoLA* genes have been linked to variability in resistance to diseases such as mastitis and bovine leukemia virus-induced lymphoma [6].

In addition to their central role in innate and adaptive immune responses, MHC gene products also perform significant functions in behavioral contexts [7] and reproduction [8]. In wild mammal animal populations, females often have the opportunity to select mates before, during, or after copulation, whereas in domesticated livestock, mate choice is largely determined by breeding management [9].

In humans, MHC genes are involved in maternal–fetal immune interactions and have been associated with embryo implantation, pregnancy maintenance, and reproductive success [10]. Furthermore, some genome-wide association (GWAS) or transcriptomic studies have reported genomic regions at BTA23 associated with reproductive traits in cattle [11,12,13,14]. These findings suggest that genomic regions near the MHC may be associated with reproductive roles; however, evidence remains fragmented, particularly in cattle, and the potential functional relevance of candidate genes in this region has yet to be systematically explored.

With recent advances in omics approaches, particularly functional and structural genomics, a deeper understanding of the multiple roles played by MHC genes and adjacent genomic regions in cattle reproductive processes is emerging. In this context, GWAS have been essential for identifying candidate genomic regions associated with economically important traits in various domestic species [15,16,17,18]. Identifying genes associated with reproductive traits contributes to a better understanding of the biological mechanisms underlying fertility and may support the identification of genomic markers for incorporation into cattle breeding programs [19,20,21].

Although MHC genes are traditionally related to immunological processes in various species, some studies have also linked them to reproductive processes, especially in humans. Thus, this study aimed to investigate the association between MHC genes located in BTA23 regions and reproductive functions in cattle and to validate the results in other mammalian species by integrating GWAS, transcriptomic data obtained through a systematic review, and sequencing results.

## 2. Methods

### 2.1. Systematic Review

A systematic review was conducted as per PRISMA guidelines [22] to find articles published in indexed journals that identified genes of the MHC associated with bovine reproduction. The search was performed in the PubMed https://pubmed.ncbi.nlm.nih.gov/ (accessed on 30 May 2024), ScienceDirect https://www.sciencedirect.com/ (accessed on 30 May 2024), Web of Science https://clarivate.com/academia-government/scientific-and-academic-research/research-discovery-and-referencing/web-of-science/ (accessed on 30 May 2024) and SciELO https://www.scielo.br/ (accessed on 30 may 2024) databases between April and May 2024 using four keyword combinations: ((bovine) AND (MHC)) AND ((reproductive) OR (reproduction)). Two authors (A.K.Z. and D.P.A.R.) independently performed the search in the databases, and inconsistencies were resolved by a third author (T.P.M.).

The inclusion criteria comprised (i) articles published in English, (ii) from 2014 to 2024, (iii) conducted with cattle, (iv) using transcriptomic analysis or GWAS, (v) focused on reproductive traits, and (vi) that explicitly reported the names of MHC genes or at least their respective genomic regions. The information extracted from each article was organized in a Microsoft Excel spreadsheet and included the author, year of publication, journal, article title, cattle breed, country where the study was conducted, investigated phenotypic trait, sample population size, and MHC genes related to reproductive traits. All studies were independently analyzed and any disagreements were resolved by consensus among the authors. Systematic reviews and duplicates were excluded.

### 2.2. Network of Biological Processes

After identifying the MHC genes found in the systematic review, we determined the genomic coordinates of these MHC genes using the BioMart platform (https://www.ensembl.org/biomart/martview/a90116daed5edbc7b40d7607471aca83, accessed on 30 May 2024) [23], based on version ARS-UCD1.3 of the Ensembl database (https://oct2024.archive.ensembl.org/Bos_taurus/Info/Index, accessed on 30 May 2024). The chromosomal positions of all MHC genes identified in this study are provided in Supplementary Table S1. A genomic window of ±0.25 Mb surrounding each MHC gene was then constructed. This window was defined to account for linkage disequilibrium between MHC genes and neighboring genes, allowing the inclusion of genes that may be functionally associated with MHC genes through genomic co-inheritance. A recent study on linkage disequilibrium decay in cattle indicated that linkage disequilibrium may extend up to approximately 500 kb in several breeds, including Nelore and Holstein, with average values around r^2^ ≈ 0.20, which are informative for genomic association studies [24]. Therefore, a ±0.25 Mb window was adopted as a conservative interval to capture genes potentially in linkage disequilibrium with the MHC region. This step produced two candidate gene lists: one from the GWAS and the other from the transcriptomic studies (Supplementary Table S2). These gene lists served as input for the DAVID software (https://davidbioinformatics.nih.gov/, accessed on 30 May 2024) [25], which provided a separate functional analysis and visualization of the genomic data for each set of genes (Supplementary Table S3). From these results, the most significant cluster with the highest enrichment score was extracted, and the five most relevant terms from this cluster were considered for discussion.

The next step involved a search in the National Center for Biotechnology Information database (https://www.ncbi.nlm.nih.gov/gene/, accessed on 30 May 2024) to identify protein-coding genes on chromosome 23 of the bovine genome. Each gene was identified and located, and the corresponding nucleotide sequences, consisting of 3300 base pairs around the genes (including 3000 base pairs upstream and 300 base pairs downstream), were extracted from the ARS-UCD1.3 assembly on the National Center for Biotechnology Information website. This was done to include promoter and regulatory regions that may influence gene expression [15]. A FASTA file was generated containing promoter sequences of candidate genes identified in the GWAS and transcriptomic studies (Supplementary File S1). Each file was used as input in the TFM-Explorer software v.2.0 (https://bioinfo.cristal.univ-lille.fr/tfm-explorer/tfm-explorer.php, accessed on 30 May 2024) [17,26], which analyzes the regulatory regions of co-regulated gene sequences. The sequences from the generated files were scanned to identify potential transcription factor binding sites using weight matrices from the JASPAR database [27,28]. Significant clusters corresponding to transcription factor binding sites within the selected gene sequences were identified using the TFM-Explorer program. The score threshold was established to retain only significant enrichment signals with *p* ≤ 10^−4^, representing a more stringent criterion for identifying potential regulatory relationships. Based on these results, gene–transcription factor regulatory networks were constructed using Cytoscape software, including only interactions meeting this significance threshold. Functional enrichment analyses were subsequently performed using the BiNGO plugin in Cytoscape to identify significantly enriched Gene Ontology biological processes associated with the candidate genes.

The list of transcription factors (Supplementary File S2) identified was imported into Cytoscape v. 3.10.2 [29]. Gene Ontology enrichment analysis was performed using the Biological Networks Gene Ontology (v. 3.0.5) plug-in to determine significantly overrepresented functional Gene Ontology terms based on hypergeometric tests and multiple test corrections (*p* ≤ 0.05) [30,31]. In this step, the significance threshold was only used to filter and visualize the most relevant biological networks associated with reproduction. Afterward, the transcription factors and identified genes were re-entered into Cytoscape to create a functional network, highlighting the transcription factors and their respective genetic groupings [32]. Additionally, comparisons were made between genes, transcription factors, and biological networks identified in the GWAS and transcriptome studies to assess possible similarities and differences between both omics.

## 3. Results

### 3.1. MHC Genes from the Systematic Review

**Figure 1:**
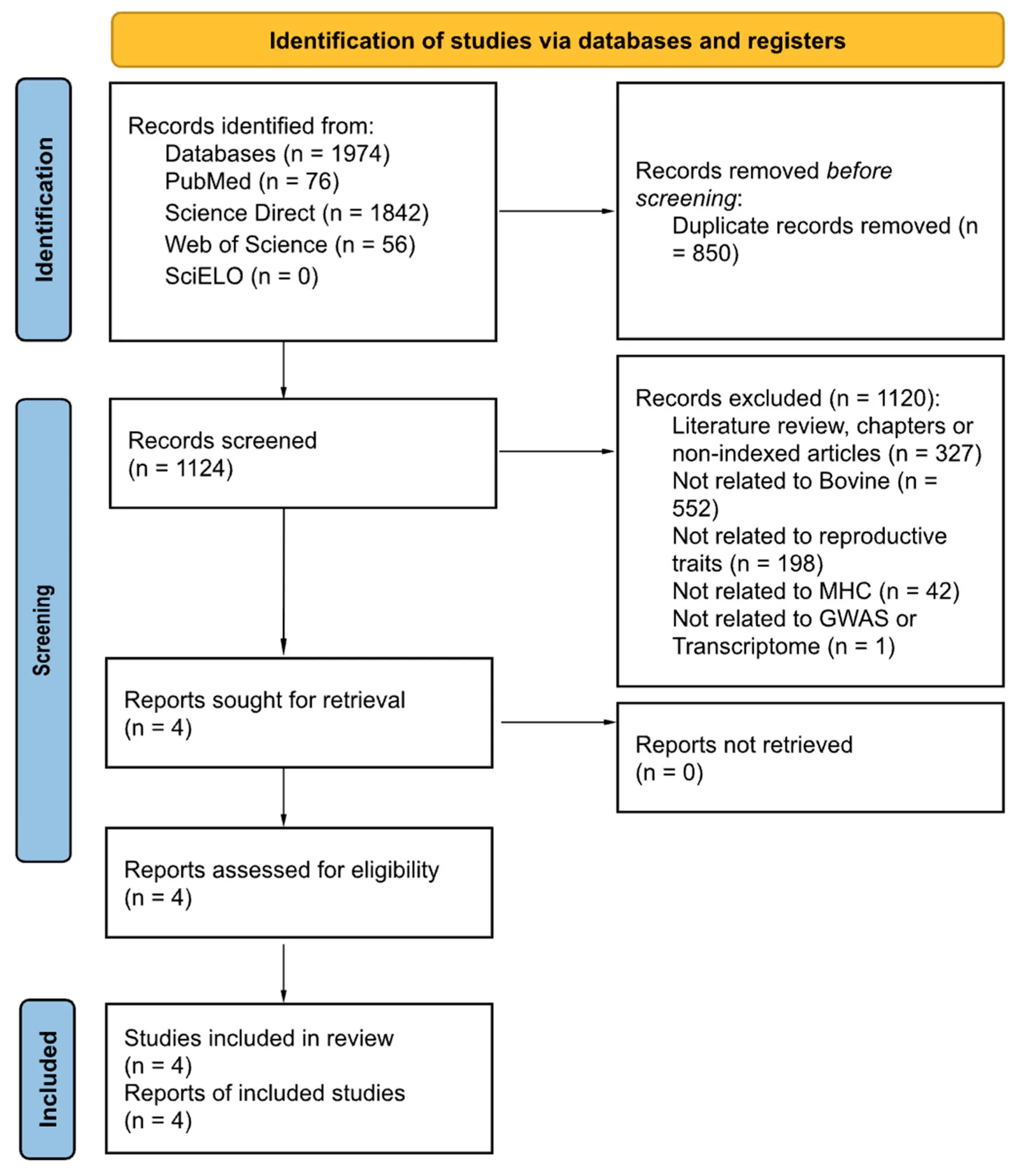
PRISMA flow diagram of the search, inclusion, and exclusion process for identifying review articles.

A systematic review was conducted through a rigorous process of article search, selection, and exclusion (Figure 1). Consequently, four studies were selected for analysis.

### 3.2. Review and Functional Analysis of Candidate Genes and Metabolic Pathways

After quality control, four articles [11,12,13,14] were retained for the systematic review. These studies investigated reproductive phenotypes related to female fertility, including early first calving, heifer rebreeding, and the number of calvings by 53 months, as well as developmental processes associated with successful pregnancy, such as conceptus elongation and fetal development. The studies included Holstein, Nelore, and crossbred cattle (Supplementary Table S1).

A total of five MHC genes were extracted from the four selected articles, following the inclusion criteria previously described: *BOLA-NC1*, *BOLA-DQB*, *BOLA-DQA1*, *BOLA-DQA2*, *BOLA-DRA*, and *HLA-DQA1*. All five genes were identified in GWAS studies, while two (*BoLA-NC1* and *BoLA-DQA1*) appeared in transcriptome studies. The complete list of candidate genes identified in the literature, organized by chromosome and position, is provided in Supplementary Table S1. A genomic window with a total size of 0.5 Mb was constructed using the BioMart platform (one based on GWAS studies and the other based on transcriptome studies) resulting in the identification of 54 and 46 candidate genes, respectively, surrounding the MHC genes, all located on chromosome BTA23 (Supplementary Table S2). In subsequent analyses, only protein-coding genes were considered.

### 3.3. Functional Analysis and Candidate Genes

Of the 54 and 46 candidate genes identified by the GWAS and transcriptome studies, respectively, the Database for Annotation, Visualization, and Integrated Discovery (DAVID) software grouped these genes into three distinct clusters. For discussion, we present only the cluster with the highest enrichment score (8.42), which was the top 1 cluster (Table 1). This cluster contained nine genes organized into five main terms, based on the most significant *p*-value. No differences were observed in the main terms or grouped genes when comparing the GWAS and transcriptome data (Supplementary Table S3).

In the Cytoscape software, after biological interpretation and manual curation of enriched terms, biological processes directly related to reproductive tissues and embryonic/fetal development were retained for further discussion. Twenty processes were identified as related to the candidate genes from GWAS studies, and fifteen processes were linked with candidate genes obtained through transcriptome studies (Supplementary Table S4). Among these, seven biological processes were common to both datasets; these processes encompass key aspects of reproduction such as embryonic development, oogenesis, ovulation, and the formation of reproductive organs and tissues (e.g., mammary gland and prostate).

**Figure 2:**
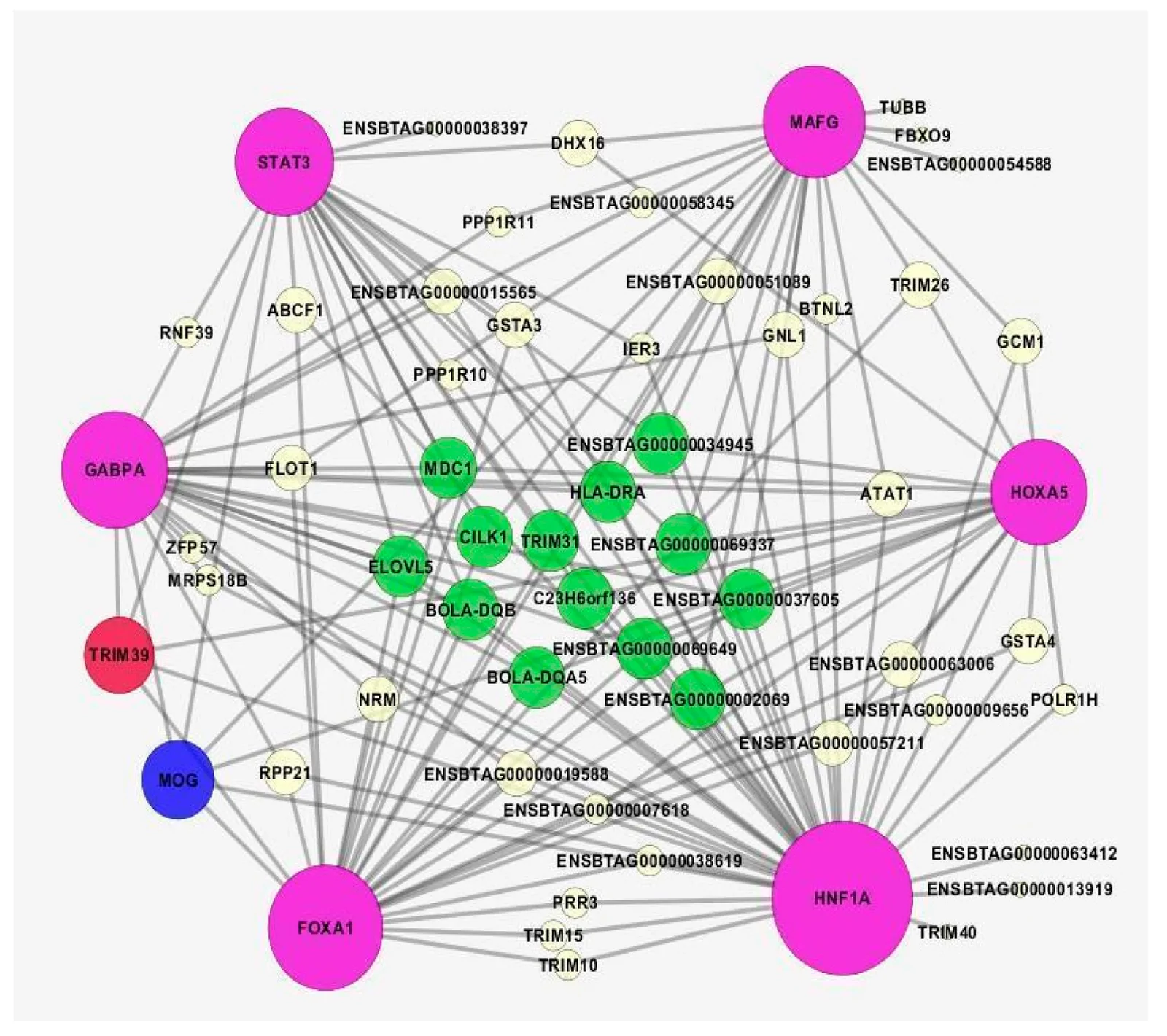
Gene–transcription factor regulatory network based on candidate genes identified in GWAS. Rounded bigger nodes (pink) represent transcription factors (FOXA1, STAT3, HNF1A, MAFG, GABPA, and HOXA5). circular nodes represent candidate genes. Red (TRIM39) and blue (MOG) nodes highlight the candidate genes with the highest number of predicted regulatory interactions with transcription factors in the network. Edges represent predicted regulatory relationships between transcription factors and candidate genes identified using the TFM-Explorer tool. All interactions were selected based on significance values of *p* ≤ 10^−4^. Candidate genes are color-coded according to their significance values obtained from the TFM-Explorer analysis, with dark pink representing the highest significance values, followed by green and light-yellow representing lower significance values. No predefined thresholds were established for the color categories.

In total, eight transcription factors associated with these processes were identified: *FOXA1*, *STAT3*, *HNF1A*, *MAFG*, *GABPA*, *HOXA5*, *FOXO3*, and *ELF5*. Of these, two were from GWAS studies (*FOXA1* and *HOXA5*), while two were identified exclusively in the transcriptome studies (*FOXO3* and *ELF5*). Analyses of 54 GWAS candidate genes identified 15 genes most enriched for the studied pathway (Figure 2). The *MOG* gene was associated with all six transcription factors, while *TRIM39* was associated with five of them, suggesting a possible central role in the regulation of reproductive processes.

**Figure 3:**
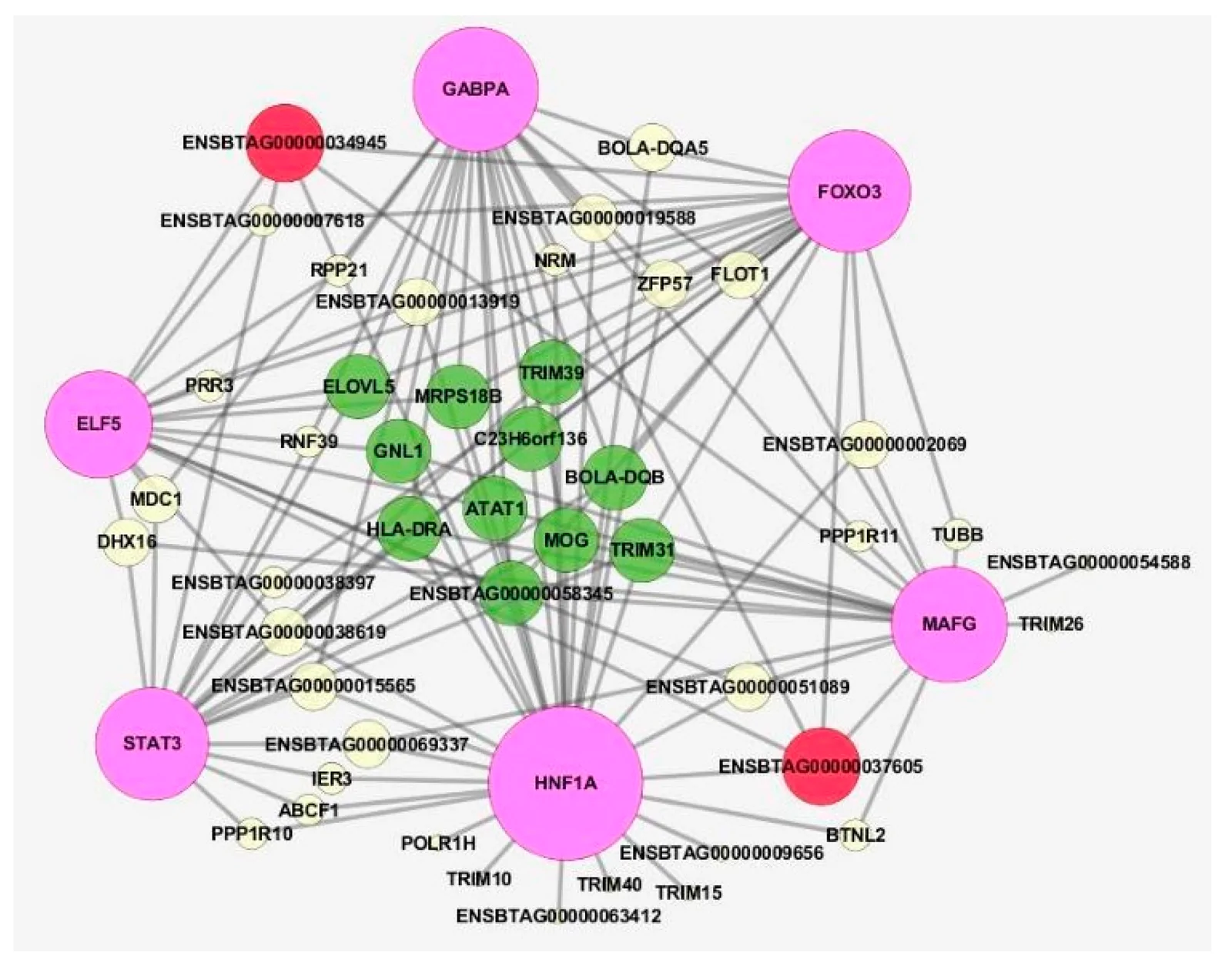
Gene–transcription factor regulatory network based on candidate genes identified in transcriptomic studies. Rounded nodes represent transcription factors (STAT3, HNF1A, MAFG, GABPA, FOXO3, and ELF5), circular nodes represent candidate genes. Red nodes (ENSBTAG0000034945 and ENSBTAG0000037605) highlight the candidate genes with the highest number of predicted regulatory interactions with transcription factors in the network. Edges represent predicted regulatory relationships between transcription factors and candidate genes identified using the TFM-Explorer tool. All interactions were selected based on significance values of *p* ≤ 10^−4^. Candidate genes are color-coded according to their significance values obtained from the TFM-Explorer analysis, with dark pink representing the highest significance values, followed by green and light-yellow representing lower significance values. No predefined thresholds were established for the color categories.

Of the 46 candidate genes identified in the transcriptome studies, 13 were highlighted in the gene–transcription factor network (Figure 3). Among these, *ENSBTAG00000034945* and *ENSBTAG00000037605* stood out due to their association with five transcription factors each, underscoring their potential importance in the analyzed pathways, as these factors are linked to reproductive processes.

Some candidate genes identified by transcriptomic approaches overlapped with those identified in GWAS studies included in this systematic review, indicating consistency between these complementary methodologies, as *BoLA-NC1* and *BoLA-DQA1* were identified in the systematic review using both GWAS and transcriptome methods. The analyses using both DAVID and TFM-Explorer tools were conducted separately for the gene sets derived from GWAS and transcriptome studies. Notably, two genes (*BoLA-DQB* and *BoLA-DQA5*) were identified by both tools, regardless of the dataset. When comparing the results obtained from GWAS and transcriptomic analyses, differences were observed between the gene sets. Specifically, the genes *GSTA3*, *GSTA4*, and *CILK1* were identified solely in the GWAS dataset, whereas *HLA-DRA*, *ZFP57*, and *C23H6orf136* were unique to the transcriptomic analysis.

## 4. Discussion

Understanding the biological processes that regulate the bovine reproductive system is essential for accurately assessing the factors that influence or impair pregnancy [33]. Bovine reproduction is a complex process regulated by a series of hormonal, physiological, and environmental interactions [34]. Genetic factors also contribute to this process, as certain genes are responsible for coordinating the regulatory mechanisms that govern these complex interactions, whether hormonal or physiological. Hence, we identified five MHC genes related to reproductive traits of economic interest in four studies, which may suggest a possible association between MHC genomic regions and processes related to reproduction and embryonic development and, consequently, contribute to providing a better understanding of the underlying biological mechanisms of reproductive success.

### 4.1. Gene Ontology Analysis

The identified functional categories were mainly related to immune processes, such as “Peptide antigen assembly with MHC class II protein complex,” “Antigen processing and presentation of exogenous peptide antigen via MHC class II,” “MHC class II protein complex binding,” “MHC class II protein complex,” and “MHC class II,” were the candidate genes primarily associated with MHC-related and immune system pathways. This result is expected given that the candidate genes analyzed belong to the bovine major histocompatibility complex (BoLA) and are strongly related to these pathways. Because these genomic regions were linked to reproductive traits in the original studies, these findings suggest that genes involved in adaptive immunity and antigen presentation may also influence bovine reproduction.

The pathway “Antigen processing and presentation of exogenous peptide antigen via MHC class II” plays a crucial role in fetal antigens, enabling the activation of maternal CD4^+^ T cells [35]. This process facilitates immune response modulation during pregnancy, promoting the immune tolerance necessary for the maintenance of gestation. As for the “MHC class II protein complex,” antigen-presenting cells (APCs) expressing MHC class II have been described in the bovine reproductive tract, including the uterus, indicating local immune activity [36]. These APCs exhibited morphology consistent with macrophages, dendritic cells, and fibroblasts, which are integral to immune processes. The presence of these cells in different regions of the reproductive tract suggests a complex immunological organization involving multiple cell types in uterine immune control and regulation.

The expression of “MHC class II” in female reproductive tissues is induced by exposure to semen, which locally activates the adaptive immune response [37]. This activation facilitates immune tolerance to paternal antigens, crucial for preventing fetal rejection during pregnancy. As for pathways “Peptide antigen assembly with MHC class II protein complex” and “MHC class II protein complex binding,” they were associated with immunological processes [38,39], albeit researchers have yet to directly link them to reproduction in cattle, thus indicating the importance of conducting further studies.

The genes *RPL37A* and *RPL35A*, which encode ribosomal proteins essential for protein synthesis, play a fundamental role in bull sperm mutation and embryonic development [40,41,42,43]. These genes are potentially related to reproduction in cattle as they support the high demand for protein translation during key processes such as folliculogenesis, luteinisation, and embryonic development.

*BoLA-DQB1* and *BoLA-DQB* also play critical roles in modulating the immune response, contributing to maintaining a uterine environment favorable for pregnancy. *BoLA-DQB1* regulates the maternal immune response, which may contribute to the creation of a uterine environment conducive to fetal development [44]. Similarly, *BoLA-DQB* promotes maternal immune tolerance and facilitates embryo implantation by reducing the risk of immune rejection of the embryo [45].

MHC genes such as *BoLA-DRA* also play an important role in reproduction, with associations between *BoLA-DRA* and genetic variations linked to higher fertility in bulls [42]. Conversely, *BoLA-DQA5* shows immunological variability in cattle, potentially influencing how the maternal immune system recognizes the fetus during implantation, directly impacting pregnancy maintenance [46]. Additionally, *LOC100848815*, which is expressed in developing embryo cells, has been suggested to play a key role in the onset of cell differentiation, indicating its possible contribution to the early stages of embryonic development [47].

### 4.2. Transcription Factors and Associated Biological Networks

In addition to the identified genes, transcription factors add another layer of regulation that can directly influence reproductive and immune processes in cattle. Candidate genes identified through GWAS and transcriptomic studies were analyzed separately because these approaches represent distinct sources of biological evidence. GWAS identifies genes located within or near genomic regions associated with reproductive traits, whereas transcriptomic studies identify genes differentially expressed under specific reproductive conditions. Furthermore, we would like to confirm if both approaches were in agreement regarding the identification of potential MHC genes related to bovine reproduction.

In the GWAS and transcriptomic studies, seven similar pathways were identified (“reproduction”, “in utero embryonic development”, “embryonic development”, “embryonic development ending in birth or egg hatching”, “chordate embryonic development”, “reproductive structure development” and “mammary gland epithelial cell differentiation”). These pathways are related to reproduction, embryonic development and mammary gland development during pregnancy in cattle.

Although MHC class II genes are not directly involved in embryonic developmental programs, they may indirectly influence embryo growth through regulation of the maternal immune environment [48]. Antigen presentation by professional antigen-presenting cells contributes to the establishment of maternal–fetal immune tolerance, balancing immune activation and tolerance toward paternal antigens. This immunological equilibrium is essential for successful implantation, placental development, and maintenance of pregnancy, thereby providing a favorable environment for normal embryonic development. Consequently, genetic variation within BoLA class II genes may affect reproductive success by modulating immune processes that support embryo development rather than by directly controlling developmental pathways.

The transcription factors identified (e.g., *HOXA5* and *FOXA1*) were detected exclusively in the GWA-based analyses and are closely associated with the development of reproductive organs. *HOXA5* has been frequently associated with the mammary gland and, although specific studies in cattle are lacking, it has been associated with ovarian function and sexual differentiation during embryonic development [49]. *FOXA1* was related to prostate regulation, with studies underscoring its role in maintaining normal epithelial differentiation in this organ [50], albeit direct evidence in cattle is also lacking.

The transcription factors *FOXO3* and *ELF5*, identified exclusively in transcriptome-based studies, were associated with processes related to oocyte development, maturation, and epithelial differentiation of the mammary gland. Notably, biological processes involving oocyte-related events were only observed in transcriptomic analyses. This difference may be associated with the higher ability of transcriptomic approaches to capture gene expression patterns related to specific biological processes, but may also reflect differences among studies regarding the reproductive traits evaluated, cattle breeds, populations, and biological samples analyzed. *FOXO3*, in particular, has been described as a negative regulator of follicular activation, as its silencing accelerates primordial follicle development in cattle [51]. In contrast, *ELF5* has been directly implicated in epithelial differentiation of the mammary gland, with evidence pointing to its role in the functional preparation of the gland during pregnancy in cattle [52].

The transcription factors identified in both GWAS and transcriptomic studies (e.g., *MAFG* and *HNF1A*) also showed associations with embryonic and reproductive processes [53,54]; nevertheless, to date, specific evidence in cattle has not been found. Additionally, *GABPA* and *STAT3* were related to the regulation of reproductive processes, playing essential roles in maintaining an environment suitable for embryonic development in cattle and contributing to semen quality [54,55,56].

*STAT3* was enriched among the transcription factors identified from both the GWAS- and transcriptome-derived datasets, suggesting a potential role for this pathway in early pregnancy. *STAT3* is a key downstream effector of JAK–STAT signaling and regulates processes that are essential for successful pregnancy, including immune modulation, cell proliferation, differentiation, embryo implantation, and placental development. In cattle, recent evidence has identified the JAK2–STAT3 axis as the principal signaling pathway mediating IFN-τ-induced *IFI6* expression, linking its activation to embryo implantation and reduced early pregnancy loss [57]. In addition, Zhu et al. [58] demonstrated that IFN-τ enhances *STAT3* expression, promoting a uterine environment favorable for conceptus implantation through increased *BoLA-I* expression and immunomodulation characterized by increased *IL-10* and reduced *IL-6* expression. Together, these findings support the hypothesis that JAK–STAT signaling, particularly through *STAT3* activation, may represent an important regulatory mechanism underlying several of the candidate genes and biological processes identified in the present review. However, because our conclusions are based on enrichment analyses rather than functional validation, the involvement of this pathway should be interpreted as a biologically plausible association that warrants further experimental investigation.

### 4.3. Transcription Factors and Candidate Genes

Among the 15 candidate genes identified in the GWAS, *MOG* and *TRIM39* are notable for their significant enrichment (*p* ≤ 10^−4^) in biological networks associated with reproductive processes in cattle. *MOG*, which was associated with all the transcription factors analyzed, is primarily associated with the stability and maintenance of myelin, essential for efficient nerve impulses [59]. Therefore, alterations in MOG expression may affect neural functions, although the potential implications for reproductive regulation require further investigation. *TRIM39*, associated with the activity of five distinct transcription factors, is located near the *MHC-Ia* gene and involved in apoptosis regulation and the cell cycle, pivotal processes for tissue homeostasis and immune response [60,61]. Nevertheless, researchers have yet to establish a direct link between these genes and reproductive functions in cattle.

The MHC genes, including *BoLA-DQB*, *BoLA-DRA*, and *BoLA-DQA5*, are prominent for their association with immunological processes. Studies have suggested that the low expression of *BoLA-DQB* may act as a natural mechanism to modulate the local immune response, promoting maternal tolerance and supporting implantation by reducing the risk of immune-mediated embryo rejection [45]. Furthermore, the high prevalence of *BoLA-DQA5* deletion in cattle indicates significant immunological variability, potentially influencing how the maternal immune system recognizes the fetus during gestation [46]. In cattle, *BoLA-DRA* is involved in antigen processing and presentation pathways, mediating the presentation of peptides to CD4^+^ T lymphocytes and contributing to the regulation of adaptive immune responses [42]. Additionally, this gene has been reported to play a role in sperm membrane integrity and morphology [62]. In the bovine corpus luteum, *BoLA-DRA* expression has been detected; however, its precise function remains unclear, with evidence suggesting that it may modulate T lymphocyte responses in a way that supports normal luteal function [63]. Although the direct link between these genes and reproduction is not yet fully understood, their immunomodulatory functions suggest a possible role in maintaining maternal–fetal tolerance and reproductive success. Among the 15 most significantly enriched genes from transcriptome studies, *ENSBTAG00000034945* and *ENSBTAG00000037605* stand out, each associated with five distinct transcription factors.

While the present study did not investigate the structural consequences of the identified variants, polymorphisms within *BoLA-DQB*, *BoLA-DQA5*, and *BoLA-DRA* may influence antigen presentation by affecting peptide-binding specificity, heterodimer assembly, protein stability, and interactions with T-cell receptors. Future structural and functional studies will be required to elucidate the biological impact of these variants on reproductive success in cattle, such as knockdown studies. Although no functional studies have specifically evaluated the knockdown of *BoLA-DQB* or *BoLA-DQA5* in bovine endometrial or trophoblast cells, RNA interference has been successfully applied in bovine trophoblast cells to investigate placental gene regulation of the genes *CSH1*, *PRP1* e *PAG1* [64], and similar approaches have recently been used in bovine endometrial epithelial cells to study genes involved in uterine receptivity (e.g., *HOXA10* [65]). Therefore, future studies investigating *BoLA-DQB* and *BoLA-DQA5* knockdown in bovine endometrial or trophoblast cells would be valuable to clarify their functional roles during early pregnancy.

Although these genes have been identified as associated with reproductive traits in the original studies, the specific biological mechanisms through which they influence reproductive processes remain to be fully elucidated. Future research should further investigate their functional roles in reproduction, for example, employing regulatory genomics approaches, such as ChIP-seq and related assays, in order to validate transcription factor binding and to clarify the molecular mechanisms linking these loci to reproductive success in cattle. Despite some limitations, particularly the scarcity of functional studies confirming the mechanisms suggested by the associations reported herein, our study contributes to a better understanding of the relationship between the MHC gene complex and cattle reproduction. Similar patterns have been reported in GWAS and transcriptomic studies across cattle breeds and other mammalian species.

Lastly, although additional records were initially identified, only four studies met the inclusion criteria for the systematic review, highlighting the limited number of studies in this area. Nonetheless, this limitation is not uncommon in specific areas of cattle research, including reproductive traits [66] and tick resistance [17]. These findings underscore the need for further research in the MHC genomic region in cattle, which has been more extensively characterized in other mammalian species, especially humans.

Future research should prioritize experimental validation, evaluations in different cattle breeds, and the integration of genomic, proteomic, and other omics approaches. This would help to better elucidate the relationship between the expression of immune genes, especially MHC genes, and reproductive efficiency. In addition, a deeper understanding of these molecular pathways may contribute to the development of reproductive biotechnologies and genetic selection strategies aimed at improving fertility and overall reproductive performance in cattle.

## 5. Conclusions

Our findings suggest that MHC genes are possibly associated with biological pathways related to reproduction and embryonic development in cattle. Beyond their established role in immune response, these genes and surrounding genomic regions may contribute to reproductive processes, including fetal recognition, pregnancy maintenance, and embryonic development. The analysis of transcription factors also revealed candidate genes associated with reproduction, supporting the relevance of these biological pathways to fertility. The results obtained from both DAVID and TFM-Explorer, regardless of the approach (GWAS or transcriptome), suggest biological support for the associations observed in MHC regions, providing preliminary insights into the potential role of MHC genes in bovine reproduction. Nevertheless, further research, including functional validations, is necessary to clarify the biological mechanisms underlying these associations.

## Figures and Tables

**Table 1:** Terms and genes grouped in the gene ontology analysis of major histocompatibility complex genes and adjacent regions for the top 1 functional cluster (enrichment score = 8.42).

Main Term	Grouped Genes	*p*-Value *
Peptide antigen assembly with MHC class II protein complex	*A0A3Q1M088_BOVIN*, *E1BLZ1_BOVIN*, *F1ML60_BOVIN*, *BoLA-DQB1*, *LOC100848815*, *BoLA-DQA5*, *BoLA-DQB*, *BoLA-DRA*, *BoLA-DRB3*	7.2 × 10^−16^
Antigen processing and presentation of exogenous peptide antigen via MHC class II	*A0A3Q1M088_BOVIN*, *E1BLZ1_BOVIN*, *F1ML60_BOVIN*, *BoLA-DQB1*, *LOC100848815*, *BoLA-DQA5*, *BoLA-DQB*, *BoLA-DRA*, *BoLA-DRB3*	2.2 × 10^−15^
MHC class II protein complex binding	*A0A3Q1M088_BOVIN*, *E1BLZ1_ BOVIN*, *F1ML60_BOVIN*, *BoLA-DQB1*, *LOC100848815*, *BoLA-DQA5*, *BoLA-DQB*, *BoLA-DRA*, *BoLA-DRB3*	2.5 × 10^−15^
MHC class II protein complex	*A0A3Q1M088_BOVIN*, *E1BLZ1_BOVIN*, *F1ML60_BOVIN*, *BoLA-DQB1*, *LOC100848815*, *BoLA-DQA5*, *BoLA-DQB*, *BoLA-DRA*, *BoLA-DRB3*	4.2 × 10^−15^
MHC II	*A0A3Q1M088_BOVIN*, *E1BLZ1_BOVIN*, *BoLA-DQB1*, *LOC100848815*, *BoLA-DQA5*, *BoLA-DQB*, *BoLA-DRA*, *BoLA-DRB3*	4.8 × 10^−15^

* Based on *p*-value ≤ 0.05.

## Data Availability

The authors will provide full access to all relevant datasets to interested researchers in order to ensure transparency and reproducibility.

## Supplementary Materials

The following supporting information can be downloaded at: https://www.mdpi.com/article/10.3390/genes17080895/s1.

## Abbreviations

The following abbreviations are used in this manuscript:

MHC	Major histocompatibility complex
BTA23	*Bos taurus* chromosome 23
BoLA	Bovine leukocyte antigen
GWAS	Genome-wide association
APCs	Antigen-presenting cells
