# Supplementary material for: Major Histocompatibility Complex Genomic Regions as Candidates for Reproductive Success in Cattle: From GWAS to Post-GWAS Analysis
Source: Genes (Basel). 2026 Jul 30;17(8):895. doi: 10.3390/genes17080895 (PMC13511974; doi:10.3390/genes17080895)
Supplement: Supplementary file 1 [file genes-17-00895-s001.zip › Supplementary File S1.pdf]

Supplementary File S1: FASTA file containing promoter sequences of candidate genes from GWAS and transcriptomic studies.

```
>IER3|hg19
TCTGGAAGAGCTCAACATTTGTTGGAGGAAACGATCTTTTGAGATGAACTTGAGACTTTCACGTGTCTTG
TGGCTACACCACGTCCTCCTAAGAAAAGTAGCTGGAGATATCTTAGGCTTGGGTCACCCTGCGGATGTCC
TTACCTCCCCTACTTCCCCTGCTGGGTTAGGATCTTTGACCCAGCTAGACACATCTCCCAAGGGCTGGCA
AGAGGCCATCGTTGGCCAGAGGTGAGACTACCCAACACGGCTGCCCAACAAGGTTGTTCTACCTTGGGGC
ATGTGTGCATAGCCCATTCAGAGTTTGAAAGTGAGACCCAAGAAAGGAGGCAGACATGAGAGCAGCGTGA
AACCTCAAAGACACAAGAGCCAGGGCAGTGAGCCAGGCGATCGTGTTAATAATGAGGACTGTTGAGAGGC
AGGGAAGTGTAGAGTTTGGCTGCTTTGTCTCTGGAGCCAACTTCCTGGAACTCAATGCCAGCTCTAGCCC
TCACAGCTGTGCAGTTTTTGTGCTGTCTCCTCGTCTATGAAATGGATATTGTAAAGGCCTTCTTCTGAGA
CTTATCACGAAAATTAGATGAACTAGAACATGAAACTGAACGTGAACATGGAACTGATTCTCCAGCTTCT
ACTCTCTGATCGATGTGGTACAATGCTGGATTGTGGGATGAGCTAAAGTAGAGTCTGAATCTAACTCCCA
CTAGGTAATCTTGAGAAAGTGAACTGCCTTATGGAACCTCTCAGTTTCTTCTACGGTAAAATGAGGAGAC
AGACAAGGATGACAGTGGGGGTTTGTGAAGGTTTAATGGATTGATTCCTCGGTAGTGTTCAGGACAGTGC
CTGGGAAGCACTGGGGGGCCCAAACAGTGTGGGCCCACGTTCCTTTACACTCCATCCCAGGGAGTGCAAA
GCCATGTGGCAGACACAGAGGCTGGGGAGAGCTACTGAACCACAGCGGGGTCTGGGAGGCCGGGAGGCAG
GAGGTGCTCCTGGAGCTGCTTCAACAGCAGGGTGGCCCTCAAATCCACTGTGGCCTCCTGAGGACATGAC
AGGCATCTCAGGGCGCAGCGGGTTCTGGCCCATCCCTGTCCCCTGACGGAACCTGTGTGTGAACCTCTTC
CCTGACAAGGAAAACATTTTGACTTGGCAGATCTGATGCAAGAACTTAAACCAGGGATGTTTTGATGTAG
TTTCTTAGAACAGCCCTGTCAGACTGTAAAATGGCTAATGATGGCTATTTCTTTCTTTCTTTTTATTTAT
TTATTTTTTTTGCTGCACCCCACAGCATGTGGGACTTCTGTAATCAGGGGGACCAGGGATGAAACCCATG
CCCCCTGGAGCGGCAGCATGGAATTTTAACTGCTGGACCACCAAGGAAGCTCAGCCATTTCTTATAAACT
TAAATATATCCTGAGTTATACTACTCCTAGATATTTATTTAACAGAAATAAAAACACACATCCACAAAAA
GAGTTGTATATGACTGACTGCTCATGGAAGTTTTATTTAGAATAGCCAAAACCAAGGGGGAAAAAACACA
CAAAAATGTCCATCAATGGTGAATGAATAAGCCAACTATATCATACTATGAAATACTATTCAGAAATCAA
AAGGAAAGAACTACTGATTCAACCAAGAAAATGGATGAATCACTGAAATACTGAGCAAAAGAATCTAGAC
ACAAAAGAGTGCCTACTGTAGTTTTATTTATATGACATTCTAGAACAGGCAAAACTACACTGCAGAACTC
AGATCAGTGGTCACTAGAGGCTAGGAGTGAAGAAGGGTGTGAGGAACTTTCTGGGGCAATGGAAATATTC
TATATCATGATGGTAGTGGCATTTATACAACTATATGAGTATGTCAAAACTCATAAAATTGCATTCCTAA
AAAGGATAGATTTTACTGTAAAAGAAGCTATATTTCAATATGTTTTTGTTTACTTGGCAAGTTGTATCTG
ACTCTTTTGCAACTCCATGGACTGTAGCCTGCCAGGCTTCTCCATCCATGGGATTTTCCAGGCAAGAATA
CTGGAGTGGATTGCCATTTCCTTCTCCAGGGGATCTTCCTGACCCAGGGATGGAACACAGGTCTCCTGCA
TTGGCAGGTGGATTCTTTACCTCTGAGCCACCTGGGAAACCATATTTCAATATACCGGGCATTAAAAAAG
AAAAAAGAAAATGTAGGGAATTCCCTGTAGGTCCAGTGGTCAGGATTTGGTGGTTCTATTGCAGGGGGTT
CAGGGTTAATCCCTGGTTGAGAACTAAGATCCCCCATGACACACGCAGTGCTTCCCCCCAAAAAGTACTA
TAATCAAGCACTACCAAATTCTCTGTGAAAGGGTAGGAGTCATAGAGACAGGATGAGCACAATCCACAGG
GATGGGCTGAGACTATCTTGTTTATAATTACTGATTTTATTTATTTGACTACACCAGGTCTTACTTGCAG
CACTCGGGATCTTTGACCTTCGATGCAGCATATGGGGTCTTTAGTCATGGCATGGGAACTCTTAGTTGTG
GCATGTGGGATCGAGTTCCCTGACAAGTGATCAAACCCAGGTCCTGCATTCGGAGCCCAGAGTCTTAGCT
ACATGACCACCAGGCAAGTCCCATTTTTACTGATTTGCTTTAAATGCTGACAATTTAATAACTTTAGAAG
GCCTTGTCAAAAGGTCCCTGGCATCAAAATGGCTGCATCACAACGCAAGTGCACTTCTAAGCAGTCAGAA
AGATCTACAGACAAATGTCTATAATATACTGTGTTCGATGCTTCAAGACATTTATATGAAAGTATTAATA
GACGGACTGGTCTACACCAAGGAGTCACCAGTGGGGTTTTCTCAGGAGGATGGAGAGAAAACAGCACGTG
TGAAGGGTCTAGTGGATAAGAGTCTGGCCTGAATGAGAAAAAGTACAAAATCGTATATAGTGCCCTAGCT
CAGGGTGTAGGGAAAGCAGTGCGGGAGTTGAGAGAGAAGCCTGTGAAGCTGCCTGGATTCAGGATGGGAG
GCCAGTGTCAGCCCCACCAGAGAGTCTGGAGACACTGAAGTCACCCACCCAGACAGTGAATTGGTCCAGT
CCATCTTTTCATAAGAAAGCTCTGGAGAATGCACAGAAGATGGAATGCAGGGAGAGGAGTTAGAAGTGTT
CTCTGCAGCCCGTCCTTGTCATCATTTCCATTGAAATGAAATGTGCCTTTCTGGTTGTGGACTTTCTGAA
TACCAAAAGTATTAGGTAAGTAGGTCTCAACCAGGGTACATCGGCCCTGAAGGTGGCTCAGCTGCCCAGT
ACTTTGGGACC
```

>FLOT1|hg19
GAAGATCCCCTGCAGGAGGAAATAGCAACCCACTTCAGTATTCTTGCCTGGAGAATCCCATGGACAGAGG
AGCCTGGCAGGCTGCAGTCCATAGAGTTGCAGAGTTGGACATGACTGAAGCAACTTAGCGCACACACACA
CACACTGGCCCTGTTGAATGCTCAAAAGATGTTAAATTCTGTGTCCAGAGCAGCTGTCTCTAAGGCAGTT
ATGAAAATGTGTCCATTCTCTCTTGTGTTATTATCATCACTACAACATTTGCATAATGTCTCACAGTTTA
AATGTTCTTCATATAAACTGAAACCCAGGTGGCTGCTCACTTCTTGCCTTTGTGTTTTGTGGTCCTCTGC
TCCAGTCATGCACGCACATTTCTGCACATCGTGGGGTTGCCCCTGTGCAGTCGCTGCACTTTCGTTCTGT
GCATGTGTGTATGTAATATGCATGTTGAAGGGGGACAGAATATGCCACCCCAAAATGTGCTCCTCTGGCA
TGTGAATTATTTGGAGCAATCAAGATCCAGCAGAATCAAGAGAAGCCTTGCACCTCTCATTTCACGGCCT
AAATAACTGAAGTAGGGGGCCTGGTCCAGCGATGACATTGATCGGAAAAACTTAACCTGCTTGGCAGGAC
AAACATCTCATTAACAATCACCTGCTCTTCCCATCTTCCTGTGAATTACCCACCTCCCCTTCAAGCCCCA
GGCCTCTTTCCCATTCCTTAGCTAAAGATGGCATATAAACTTCAATTGCCTGACTGCCTTTGAGTCTCAA
GTTTTCTTGGGCTCCTGTTAAGTATGCAATTAAATTTGTTTTCTCCTGTTAATCTGTCCTAGGTCAATTT
AATTATTGAACCAGCCAAAGAAACTGGAAGAAAAGGAGAAATTTTTCCACCTCTACAACGTGGTGTGTGT
GTGCGTGTGCGAGCTAAAGCAGACAGACTTGTGTCTGTGTGTGCTACTGTGTTCATTTCTCTTGGGCTTC
TTGAGTGTTTCTTAATGACTTCCTTCCTAGGCTGCTGTTTTCTCAGTCCCTACAAACGTGTAAATATGCA
CTGTAACTGTGGTTCCTAGACTCTGCTGGATCAGGATTGTAGCAAGAGGGCCAGACTTCCTTATGTAAGC
CTTTCTGCACGTCCCCAGAGATGGGGATTTGTGGCTGAACCGTCTCAAACTGTCTGTGGGCCCCTCTCCA
GCTGCCACATGTCCTGACATGTGCCCGCAGTCTGGGATTTCACCCGCCTCCACTCACAACTCACAACACA
CACTTGAGGAGTGCCTACGGTTGGCACTAGCCAAGTCCCCGCCCCCTCTCTATGCTTAATCTTCGGAATT
TCCAGCCGACCTTCCAACCGCTCCCACCCCAGGGTGGAGTTGAGAAGGGCCCGCCCAGTTTTCGGGGTAC
ATAACTAGCTCTGCGGGACTGCGGGGCCACTCGTCCTTCGGCTCAGCGCAGCGCACAATGTGTCACTCTC
GCAGCTCCCTCCCCACCATGACCGTCCTGCGGGCTCCGACCCCGGTTTCCTCCGCCAGCCCGGGACCCCG
ACGGGGCTCCGGTCCCGAGATCTTCACCTTCGACCCTCTCCCGGAGCCCGCGGTGGCCCCCGCTGCGCGC
CCTAGCGCCACCCGCGGGCATCGAAAGCGCAGCCGTAGGGTCCTCTACCCACGAGTGGTGAGTGTCGCCG
AAGTGGACAATCCCGGGGGAAGAGGAGGGTGAACTACTGGACGCCGGGGCCCCGATCTGACCTGTTCTTT
TTATCACCTCCCCTCAGGTCCGGCGTCAGCTGCCAGTCGAGGAACCGAACCCTGCCAAAGGCTGCTCTT
TGTCCTGCTGACCATCATCTTTTGCCAGATCCTAATGGCTGAAGACAGTGCGTCTGCACCCCTGGCCCCG
GAGGACATCTCCAGCGCGCCGTCCCCCGCGTCCACCGCTGCAACCCCGGTCTTCGAGCCCCTTAATCTGA
CCTCGGAGCCCTCGGACTACGCTTTGGATCTCAGCACTTTTCTCCAGCAACACCCGGCCGCCTTCTAACC
GGGACTCCCCACATCCCCTAAAAGAACCTGAAAAACCAAGAAACACTGGGTGTACCTGGTGCGCGAGAGC
GTATCCCAAACTGGGACTCGCGAGGCAACTCGCACACAGAACACTACGGCTGAGACGCCAGTCCGAGCCT
GGGAGAGACAGGCACCGGCACGCCGGGGCTAGGCCGGGTGGGGAATGAGTGCGTCGTTAACTTATTTCTC
ATTGCTCCTAATAATATTTATGTGTATTTATGTGTGTCCCCCTAGGTGATGGAGGGGTGTATGTAATATT
TATTTTAACTTATGCAGGAGTGCGAGATGTGCACCTTGCTGCAAATGCAGGTCTCTGGGTATTTATTGGG
CTCTTTGGGGTTGGTGGAGGCAAGCACCCAGAACTAAGCGAGGCCGGGAAAAGGGATGGAGGAAACCCGG
GTCGAGAAGGCGCCCAGGCTGGGAGTCAAGGATGGTGGTGGGTCGCAGTGACACTCTCAGTCCTCCAGCA
TCTCAACTCGCGAGTCTGTGAAGATTCGGGGAACCGTTGAGAATAAGGTCCCTGGCCTACACTCTCCTTG
AAGTTGCCTCCAAGGGGGTGGCTGCGGGGTCGGTGGGCTGTCAGGAGGCTGCTCTGGGGTCGCCCCGTAT
GTTCTATGAACACAAATAAACTTGATTTACTGTCAGCAGTCATGTGAGTGTCTCTTTACAAGTGGCGGTC
CCCCATTCTCCTCAAAGCGCCAGGAGGAGGAGGAGCCGCGGCCAAGTCCCCAGCCGGTGCTCCTCCCGGG
GCCCCTGAGTCCCTCCTCCCGGAGCCACGGAACTCAGCATCTTGTGACCGTGTGATGCTGTCAGTCTTTG
CCCAATCTGCGGTTTTTCTCAGTTAGTTGACAAGGGGCAAATCCGACATGAGTCAATCTCCCTCTCCCCG
GGTGCGGGACTCAGGGAAGTCACGTCGCGCGGGGCGGGGCGTGGGGCGGCCTGGGCGGGACTTCCGGCCC
CGGAAGGGCGGCTAAGTGGGGCCTCCTCCCCCTTTCCGGGGCCGCTTCGGCAGCTCTCGAGGGCTGCGGA
TCTGAGCTGGGCCTGCCCGGGAAGGGGCGGGTCGGACGCCGGAGGCCGGGGGTAGGGTGGAGGGGGTGGT
CCTCCGCGGGGGGCGGGGTCGCGTCTGCAGCAGCAGTAGGGTGCGGCTGGGTTGGGGGCGCGGGCCCAGC
TCCCAGGGAGACCCTACACGGGTGGCATCTGCTAGACTCCCAGGTCCCCTACGTAGCCCCGCCCTCCCCG
GGACCCCAGCC

>MDC1|hg19
CTCGCTTCCCGGTCCTCGGACCGTTAGGAGATCCCCTTTCTAAGTAAAATTTTACAGAAACTCGGTTTTC
CCGCCCCCTAACCCTGAAGGGTGCTTTTTACAGCTTCCCCAAAATGAACCTGCCAAGAAAAAGAATTCCT

CATTTTTCAAAAGCAAATCCTAAGGTTCAAAGGTGCATTTATATAGAAAAGTTTTGAGGGGCAAATCTTG
ACTAAGAATGGAAGATGCAAACGTTCTACAACCATCCGTCATAATATACCTGTTTTTTTTTTTTTTCCCT
TAAAAATAAATACAGTCAAAAGAGGTTAAAAATCCTTACCTTGGCACCGATCTGATTGCCACACTGACCG
GCCTGGATGTGCACGATTTCCCTCATTGTTAAAATTTATTCTAATCTTTTTGCTCGCCTCAAAGTGTATA
CGGGGCAAAAAAATATTATGGAATTAGTTTTTCTCTGCTGCTAGTCGCAGGGTGGAAAGATGGAACGGGA
CCCGGAGGCTGAAGCAGCGAGGTCCGAACGCGGCGGCAGGAAGGTTCTGAGAGGGAGAAAGGAGAGGGGA
GGGCGCGGGAGGAAAGGCGGGCAGGGCGGGGCGCGCGTGCGCGGGGGCCGGGAGGCGGGAGAACTCGCCG
CGCGCCGCGGTGGAGAGACAAAGCCTCACTAGGCTGGCCCTGATTAGTCCGTGCCGGTCCTGTATCACAC
GTCCATTGGTCCCTGCTGGACGAGCGCAGTCCTGCCTGGGAGTTGTAGTTCTCTATTGTTGTCCACGCTG
CAAAATGAAAGGACGAATGGGTGGGTACTGGGTCTGGGGGTTTTGGTTTGGGTGTTTTGACTGAAAAACG
GCCTGGCGCTAAGGTTCCTTCCTCTATGCCAGACTCTTCTGGTATCAAAAGAGAGTAGTTTTCGAGGGTA
CAGGAATTGGGAACCCTGGTGTGCCAGGCTTTGGAAGGTGGAGTGGGGGCACGCCCTGAAAGCTCCCCGC
TGCGGTAGCTCTTGGGAAGACAGGACTGCTTAGCTCAGCGAAAACGGGGAGAAATGCGGCACCCTGATCT
GCTCTCTAACAGCTGGTCCCTGGTGACCCAACTGGCATGAACTCCAGTAAGCTGCGGCTCCCCCAGATAC
TTCGGGAAGGAGGAGGGCATTGCCTTCCTGCTATTTCCTTGGGAGCTTTCTAATGATAATATTTAGAAAG
AAACAAAATTGCATCAAGGACTTTTCAGTATAAAAACTAATTGTCTGGAAGGTATGTTCAAGAATAGAGA
AGCAGATATGCCGGAAGTCTCCGTCCATGTGGGGATCTGCGCCTTACAGCCGCATTTAACACACTGTTAA
GAGGCGAACTGTGGGGTGAGATACCTGATAGGCACTTTGATGAAGTCAGTGTTTTTTCAATTCGCTGATT
TCACTGAGAGGTGGTTGACTATTGCAACCTTTCAAGAACCTCGGGATTAAAATAAAAGGGGTAGGGAGGG
GGATGGGAAAAAAATTGGATGAGCTCAAATCTGAGTGGCATGTTTCTTCGCAGGGATTAATTCAACTTGA
GAGATATTAAATGTCTACCAAACCAGGAGCTAGGAATACAAAAATGATTAAGGATTAGCTCACTGTTCTC
CAGGATCTTGCAATTTGGTGAGAAGTGTATAAACAAACACTAGAGTGTGGTAAGCACCATGATAAAGGAC
ATGGGATATCAGGCTCCCTGAGTGTGTGCTCAGTCATGTACGACTCTTTGCGACCCCATGGACTGCAGCC
TGCCTGGCTTCTCTGTCCATGGGAGTTTCCAGTCAAGAATACTGGAGCTGGTTGCCATTTCCTTCTACAC
GGAATCTTTCCGGCCCAGAGATCGAACCCGTGTCTCCTGCATCTCCTGAATTGGCAAGAGGGCTGTTTAC
TTAAGAGGCACTTGGTTAGATGATAGCAAGGTGTCAATTCACACTGGCTTCAACACACATTTCTCGTCTT
CTAAATAAAAGAACATAAATTTCTCTAAGACTCGTCTACCCAGTAAAGGACTTGCCTGGTGGCTCAGACC
GGTAAAGCATCTGTCTACAATGCGGGAGACCCGGGTTCAATCCCTGGGTTTGGAAGATCCCCTGGAGAAG
GAAATGGCAATACACTCCAGTACTATTGCCTGGAAAATCCCATGGACAAAGGAGCCTGATAGTCTACAGT
CCATCGGGTTGCAAAGAGTCGGACAAGACTGTGCGACTTCACTTTCACCCAGTTAAAACGTTCATGTTTG
AAGGCACAATCACAGTGAAAGTAGTTATGTATTTTGTGGTAGTGAAGAGAGTTCAGAATTCACGATTAAA
CTGTTTCAATTTGTCTCTAACAATAGCCGTAGGAGCCTAGGCAAGCTAACTCCGCAGAACTCTTATTTCT
GGTCAGAATGAAGTGAGAATACGAATGCAAAAGGTCCTCCTAAATTATTCCTGTGAAATTGTCTTGTCTT
AGTATGAAATTTAGCAAAATAAAAATTATCATTCTGTAGCCCTTCAAAGACCTCGGTCAGCCTTTAATAT
GGTTAACAAAAGAGTTCTCTCCGTTTGCCTAGATATCTTTACTCTAGGAAAATGACCTCCAAAAGTGTCC
ATCAGGAATGGATTCTCAGGGAGGAAATGACATGACTTCTAGATGAGTTAAATCAAAAGGAAATGAACTT
CCATTGTAGTGAGTCCTCAAGCGCCACGCCTACGGGCTATTCTCCAAACTGAAGTCTTCAGCCACGACTG
CAGCCCGCAACCCTTATATTTCTCATGAGTCAGCGGACACCATTTCTGGGAGGACCGAGAAGGCGCTCTG
GCCGCACTACATGCTTAGGTCGACGACTGTGATAAGCCCCTCTATCCGCTGCTGGCATCCAGACTCTGTG
GTCGCAACTTGGAAATATACGCGGGAAGTGGGTGGTGAAACCGCAGTGGGTTGTCCCTTTGGAGCTGCCC
AATCGAAGTGCGCCATTCAGTGCGCCTCCGCGGCTTCCGATTACCGCCTCGCCCAACGGATCTCAGTCTC
CCCGGAGAAGCCGCCCCTTTGAGCCGTAAGGGGGCGGGTCGGGTCGGGTGGGGCTCCGGCTTTGCCTCCG
CGTTGGATTGCTCCGTCTGCTTGCCGCAGGGCCGAGAAAGACAAAGGCTTCAGCCAATCAGCTAATCGGC
CCCTTCCCCGCTCAGGCACTGGCACCGCCCCTGTCGCAGCCGTTACTGGTGGCGCGCGCGGGGACTCAAA
GTAGGTGAGTTCTGGGGGGCCTGGCCCACGACTCCCCGGATGCCATCTTGGTTCTCCCGGAGGGCGGGAG
GGGCGTGCCTT

>NRM|hg19
ATAGGGAGATTGGGAGCCATGGGTTTTCTTAAATGGCCATTTAAAATTGGCCAGTCCCAGGCTCAGATAT
CTTAAGTCACTGCTCATATTTAGCTGGCCGGATGTACCGATTGCTTTGTGGCACACACAGTGTCCTGCCT
CCTGTTTGGAAGCCATTTACTTCTCCTCATCTTTTCTTTCTGGGATTCTTACTCATCCTGTACAAGATAG
TGAAAACAATTTTAGTGTCAGGAACTTGAAAAGATGCTTGGAGTGAGTAAGTTTGAGGGTGGAGTTATGG
AACGTCCCTAAAATATTTTCCTTTCTCCTTACCCCATCTTGTTAGAAGCATCCTTTAGCTTTGACATTGA

GCTAATCTCTGCAATTTATCTCTTTTTTTTTTGGCTTGCTTTTCTAGTATTTATAAATTTAAAGCCTAGG
CTTAATGTCTGTGATAAATAAATATTCACTCTGTGCCTTATGGTGTAGTGCAGTTTTATTTTGTAAATCT
TAAAAGTTGGTTGATTATAAGGATTTGAGGAAATTAGAACCCATATGCATTGCTCATGGGAATGTAAAGT
GATTCATCCACTGTGGTAAGCAGTTTGACAGTTTCTCAAAAGTTAAATATAGAATTACCATATGATCCAG
CAATTCTCATAGATACACAGCCAAAAGAATTAAAACAGATGTTCAAACAAATACATCTGCACAAATGTTC
ATATCAACACTTTCGCAAGAGCCGAAAGATGAAAGCACAAATGTTCATCAGCAGATGAATGGATAAACAA
ATTGTGGCATATCCATACAATGGAGTATTATATTCAGTCATAAAAGGAGATGAGGTACTAATTGTGCTAC
AGTATTTTGAGGATGAACCTCAAAAGCATGCTAAGTTAAAGAAGTCAGACACAAAAAGTCACATTGTATG
ATTCCACCTGTTGGAAATACACAGAATAGGTAAGTCCATAGAGACAGTAGGCAGTGCTTGTCAAGGGCTT
TCGGGGAGAGAAGATGGGGAGTAATTGCTTAACAGGTTCATGGTTTTATTTTGGGGTGAAGAGAAAATGC
TTTGGAACTAGATAGAGGTGGTGGATGCATACATTATGAATGTACTAAATGCCACTGGACTGTTCTCTTT
AAAATGATTAATTTTATGTTATGTGAATTTCACTTCGATTAAAAAAAATAGATTGGTTTTGTGCACTTTT
CTGCAAGTTAAGTTATACTTAAGTTTGGAAAACTTTGGTGTAGTAGAAACAGCATTAGCTTTGCCAAACA
CTGTGCTAGCTTTTAAACATATCCAATCTTGCGACTTCTGTGGTGGCCCAGCGGTTAAGACCCTGTGTTC
TCAATGCAGGGGGCATGGGTTCGATCCCTGGTCAGGGGACTAAGATCCCACATGCTGCTGGCCAAATAAA
ACATAAATTAAAAAAATTAAACATACCCAACCTCACTATATATTAAGCACAGCCATTGTGGGTGTGGCCC
ACAGTAGGACCTAGATAAATAGTAGTCCCCTTGCCCTACCCAAAGGCCTGTATTGTCTGCTGCTGTCTGA
GTCCTTGAGGTTCTGGATTTCTTACAGCACCTGCTGCCTCTATAGCCCCGCTTACCACTTATGTGCACAT
CCACTTATGACTGGTAACATTTAAGTGCCCCAATTCAAATTTCTAGAGAAGACATCTTTTCTTACACTGC
CAATGAATCCTACAGCTCATACGTAGTACATCGTGTAATGTCAATCCATCAGGATCCTAACAACAAACGG
CACAGTCAAGATAATTTGGAGAGCGTTTAATGAAGTGAAAATTAGGAGGCAGAGTGTAGAGCAATCACAA
AGAATATTGCAGTATCCCAGTACCCACAGGTCTGTTGTGACCACCTGTGAACCCAAAAGGTTTGCCAAGA
CAGAACAGTTATATAGAGAGGCTTCCCTCTAAAGCTGTGATGTTCTTTGATGAGGTACATTCAGGACAGC
TTCCGGAGGCAGAAAGCAGGATGGAGAGTGAATCAGGAGGGAAACGTGCTGTCTGGTAGCACAGTTCACA
ACAGCTTTGATTCAGAGGAGAATGGGAGTATTGAGTCTGGGAGTGGAAAGGAATGTAGAGCCTGTGGCAT
GCTGTTGGGCAATTTTGTCATCCCTGAGCCCACAGGACTAGCAGTTCCTTATCTTAGGAAATTTTCTTTA
TCTCTTTAGGCCTATGTTTCGTCTATCATCTGAAAGAATTAGCTTAACGATCTCTAAGGTCCTTACTAGC
TTATGCCTAGGTCCTTAGCTACATGATATTTAGTGGAGAAAAACCTTGCTTTTATTTTGATTTTTAGATG
TGAATGTTCGTCCGTAGGTATTACAAATAGATCTGTAGGAAAACATTTTGGACAAAAGATCTTAGTGCTT
TCTGACTAGTAAGTGGAGTCAAACTAATGAGGTTTCAGACTAAAGTCATAGGTAGTTAATTATCACAAGT
TTAACCTACTACAAAATTTACAACTATAACAAAGTTTTCTATCACAAATATTTTAACATATTGTTACACT
CATGGTTACAAAACAGCCACCTTTGGCATTATTAGACCCAGTCTTTCAGTGTTACAGACAAGTTCTCTGC
CCTTGGCAAGTGGAGAGGCAACTTTATAACACATTACAGAATAAGCATTAATTTGTGGTAAAATAGTCAC
TCTTTTTCACCTAACAAGCTGCAATCGGTGATTCAGAGCTTTGGGTTCTCAGCCTAGGTCAACAGTCTTC
CGTTCTCTCACCGCTCATTACTAAAGTGTTAACTGAGGCTTTGTAACCTCTGACTCTGGCTGTTTTCAAG
TCAAAGCTGTTAAAGGAAAGGGGGTTGGTTCATAATGCTATTTCTGCAAGAACCCCGCAAGAGGGAAATA
GTCTTCATTTTGAATGAGAAAAGTAACCCAGCTGCGATTCAACCCACGTGAGTGACTGCACAACCTCTGC
CCTTTTCATCCCTTACTCAGGATTCTAACACTCCCTTCTGTGACGCGGAGATCGTCGGGTGGGGAAAGGA
ACCCAGAGGAGCTGCTCCGCAGCTGGGTGTGAGCGAGCTGCGCGCACCGGCCGGAAACCCGGCGCAGGCG
CGCGGGAGCCCCGCCCCACGCCAGGAAGGGTTCCAGGCTGGGGGCGGGGCCGGGGCGGAGCCTGCGGCCG
CGGGAATCCCAAACCCGCCCCTTTCCCCACCCCTCCACTTCTCGCCATGGCCCCTGCACTGCTCCTGATC
CCTGCTGCCCTCGCCTCTTTCATCCTGGCCTTTGGCACTGGAGTGGAGTTCGTACGCTTTACCTCCCTTC
GGCCACTTCTT

>PPP1R18|hg19
AGACAGTGTTTCCCACAGTCTTCTTGATCTTTTGTACCTTTTTCCAACTCCCGCTAGATGCCCGCCAGGG
ATGGCTGGCTGCCCTGCAGGACCAAAGCATCCTTGTCCCGCTGGTTTGGGATCTGGGGCTCCTGTTACTG
TTTGTGGGGCAGCACAGCCTCATGGCAACTGAGACCGTGAAGGAATGGATGTCCCGGTACTTTGGGGTCC
TTCAGAGGTCACTGTACGTGGCATGCACTGCCCTGGCCTTGCAGGTATGAAGCCCTAGCAGCTGAGTCCT
CAAGGGAAACAGCATGTGTGAAGGATGTCATTGATTGGAGCTGCAAGAACCTCAGGCTTTGGATAAAGTG
TGGGTTTAGGAGGAGAACTGAGGGTGTTCAGGACTAATCTCAGCTCCCATTCTTTCACAGCACACATTCT
GTTTTTTATCAAGAAGTATGTACTGCTTGTTATCCACTGGGCACGATGTGTGCATTCTCACCTTTGCAAC
TTTGTTCATGTTGTCCCGCTTGAGAGTGCCTTGTTAACACCTCTTCAGTCTTATCCTTTCTTCAAAAGCA

CTTCCCTAGATCTCAAGAGAAAGCTTCCCAGTTAGTACTCACCTGTCTTCTCAGTGGGTGCTTGGCATAG
ACTAGTGTGCGTGCCTATCTGGTCACTCTCATCCATTCTCTAAGTCCTCAGAGTAGAGACATGTCATTCT
TACATCCTTAGGACCCCACCCAGCACCCTCCACATGGTGAACATGCTCATGATAGAGGTGGGGAGAACAG
CCGGCCAGCCTGGACTGGGGGCAGCCCCTGGGTCTGGGTCTGTCAAGGGTGGGCTGCTGGAAAACCCAGT
CCCTCTTCCTTTCTCAGCTGGTGATGCGGTACTGGGAGCCCGTGCCCAGGGGTCCTGTGTTGTGGGAGAC
TCGGACTGAGCCGTGGGCCACTTGGGTGCCCCTCCTCTGCTTTGTCCTCCATGTCATTTCCTGGCTCCTC
ATCTTCAGCATCCTTCTTGTCTTCGACTATGCAGAGCTCATGGGCCTGAAACAGGTGAGGCTCCCAGATC
CATACCCAAGACATTTGATCCTTTCTAGAATACCTCTTTCCCCTCCAAGGATGTCCCTCTGCCTCCCATT
GTCTTCCACCTCCCTTTCTTATAAGATAGTCTCCCCCCGGCCCTCCCCCAAAGGCTAGGAATCATGGGCT
TCTTGGGCCTGGGAAGGTTCATGGGCCAGAGATGAGGGTCAGGGACCCCCAGTCTCAGAAGGGTGGTTCC
TGAGCCTGTCATAGAAGGAGGGTGCCCAGAAGGGGAGGAAAGAGGCAGTTAAGTTGTGACAAGGGCTTGA
CTCCATTTCTAAGCCATTCTCCCTCTGCTAGGTGTACTACCATGTGCTGGGGCTGGGCGAGCCTCTGGCC
CTGAAGTCGCCCCGGGCTCTGAGACTCTTCTCCCACCTGCGCCATCCAGTGTGCGTGGAGCTGCTGACTG
TGCTATGGGTGGTACCCACCCTGGGCACTGACCGCCTGCTCCTTGCTCTCCTCCTTACCCTCTACCTGGG
CCTGGCTCACGGACTGGACCAGCATGATCTCCGCTACCTCCGGGCCCAGCTACAAAGAAAACTCCACCTG
CTCTCCCGGCCCCAGGATGGGGAGGCTGAGTAAGGGGTTAACCCTGGTTGCAAGCCCTGTACTTCCTCTC
TCCCTCCTATCCCAAATTCCCTTAACATCCAGGCCTTGACTGCTTCAGACCAGAGGCCCAAATCAATGGA
CTGAAGGAGTGGTCCCTTTCCTTGCTTGAGCCTCCACTCCTTGGTTCCAGCTCCATACTGAATTTCAGCC
ATTGGACTCCAAGGGCCACTTCTCACCAGCCAGGAAGGAGTGGCTGGGAACTTATCCCTCTACTTACAGT
TCAGGGCTTGACCCTCCATTAACAATTTCCTCACCAAGAGAAGAGTCTGGCTTGACCACTCCCTTAGCCC
TGTTACTACCCTCTGCGCCTCAGGGATCCTTTCTCCACGTCTAGCTCCCTCCCAGGAGCTGGACCAAGGC
CCACTGTCTGGCCGTTCCAGGTCCCTAGGCCCCGCCCCTAGGCTCCATCCTCCTGAACCTAGTTGAGCGG
TTTCTCCCGCTCTTAGCTAAGGTGACTAAAGGCTCCCTGCTCTCCCGAGGAACTCGCGCTACAGGAAAAT
AAAATTCAGTCTGGTTTTTCTACGTGTAGAATGCAGTCTGTCAGTGTCTCTCAAAGCTGCAGGGCTGGCT
CTGGGAAAGGCGAGTTACATTGGAGTCTGGGCATCGGCCGCGCCTTCTGGTGACCGCTGGGCGGCGGCCA
CTCCCCGCGGACCCAGCCCTCCACCCCCTTCCCGGAGCACCCGGTCCCCTTTCCTCCAAATCTCCCAATT
TTCTCGCTGCAGACCCGGGTGGGGGACGGCTGAAACGGAAGAGTGGGGCAAAGGTTCGGGTAAGGGAGC
GAGCTTCCGAAACCGTTGTCTTCCCAGCCCTGCTCCTGCGGCCGCCCCCTCCCCACGGGCCCCGCAAGCG
CCCCCACGGTAACAAAGGCTGGCCCGGATAGGCAAAAGCGTGGGGAGGAGCGGCAGGAGATGGGAAGGGC
GGGCCCGGCTCCGAGCAGCTGCCGCTTCCTCCCCAAGTCCCTCGAGGGGTCTGAGTCACGGGCCGCCGCC
CCGGGTTGGCGAGCTGGGGGTGGGGGTCTGGACACCTGGGTTGTCCAGGGTTACCGGGCGGGAGCCGCGT
GAGAGGCCCTGGGGAAGGACCGCGGAGGGCCGGGGATCGCGGCGGGAAAAGAGTCCCGGGGCCGAGGCCC
CAAGAGGGGGACCCGGAGAGCGAGGCTCCGCCCCCGCGCCCGGCTCCGCCCTGCGCCCCCTTCCCTCTCC
CCATTGTCCTCAGACAAAGCGGCGGCCGCCCCCTGCCCGGCCCCCGGGTCTCAGTCTCCGTCCCTCCCCT
TCTCCTCCCTTCCCCCGTCCCGCGCCCCCCCCCCCCGAACCCCCCCCCCCACCTTCGGCCCCGGATCTC
CCTCTATCCCTCTCTCCTCCTCTTCCTCTCTCCTCCTCTCTCCGGACGTCAGGCTCCTTCGCTCCCCCTC
CCCCGGGAGCCCCGCGGCGTGTGAGTTGACCCAGGGGCTTCAGACTTGGGGAGGGGGTGTCCCCTCCCTT
CCGTCCCGGGCGCGGACCTCGGGCTGTCTCTCCTCTCTTTGTGCCGAGATTGCCCATCAGTGCGGTCATA
GCCGGGTGGGGACGGCTGGCGAGGTCGGGGTTCCATGAGCGCCAAGACCCCGGCGCAAGACTGGCCGGCT
CCGGAGCGAAG

>DHX16|hg19
CCAAGGACCAGCAGGAGAGGGACTGGGGATTTTTTGTTGTTGCTTTGGTTTGGTTTTGTTCTTGCTGTGC
CTCTCAGCTTGCGGCATCTCAGTTCCCCAGCCAGGGACTTATCCTGGGCCTCCATGATGAAATCATCAAA
TCCTAACCACTAGACCACCAGGGAACTCCCTGTTTTGTTTTCTCTCTTTTTTTTTTTAATTAAAAAATTTT
TGGACTTCCCTGATGGTCCAGTGGTTAAGAATCTGCCTGCCAATGCAGGGGACACAGGTTTGACCCCTGC
TCCAGAAGATGCCACCTGCTGCAGGGCAACTAGGCTGGTGTACCACAAGTACTGAGCCCGGGTACCTAGA
GTCCGTGCTCTGAAACGAGAAAAGCCACCACAATGAGAAGCCCTCCTGCCACCTCAGCAAAGAGGAGCCC
CGACTTGCTGCAACTAGGGAAAGTTCATGCGCATTGAAGACCCAGTGCAGCCAAAAATAAATACTTTTAA
ATGATTAAAAAGTAATTTTAAAAATTATTATTTTTGTAAGTCTAGGGCTGGTGTGAGGAAGGACAGTTGG
AGCACCCACACACTGGACATACCATGATGACAAACAGTCAGCTGGGTTCACTGCGGCGTCCGCATGCCTG
GCACCTGATAGACACACACAATTATCTTTAGAATGAATGAACGAATGAGCAGATGAAAGGAGATAAAAAT
TTGGAAGCAGAGGACACTCTGAATTGAAGGATTCTGGTATTCAGAAGCCTAGTGCAAGGCAAGCAGCCAG

AGAGTGTATGATTAGAAGTGGCTAATCCAGGGGATTTTACTCCAAGACTCAAGACCGTAAGTAATAAATC
TCTTGAAACTGCTGCAGAATAGTACAGAGGGGCAGATTCCTGAAAAAGTTCTCCTCTGATAGACAACGGG
TTTACATTCCTAAAGTTTTAAGAACCATAGATAATTCATGGCTTTGTGGCCCAAGTATAGTTGGCACTGG
GACACTTTCCCGTTTCGGTGAATTTGTATCTTCTACGTGTGGAATATTTACATTTTACTTGTCAGATTTT
TTTTCCAGTTCTCCAGGCTAATAAAGATATGGCAGATAGAGTCCTATCACTAGAGGAAGGTCCTTCTTTA
TTAAAAACAAAATGTATTAGAAAAAATTGATCTTAAACACATGCCAGAGATGTTCCCAGCAAGGGAATAAG
CAAGGTGTCAAAAGAATAGGCTACTTCAGAGCAGGTGTGAGTATGGAGGGAGAGTACTCACATTAGTAGC
TAATTAGGTAAAGACCATAATTAGTTTTGACCCAGGAAAGCAGAAGTAAGAAGCTCTGAGATGCCAGGGG
TGTTCACAGTCCATTTCAGAAACAGAAAACAGTGCACTCATGACGTCAATGATTCGGAGCTACTTGTGGC
TGTTAGTTGGAATAACAAATGTTTAGTTTCCCAACACGGTGTCAGATAAATCCAATTAATTGTAAAAGTA
TGAAGCAGAATCAGAGGCCAGACAGTAGTCAAGCAACAAGTTCTGCTTATTTAAAATTAAGCAGCCACCG
GTGACTTTCTCAAGCTGTTAGTAAAAAAAAAAAAAAAAGCAGAATACAATTTGCATATTTCTTAGTTTGCA
GATCTTTTAAAATAACATTATGAGATAGCACCAAGAAAAGAGGGTACCTTTGCTTTTTCTCCACATCCTG
AGTTTTCATCTGCCTGGAGTCCTCGATTAAGCCTAACCTGTATTCACTTCTTGCAGAAAGTAACTGTCCT
AGCAAAGGTCACTCCTAATCAGTTGATCTGTAACAAGCTGGACCTGATCTTCTAATTTGAATGCCCCACC
CCACAAGCGCCCACACACACCTTGCATTATTTTTGGCTCCATATAACTTAGTATTTTATATTTTTATGTA
TCTTGTCTCTGTCAGGCTGGTCCCCACCCCCAACCCTCTGCCACTGTAAATTCAGTAAGGGATTTGTTTC
TGATTTGTTCTTTGCTATATCCCTGGGTACTAAAACTGTGCCTGGCCCACACTGGAAGTGTTGGTCTCTC
AGTTATGTCCGACTCTTTGTGACCCCATGGACTGTAGTAGGTCAGGTTCTTCTGTCCATAGAATTCTTCA
GGCAAGAATACTGGAGTGAGCTACCATTTACTCCTCCAAGGGGGTCTTCCCCACCCAGGGATCAAACCTA
GGTCTCCTGCATTGTAGGCAGATTCTTTCCCACCTGAGCCACCAGGCACTTTGCTGCTGCTGCTGCTAAG
TTGCTTTAGTCATGTCTGACTCTGTGCGACCCCAGACACGGCAGCCCACCAGGCTCCCCCGTCCCTGGGA
TTCTCTAGGCAAGAACACCGGAGCGGGTTGACATTTCCTTCTCCAATGCATGAAAGTGAAAAGTGAAAGT
GAAGTCTCTCAGTCGTGTCCAACTCTTCGCGACCCCATGGACTGTAGCCTACCAGGCTCCTCCGTCCATG
GGATTTCCCAGGCAAGACTACTGGAGAGGGGTGCCATTACCCACCTGAGCCACCAGGCACTTTAGGTTCT
CAATATGCTGCTAAGTCACTTCAGTCGTGTCCGACTCTGTGTGACCCCATAGACGGCGGCCCACCAGGCT
CCCCCGTCTCTGGGATTCTCCGGGCAAGAACACTGGAGTGGGTTGCCATTTCCTTCTCCAATGCATGAAA
GGGAAAAGTGAAAGTGAAGTCGCTCAGTCGTGTCCGACTCTTCGCGACCCCATGGTCTGCAGCCTACCAG
GCTCCTCTGTCCATGGGATTTTCCAGGCAAGAGTACTGGAGTGGGGTGCCATTGCCTTCTCCAAGGTTCT
CAATATAGTTGAATGAAATGAATGAATGTAATATTCACTAATGACCAACAGAGGGCGACAGAAATTCAGT
AGGGGAAGAAATGAACATGTTTTATTCCGAGCTTCGCGTACAATTACGCGAGATTTGAACTCGCTCGGCC
ACCGTAGTGCGTAGGCCGAAAAAGAGCAGCCACTTCCGGCCTCTGCGGTCCGGGAGGATTGCTTTGGACT
ATCGGAAACTGAACTCCTAGGTTCTCAGGCAGCTCCATTCCGGAGCAGAGGCTCCATCCTGGAGCTCTAG
GCCTCCGATCCGCCCGGCCGCCGACGTGGCAGCTGGAGACACTTTCAGGGCCAGGAATAGGGAGCGTGTG
TCGCCATGGCGACACCGGCGGGGCTGGAGCGCTGGGTGCAAGACGAGCTGCACTCGGTGCTGGGGCTGAG
CGAGCGGCACGTGGCCCAGTTCCTGATCGGTACCGCGCAGCGCTGTGCCTCGGCAGAAGAGTTCGTGCAG
CGCTTGCGAGA

>PPP1R10|hg19
CTAAGGTAATCTCGTTCAGTACTGCAAAGAGCCTTTAAGTTTTTATACCGAAGACAAGATTCTCCTTACC
CTAAAGTCAACATGCAGCTTCTGATCCCGGCAGATGGGGCAGGGGTTCCCAGCAACTTTATTTCCACGCT
GTAGAGAAAAGAAACAGGCAGGTGAAGACGTTCAAAGCCTCTGTCACTATGGCCTTCAGCCCCACTCCAC
ATCCCATCCATGTGCCATCCTCAGAAACTCACAATACACATCTTTCGAGTCCGCTGTGGGGGTATTCCAC
CTTTGTGGTTTCGACGGTAGTCAGCCCAGACAGGGCGAGAACCATAGCGGTTGTGGTATTCTGAAATGAA
AAGACCATACAAGCTATTCCTGGGTGATGATGGTGAGGTGTCTCTGTCCACTTATTTCAGACCTCACTTG
ACACGCCCCTTGTCCATTTAGAAGTACCGTCTGGGGACTTCCCTGGTGGTCTAGGCAAGTGGCTAAAATC
GTGTGCTCCCAATGCAGGGGGCCTGGGTTGGATTCCTGGTCAGGGAACTAGATCCCACATGCCGCAGCCA
CAACCTGGTACAGCCAAATAAATAAGTAATAAATATTAAAAAAAATAAACAAGTACCTTCTGAGTCCAGG
TATTTCCAGGGTTCATCCTCATAAGGGGAAACAGGAATTGGGGGCAAAGAATCATCCTCAGGGGGGGCTT
TGGTGCAAAGAGTCTGGAGGGGAACCTATAAAAGAAGGGGCAGAGAGAAAATGAGACAAGAGTTCAAGGA
CACAACATGATTGGTTTATTAAACTTACACCATCAGCAACAGACGGGAAGCTATTAAAGGAGGGGCAGAG
CAGAAATGGTTGGATGGCATCACCAACTCAATGGACATGAGTTTGAGCAAGCTCCAAGAGACGGTGAAGG
AGAGGGAAGCCTGGTGTGCTGCAGTTGATGATATCGCAGAGTCGGACACAACTGAACGACTGAACAACAG

AAGGAAAACGAGACACTGAGTTCAAGGACAAGAACTGACTGGTTTATTAAGTCTACACCATCAACAACCA
GTATCCCTGCCTCCAGCTGGATCTTGCAAGAAAAGGTAACACTGGAAGATAAATAGGTGACTGGGAAGCA
AATTTATTTGAAAATACAACTCAAAAACCAGGCACACATAAGGTTTCTGTGTTTCCTGAAATGAACTCAG
TGTAATGGAGAAAAAAGGGAGAATTTCTATCTTCTGGGAGCTAAGATGTGTACTACGAAAAGGATGTAAA
GGACAGGTGAGTCTCCCAACTCAACTGAATCCTGAATCACTGAGTTGCCCGGTCTTATAAAAAGTTGATG
GTCTACATTCCATATGGCAGAACAAACTTGAGTCCTAACGCTATGTCCGAAGCTACCAAGTCTATATTTA
ACGCTGTGGTAGCCCCTCTCATCACTCTTCAGTCTCACTTTTGGGAGTGGCAGATGGGCAAGCCTTCCAA
AATCACGCTGCCCCATCAAGAATCTGCCCTACCAAGAATCTCCAGGGTGGTGAGTACGTGTGGCTGAGGG
AAAACCTCAATCCAAAGTAGTTAATGCTCTCATGTTGGTAAGAAACTGATTTAGTGAGATAAAGAGCCGA
GAAACTGGTCCCCCGGGCGGGAGAATGTATTCTGGCTTTACTGAGCGATAGCCGGGCTGGTAGTAGTTAG
TGTCCCTCACAACCCAATGACACCCTCGGGCATCAAAGATTTAGACACACATACAGGTAACAGCTGGTTT
GTGTTTGGCCAAGGGGTCCAATCCATTCCATCGTCTAATTATTCAGCCGAGACGGAAACTTGGGAAGAGA
ATGACATCCAACCCTGGCTTTTTACACTTGTCTCGAGATTAGAAAACGCGCTGTACTGAGGAATGAGAGG
GGAAGGGGGAGGATGGTGGAAGCAGAGCGTTTCCTTGACAGCCAATGTATAAGCCCTTGGGAGGGTATCC
GTTCCTGGATTCCCGGCAGTGTAGCGACGACCTAGAGAAAGTACAACCAAATCTGAGGTTGTACAAAATG
TGCCCCAAGGAGTTACCTGAACTCCGTAGGCACCTCTGAAGGGCGAAAAGTAAGGAAGGCGCCTCAGCAA
CACGTTCAATACGGAGGCAGCCATCTTGACGTACCCGCAAAGCAGGAAAGCCCGCAAACGAGACTGCGCA
TGTTCCAGGAACCAGAGTAGGGTGGGGCCAAGGACGACTCCGCAGTACGAAAGGCGGACGCGCGTCTGCG
TCAGGACGGAAGCGTGAGGGGCGGGCGTAGGGAGAGGGCAATTTCAGCTGCGTCACAATCGAATTCGGCC
TTAAAATTGCGTTTTTGCTGGACCAGTCACTATGACTCTTAACTCTCATTCTTAGAGTCTGTGTTTTAAG
CAATTTTGAAGTCGCTTTTGACCGTAATCCTTTCATTGCGCATGCGCCGGTCCGATTCTACCAACCCTTG
GAGAGAGATGTTCTGGAGGGCGGAAGTGGTGGAAGCAGAGGAAAGGGCGGTACTAGGCTGCTTGGTCACG
CGCACGCGTTAGGGGGGGCACCCCCCTCCAAGGAAGGAGGCGGAAGAGGGTGCCCGGGGGAAGGGGCTGA
GCGAAGGTTAAGGCTGGATATCCAATCAGCTGGTGAGTGGTTTATCGGGTTGACCAATGAATTCACGCCT
CGCGCATACTGTCTCGCGAAGAGGGCGTTTCACTAGCACGTTTGGGCGCATTGGGCGGTGTCCTCGTATA
AAAGACTCCGCCCGAGCGGGCAGCCGCCATTCTGGGGTTCGTTTAGAGGTAAGTGTTGCCATTTTGTCGG
TTGGTGGGTAACATTTTGCTCGGAGAGTTGGCCTGGGGAACGGGTCAAACGCGGGCTCAGTTGAGAGCCA
TGACTCGTATTAAGCGGCTGTGTTCGGGGTGGGACATGGAAGTGTTTGGGGACAACAAAAATGGTACTTT
TGGGTGTTGTAATAGCAGCCCGTACTGATTATCTTTCTCCCTTGTGTTCCTTTTATCCCAGGTTTGAATT
TTCTCGGAGAAAGACAGGCCGGCCACCAGGAGAAAAGAAACAAGCCGCAGCAACATCTAAGCCCTTGAAA
GGATCCAGACAGAGGGGGGAAAGGGAAAAGAGCAGCCACCAGCCCAACCACTTGTGTCTTCTGCCCCTTC
CCACCTATCTTGCCCACCCCACCAGCCCACGCTGCCTGGGACTTGAAATCTGTGGCCGAAGGACCGTCAC
CACATAACTTCAAAAATAATCAACCACCCACCCTTCCCAAACTCACCCAAGTCCACTCATCCAGCGTTTA
CTTTTTTTAAT

>GNL1|hg19
GGACTTTCTTCTTTTGAGGGGCAGAATCATATTCCACTACATGATTATACCACATTTTGTTTATCCATTC
ATCCATCAATGGGCATCTGGTGGCTTCCATATCCTGTCTACTGTGCACAGTGCTGCTATGAACATGGGTG
TGTGACTCCCTCTTCAAGATCGTGCTTTGCATTGTTCTGGGTGAACACCCAGAAGTGGAATTGCTGGATC
ACATAGTAGTTCTATTTTAATTTTTGACGAATCTCCATACTGTTTTCCACTGTGGTTTCACCACTTTACA
ATCCCATCAACAGTACACAAAGGGTTTGGTTTCCAAGTTATATGCTTTTATCTTGCCCTTATTTGATGAC
TAATGATGCTGTGTTCTTGTTCATGTTTTTGGCCATTTAGATTTCAGTTTTTATTAGGTGGCTGTTCTAG
TCTTTTGCCCACTTTTCTAACAGATTGTCAGAGAGTTTTTTTATATATTCAAGATTTTAACTCTTTATAA
ATTCAAGATTTTATTGGTTGTATTTGTTTCAAGTATCTTCTACTCTATGGCTTCCTATTAACTTCTTAAT
GGTGTGATTTGATTAATAGAAGTTCATATTTTCTAATGTAATTCACTTTATTTTTCCTTTATGCTTAGTG
TGTTGCTTGTCTGTTTAAAAACTCTAATCTACTTTAAGACTATAAAATACATTTGTTTTCTAAAATTACT
TCTTTACCCATGAATATCCAATTAACCTACCATGATTTGCTAAAAAGGCCATTATTTCAATTCCGTCTTC
AAGAATGAAATAACCATATGTGTGTGGGCCTGTTATTGGACTCTTTATGCCATTCTGTTAATTTATATGT
CTATTTTTGGGCCAATACCACACTGTCATAACTGCTACAGCTTTAAAATAAGTCTTGATATTCAGAGTAA
GCCCTTCCCCTTAAAAAATTGTCTTTGCAATTTCTCACCCTGATCCTTTGCATTTCAATACAATAATGTA
GAATGCTTATTTCTACAAAAACATTGTAGGGATTATAAGTGGGAATGTAATGGATCTATGGGTTAATTTT
GGAAGAACTGTCATTTTATAACATTGAGTTTTTCATTTAAGTACAGAATTCTTGATCTTTTTTTTTTTAA
TCCCTTGTGGGTTTTAGTCCCTCCCTTGCTCCCTTTTCAGGACCCAATCAAAGGATCAGAGCCCCTTTCT

TCTCACCTGACTTGGGGTCACCAGGCTTTCCATTGGCCATGGGGGGAGGGCCCAGAAGGCTGGGTGGTCC
TAAGAGTAGACATGAAAATATGGTCATCAGAATAGACTAAAAAACTGATGATCCTGTTCTTATATGCTTT
CCTTCTATTACTCATTTCTTAACCAGTAAAGCCAGATATATTTTCAACAGTGTTATGGGTTCTGACGCCC
ATCTATTTCCAAAGCATTTTCAGGATTTGCAACGTGGAGACTTAGCCCTGTCACGGCGTATAATGAGAAA
ACGATACCTACAAACTTTCTCTCTCCCTTACATTGTTGCTATCCAATAGATCCCAAGTGATTCCAGAGGT
AAAACCAATCCCTCTCTTTAACTGGCCAATCAGATACTTCTCCGCTAACCCTACCCGCAAGGAGTTCACA
GAAAGCTCTACTTCCACCTTCCCCATGCAAGTGTGGTCACAAACTGTCATCCCAGTCATCGCAATCTGCA
ACTTCTCTAGGGCCCCTCTCCCCCTGACGGTTTGCGGCTGCGCACATAAGTTCTTTATCTCCTCTCCCTT
CTTTAGAAAGGTTTAGAGGCCAGTTTGAAGTTGAAGATTCAAGTAAAAGAACTCATTTAGTGTTCCATCC
CACACAGGCTTCTCCCCAAGCCCCCGCCCCAGCGCGTGCGCCCTTCCTCCCGTTAGAGCCCTCTCCCCCG
CATCTTTTCAGCTTCTAACCCTTCGCCTCGACAGGCTGACAGGCATGTGCGTACCCCTCCAGGGCTCCTC
ACCGATGGGGCTCCCATCCTCCTCGTCTCCAGTCTCATCCCGCTCTGGCAGTGGGGGCTGCTGCTGCAGC
TGCTGCTGCTGATTCTGCTTCTTTCGTTTCGGCATCGTGGTTGCGGCAATGGCTGCAGCGGGATTTGGAG
GGCAGTGAGGAAGGGGCACGCTGGGATTCTGTTTCCGCTTCCGGGGGGCGCGGTGGACACTGAGGCTGAG
GCTCCCGCCATTTCACTTCCGGAAGGCTGCGGGGAATCTTTGTGCGGCTCTTCTCGCATCAGCCGCCTCC
CGCAGTCTCTGCCAGGCAGGGCAGCAATCTCAAACCTTTCCGTTGCTGCGTTGCTATAAACGCTTTGAAA
AAGTTGCTAGCTCCTCCCCCTCGCTGAAAAAAAAATAAACAACCGGCTGCTCCTCACAGTCTGCCATAGA
GTCGACAGACTCTCAGGCCAGAAGGCCTGAGAAGAGAAGGCTGTCGCATCCTGAGATCGCCATATCCGGT
CCCCGGTTTCTATGGTAACAGCTGCAGGCACAACTCGGTGCCCTATGACTTCCCTCCAAAAATCCTTGCC
CCATCTGGGTTTCTCTCCTCGGCGGCGCTTGCATTTATGATCTGTAAGCCTGCTCCTTATCATCGTTCCC
ACAAGGAATCTGGGTCCCAGCAGGGCGCTGCCCAGGGCTCTCCAGACCGCCACCCGCCCTTCAAGACCTC
TGACCCGGCCCCCAGTGTAGTTCAGTAACTCAGCTTCCTTCCCGGGACGCGCAGTCGCGCCTTTCCTCTA
AGGCCCAGCTGGAGGATCGGTAAAGAAGCTTATGCGTGTCCACGCCCCTTTCTCCCCAGGTGGTAGGACC
CGCCCTCCGCCCACCGCCCCCTCCAGGTGAGTGGTAGCTTCTCCCCGGCGCGCAAGGTAATGATGACACG
CGTCCCCCCTCCTCTGAAGGCGAGTTGGTAGCGTCCGTGACGAAGTTAGCCCGATCCTCCCACGCGCGCC
TCCTTCCTCGCCGCCGCAGCGCCCTCTCAGTGCCACTGGCTCTCACGTGCGGGTAGCCTACCCTGCATCA
TCCTCTCGCCTCGCTCTCACTGGGACGTGGCTGTATTTCCCCGTTGTCTTCCACCCGGGTCGCCCGGGCG
CAGCGGCCGTGCCCGCCCGCTGACGTCACCTTCCAGCCCCGCCCCCTCCAGGGTCCCGGGCCCTTGAGGC
GGGGGCTGCCCCGGGGGGAGTCAGTTGAGGCGGCGGGAGCTCGGCGGAGGGCGGGCCAGGTGACTGGTCC
GGGCCATGCCGAGGAAGAAGCCTTTCAGCGTGAAGCAGAAGAAAAAGCAGTTGCAGGACAAGCGGGAGCG
GAAGCGAGGTC

>ENSBTAG00000063412|hg19
GCTTGACGGCCTGCAGCTGCTTAGCAATTTCCACCATACTGCCCAGCTGTCGATTAGGCCGGAGACTGCG
GTAGCGGGACGTCTTTCGACATACAGGACAAGGGAAGTCCCTCTCTAGGTCCTCCCACCAGCGAGTGATG
CAAGCTTTGCAGAAGTTGTGCCCACACTCAATGATGACAGGCTCCTTGAGATACTCCAGGCACACAGAGC
AACTTGCCTCCACCTGTAGGTTCTCCAGAGCTGCAGCTGTGGAGGCTGCAGAGGCCCCGGCCTCTAAGAG
ACTTGTCTCTGCCATTATTTAACTTGAGGTTAAGAAGAGAGAAGAGAAAAAAAAAAAAAAGTTAGTTGGGA
GGGTTAATTTTTTTGCATCCCTGGCCTGACATTTACCCCCATGTTAAATCCTAACTCCAGATTTAGCACT
GTCTTGGTATTTTTACATCTTCTGTTCCACCAGTCAATCTCTTCGGTTTCTCCATTTCCAAAAATTGTGA
CTCCCAGCTATGAGCAAACTGACACACAACCCTCAGTCTTGTCTTTCTCCACTGCCTTCTCTTGTTAGAA
GCAGAGGCTTCTTTCAAGTCTAGCCCAGAGTATTCATCCTGATCCAGGTTCATTCCACTAGTATTGTAAC
AGTTTCAGAAAAATGTATTCTTAGTTTGACATCTTTTTCTAATAGATGAAAAAAGAGTATGGGAAAGTGA
AAAGTACCTTAAGAGTCAAAGCATTAAGATTGCTTAAATCAAGTATATTTTAAAGTTGAATGCTACAGAA
GAGTTCTACTACAATAGCCCACTGAAGCCTAAGCTTGATTCTCTGCCACTGTTCCCTCAGTCTTTCAAAT
GGTAGATATTGGAATGAGTTAGGCAAGGGTAACAACCTCTTCTCTTCAGAAAAGTGTGACACTATGCTTC
CCTTCACCAGGCCTAGAGAAGTGTGCACTTATCATGGAATGAAACGATTCACACAACTGTTTCCTATTTG
TGTGCATTTGGATGTTTGGAGCTGCTGTAAAAGTGATAAATTTACAGCAGCTATACCAAAGAGTTTATTA
CTGAGCCCAGGGCAATAATTTCTTGAAAAGAAGAGTTGACAAATTTATTCTAAGTATTCAGTGAGGACTG
GAGTCTTTTGAAATAGTTGTTGATAACTAATTCCTTGGATATACTGTACTTTCAACTAATAGGTTCTGGG
GTGGTCGGGAATTAAGAGAGAAAAGTAAAATGGCAATCAAAAACTCTAATGAAGCCTCAAAAGAATTGGA
AATGCGCCTGAGAGATGTATAAAGCAGAAGATTGGTCCTCTTTAAGGTCCATCCTTAAAACATAACCACT
ATTATCCATGAAATATTTCATTTCTCTACCCCTTAACCTCTCTTTCTAAGGAACCCAACAGAAGAAAATT

TAATACGTCTATCTATGTGTTAGGAGAGAAGTGGCCATGTAGTGAGTTGGGTTAACAATGATTGTATCTG
TATATACTGATGGCAAACGTGAAAAGTGTTTAATTTGGAGTAGGAACTGATAACATATTTGGACCTGAAT
TTCTCCCTCAACCACGAGAGAAGTTCAGTTAGATGATTTTTCTCTACCTACAGGAATCCAACTTCGACGA
GTAGCCCTTCCCTTTTCCTAATCTAGAAATTAGATGGTCGCCTCACCCGAGATGAGTTGAATTGTATGCT
GAGTTAGGGACCAGGATGGATCTAGACTGGCGAAAAAGTAGCGTTAGAGAGAGATGGGGGAAAAGGTTAG
GGAAGTATGGGGAGAGGGCTTGGAGCTACAGCATTAGTGAGGCCTGGGTCATTTCCCCCCTATTCCTCGT
GTCTCAGTAGAAGGACAAACTTCAGAGAGGTCCTCATTGTGTTTCTACCACTGTCGGGGGTCCCACCTTG
CGCCCCTTTCCTGAGCGTGGTCCACAACTCGGTCCCACCCCCGTCTCCATCTTCTCCCGCCTGATCAGGC
GCAGGGGGGCTGGAGAGGGGGGAGAGGAAACGTCGTTGTTGGCAGGGAGGGAGGGATGCGAGTCCAGGTG
AGAAGGTGTGACGTGAAGATGGGATGCCCGCCGCTATGACGCAGCCGAGACCCGCGGTGACGGCGTCAGT
TTCACCCGGGGAGGCGAGCGGCAGCGGGACGGGGTAACGGCGGGGTAGCCAAGCAACGCCGGACGCGCTG
ACGTCGCCGGGGCGGCAAAACGTCCGCCCGGGCCTGAAACGGGCGGAGGCAGCTGTGGTTACGTGCGGGG
GGCCGGTGACGCAGTCGCGGGGCGCTAGGACGCGGCGTGCGGAGCCGGCGGGCAGGGGCAGGCCCCAGGG
TCCCGGCGCGGCGGCGGGACAGCACCTACCTTCCCGGCTTCGGGTCCGGGAGTGCGGGGCGCGGAGCCGC
CGGAGCGCTGCCTCCCTCCTTCCTCCTCCCCCCGCCCCCGCCCCGCAGCGCGACACACAATACTCGCCGG
AAGCGGAAGCCTCGCCGAGAGGCTCGTGTCAGTGGAAGCTGGGGTGTAGGCGGGCGACAGCGGGAGGGTC
TAGCGGCGCCTGCCGAACAAGAAGGCGGGGGGCCGGAGGTGAGCACCAGCTGGAGGAGTCAGGGGCAAGA
AGAGACCCGGAGGGGCAGGTGGCTGGAATGCGGTCGTCCAGGTGACCCGAGGAATCCGCTGGAGAAAGAC
AGCCGCGCATGCGCGTGCCGGAGCGCTGAAGCCCATTGCCCTCAGGGCGCCTACGGGCGCCGGGGCAAGC
GGTACTGTCCCGCCCAGGGTCCGCGCCCGGACTCTGGCCTGGCTCGGTTGTTCCCAGGTGGCACTGGGAG
CCGTCGAGGGCGGAGCAGGGGCGGGTTGCGGACTTTGAGCTACACTTCTCCTCTCTCCTGGCCAGTCTCG
GGAGAAGGTGGCTCGCCGAGTCGGCGGCCAATAATGTGGCGGTCCTGCTGGTCTCAGCTGGCACGTCATT
TCCGACTAGTTGAGTTTTAGGCTTTTAACATTCAAGGATTAGCTCAGTCCTGCTTTGGGCTTCGGATATT
CCTTACCTTGTCCCTTCTAGGCGGGCTTTATGGAAATTACCTGCTGTCTCCGCTGGAGTGGGAGCCTCGA
AAGAGCTGGTACTGCGACTCTGCTGCTCACCTTCGCATCCTCGGCTCCTTGCTCGGGGCCCGACTGCAGT
GAGCGCGCCTTGAACTCGGGTTCTGAATGCGAGAGAATCCTGTCTTTTTCCAGTTTGGGAGCTTTGACTT
GAGTTTCTCCCCAAGAGTAGATAGTGCTTGTATGGAGACTCCAGACTCCTGCGTTATTACCTCCTTACTT
TCCTCTCCTGT

>ENSBTAG00000038619|hg19
AGTAGTGGAAAGCTTAGCTAAATTATCTCCTGGAGAATTTGTAAGAAATGAAACTGTTTATATAGCTAAG
GAGATTTCTGAGCACAGTTGGTGCTGCTTGTTCCTGTTTATGTTACAGGAACATGAGAGGAAAGAGATAA
ACAGGAACCAGGGTTGGCTTATTTTAAAAATTCTCTCTAGATGACAAAAAGCCAGGAGGAGATAGAAAGG
CGATGTACTTTGTTTTTTTCATCTTTCTTTTGGCTCCACGTCCTTGCCTCAACCCTAATGTGGAGGTTTG
AACCTTCTCCCCTTAAACTGCCAAGCCATGGAAAAGAGCTTACTCTGAGGCAACTCTGACTCAGTGGTGG
TCTCATTTCCTCCAGATCAGGGGAAGGAACTGCCCCTTCCAAATTCCTCTTAGTAGTTTCTGGCTTAAAC
AGTAATCCTGCTGGATTTATTTTAATTGAACATTTTATTTTCCCCCCAGTGTTTCAGATAAAATTAACCT
ATAACATATTCATTAAAGGTGGAGGAATGATGATTTGATATATGTTTGTACTGATAACAGGACTGCCAGT
GTGACTAATGGGACATTTTATTTTTTTATTTTTTTTTAAGTACCAAGGATCTTTATTCTTAGGTCTGATA
TTTAATCTGTGGCTGAAGATAATATGTCACTATTTAGAGAATAAAAGGTCCCATTATCTTCATTCAAGTC
TTCAGCCACAAAGTTACACCTTATTCTCATACAAGATAAGGGTTGGTAGGGGATTTAGGAGGTACACTGA
GGATAAAAGCAAAAGGGAACAAACACTTTTACAAGCATATTGTCCCAGTTACCGTGAAGGTTACGTTTCA
GGTTAATTTACAAAAGTAGTGACAGAACCATGATTTCAGTGAAAATCAACACATTATACCTTTTAAAAAA
CCAGAAATACTAAGTCAGGCAAGCATGCGAAATTCAGAGTTAAAAAAACGCAAGGGCTGGCTGCCCAAAC
AGGTATTTCCCTCAGAAAGAGGGATTTGAAAATGCTGCACAGAACCAAAGGAATTAGAAGCTGAATTAGC
ACACTCATTTCCTCAGTTTATATATGAGGCAAGTGGGCAGACATTAACTGGTAAAGCTTACTCAGCCAGA
TTAAGAATGTATAGACATCCAATTCCTGGTGCTATCCGCAACTACATGTATCTAAAATACAAGGAGGTAA
ATATCCAGAATACATCAAACCCATTGATTTAAAGAAAACATTTCAAGGGAGGGGGTGAAGTTGTTATTAT
AGCATAGCAGCACATTACACATATTTAAGAGATTAAATGGAAAAAAATAATCTCTTAATGCTCAGTTTTG
ATCAACACAAGTTTCCAGCCAACTTTCTGATGTAGAGCAAAACAAGTATATTCTTCAGTTTTCTTCTACA
TTTTTTGGGGCCTGTCTTTCAAAACCGAGCACCAGGTATTTTTAATGTAAGTAATGGGATGTTATATGTA
CATTCTAATCACACATTTTAAGAATAAACAAAATGCATATTTCAGTAATTCGTAGTGTATTTATACAATG
AAAAGCTCTCTATCAAAATACACTTCACTGGGAAAAATAAATAAAACAGACAAATGGATCTACACAAAGT

AAAAATTAACTTTGGTAGACTTCACTGTGGAGGACAGTCCATAACAAGCTTATCTGCCCTTACTCCCCCA
GAGCCGATCATCTTCTGAGTTAAGATTTTAAAAATATGTTTTAATTGAGATCATTATGTTGACATTTGTT
TCTCATTCTACATCATCTTCAGCCAAGCTCTGAGCACTTACAATTCTCTGACTAATTGTTGGGAGATAAG
TCAGAAGCATCTGGAACACTGGTGGTGGAAGTTTGTTGCAACACTTGCAGTAACTGCTGTGGCTGTCCAC
ACAGCAATCGCATTTGTCAGATGGTCCACACCCTTCTCATATTCACCTTGAGCTAGTAATTCTTCACCAA
GCTGTATTTCTTCTACGAAGAATTTCTGAACGGCTTCAGCATCTTTAAGGTCAGGTAACTTGGAAAGCCC
AGCTCTCTCCTTGGCAAGCTTCTGTTTCTTTCTTCGTTCTCGCAGCCTGTTCTTGAAGTTGGGGTCACTT
CGTCTCTTGCGGTCGAAGTAAATGCAGTAACCGATGAAAAGGGCCCCGCACACGCCGGCGGCGATGGCGC
TGTTCCGGCCCACCATCTTCTCTCCACCGAGGAGCCGGGTCGGCTGCGGCGGGGCCCTCAAAGAAGCCGT
TGACCGCGAAGCCTCGGATCCGAGTGCGCGGCTCAGTCCCGACGCCCGACGCCCGCCGCGAGGGACCTAA
TGGGACATTTTAAATGAGTGGCAGTTCACACCATAAATCGCCCCAAGTGAGAACCTTATTTTTGTGTCTT
TATTGTCGATTTCCTCCCTAAACACGTAATTGTTTTTTTTCTTGCCCCGCGGTCCTCAGCAGGGAAAGCG
CCTAGTCCAAACCACTGGACCGCCAGGGAATTCCTTAATGCTCCTTGTTTTTGTTTTTTGTTTTGACATG
GCTATCAATTCAGTTTTAATTATTTGTTTATCACGTTCCCCCACTTAACATATATATGGCTACATTTTTC
CAAGCTGCTGCTGCTGCTGCTAAGTCGCTTCAGTCCTGCTAGTCACTCTTAACTTCTCCGTAGTTTTCCA
AATACAGATTAACCTTATTTGCATTTGGATGAGGAAGAAACCAAGGCTCCGAAGCGGAGAGGCCCGGCTA
GGATCTGAAGTCCAAGCTGGTGGGTCCCATCTAGACCTGTCCTGGGCTACTGGCTGACTTGCTGTGGCAT
TGGCTATGCGGGATGCCATATGCTGGGGCTTTCGTTTTCTCCAAATTCAGCTTCAGTTCCGCAGATGTGT
TCCTGACCAGCGTATCGCTGAGTGACTGGTGCTCTGTAATAGTTTCCGCCCCCCACTGCGACTATTTCTT
GCGCTTTCAAGCGGTTTCAGTTCCCGTAAGGCGCCACCCCTAATGCGGTCCAGAGCTCGAGTGAAGCCGG
TGGTCTCCGAGCTGTGTGCCACAGCCGTGCCCGGAAGTCTTGGGGCCAATCGGAACAGCCAAGGCGATGC
TGTGGGGCCCTGTCCTCTTGTATTTCTTCCTAGCATACCTGCCAGAGACACAAACCCGTGAGTTGGGAGC
GAGGGGTGGCGGGCTGGGAGAGTGGAAGAAGCTGAAACCGGACGAGAGTGGAATTTTTCAGATCACTGGA
AGCTTAGTGGTGACTTTGAAAATCTTGGTGCAGGCCGTCACTGGCTTATTTGTGCGGGCTCTGTCAATAA
GGGCAGCTAGT

>TRIM26|hg19
CGAACCCGCATCTCTTACATCTCCTGTATCGGCAAAGTTCTTGGCTGGTTCTTTACCACTAGCAACACCT
GGAAAGCCCCTGTGCAGTCCTAAATCCTGTAGCATGCATAGCCTAAAAATACGGACATTCTCATACATAA
CTTTAATAAATGCCATTAAAAATCAATAATAATTTCTCAATATGATCCGAAACCCAGTCTTTAGTCAAGT
TTCTCCTAATTGTCCCCAAAGTGTTTTATAACTTTTTAAAAAAGCCAAGATCCAATCAAAGTTTTTGCAT
TTGGTTGTTATTTCCCTTTAGTCTATTTATATCAAGAACAATCCTGCTTTATTTTTCATAGCTTGACTTT
TTAGAAGACCATGCCAGTTGTCTTGGGGAATGTCTCATGTTCTCGATTTATCTGATTATTTCCTGATGAT
GTGGTTTATAGCTCATCTCTGTCCTCTGTATTTTTGGTAAACTGCAAGTTAGGTCGAAAGATTTGATTCA
TTCTCGTCAAACACTTTTGGCAGGGCTACTGCAAAGGTGACATTTTACAATTCCTGTGGCTCCACACTGA
GAAACATATGCCAGTCTGTCCCTTTATTGGCGATGCTGGATTTGGTCACTGGCTGAAGCCATCACCAGAT
CTCCCTTATCAAGGTGCATTAGGGAGTAACCCACTGGGCGGTACCTTGGCACCATGGTACCATCCTGTCC
CCTGGTACCCTTCATCTCATGGTTTTAGCATCAGATGATGATCCTTGTCTTATATAGTGGGGGTTGCAAG
ATGATGTTTTTCTAATTCTATTATCCCTCCTACACTTTTTCACTGGTGTTCTTCCATAATCCCTGGACAG
GTTTGTTCGTCTAGTACATATGCGATCATTCATATAATGTTGGGGGTTTAAAGATGCATCAAAGTCCAGC
ATGGAGCCTGGGTTAAGAATCCCTGCTCCAAGTGATGAGGGTATCAGGTTTTGTGTGGAGTGGGAGCCAT
AGCTAAGCTCTTCATAGTCATTTGCAACAGAATTAATTTCCTAGCCCTAAAGATAAACACATGCCATTCC
CCATTACCACAAAAGAAACAAATAACCTCAACCTTTGAACTTCCAAGCCCCTAGCAAAGTGATTGCTGAA
GGGCTCCAGTGCCCACAGGAATCACCAACACTGCCGCCCACCGGTCCCAACCTTGTGTTGGGACGACAGA
CGATGAGATGGCTGGATGGCATCACCAACTTGATGGATGTGAGTTTGAGCAAGCCCTAGGAGTTGGTGAT
GGACAGGGAAGCCTGGTGTGCTGCAGCCCATGAGGTCGCAGAGAGTCAGACATGACTGAGCAACTGAACT
GAACCTTGTGTTACATTTCCAAGTGCCGGGTTAGGCTCACTTCCTCAACAGACAAACCAGGGAGTTGCAC
CAGGCAATTCCTAGACTTTATACCATCCTTATTAACATTCTAAAAACTTTATTACCTGCATTTACCAGTT
AAATTCTTATTTCATTTGAGCTTTGTAGGTACAGGTCTTTTCCACACTCGTTACATCTCTTAAAGTCACA
CATTTCTAAATCTGTTGGAGCTTCTAGTTGGAGTTCATTGTCAGTAGTAAAATAATCCCTTACATTTGTA
CAGTACTTTCACTTTATAAAGCACTTTTTATATACTGCTGTGTTAGATCCTCAAGTAGACTTTGTATTCC
TGTGTTGTTTTCTCTTTGGGGCTTTATTTCTAGCTCATTTGTGTCAGTACGTGAAAGAAATTGAAGGCCA
CTGAACTCTGTTGTAATGAACTTTCATGTACATAATAAGTAATCAGATTGATTTGCCTGTGGTTCCAAGA

GCTTCATTCCAGCCTGGCAGTTACCTGTGTGCCCTGTCTGCTGGCTGTCTTCCCAGAGACTGCTCCGGTG
GCCTACCATGGGTCTCTCCACGAGACTTTAACTCTGGAGCCCATGTGTCACCGTGGCGTTTCATCCACTT
CCTCTCTTGCTGCCACTTTAAATTTCCACTCTTGCTTTTCTTGTGCTTTGAATACGCACTGGATTTGGGA
GCCAAGGGTTCTGGATTCTCTTCTAGATTCCCCCTCAATACCACGCTTATGCCTAATACTTCTAGCATAA
TTCTTTTACTCAATCCATGTAGAAACCCCTTGAAGGTAAGTATGACTCTCCCCATTTTATGGATGGATAA
ACTGAGGCTCAGAGTACTTAAATGAGTTTTCTCTTGTCACACCGTGTAAGGGCAGGAGGCAGCTGTTGGT
TTAGGTGTGCTGAGGCTCCTAACCTCAGACAGGAGGTGCACCAAGGCTGCAGAGGTAAACAGAACTAAAG
TCCTCTGTGCTCAACTTCTGTGCTCCCTCTGAGGGCCACGCTCTCATCCGCACATCCCTGTCAGAGCCAC
CTGAAGGTTCCTCCCACGTTGGTTTCAGTCACGCTTGTTAGCAGTAGGGCGACCAGGCCAGCTGCATGGA
GGGGCACAGCTGAGACCAAGTCCATTGTGGCCTGGCAGTGCTCTGCCAGCAGACTGCGTAGGTAGGGACC
AACTCTCTAGGGCAAGTGAACCCAGTCAAAAACTCACACAGCCCAAGGAAACATCTATAAAGGAGGACAC
CTGAACTCAACCTCTTGATGCTCCTTGAGTCTTAGTAAAGAACCTCAGGGTACTTAGTCTTTGCAACCTG
AAGTCTGCAGGGCAAACAAAGGAGTCTCTTGACTCTGTCAAGGCCCCTCATGAACAGCTGTGCAGCCCAT
GCACTGCACAACTCAGGGAGCATGGTGCACATAGTAGGTGTTGTGGATTTGTATATTATGACAGTTCAGC
AGGTAGTTATCTGGTGTCTTGTTCTCATAATCGGTGCATTAGAGCAATTTTCCAGCAGATGGAAGGAAAG
GGTCTGGAGATAGGAGTGTGTTCCCAAGAGTACGGCATGGGCTGGTACCTGCACTGCTCTCCCTTCTTCC
CAGCTACACACTCTGGGCCTCAAGTTTCCTCATCTGTAAAATGAGGGCGTTGGACTTGAGGATCAGTAAG
GTCCTTTCCCTCTGATTCTGGGGGAGCCTCATAGGAGCTGCAGTTGACTGGTCGATTGCTTGGGCACGCA
GCCCCATGTGAGGCTGCCACTCCTGCCCCTCTGCTGGGCCTGCTGTTGACTCCGTGTCTGGTCCCCAAGG
AGGAGAAGTCCCAGCCGGTGAACAGGGGCCCCTCTGAGTTCGGGATACCATGGCCGCATCGGCACCGCTG
CGGAGTCTGGAAGAGGAGGTGACCTGCTCCATCTGCCTCGATTACCTGCGGGACCCAGTGACCATTGACT
GTGGCCACGTC

>TRIM10|hg19
GGTCACTGCATCCTCCAGGGGCCCTTTGCACTTGGAACAGGTAGCCCCTTTGTGGGAGGGGGTCGAGGGC
ATCACGGTGTCTTCCCCAGCCCGTCCTCTCAGGCCTCTGCCTCAGTCCACATGAACCCTCTTTCCCTGAA
ATGGAATCACTTTACATGAGTCACAGAGGAAAGGAGTGGGAGGCTGAGAGAGGGGAAGGCAGGGGTGTCT
GGACAGAAAAGCCACCTGGCTCCACCTCTTGTGCTTCCCAGAGTCCCGGCTACCACTTATCAGCTGAGTG
GGTTCAAACTCCAGGGCATCTCGAATTACCCAGCATTCCCCAACAGCGGCCCTGGCAGCCCGTTCTCCCC
TCGTCCTAGTCCTTCCGGCCCCTGGGGTGGGGAACAGAGCACACCATGAGGTCACAGGGAGATGGGGTTT
CCTGGAATCCTTCCCTTGACCACTGGCGGGTGGGGAGAGTTGGGGAGGAGACCTTGCTTCCTGTCCGGCT
GAGCAGGTGGGAGCCATAAACTTCAGGCCCTGAGGTATGCACAGGTGTGCACACAGCGGCAAAGTCCAGC
TGGAGTGGGAGTGCTCACTTCCGGGAGATTGTTCTGGGGGCTTTGCTCAATGCTGCAGGTGTGATGATCG
CCCACCAGGACCCCTCCTCTGCACTTCCATTTTAGCTTTCAACTGCAGCAAGGAGAAGGCAAAAAAGCAT
AATACAAGTGATCTGTGTTGATACAATTTAGAAAAGTCAATAAAAAAAAAAGATAAACAGGTTCAACCAC
ATTTATCTAAGGCTTAGTATTTTCGTATTGTTACATCCAATCTGATAACAGCCCTTGAGAGCAGGTGATG
ATTATAGGAAATGTAAAGTGAAAGGTAAAGTGACCTTTCCTGAGTCACAGAGCTAGAAAGCTGCAAGGCT
GCAGTCTACTCAAGTCTCCTGGCTTCTAAGCTGCGTGCCCTTCCTGAACTCCCAGCCAGGACAAGGGGAT
CAGACAGTCATGGGTCTTCATCCGCTCACATGCATCTGTGTATAAAACCAAGGAAAACACCTCCCATAAC
CCCACTGGAGATCAGTCCTCCACCCTGTCTCACTCCCTTTCCACTCCAGCTACTTTGTGTATCGACTTCC
CTTCCCCCCACCTACACCCTCTGCCCCCAAGAGTGCAGTCCCTGGAGCTGCAGTGTCCAGCACAAGAGCC
TCTAAATGCCCGAGGCTGCTGGGCATTGAATGTGGCCGGTCCCAACTGAAAAATGCCGTATGTGTAAAAT
ACGCCCTGATTCCAGACTTTCTTGTTGTTTACTTGCTCAGTCGTGTCCAACTCTTTGCAGCCCCACGGGC
TGTAAGCCTCCAGGCTCCTCTGTCCATGGAATTCTCCAGGCAAGAACACTAGAGTGGACTGCCATGCCCT
TCTCCAGGGGATCTTCACGACCCAGGGATGGAACCCATGCATTGGCAGGTGGATTCTTTACCACTGAGCC
ACCAGCGAAGCCCCTTCAAAGACTTTAGCACCATATAAGAAGAATGTAAAATGTCTATGACTAATTTTTT
CTACCGATTACACACATTGGTACTATTTGGGGCTAAATATTAGTTTCCTGTCACCCTGCTTGTTTAATCT
ATATGCTGAGCACATCATGAGAACCGCTAGGCTGGATGAGTTACAAGCAATGGAATCAAGATAGGCAGGA
GAAACATCAACAACCTCAGATATGCGGATGACAACACTCTAACGGCAGAAAGTGAAGGGGAACTAAAGAG
TCTCTTGATGAGGATGAAGGAGAAGAGTGGAAGAGCTGGCTTAAGACTAAATGTTAAAAAAAAGATCATG
GCATCCAGCACCATTACTGCATGGCAGATGGAAAGGGAAAAGGTGGAAGTAGTGAGTGACAGATGTCCTC
TCGGGCTTCAAAATCACTGTGGATGGTGACTGCAGCCTTGACATCAGAAGATGACTGCTTCTTGGCAGGA
ACGTGATGACAAACCTAGTCAGTGTGTTGAAAAGCAGAGGCATTACTCTGCAGACAAAGGTCTGTATAGC

CAAAGCTATGGTCTTTCCAGTGGTCACGTACAGTTGTGAGAGTTGGACCGTAATAAAGGCAGAACGCCAA
AGAATTGATGCCTTTGAACTGTGGTGCTAGACAAGACTCCTTTAAGTCCCTTGGACAGCAAGGAGATCAA
ACCAGTCAATCTTAACGGAGATCAACCCTGAATATTCACTGAAAGAACTGATACTGAAGCGGAAGCTCTA
GTATTTTGGTCATCTGACGTGAACAGGCAACTCATTAGAAAAGTCCCTAGTGCTGGGAAAGATTGAGGGC
AGGAGAAGAAGTTGTCAGAGGATGAGATGGCTGGATGGCATCACTGATGCAAAGAACATGAACTTGGGCA
AACTCCAGGAGACGGTGAGGGACAGGGAGGCCTGGCGTGCTGCAGTCTAAGAGTCGGACACGACTGAGTG
ACGAAACAACAACAATAATTAGTTTCACTTATAAAAAAATATGGCTACTGGAACTTAAAATTGCATATGT
GGCTCACATGGTATTTCTGTTGGACAGCATGGCTCTGAGATTTACAAATCTGAGTGAAAATTATGGCCTC
TCATCTCCCTTTCTGTGTGCCTTGAGCAAGCTAAGTAAACTGGCTGAGCTCAGCTAATATCGTAACAGCA
CTGACGTCACAGAGCAGACTGGAGAAATAAATAAGATGGCGTGTTTAAACCGCTTATGTCAGTATGAGGA
ATATGGTTATCGTAAATAAACTCTGGCTGGTGTTATTTTTCTTTCTCCTCCCCCACGCAGAGTACATTCC
AACCCCTTGCGCAGCCAAAGGCAGGCCGGTTGGAGCATCACTGGTGAAGTTGCTTGTGCCCTGATCCACC
GCCCCTGCCCCGGGCACAACACCCTTATCTGAGTCCCCACACTCCCCTGGGTTGGCTGATACCTTCAATC
TCCCTGGAGACAGCTGGTCATACTGCAGGGAGGCTGGGTGGCATCACTGCTCTGTGACCCAGTCTCCCTC
CTGCTCCCAAAAGAACAGTCTCTTCTACCCATGGGTGGTTAAGATACTTCTACCGTGAGCGTCTCTGGAT
GGACGGGACGAGGCAGCAACCTGCTGGTCATGGGCACCAGCTCAGACAGTAACTGAGGTCATCTCTGGGA
GGCTGTCACCCCTTCTTCTTCCCCTCCTCTGCTATTTCTCTGTTTCTGCCACTGGAGGTGTGTGTCTGTT
CCTTTGGGAGGGGGAAAGCAGAACCCTGGCTGCCAGGCGTGCAAGTGGGTGCCTTGCTCTTGTGTGCAAG
TGCAGGTGTGT

>TRIM31|hg19
CTCCTCCGACCTCTTGCCGAGGAGTCATCTCCATTTTGCCCGTGGAGAAACCGAAGGCTCTCAAAGGCGG
ACCCCGAAGACTCCTGAGAGTGGTGATCAAAAGCATGTACTGTTAGTGACTCACCGTGGGAGGATTAACA
CTTAAATTTCCTACAACAGACGCAGCCTCAGAAAACCGGCCTCGGAACTTTCTCCCCTACAGGACCCTCA
GTCCTTCCAGACGGCCCTATGCCGACCCATTTAGCAAGCGCTCTGGGCACACAGCTCACCCCAAACCAAG
GAATGAAAAAACTTTGTTTAGAAGTGAGGCCTGCAGAAACCTTAGGCTACAAACTTTAAATGCATCATTC
TCTCAGAGTTTTCTGTTTGGCTGCTCTTCTGCCTCTAGAGCTGGCTGCAGCTCCAATCTCCTTGTCACAT
CCTGGTCTGCAAACTGAGACCTATCAGGGGCCCTTATGCCGTATGCTGTGCAACTCCTTATGTTGCACTG
AAGCTAGAATCCTCACAGCCACCATGAAAGCTCGTGCTTTCCAGGCACCAACCTGACAATGTGCAGGAAC
AGGCTTTTTGTTTCTGAGCCTAGTAAGGAGGGCCGCTCTGACTCCAGCTCTTTTTGGAATATTAGGTGTG
CAGGATGCAAGATTCTATTTCAGCTCTGGGAGTAAAAGGGCAATGTGTGTGTGCACCAGCAAGAAAAGCC
CATATAGACTTTATTACTAATGGGAAAAGGAAAAAAAGAGGAAAATACATTTTCTGAAAGCTAAGGTTCA
GAGAAGAGCAAACTACTGCAAAAACTGAGGCCTGAATCAGAAAGTTTTCTGCTCTTATTAGTGGACTGAG
TAACTCAGACTTCTTCCAGGTGAAGATAACTGAAAAAGCTGGATAAAATATTTTTCAGGTATCTTAAAAA
TTCAGAGGGCCAGTAAAGAATGGCTGGGCCAAAGTTTGCGAAAGGAATCTGTGGATCTGAGCCCAGAACT
TAAAAGTTGCTTCTGTCTGGGAACATTTGCTGATCCTGCATGTTGCTGAGATCCCACCCAGACAACATTT
TTGACAGCCTTTAAAGGCTTACAGAACTAGAAATAGAGTCCCCAAGCCCCCAAAATATCAAGTGTTGCAT
TTGTTTAAGGTGACTCTAGGCTGCAGCGTCAAAAAAAAAAAAAAATCCTTCTGGGAAGAAGCTCACATAA
TCCTAGGCTTTGAATTATTTCTACAAATATGTCTTCAAATATATCTAGCACACAGGCAAAAATAACCAGG
CACCAGAGGAACAAGAAACCCCACCAGTAAGGAAAGCAGGGACGACAGAATTAGATTCACAAGGATTTCA
GACACTGCCATTATTGGCAGAGGCTAACGACTTTGTATATGCTGTGTTCAAGGAGATACAAGCCAGACTT
GAAAGATCTGGCAGAGAACCAGGAATTTTAGTAAGTGGCATAGCAGATGTTAAAAAAAAATAACTTTAGC
CTGAGAGAGTCACGGGTACTGAAGTTATGAAGTCATGGAAACTCAAGTGTCGTACTCTTGTTGGTCTGTT
TGAAGATCACACAGATTTGGAGACAGTAGTTCCTGTTTCCAGTTCAGCTTTTGATATCACAGAATGACAT
AAACAGAAAAATAAAGCATGTAATTTGAACTATATAATTTTTAAGGGTTGTACATTCATCTGGTTCAAAA
AGTAGTATATAAGAACTATGTACATGATTTGCAACCATCCACCCCTTTTCATCTCATCTGCCACTACCCA
CCCCCACCCCTGCCCCACAGGTAAACACTTTTACTAGTTTCTTGTGTATCCTTTTGATAGGGATAGAATA
GGATAGTTATACAAGTAGTTGTAGAAGTCCCCAAAGGGGCCTATAGATAGGGAAGGAAACCTAGGAAACT
TTGGGAGGCCTTTGTAAGTCAGTGATGGCTCAAAAAAGCTATTGGAACATTATTCAATGAAGCAGTCTTG
CTTAATTATAAAACCATACACAAAACCACAAGATATGCCAGAGGTCACTAGATGGAAGACCACCACCCTT
TTACTAATACGATAATCCTAGTTTGACCTCAGAGGGTCAGACAATTTCCTACCTAATATGAAGGAGGAGT
TAGTGAAGGAGGAGTTAATGCCTTAACGTCTGCATTATGTCTGCCTGAAGAAAGCGGTCTTTTCCCCTCT
CCCTTTTTCCACTATAAATTGTAGCCCACTCAATTCAGGGCAGAGCTACCTCGCCTGCCCGCTTGCATCT

CTTACAAGCGTCCTGTGTTAGTAAATCCTATCACCTTGCCTCTCGCTGAATTCCTTCTGCGCTGAGACAC
AAAGAACCTGAACCTCAGTAAGTCCAGACATTGGGTGAGTGATTCTAATTAAAAGATTGTGGGTTCAAGT
CCCAAGCAGGGTTTTGGCCGGGTTTGAGTCCCAACCATGTGGGTTCGAGTCCCAAACTGGGTTTTGGCTG
GGTTTGAGTCCCAGCAAGTGGGGTCAAGTCCCAGTCTGAGGTAAACAGTTTCAATGTTTATGTAGGTAGA
AGTAAATACAAGTGTATTTATACTATATATATATAAACATATATAGATTTGTACCTTTTCCCCCTTTAAA
GCGAAATTTTGCCTGCCAAATAATATTCTTTTTCACTTTTATAGCCTAGCTATCTTTGTCATGTCAGTAC
ATAGAGCCCTTATAAATTGGTTCAGTTTAATATGGCAACAAAATTATCTTGCACCAGGTTAAGTGCTGAA
GTGATCAGATGGAAAAACATTGCCATGAGTAAGGACTTTTAGTTCCCACTGTCTTAAGGGGAAAGTTTTT
TCTACCATGTGGCTTTGGGAGGGGTTTTCTAGAATTTGTCCTGGGTGTTTATCCAGCTGCTTCCCCATCT
GTCTTGTGATTTGGACTCACTGGAGCAGCTGGCCAGCATTAGATTTGTTGTGGACTGGAGATGCTGGAAC
TCCAGTACTTTGGCCACCTCATGCGAAGAGTTGACTCATTGGAAGAGACTCTGATGCTGGGAGGGATTGG
GGGCAGGAGGAGAAGGGGACGACAGAGGATGAGATGGCTGGATGGCATCACTGACTCGATGGACATGAGT
GTGAGTGAACTCCGGGAGTTGGTGATGGACAGGGAGACCTGGCGTGCTGCGATTCATGGGGTCGCAAAGA
GTCGGACACGACTGAGCTACTGAACTGAACTGAACTGGAGATGCTGGACAGTAGCCCAGGGGTTTCTGGC
AGCCTGGGCGTCACAGCTTTTCTGTGTCAGGTCTGGGTCAGAGTACCTTGCTGTCTCCTAGGAGAGAGGA
GTATCAGCTTT

>RNF39|hg19
AAAATGGAATAAACCAAGGGAGAATTAGAGGATCAAGTGTAGAGTTGTTGGAGATTCTAGCAAGGAAGGG
AGGAAGTCTTGAAAAGTGAGACATTTCCTGGCGGTGAAACTACTGAACCACTGAAGCTCATGTGATCTAG
AGCCCATGCTCTACAATGAGAGAGTAACCCCTGCTCACCAGCAACTAGAGAAAGCTGGCACGCAGCAACG
AAGACTGAGTGCAACCAAAAAGTAAGTTAATTTAATTAAATTTAAAAGACAAATTATCATGAACTTTTGT
TTGAAATCTGTGAAACAACACACACTTGTTATCTTATAGTTCTGTAGTTCAGAAGCCTAAAAGGGGTCTT
ACAGGGTGTCAGAGTGCTGGACTCCATCTGGAATCCATCTCCTTGCCTTCTCCAGTGTCTAGTGGCCACC
TACTTCCTTTGCTCACGGCTCCCCATCCTCAGTCTTCAAAGCCAGCAGTGTCAGGCTGAGTACTCTCATG
ACTCTTTAATCCAAAGAGGAAGATCGTCCTCTTCTGCTTCTGTCCTCCACCTTTTTTGGCCACACCACAA
TGCATACCAATTCTTAGTTCCCTGACCAGGTATCCAACCCGCATTCCCAGCAGTGCAAGTGTGGAGTCTT
AATCACTGGACTGCCAGGGAAGGCCCTGTCCTCCACTTTTAAGGACCTTGGATAATCTAGAATAATTTTA
TGACTTAAAACTCAACTGATTAGCAACCTTAATTCCATCTGCAAGCTTAATTCCCCTCTGCCATGTAAGG
TAACATGTTCACAGGTTCCACACCACAGGTAACATGTTCACAGGAATTAGAACATGGGCATCTCTGGGGG
GATCGTTATTCTTCCTATCATAACATGGAAGTCCATTTCACAACATAGATTTTGGTTATATCTGTAGCCA
TTTGATTGCATAGCTGTGCATTTTATGAGAGGGGAGCTGGGAGATTTGTTTCAAGATGAGTGTATGTGTG
TTCATTTGTGTACTTTGTTTATCAGAGTGTCATGTTTGAGTGTCTCCGTGATAAGTAAAGGGGGAGAAGG
ATGCCTGGAGGGGTTGCCTAATAACCTGCTTCTGCTCCATAAAGACTTACTGTTTTTGTATTGTTGTTCT
TGTTGACAATAAAACTTAATGACAGAGTGTCTTTGCATGTTTTCTTCCAGTATGTCTATCTGTTCCTAAA
TAATCCGGGTGTCCATCCCAGTGATAAAGTGTGTATGAGACCATTTGTGGCATATGTGTGGTTTTGCACT
GTATGAGGGTATCTGACGATTAGGGCTGTGCATTTGGGGGCATTTAGACATTGTTGGATGCGCATGCATG
TTTGTGCAACAGTGTGTGATTGCAGTTGTAGACATCTGAATATTTACTTATTCACAACCCCCCAGGAGTA
CCCAGACAGAAAGGCCTAGCATGAGAGAGGCAGAGGAACTAAGATAAGGGGGAGAGAACAGGAGGGTGGA
GGAGGAGAGAGACAGAGATGGAGGTAATGAACAGGGCATGAGGAAAAATCCAAAAAAAAAAAAAAAAAGA
AAAAATCAGAACGTAGAAGAGAAGACTTTCCTGGTGGTCCAGTGATTAAGAATTCATCTGTCAATGCAGG
GGACATAGGTTCAATCCCTGGTCTGGGAAGATTCCACATGCCACGGGACAACTAAGCCTTCTGTGCCACA
ACTACTGAGACTGTGCTCTAGAGGCCAGAGGCCACAACCACTGAAGCCCGTGTGCCTAGAGCCTGGGCCC
CACAACAAAAGAAGGCACTGCAATGAGAAGCCTGCTCACTGGAGCTAGAGATGGACTGTGTGCAGTAACG
CAGACCCAGCGCAGTCAAAAATAAATAAATACGTTTTAAAAAAATGTAGAAGGAAGTTCCATGCAAAGAG
TTGACTCATTGGAAAAGACTCTGATGCTGGGAGGGATTGGGGGCAGGAGGAAAAGGGGACGACAGAGGAT
AAGATGGCTGGATGGCATCACCAATTCGATGGACATGAGTTTGAGTGAACTCCGGCAGTTGGTGATGGAC
AGGGAGGTCTAGCGTGCTGCAACTCATGGGGTCACAAAGAGTTGGACACAACTGAGTGACTGAACTGAAC
TGACTCTAGGGATCTGAAATTAAAGAGGGGCAGGGTGGGGTCACAGCAAGGCCATAAATCAGAAGATCTG
GGTATGGCTTCACTTTGGGTCATCACCTGTGTGACCTTGACAATGCCAGCTGTCTGGGCCTCAGTTGACC
AAAAGAGTGAAAATAGATAACCTTTGGGTACACACCAAAGAAGGAGAGGAGAGAAAAGTGGAAATAAAAA
CATACACAGGGAAAAGAGACAGAAACAAGTAGAAAGAGAGATAGAGGAAGAAACAACAGAGGCCTGGGAG
CCAGCCAGAGGTAGAACGGAGAGAACCGGACCCGGACTCCTTGAGTCAGGAATGTGGGCGCCTGCTTCCC

ACCCCTTACCCCAAAGCTTCGATGTCCTTAAGCCCTTAATAGGTAAGGCAGGCAGATGGAGAGAGGCGCC
TGGAATGACAGCCACTCGCCAGCGCTCAGAGGCCTGCCAAAGCCACACAAACCAGATCTGTCTTCAGTGC
CCCCAGCCCCCCAGATGTAGGTGTGCCAGCCACCTGGAATGACTCATACCCTGGCGATGTGGGCACACAT
ACCAAAAGCAAGCCTGGGCATCCTGCCCACCTGCACAGTGCAGGCCAAGGGGAAGACGATATCCCGGCTC
TTGAGGGAGGTTGGGGTGGTGGTGTCAAGGGCTACGCACCCCACATCTTGCTACTTTGCTACCTTGCCCC
CAGAGCGCCTCCTAGCGACCACCTGCTCGGGTCGAGACCTAGGTGTCCCTTCCACCCTCCCCCCCCCGAA
TCTTACCGGGAAGCGGGAGAACCAGATGGGGTGTTACGCAAGGCCTGATGTTTACCTCCCGCAGGGGCCT
CCTCCCTCCCTATAAAGCCTGACGTGGTGGGGAGATTGGCGGAGACAGACTGGTTGAAGACGGAGGCGAT
CCCAGGAAAGCCTCAGAAAGTTAATGCGGGAGGCGGAGAGCGGGGCAGAGACGCAGCCAGCAGCCGAGGC
GCACCGCCATCTGCGCACCCGAAGGCGGTCGCGGTGGTCGCGGAGGGGACGGCGCCCGGGCGCTGGCTCC
CCATGGAGGCGTCCGAGCTTCTGGGACCGGGGCTGGTGGAGCGTCTGGAGCAGCTGGCGACGTGCCCGCT
GTGTGGGGGCCCCTTCGAGGACCCGGTGCTCCTGGCGTGTGAGCACAGCTTCTGCCGCGCGTGCCTGGCC
CGCCGCTGGGG

>ENSBTAG00000058345|hg19
CCTGACTACCTTCCATGCTTACAGCCATTTCCTGTTAAGAATCTTCTTAACCAACAACCCACTCCCCACC
AGCCCCACAGCCAGCTCCATATGAGCATGGCACATACTCATAACACAGAACCAATGGTGCTACCTCTCCT
TTTCTCAACCTGGGCATGGCCCAGACACCCCAAATTTGCCCTTGTATATACAGCCTCACTTCTTTCCCCA
TATGTATAAATGGGTCCATATTTAACACATTTTGTGATGTTGGGTTATTTATTTTGTGCATCTGTGGCAA
TAAATGAGATCTCAGTGGTGGTATGGATTTGACTGATCTCTGCAACTGTGCACTGCAAAAGGCCTGGAAA
ATGATATCCAGATCCCAGTAAGGGGTGGGGAGGGCTGGGAGGGCTGGATATCTGGGCTGGGAGAGACATC
AAGGCCTTGGAAGGAAGGGGTGAGATTGCATCTCTTGGCTCCCTCCCTTTTCCATCCTCTCCCAGGGACC
CAGGCCCCTGGGTCGGATCTGTCTCCTAAGTTGGAAGACCAAAGGCTGAGGAGATAGTGGGCTCTAAGTG
ACTAGTCTTGGAGCCAGTTTTGTCAGAAACCCTAGTCACATGAGAACGGTGAGGAGAGAGGACATGCCCA
TTTCTTTTTTCCATTTCTCATTAAAGGTGAGAAGGACAAAGACCTCCCTCTCTCCTAACTCCTCTTAAGG
GCTTGGGGGAAGTGGGCAGGAGAGAAGGGAAAGGGCTAGGAGCCCTGAGGTCTCACAGAAGACCTCACAT
CCCACAGGCAACGACTAAGAGTTAGGATGACATAAATACATAAGTGGAAGAGCTCTAAGCCAGGATTAAC
AAGCATAGCTGGAGCAAGATTGCCTGGAATCTGACTAGGACCCTGGCAGTGAAAGCTGGGGCTGCTGGAA
ACCCAATTTAGCTGTTTGGAACTCAAGTAGAGAGTGCAAGGGGCCAAGATAACTGGATCCCAGGACAGGC
AGTTTGGCAGTGAGAACAAAGGAAAAAGATTGGGATCAAGATGCCTGGATCCCTGGAGAGAGGAGGCTGG
GTATTGTAAGGAGAAATGAGGGCTGGGGGAACCCAGAAGCCTAGGTTCTGGAATAGCAGCAAGTCAGAAT
TCCAGGATCTCTGTCCACCTTCCTCTCCCTCTTCCCTCTGTCAGGCAGAGGAGAGGGGAGGAGGGGGCTG
GAGAAGGGATCAAATCGTGGGTTTAGTCCCCGAGTAGCCACATGAATACATTCAGGGCCAGACAGACACA
GGAATGGTGGGGGAGCGAGGAATTTAGTGCTGCATTGGCCCTGGAGGGGGCTGGGAGGGGTCAGGAGGCT
GGGGAGGGCTGGTGGGGCTTGGTCCCGGGGTTGCATGACGCCGTCCTTTGCGGTGGCCCCGCACACAGTG
TGTGTGACCACAGCCCTCCTCTTCCTCGTCATCACTCTCTGTGGAGCTCTCGCCAAAGGCCCGAGGTTTC
TCATAAATACAGCAGCCTGGATTTGGATGGAGGAGCCCAGTGAAAAAGGAGGAAGAGTTCAGATGTAGTG
TCAGTTCCATTCCCACTGGATCTCAGTCAAAATTCCTACTTCCCACCATTTTCTCCTTCTACCTTCCAGA
TCCAGCTTCCCCAGGCCTACACTCTGGGACTCTGTCACCTACCCACCTTGCCTTTCCAGCCGGGAATCCA
AAGCTCCTCTTCTCCCACCTCAGATACCTTTGACTTTAGGCCTTGCACATGGAAATTGAAGCCCCTCCCT
ACCTCTTTCCCTCTAACTTTAACCTCCTCTCCCTGATAACAGAGAACCACCAAGGCAGCCACAGGACTGT
GAGCATCTGGGGGCTTTGGGAAGGCCAGTGAATGCCTGAAGGCAAAAGATATGCTTAGGGGAGCCAGGAC
TCCAGCGTTACTGTGGGGCAACAATTACTCACATTTTGATGAGCGGCGGCCCATGTGTTCATTGTCCACA
GTGTCACTCGTCCATTCCACCTTTTTCTCTGGCTTCCGTTTCCGAAGTTTGATGGTTAGGCTCCGGTTCT
CCTGGAAGGTTAAGAAGGATAGGCATACCTAGCTAGTCCCCTCCTACTAACTCTTCCTGCGTCCTCCCTG
GGGCCTCCTTTCTTCCCTTTCCCAGTTCAATCAGGATCACAAACACAGCTACAGAGGAGCTGATGCTGGG
AGGAGATAGAACAGGTTTTTGACCTCAAGGAGTCCCTTGAGGAATTCTGTTCTTAGTATTCTTAGTAACC
ACTAGTATACAGAGAAGATACAATTCCTAACCTAGGGAAAGTCCCAGCAAATCTTCAAAGGAAAATATAA
TCCTCGCTGTTCCTCCATCTTCCTCCCCAAATCATCTTTGGTGGTATTAATACATGAAAATTATTTTTTC
AGTCACCAATGCCTAAACTTTGATCTCCCTTTCTTTTCCCAGAACGCCTATTCTTTTTCCCATTAAAGAA
ATGACTCTGTTCTCACATCTCTCATCCTCAAGCCCAGCCCTCCCAATCTGTTATCCACCTATTCTCCAGC
ATCCTGGCATCTCTAGTAACTATTACTCTAAACTCCCTTAATCAAAGGGTTATTAAGCCCGACAGGAACA
TCTCCTATTGAAAGCAATGTTTTTGCAAAAACGCTGTTTTTCTCTACATCTCTGATTTTTCAAAATACTC

CCATCATCTCCCTTTTCTTGTTCCCTCATTCCCTCTGCTTCCGTTTGCAATAATCTCTTCAAGAAATATT
TATTGATAAGCATGCTTAGTGTCAAGTTTCCCTACAGCGAGCTTCCAACCATGCTTTCTCACACAACCCT
TCATTTCCCCCAGTACATCCCTTGCTCATTAATCGCCAAGTTCCCAGTCTTTCAATACTTACAGACACCA
CCATCCCAAGCAGTATCACCTACATCTTAACTCTATTGCATGTCACTTGACCAGCCTTCTGCACCAACTC
TGACACACCAAAAAATCCCTAATACTCTGCGATTCTCCCAAACCCACTTCCTCCCATATCCCCAGGCCCA
GCCCATCCAGCAACTCTCCAAAGCCTTATCCCTTCCCTCCCGGTATCTCTCAGACCCCTAAACAGCACCG
CCCCCAGCCTTTCTCACGGGCTCCGTTGTCACGGTAACCGTTGTCTCAGTGACGGTCTCACTCAGCCCGG
CCCCTGCCTCCGCCATGGCTAAGGCTGAGGAGAGAGAGAAGCGGAGGAAAGGGGATGAGGCACCCTTCTG
TCTTTTCTGTGTCTCTGAGGTCTAAGAGAATCGGTATCGATTCAGATTAGCAGCCAGGATCCTCCACCTT
CTTTTTTCCCC

>ENSBTAG00000054588|hg19
ATTAAAAAAAAAAAGTTAAAAAATTTTAATTTTCTTAGTTGTTTAAATTAAATTAAACAATTTCTCAGCC
TGACTGAGAAATACTCCCCAAAGGCATTTCTAACTATAAATATTTTTAATCCATTGATGAGAAGGCAAGA
AAGTAAAGGCACTCTTCACACTGAGTGAAAGTATATAAAACCAGGAACCTAGACAGGTACATTCAAACTG
AAGCCCCTGGTACACTGGTGCCCTTGCTGTTTTGAGAGCCACATGAGTGAACCGGCGACAAGCCTACAAT
GTCTGAGAGGAGATACTCTGAGTAAGTTTGAAACCTCCAAAGGGCTACACCTGTGTATAAAAGTGAACAA
GAAAAAAAATCCAACTCTGCAGAAGAGGAATTTGACTGTCTCACTAGGTAGAGAGAGAGAAAAAAAAAAA
ATTTCCCTCTAGAATTTCTAATCAAAACCTGGCCTTACATTGGATTTGCAGCCCAAATCCATACTACCTA
TTTGCTACAAAACACATGAAGCTGAGAATTCATTTAAAGTCATTCCAACTTGGTAGTGCTTCAAGGCACC
TGAAGAGGAAACCCATATGCTAAGAATATAGTTTTCACTCCAGGCCTCAAAGAATTCCATAGTCCAAATT
CTTAAAAATCTGAGCTTATGTTCAAAAATTATAAAACAAAAAAAGCACCATGAGAAAGTCTGCAGGAATA
ATGGCAGCATCAGACCCACAAAAACTGTGGGTATGAGAATTAGGAGACACAGAATATAAATATCATGCTT
AATATATTTAAATATATGTAAGAATTAAAAATAATTTAGGATTAAAGAATCATGAATTATTAAAAATGAC
TGAGGAGACTTGAAAAATAAGCAGAACTCAAAAAAAAAAAAATGTTTTTAAGTAACAATAACTGACACAA
ACTGAAGACAGAATTAGTGAAGTGAAAGAAAGACCAGAAGAATTTACCTAGAAGCAATATAAAGAGAAAA
TAGATGCAAAATTACGAAAGGGTGAGAGAAATGGAGTAGTGGGTAAAGTCTAATAAGCAACTAATTAATT
AGAACTCCAGAATGCGAGAACATAGGTAAAGGCAATATTCAATGAGATATGGCAGATAATTTTCCAAGGT
TGATGGAAAAGAAAAAAAAATCAATCTTTACTTTCAGAAAGCCCAACAAATCCTAAACATGATAAACAAA
AGAAAGCCACATCCAGATATACTTTAGTGAAACCACAGAACACCAAAGACAAAGGGGAGTTCTCAAAACT
ATTCAGAGAATCATGTTCTTCAAATCACCCATGACTAATTCTCACCGAAATAAAACTACCTTGAGTAAGT
ACAGTGGAAGATGCAACAGTAACACCCAGATCATCAAGCAACATTTGCTTTCTGTATTTTTTTTCTTTCC
TTTTAAATATCTTAAAAGGTTTAATTCACCAAGTATAAAAAGGATAAGAAAAGTGTAATTTTCTAAAATA
AAAAACGACCACAAGTTTCCCTATGACTTCAACTGTACAGAAGAGGGATGGGACCAAAGAACACAACTGT
TTATTCAGTACAAAAAGCAAATCACTGGATTCGTTTAGGTCGCAAACAGACTGAGAAAGGGCAGAGAGGA
GGGGAAGGATACGCACTTGCAGTTGGTACAAGTGTAGAAGACAGTCTGCCCCTCATCGGCTGATCGCATC
TGCCTGGTGTGGTATGCCATTCCCTCGTGCCCACATCGAGAGCAGCGCCTGTCAACCTGAGAAGCAACTG
GTGGGATTAATAAGTCCTCTTCCCGATCACAGACATCGGGCTGTCACACTCCCCAGACTCTGATGTTTTA
ATAGACCCCTCTTCCCTCTTGGAGGACTTCCCACGTGGCGCAGTGGTAAAGAATCCGTCTGCCAATGCTG
GAGACGCGAGTTCCATCCCTGGCTCGGGAAGATCCCCTGGAGGAGGAAATGGCAATCCGCTCCAGTATTC
TTACCTGGAAAATTCCATGGACAGAGGAGCCTGGCAGGCTACAGCCCACGGGGTCGCAAAGAGTCAGACA
CGACTCAGCATAGCACACACCCTTCCCTCTTGGAATTGGGTACAAAACTGTCCAGATCTGAGCAATCGCC
CCTCCATTTCTTTCCCACCCTCCTTCGTTGGGGCCAGTCCTCGATGTCATTCACTCACCACGGGTCCCTG
GAACTCAGGTCCTTCCTCCATCGACAAAGGCATGGCTGTGCCCAGTTTGTTAAACACAACTGAGGTCTTC
ACAACCTTCCCCTCAAAGTCTGCGCAAGCAGTAAGAACTGGTTGTGGAGACAGTAAGGAGGGCTGACACC
CAAAACTACCTCTCTGTTAGGACAGGAGGCCTTATATTCTTCCCTTCAGGTTTCGACCCAAGGCTAAAAG
TGCCTGATTCCCTGCTTCAGGGTCCCCCTCTTCAATCCAACCGATATTAGGGTCCTCAAGCCACTGACCC
TTTCACGCTGGGCCCTCTGCCGGGGTCGCTGAGTACAAGCCCCTTACCTCGCACGTTGATGGAGAAGCCA
CAGCGGGTACAGGCGACAGCATCCTGAACCCCGGGCAGAGGCAGGACCGAGCCGCAATCTGGACAGAAAT
CCAAGTCCGACTGAAAGCTGGAGCAGATGCCGGCGAGGTCCATGACGGAGGGTGGTCGAGGGCTGAGAAG
AAGGGGAGACTGCTAATAAAAGAGAGTAGCTCCCTCCCCACTGAAGGGTCATTCCTATTCTGTCCCTGGA
CCTAAGTGTACAGAACCCGAGTCCTTCTCCCACGTATGCCTGTGCCTTTAAGACCAATGAGCCCACATAT
CTAGAATTCCAAGCCGGGGTCCGGTACTTCGACTTGGGGGCTTGCTTACCTGGGACAGGCGAGAAGGGGA

AGCCAACTCCGCAGGCTAGAACGCCGGCTCTACGCGGCAGCGAAGTAGCGAGAGCAGGGCGGGCTAGAGA
GCCCCGTCGTTCGGCTTGTCGATTGGCTGTTACGTCATAATTAATCGCAAACTTGGTCCCCGGAGGGAGA
GGAACCATTGAAGGAAGGTTGTAGCGCTACCTTCAGGCTGGTTTTGTGACGTTGGATAAGAAAACAGGTG
ATTGAAGCTGAGCCTCTGGTTGTTTTTCCCCTCATTACCCAGTTCTGGGAACTACAATTTCCTCTGTTCC
TGAGTTGGTTTAAAAAAAAAAAAAAAAAGCGAAACATTCTGGGAGTTACAGTTCTAGCGCTCCTCGGACCT
CGCGATGCAAGCAAGGGCGCTCTGCAGAGCACCGCCTGGCTCGAGTGCTGGGCCCTTGGCGACGAAGAAT
TGATGCTTTTG

>ZFP57|hg19
GTCCTGTTAGACTCTTTAGTGACCCCATGGACTGTAGCCAGGCTCCTCCATCCATGAGATTTTCCAGGTA
AGAATACTGGAGTCGGTTGCCATTTCCTTGTCTAGGGGATCTTCGCAACCCAGGGATCAAACTCAGGTCT
TCTGCGTTGCAGGCAGATTCTTTACCATCTCAGCTACAGTGGTGAAGAACCCGCCTGCCAGTTAAGGAGA
GGTGGGTTTGATTCTTTGGTCAGGAAGATCCCCTGGAGAAAGGAACGGTAACCCACTCCAGTATTCTTGC
CTGGAGAATTCCATGGACAGAGGAGCCTGGTGGGCTAGAGTCCAAGGGATCACAAAGAGTTGGACATGAC
CGAGCTACGAACATATTCTTTACCACTGAGTCACTTGGGTAGCCCAACTGTGTGTTTTCATCAGAAATAA
TTCAATCCCTTTTCTCCCAAGAGTGAGTTAAGACTGGAAATGAAATCCTAGCCCTGGGTGGGACCAGCAG
CTACAATTGGTAGGAGGGATGGGCTAGTGGGATGGGCGGATCCTTTTTCTTTTAAGCTTCCTAGATTGTT
TTGAACTCTTGACTTTGGCGGTCCCACAAGCAAAGCCAGGCAGGGTACGCGGTGGGTCTGCGGAAATCGG
CGAGGAGAGTGGGGTCGGGACGGGGTCCTGGCAGGGCCGGGGCGGGGTCGCGGGCTCCCGGTACATTTGC
GCTGGGTTGGCGCGGCTAGCCGCTGGCCCACCGGAAGTCCTACGTCCTGGAGCCCCGGTCCTCGGCAGCG
GCGGGTGAGTAAGGGCAGCGGGGAGTTGAGCTCGCGGTGGTGGAGGCTAGTGAAAGGTTTGTCCTGCGAT
CTTCCGCCCCTGACCTCGGCAGGCGAGGGGAGCATCGCGCCCCTAGTCCTGGAGGAGTTTCCAAGCCCTG
AGGGCCCCTGGGGAAACCTGGACTGGTTAGGGTGTGGTCCGGTGCGGCGACCACTCACTCCCTGGGGATT
CGGCCAAGAGCACGGGCTGCTAGCATGTGTTGAAAAAGTCAGGGTCCCAGCAACGAGGTTTCGGGGCAGA
CGGGAATCGGGCAGTGCAGTTCCCGTCAGCGGGACCGGTTCAGTGGGTACTGGGTAGCGGGCCACGCAAT
CGTGAGCGAAACTCTCCGTCCCTGCCCGCGGGAGCCTCCGAGGCTGGGGTCCGAGGCCGGGCGCGAAGTG
GCCGGAGGTGAGCGCCATGGGCCCGCGATCTGACCGGGCAGAGCTAGGACCGAGCAGAGACCCAAAGGCC
GGATGAGAAGGGCGAAGGCCCTAGGGTCGTTCTGCTTGGCCGGAGGGCAGAAAACTCCAGTGGCTGAAGC
AGCGGCGAGCGGGGCGGCAAGGGCGGACCCCCGAAGCCTGGAGCGGGTGTTGGGGATTTCAGTGGTTCTC
AGAATAGCTACAGGAAACACTGGACGAGTTTGGGGTTAGAGATCGGGTGGACTCAGAGCACTGGAAAATG
GCGACACATTCCCTCGAGGGGTTTACGTTCTTGGAAGGGCGGGGACGGGCCCTGAACTGATAAACAGGTC
TGCGCCTAGAGTCGTGTTACCCCAGCCGCCTTGCACTAGCCGGCCAACCGGTTTGAGCTCTGGGGCGAGT
GGATGTAGAAAGTACTATCGAGGTCAACGTGCCAGGGGCAGGCGGCAGCGGCCAGAAATTCAGACTCCAT
AGTTATGCTTTCTCCCCAAACCAGACAACCAGCCTAGAATCTGGACCGAGCGCCCCTGGCCACCACCCCC
AGCAAATGCTTTTGAGAAAGACTTACAACGGTGGGTCTTTCCTGAGGGAAAACAAGAGAGCCAGAACTTT
GAGGATACGATTATAGAGACTTTTGAAGTGTTACTTTTACTGTGTTCCACGGGCTAGTAGGAGAATAATC
CAGACCTGCACCATCTCACCGCTTGATTATGGATGGGCTTGAAGCGAAGGGAGGGAGGGACCAACTTCAC
CTCCTAGGAAATGCACCTTTCCTGTCAGCTTTCTTTTTAGCAATCCTATTAACTTCATTGTTGAACGCCC
CTTTCCTTTCTGGCATTTTCAATTCTACCACCTTTCAAAAAAGTGAAAGGAAGAACGTCAACAGGTTTTT
AAGTCTTCATTCTTTTCATTCCATTCATTCCATCTGGAGGGAGGCCTAGCGGGGTGAGTGGCAGTCCGCA
GGAGGAGGTGGGAGTTCCACCATGTTTGGGCAGAGCAGGAACTTCCCCGGTTGGACCGCAGCTCCAGCGC
TGGCTCCCTGGGAGCCAGCCCTGTGTAAGTGTTGGTTGTTGGGCTGTTACCCACTTTTTTATTTTAACGG
TCGTTAAACTAACTCTGCTGTGGGGCATGCAGGATCCCCATAAGGGATCAAGCCTGGACTTTTAACCACT
GGGCCAACAGGGAAAGTCCCTTCTTTATTCTTTACAAGGATCTGTATGTATTAAAAATTCACAGAATCAT
CCTAGATGGGATCTTAGAGAATCTCCTTTAATCTTATTTTCCAGTAAAACCACATCAGAATGCAGACACT
TGCACAAGGCTGTTTGTGACAGAACCAGGGCTGGTTACCTCAGGCATGTGCTTTCTTTCCAGAATACCAT
ACTGCCACCCTAGAGGGGGCTTTGGGACAGCGGGAATCTTGTCCTCGGTGATAGAGCCCAGGCTTTGAAC
ACTAACTGCTGTGGACTGGGAGCATCCCTGAGCAATTACCTAGCATCTCTGTACTTTAGATTACTTAAAA
TGAGGTTGTTAATCATTACCTCATGAGGCAATACTGAGAGGTTTTAGGTACAAATAAGAGGTTCAATAAC
TCTTGGTGTTTCCTGACCAAATTAGGAAGCTGTTGTTCCTCACCCCCGTCTTACTGATAAGTTTTGTTTC
TCTCTGACCTCAGGTTCCTGGAGGTGGGCAGCAGCTAGAAGCAGGGTGAAGCAGAAGGCAGACGACACCT
GCAGGGCTGCAACCTGGCATTACTGAATAGTGACTGCTGAGGCAAGGGGGACCAGGTCCCTGAACTCTGT
GGCTGAGGCTTGAAGGGAATCAAAGGTGCTCTCAGTTGTGAAAGAGGAGAGTTTGGACAGCAGAACAAAA

AAGGAAGATAGCCCCCTGGAGCAGATATCTTTGACACAGGAGGCCAATTCAAACCCTTTGCAAAGGCCTT
TGCCAGGTCTCCCCATCTCCTCAGTTGCTGGAAGGTAGATAGCCCCAGGATGGAGCCTAGTCACCCTTGG
GGGCCAGACCAGGCATGGATAAAGCTTAAAAGAGACAGCATGGAAGAGAAGGTGTCTGACCAGCCAAAGC
CAGTGGAACCT

>BOLA-DQB|hg19
TTCAAAATATGCAATGTATTAATATTTTGTTTCTTGAATTAAATGCAGAATAGGGTCCTGTGTGTCTCTT
ATGGTGACAAAACAATCATTTTGTTAATGATTTCACAGTTAACCCCACTGACCAAGTACAGATTCACAGT
TAACCCCACTGACCAAGTACAGATTCCAAGGCATGCTGACCAGGGAAGATGATTTCGCCCTAAATTTCCA
CAGGAAGTAGCACACAGCTTTTGTCTCCCACTCTGAGGGAACAGAGGATGAACCAAGGCAGTCTTCCTAG
AATGACCCTGACCCTATCAATGACTGTTTCTTGATTCCAGCATCTACTCACATGGAGTACATCCTGCCCT
CATGCTAAGCTTCTTGCCTCCACAGGGTGATTCATGTAAATACCTGGAATCAGAATGTTTGTCTGTGTTC
TGTCTTCTCAGGCCAGGCAGCAGTGACTGCCCTGAGTAACTCAGCAAAGATAGGACAGTGCAGAGAACTC
TACAACATGTAGAGAATGCTACCTGGCTTGGGAGAAGCAGCGGGCAGAAGTCCAAACCACAGCCTTGCAG
ATGAGGTTTTAGCCAATCAGAAAAAGGATTTTAAGCTGACACTATTGTTGCTAGTAGAAAAGTCTGTGTA
AAGAGGCCCATGAGACCAGACAATCCCCTGGTCTGACACAGCCTCCACTGCACAGCCCCAAACAGAGCAG
AGGGAATTTCCTCCTTAGGACAAGAGGGAGGGGAAGGGCCAGAAGCCACTGTTTAATCCCAGGGTCCCTG
AGATGTAGTCTGAGGGAACTGAGGCACCTTCAAAATCCCTCTCTGACTTTTTTTGTTTCCCCTTCAGCCT
AATTACTGGTTTGAGTTTGCTGTTTTTCCTTTATGTTTTCATCCACCCTGGAAACTATCCTAGCTCTGGA
GCTACCCTGGACCTGAGTGCACCACTGCAGGTGGAAGCCCAGTGACAGATTCCCAGTCTTAGGTGCTCTG
GAACTATGCACCTAATTAAGAATATTGAAATAACTTACTGCCTTCATGTGCCACTAACAGAAACCTCTTG
CTGTTGTCTCTTCCTTATTTTGTTTTACCTGGTAAACTTTTGATGTTCTGCTTTTAGTTCCTGTTCTCTT
TTTGAAAGAGTAACCTAAAATTTAGAAAGAGTGAGATTAGAAGGAGAGATACTTACTGTCTTGTTTATCA
ATGTTTTCCAGGCACTGTCTAATTGCTCTGTAAACATCTGATACTGCCCCTAGTCATGGGCAAGAGGAGC
CTGATAAATAATACATTTATTAATAAAACACATTGATAGTTCTGCCACTGGGGACTTGATTCTTAGGGCT
GGCGCAGCACTCTAAGCATCTGTTTCATCATTAACACTATCCAAGCCCCAGGAGGTGCTTTTCCACAGCT
TTTGCTCTCTGTTCTCAAAACTTAAGGTGACTACCTTTGAATGCTATTAATGCTTGTGGATTGTGCCTTT
GCTGACATTAGATGGTTCAGGGGGGATTTGAGGAACTGAAGGGATTTTAAGAGAAAACTCAAAACTGTCC
AAATATTTTCATTCAAAGAGCATAAATGCCACCGATTCTTGTGTAATTAAAAATCACTTGCCAGCAGCTG
TTTGTCAATTAAAAAATAGTAAAAAAAAATTTTTTTTGATCATGTCTCGTGGCATGCAGGATTCTAGTTT
CCCAATCAGAGATTGAATCTACATTTTCTGCAGTAGAAGGGCAGAGTCTTAATCACTGGACTGCCAGGTT
AGTCCTGGTTGTCATTTTTTTTTTTTTTGGCTTGTTTTCTTGTTTCATATATTATCCTGTTATTGGCAGCT
GTTGCACATGGCAGCTGAAGAATATTTTGCAATATTAAACAAGTCTTATTACTCTTGAAGGCAATGGCAC
CCCACTCCAGTACTCTTGCCTTGGAAAATCCCATAGATGGAGGAGCCTGGCGGGCTACAGTCCATGGGGT
CGCTAAGAGTCGGACACAACTAAGCGACTTCACTTTCACTTTTCACTTTCCTGCATTGGAGAAGGAAATG
GCAACCCACTCCAGTGTTCTTGCCTGGAGAATCCCAGGGACGGGGGAGCCTGGTGGGCTGCCGTCTATGG
GGTCGCACAGAGTTGGACACGACTGAAGCGACTTAGCAGCAGCAGCAGCAGGGTTTATCTGGAGAAGGCA
ATAGCACCCTACTCCAGTACTCTTGCCTGGAAAATCCCGTGGACAGAGGAGCCTGGTAGGCTGCAGTCCA
TGGGGTTGCTAAGAGTCGGACACGACTCAGCAACTTCACTTTCACTTTTCACTTTCCTGCGTTGGAGAAG
GAAATGGCAACCCACTCCAGTGTTCTTGCCTGGAGAATCCCAGGGACGGGGGAGCCTGGTGGGCTGCCGT
CTATGGGGTGGCACAGAGTCGGAACGACTGAAGCGACTAAGCAGCAGCATTACTCTTGAATTTGTATATA
TGAAGGTCTAGATATAAAATGCATGAAATATGATTCACATTTTATAAAGAAAGTCTGAGGCAAACAGGTT
CTGTATTTTGTTGACTCTCATAAAGTGCATCAATGCTAAAATCATTTCCCTCAAGAATTCTGCTCTCTCC
ACCACAATGTAAGGATCCTTAGAGTGTCTAAGATAATAGAAGTGAATAGTTCTGTGACAATACTTTTTTC
TCTTTGAAATATAATCCCTATTTTCCCCTACCCTAATTTCAAGAATAATAGATTTTAAATAAATATCATA
GAGATGTCTACTCTTGAATACATTTTTTTCTTTTTGTTATTTAAACATCTAAGGTATTCAATCTTCAGAT
CTTTTATAACTGAGAGAAAATATTCACCCCAATCCAGTGTAATTTGATGAGGGCATAGTGCCAGGCATTA
GATTAAGAACCTTCAAAAAAAAAAATCTGCTCAGAAACAGATGAGGCACTTCAGCTGATTGGTTCCTGTCA
GCTGATTGGTTTCTTTCCTAGGGACTCTCCAATCCTGCCATACGTGGGAGCTCTCATTGGCTTTTATTCT
GTGAAATGGGCTCACCAGATCCACTGTGTTTGAGCTGTGTTGACTACCATTACTTCTTCCTTTGTTCTCT
ATTATGTCTGGGATGGTGGCTCTGTGGATCCCCAGAGGCCTTTGGACAGCAGCTGTGATGGTGACACTGG
CGGTGCTGAGTACCCCAGGGGCCGAGGGCAGAGACTCGCCACGTAAGTGCAGGGCAGCTGCTCCCTGGAG
CCACCACTCTGGGGAGCAGGCTCTGAGGGACCTTTGAGCTGGGGTGTGATCTCAGAATACTGTCTTTTAT

CACACATTTCT

>ENSBTAG00000019588|hg19
TGGAAAATTTTTATAATGGATTACCTAAGTGTTCATATAGGGGACATATATGAGTAACTTCACAAAGATT
TGGAAAAATTTAAGGATAATGGCTTGACAATTTTATATTAAAGGGAAATAATATTAGGAGAAATAATTAT
ATTACTACTGGCATTAAGGAGGTTATGGAAACTTTCATAATATACAATGAATGTTGCTTCCTGAATTGAG
TGCAAAATAGGGTCCTGGATGGCCCTTCTGGACAAGATGATTATTACTTTCGCAGTTAACCCCACTGACC
AAGAACAGATTCCAGTGGATGCTGACCAAAGAAGACGATTTCTCCCTGAATTTTCACAGGGACGTAGTTC
ACAGCTTTTATCTTCCACTCCGAGGGAACAGAGGATGTACCATGAGAGTTACCCTACAGTGACCTTCACC
CATATCAATGACTGTTTCTAGATCCCTCCATCTACTCAAGTGGGATACACACTGCCTCCTCCTAAGCTCC
GTGCCTCCACAGGGTGATTCAGGTAAATACTTGGAATCAGACCAGGCCAGGCCAGCAGTGACTGCCCTGA
GTAACTCAGCACAGATAAGGACAGTGCAGAGAACTCTGGAAAATATAGAGAATGTTACCTGGCCTGGGAA
AGCAGCAGGCAGAAGCCCAAGCCACAGCCTTGCAGATGAGGTTGTAGCCAATCAGAAAAAGGATTTTAAG
CTGACACGTGTTGCCAGTTTAAAAGTCTGTGTGAAGAGGAGGCCTGTGAGAAGAGGGTAATCCCCATGTC
TGAGGCAGCCTTCACTGCACAGCCCCAGAATAGGGCGGAGGGAACGTCCTCCTTAGGACAGGAGGAAGGG
GAAGGGTCAGAAGCCTCTGTTTATTCCTAGAGACCCTGTGGTGGGCCTGAGGGAACTGAGACATCTTTGG
AATCCCTTTCTGACTGTTCTTTGTTGCCCCACAACCCAGTTACTGGTCTGAGCTTGATGTTCCTATTTTA
TGCTTTTATCCCATCCTGGAACCCTCCTTATTCTGGAGCTACCCTGGTCCTGAGTGTGCTAGAGCAGGTG
GAAGCCCAGTGACAGATTTCCCAGTCTCAGGTGCCCTGGGACTATGCACCTAATTCAGAATATTGAACTA
ACTCATTGCCTTCATGTGCAACTAAGGGAAACTTTTTGTCGTTGCCTCTTCCCTATGTCTTTTTACCTGG
TAAAATCTTTATATTCTGCTTTTAGATCATCTCCTCTCTTCTAAAAGACGAACTAAAATTCAGGAAAGAC
AGAGAAGGAGAGAGAAATTAGAGGGAGAGACACTTATTTTCTTGTTCATTAATATATGCCAGATACTGTT
CTAATTGCTCTATAACCATTTTATACTGTCCCTAGACATGGGCAAGAGAAGTCTGATAAATAAGACGTTT
ATTTAATAAAGCACATTGATACTTCTACCATTGAGATTTGATTCTTAGGGTTGGTACAGCTCTCTAAACA
TCAATAGAATTCAAACCCCAGGAAATGCTTCTTCACAATTTTGCTCTCTGTCCTCAACACTGAAGTTGAC
AGTATCTTAAAGCTTTTAATGTTAGTGGACTATGCCTTTGCACAATCAGACTGTTCAGGAAGAATTTGAG
TGGATGAAGATATTTTAAGAGAAGAGTCAACTTAACTTTCCAAATAGTTTAACTCAAAAAGCATAAATGC
AATAGATTCTTTTCTTTTTTATAAGTGTGTTAGTTGCTCAGTCATGTCTGACTCTTTGAGACCCCATGGA
CTGTAGCCCATCAAGCTCCACTGTTTATAGAATTCTCCAGGCTAGAATACTGGAGTGGGTAGCCATTCTT
TTCTCCAGGGGATCTTCCCAACCCAGGGATCAAACTCAGATCTCCTGCATTGCAGGTGGATTCTTTCTCA
TCCGAGCTACCAAGGAAGCCCTAGATTCTTATGTAATGAAAAATCACTTGCCAGCAGGGCTGGCTGTCAA
TTTTTTAAAGTTTTGGCTGTGCCCCATGGCATGCAGGATCCTAGCTTCCCAGAGATTGAGCCCACACTTT
CTGCAGTACAGGGCAGAGTTTTAATCACTGGACTGCCAGGGAGGTTCTGATTGTCAATTTTTTTTTTTGC
TTGTTTTCTTGTTCCACATATTAACCTGATATTGCCAACAGTTACACATGGCAGCTGAGGAACATTCTGT
AATATTAAACAAGTCATATTACCCTTAAGTTTGTATATATGAAGGTCTAGATATAAAATGCATGAAACAT
GATTCACATTTTATAGAGAAGTCTTAGGCAAAAAGGTTCAGTATTTTGTTGACTCTCATAAAGCGCATCA
ATGGTAGAACTACAGCTCAAAACCATTTCCTTCAAGAACTCTGTTCTTTCCACCCACAACTTAAGGGTCT
CTAAGACAATAAAGTAAACATCTCTGTGACAATAATTTTCCCTCTCTGAAATATGGTCCCCACTTATTTT
CCCTTGTGGCTCAAACAGCAAAGAATAATCCTCTTGCAATGCAGGAGACCTGGGTTTGATTCCTGGGTCA
TGAGGATCCTCTGGAGAAGGGAATGGCAAACCATTTCAGTATTCTTGCCTGGAGAATTCCATAGACAGAG
GATCCTGGAGGGCTACAGTCGATGGGGTCGCAAAGAGTGGAACACAACTCATTAATTAACACTTTCACTT
TTATTTTACCATATCCCCAATTCCAAGAACAATAGGTTTTAAATAAATATCACAGAAATATCTAATCTTG
AATCATTTTTTTTCATTATTTAAACTCGTAAGGCATTCAATATTCAGATATTTTATAACTGAGAGAACAT
TTTCATCTCAATCCAGTGTAATTTGATTAGGGCAAAGTACCAGGCATTAGATTAAGAACCTTCAAAAAAA
ATGTCTACCCAGAAACAGATGAAGTTTTTCCGATCCACTGCTGATTGGTCCCTTTTCTAGGGACTCTCCA
ATCCTGCCATACATGGAAGCTCTCATCGGCTTTTATTCTGTGAAGTGGCCTCACCAGATCCACTGTGTTT
GAGCTGTGTTGACTACCATTACTTCTTCCTTTGTTCTCAATTATGTCTGGGATGGTGGCTCTGCGGATCC
CCAGAGGCCTCTGGACAGCAGTTGTGATGGTGACCCTGGTGATGCTGAGCACCCCAGGGGCTGAGGGCAG
AGACTCGCCAAGTAAGTGCAGGGCAGCTGCTCCCTGGAGCCACCACTCTGGGGAGCAGGCTCTGAGGGAC
CCTTGGGCTGGGGTGTGATCTTAGGATACTGTCTTTTATCACACATTTTCTCCCATTGGGAATGAGGGCT
ATGTTACATTC

>ENSBTAG00000013919|hg19

TGATGAAAGTGAAAGAGGAGAGTGAAAAAATTGGCTTAAAGCTCAACATTCAGAAAACTAAGATCATGGC
ATCTGGTCCCATCACTTCATGGCAGATGGGGAAACAATGGAAACAGTGACAGACTTTATTTTGGGGGCT
CCAAAATCACTGCAGATGGTGATTGCAGCCATGAAATTAAATGACACTTGCTCCTTGGAAGAAAAGTTAT
AACAAACCTAGACAGCATATTAAAAAGCAGAAACATACTTTGCCAACAAAGATCTGTCTAGTCAAAGCTA
TGGTTTTTCCAGTAGTCATGTACAGATGTGACAGTTAGTCTATAAAGAAAGCTAAGTGCCTAAGAATTGA
TGCTTTTGAACTATGGTGTTGGAGAAGACGCTTGAGAGTCCCTTGGACTGCAAAGAGATCCAACCAGCTC
ATCCTAAAGGAAATCAGTCCTGAATATTCTTTAGAAGGACGGATGCTGAAGCTGAAACTCCAATCCTTTG
GCCACCTGATGCGAAGAACTGACTCATTAGAAAAGACCCTGATGTTGGGAAAGATTGAAGACAGGAGGAG
AAGAGGATGACAGAAGATGAGATGGTTGGATGGCATCACCGACTCAATGGACATGAGTTTGGGTAAACTG
TGGGAGTTGGCAATAGACAGGGAGGCCTGGCATGCTGCAGTCCATGGGGTTGCAGAGTTGGACACGACTG
AGCGACTGAACTGAACTGAACCAAAGGGCTAACTGTAAATCTTTCCAGGGACCCCAGAAGGCAGGGGCTG
TCTTTGTGCTCTTCCTGTGCCACCCCAGAGCACCAGTGTCTCTCAGAGAGACATTCTTACAAATATCTGG
TTTCTCATCTGCTTCCTTGTTTATTTTTTGTGGCTGCCATTCAGGGATACCTTTTGATCTCTTGACTCTG
GTGGCCAAGGGGGCTTGAATCCCTGTACACATTCCCAAGACAGTTTATATTTAGGCTCAACAGGTGATAT
TATGACACTGATTTGCCAGAGGTGAGGTGTGCTAAAGATTCATACATGCCACAAGTTACTCCCAACATTT
CAAGGCCTCCTTAAACATTCCTGAAACTATCTCATTTCAGTTCAGTTCAGTTGCTCAGTCATGTCTGATT
CTTTGTGACCCCATGGACTGCAGCACGCCAGGCCTCCCTGTCCAACACCAACTTCTGGGGCTTGCTCAGA
CTCATGTCCATTGAGTTGGTGATGCCATCCAACCATCTCATCCTCTGTCATCCCCTTCTCCTCCTGCCTT
CAATCTTTCCTAGCATCAGAGTCTTTTCCAATGAGTTGGTTCTTTGTATCAGCTGGCCAAATATTGGAAC
TTCAACTTCAGCATCAGTCCTTCCAGTGAATATTCAGGACTGATTTCCTTTAGGATTGACCGGTTGGATG
TCCTTGCTGTCCAAGGGACTCTCAAGAGTCTTCTCTAAACTATCTAGAGTTTTACAATTCACTTAACACT
TTGAATCGCTATCTTAATTAATAGAAAGGGGCAAACAGAGATACCCTTAGAATCCTATCTAGGTATACAC
TCCAGTGAATTGTTCATACAGGAACATAACAAGAAATTGTTTTGGATGGTGTGAAGTCACATAGCTATAA
GATCATATAATAGATGAAAAGAACACTGTCAAGACTAGAGATATTGTAATTAAGAAAAGCAGTAGCATTG
AAGGAATTGTAGACAGCTAAACACTCCTGGTGTTTCATTCCTGTTCTGAAAGGAAAATAAACCAACTGCT
ATGTATTTTGGTTGGATGGCAGAAACAATCTGGTAGGACTGATTATTGTCATGCTTTTGTGTTTCCCATA
GCTTAGAATTTTTATTCAGTAAAAAGCAACAAGTATTTGTCACTCCTGGAGAAGCTTAATATTTTGAATA
GTCATTATGTTATGTTCTGCTATGATTATTAAAAAGCATTTTTCTCTTGTTATTGATGTAGGCATGGTTT
AGATGGTTTTTTAAATTAACTGGCCACTTCATTCTTATTTTAACAGTTTACTGAACTCCTCTCTGAAAGT
ATTTTTCAAGGTCAGATTCCAAATCCTGAACTTCAGAGACTGACAAGCATTTCTGCACCTGGATCACCTT
TAGCATCTCTGGATGGGTATTAAATAGCACCAAGTTTTCCTCCTTTACCTTGCTTGTGCAACATATGGAA
TGAACATATGACTTAAAAGTGGATGCTAGCAATAATTACTGAGGGTCATTTGCTTCAGTGTTCTCAAAAT
TGCTCTACAGTATTGAAAAATTGTACACCACGTAGGAAGAAATGTCCTTTCCCACTGAGGGAGAATCATT
ACTAATAAGCTTACTTTGTGAGGGTACAAACTTGTTTATAAGGATGGAGTACTAGCGAAATAACTGCTGA
AATATTTTATTGTGTATTGTGAGTAAAACAGACAACATTGCAGACATACACCTGAAATCACTTGGGAATT
TGCTAGTGGAAAATCTAAGTGGAATTTTGAAAAATAATTAGACCAGTATTTGTACTTTTAGTAAAGAGGC
GGATAGTTTGACACAGCACAGTTAGATGAATATTCTGGCGATGCCACTAAACTCAGTGACAGCATCAGGA
ACAAATGTGAAAGATCTTTTCATTCAGGTTAATTATTGACTAACTTTAAATTTCCTTTTGTTTATCAGAG
ACACCCCTACCTGTTAGGAAGTACAGACTATATAGTCTCCAATCAACAATGATAATTCTGTAACATCATC
TGTTACCAGAGATACTGTTTCTTGAGGTGTTCACAGAATCATAGCACATGAACTACTGAAACTCCTGGAA
GACTGAAGTGACTTCTTTTCAATTATGAGAGTTTAAGTAATTTTCCAAAATCTGCACCACTAAGTTATGG
TGCAAGTAGGACAAGATTTAAGATCTTTGCTTGCAATCATTTTCTCTCCACTCCCTTATCTCCTAACACA
CTATGATACATTTGTCAAAACAGCTCTGTTTCCAAGTGAGAGGAATGAATGCTCCTAAAGAAACAAGTGT
GTGACCATCATTGACCAGCACAAAGCTAGATCACAATAGGGGCCCAGTTAAAATATTTCAGATGCAACAG
GGTCAAATCTTTAAAGTACTAAATTAAAACAATCCTTTTAGTAGAGAAAATTCTACGTTTCAGAAGAGGA
CCTTCATATTGAATCTCTGACCAGCAACTGATGATGCTATTTGCCTCTGATGTTGATTGGTTGCCTAGAC
TGGGCTCACCCAATCCAGGAGCAAATGAGTTCCTTGGTCGGTATTATTTGGGATGTGGGTGGAGGAGGGT
CACAGTACCCA

>ENSBTAG00000038397|hg19
AGATGTCAAGACAAGGGGAGTAGCTCCTTTTCCTACCTGGTGAGAATGACTAGTAAATTTGTGATTTGAA
GGTATAAACTTGTTTGTCTATGGTAACGAACTAATGGCAAAAGAACTGCTAAAATAATTCATTATTTATT
TTGAAATAGACAACATTTCAGATGTACACCTGGGATCACTGGGCAGTTGCTAGTGGAAAACCAAGGTGGA

GTTTTGAAAAATTATGTGGCCAGTAATTATAATGGATAAAGAGGTGTATATTTTGCTCTAGCACAATGAA
CACAAGATCTAGACCAAAGAGAGAGAAAAAAAAAAACAAAAAACCGTCTTTCTACATATGACATTAATGGA
CAAGTCTATGAGAATTCTAGCTGATCTCTCTGTGACTACTTAATAGAAAATGTTTTGGTTCTACAGCTTC
CCTGGTGGCTCAGACAGTAAAGAATCCACTTGCAATGTGGGAGACCTGGGTTCGATCCCTGGGTTGGGAA
GATCCCTTGGAGAAGGGAATGGTTACCCACTCCAGCATTCTGGCCTAGAGAATTCTGTGGACAGAGGAGC
CTAGAAGGTTATAGTCCATGAGGTCACAAAGAGTCAGACACAACTGAGCGACTTTCACTTCACTTCACTC
ACGATCCAATATTTGAACTTGTGTTATTTGGAGTCACATATATCTTGGTTCAGTCTGAAATGTGCCATTT
TCTACTCCTGTATCCTTGCAAAGGTTATTTAACCTCCAACCATAATTTTACTCTTTTGTAGGATTAGAAA
GAGTACTAAAATAACTGAAATGATGTGAGAATTATATTTCAGAAAGATGTATAAAAAGGTCCTTGTATAA
TTCTGGGTACAAGGTAAAGAGAGCAAAAATTGCTCAATTAATTATAGTCATTTTTATTGCCAAATGGTGA
GTACTATCACTAGAACTATTAATGCACATAAAGGATAAATGCTTATCAGAACTGGGTTGGAGTGTAAATT
AATGCTGAACTTTAGACAGATAGTTGGCCTCTAAAGTCCATTCTAAATCTGAGATATTACATTTATTCTA
AAATAATGTGCAAGAATTATCTAATATAGATTTATTATTTTTTTTTTAAATTTTATTTTATTTTTAAACT
TTACATAATTGTATTAGTTTTGCCAAATATCAAAATGAATCCATCACAGGTATACATGTGCTCCCCTTCC
TGAACCTTCCTCCCTCCTCCCTCCCCATACCATCCCTCTGGGTCGTCCCAGTGCACTAGCCCCAAGCATC
CAGTATCGTGCATTGAACCTGGACTGGCATCTCGTTTCATACATGATATTTTACATGTTTCAATGCCATT
CTCCCAAATCTTCCCACCCTCTCCCTCTCCCACAGAGTCCATAAGACTGTTCCATACATCAGTGTCTCTT
TTGCTGTCTTGTACACAGGGTTATTGTTATTATCTTTCTAAATTCCATATATATGCGTTAGTATACTGTA
TTGGTGTTTTTCCTTCTGGCTTACTTCACTCTGTATAATAGGCTCCAGTTTCATCCACCTCATTAGAACT
CATTCAAATGAATTCTTTTTAATGGCTGAGTAATACTCCATTGTGTATATGTACCACAGCTTTCTTATCC
ATTCATCTGCTGATGGACATCTAGGTTGCTTCCATGTCCTGGCTATTATAAACAGTGCTGTGATGAACAT
TGGGGTACACGTGTCTCTTTCCCTTCTGGTTTCCTCAGTATGTATGCCCAGCAGTGGGATTGCTGGATCA
TAAGGCAGTTCTATTTCCAGTTTTTTAAGGAATCTCCACACTGTTCTCCATAGTGGCTGTACTAGATTTA
TATGTTTAAAATTATGTGATTATATCAGAAGATATAATTCATAGTTTTCCCATAGTGTTCTCTTTCCTAA
ACAGTCTGTAATGTTTTCATTAGCAAAGTCTTCTTTTCTTAGGATAAAGCAAGGGAAGTTGATAAATAAG
GGACATATCCCCAGTAAAATGAGCAAATCCTAATGGGAAACAGGAATAAGGAACTCAACACTTTTTTAGG
TAAGATATTACATTTTAAAGCTGAGTTATTGTGCCAATGGCTGACTTGAAGTCCTTTATCCATCTCTCAG
TCTCTTGCATACTATGATGTGGGCAGTTGGTTTTATTACCTTGTTAGATAACATGCTATAAAAAACACCT
TTAAGACCCTCCATTCTACATAGGTCACATCATATAAGTCCCCCTTTCTCTCTGTGTTTTCTGGAGGTGC
CACTAAAGTCAATCATATTATCAGGGACAAACCTGAAGACATTTTCATTTACATTAAATTATTGACTAAT
ATTTAATTTCCTGTAGTTTATCAGAGACAATCTTATCTGTAAGGAGGTACAGTCTATACTCTCCATACAA
CAGTTATAATTCTGTTACTAGGGACACTGTTCTTTCAGTTGTCCACAGAATTACAGCATTTTTCAGAATT
GAAATACTTGAAAAATCAAAGTGCTTTGTTTTCAACAAGTTCAGTAATTTCCCCAAATCTACAACACTAA
GTTATGATGCACAAAGGATGAGATCATAGGACTCTTAAAATGCAACAATTTTCTCTCCACTTCCTCAATT
CCTCATACACTCACACCTACATTTGTGAAAGTATCTCAAGTAAGAGGAAAGATTTCCTAAAAGGCTCAAA
CTAGTGACACATCAGTGACCAGCACAGACTGGTGCCCAAGAGGGGCCCAATTAAAATATCTCAGATACAA
CTGGACCAAACCTTTAAAGTACTAAGTTAAAATAATCTTTTTAATGGAGAGAATTCTAAGTTTCAGAAGA
GGACCTTCCTACTGAATCTCTGACCAGCAAATGATGATGCTATCCACCTCTGATGCTGATTGGTTTTTCT
AACACTGAGATCACCCAATCCAGGAAGAAAGAGTTCATTGGTTAGTATTGTTTGGCCACGTGGGTTGAAG
AGGGTCCTAGTCCTCACTGAGTGACTCTCAGCTGCTCCCTCACGCTCTGTGTTCTCCTGTATGATGTGTC
TGTGGTTCACTGGATGGCAGCTTTGGCAGTGCTACTGATGGTGCTCAGCCTTCCCTTCTCTTGGGCCAGG
GAAACCCAACGTAAGTGCACACCTTTGAGGGGAGGGTGAGCTATTATGGGTGGGAGAAGAAAGGGAGTTA
CCATTGTCTCTATGTCCAGACTACATCTCTTAAAAAACTGTGACATTCTCTTCAGTCATCACCAGTCCTT
ATCACATGGAGCCTTCGTTCCTTCCACAAGAAAAGGGCCTTGGATGCCTGCCTTCTGAATGCTGCTTAAG
GTAACTCCATA

>ENSBTAG00000015565|hg19
AAACAAGTTCTGGCACAAAGTACTTTTGTTTTGTTGTTTTAAGAAAAATATGGTTTTCTCAATTTGGAAA
TGCAGAAAAGGTAGAAGTGCATTGAGATCATGGTTTTAATCTCCTTGAAACTATTCAGAAGCTTCCCATT
ACATTTAGCATAAATTCAAATTTCTAACATCACCTACAAGCTTTTAAGCACTCGGTTCCTGTGATTTTTC
TAGTGGAATCTTTCAGTCTCCATCCTCAATCTCTGAGCTTCAGTGAACTGCTTTTATTTTTATTTTTTCT
GGTTTCATAGAGAAGTACATCTCTTTTAAGCTTCTACACATGCTATTACCTTTACCTAAAATATGCCTCC
CTTTGCCCCCAGACATACACACACCTCCTCTTCCTCTAGCTCATATCAACATAACCTTTTAGCTCACATC

TTAAATGTCATTCTTCAGGAACTTCTGACAATCTAACGTAAGTATTCTTTGTTTTAGAACTCCACACTTT
TCTTCTGACATTTCTGATTCCCGTGTAAAATTAATCCTCTTTTGGTTGGTTTTGTCCATCACTTTCATTA
AATTTTATGCTCTTAGAAGGTAGGAGCTTCCTTGGTTGCTCAGGGGTAAAGAATTGCCTGCCAATCTTTC
TGAAACGTCTTTTAGGTCTAACTTTATTCATTTTTTGGTTTTCTTAGGATGTATTATTTTTACATGGTTG
AAATGATTTTGAAATCACCTTCCAAAATTATAATTTTTTTTACTTATGGCAGAAAGTGAGTAAGTAAAGA
GCGTCTTGATAAAAGTGAAAGAGAAGAGTGAAAAAGTTGGCTTAAAACTCAACATTCAGAAAACTAAGAT
CATGGCATCCAGTCCCATCACTTCATGGTGAATAATTGGGGAAACAGTGAGAGATGTTATTTTTGGGGGG
CTCCAAAATCACTGCAGATGGTGACTGCAGCCATGAAATTAAAAGATGCTTGTTCCTTGGAAGAAAAGCC
CTGACCAACCTAGACAGCAAATTAAAAATCAGAGACATTACTTTGCAGAAAAAGGGCTGTCTAGTCAAAG
CTATGGTTTTTCTAGTAGTCATGTATGGATGTGAGAGTTGGACTGTAAAGAAAACTGAGCTCCAAAGAAT
TGATGCTTTTGAACTGTGGTGTTGGAGAAGACTCTTGAGCATCCCTTGGACAGGAAGGAGATCCAATCAG
TCCATCCTAAAGGAAATCAGTCCTGAATATTCATTGGAAGAACTGATGTTGAAGCTGAAACTCCAATCCT
TTGGACACCTGATGCGAAGAGCTGACTCATTTGAAAAGACCCGAATGTTGGGAAAGATTGAAGACAGGAG
GATAAGGGAACAACAGAGAATGAGATGGTTGGATGGCATCACCCACTCGATGGACATGAGTTTGAGCAAG
CTTTGGGAATGCTTCCCTGGTGGCTCAGAGGATAAAGCATCTGCCTGCAATGCAGGAGACCTGGGTATCC
CTGGGTTGGGAAGATGCCCTGGAGAAGGAAATGGCAACCCACTCCAGTATTCTTGCCTGGAGAATCCCAT
GGACAGAGGAGCCTGGCAGGCTACAGTCCACAGGGTTGCAAAGAGTTGGAAATGACTGAGCGACTTCAGT
TTCTTCTTTTCTTAGGGAGGCCTGGCATGCTGCAGTCCATGGAGTCACAAAGAGTCAGACACAACTGAGT
GACTGAACTGACTGACTTAACATACTGATGTTACTATTTTTATAATGCTAGTTGTATTTAAGTAGTTTAA
TGTTACTTCTTTTTATAGTGGGAACATTTTACCTCACATTTCCCTTTGTTAGATCTTTTTCAGTATTGCC
AAATAGTTGACATTTTATATCATTTTGTTTTTATTTTATGTCTTTAATTTTTAAGGTTCCTGAATATTTT
CACATATTTATTGGCAAACATCTATTACAACTTTAAGAATTATCTAAAGTTTATTATCTCTTAGATCTTT
GTGTTTTTCTCAGTGGCTTTATGGATTCTCTGTGCATTATAATAAACACTTTTCTGGTATTCGATTCAGT
ATTTTATCTTGTTTATAGTTTATATTTTGATTCTGTTTGTTATTTTCCATGTTAGAAATTTAAACATTTA
TATTTAATAAAATTACTGCAAAATTGTTATTGAAAATTTTCTGTCAGTCTTACATTTGACATTTTGTGTT
ATTATAAAAATGCTTTTCCAACAGTTTTCACCTTAAAAAAAAATAATTGTATTTACTGTCGCTGGTGGTG
TGGGGGTCATCTTGGGCACTAAGCACACATATTAATGAATCTCGGACTCTACCCTTAGCTCAAATGTCAG
CTTAAAAAAAGAGAGACTGTTAATAAAATGATAAGTTCAGTAAAAAGACTGCTTATTTTGCAAAGGGGGC
CTCGTTGGTGGCTTATACAGCAAAGAATGTGCCTGCAATGCAGGGGACCCAGGTTCAAACTCTGGGTTGG
GAAGATCCCCTGGAGAAGCGAATGGCTACCCTCTCCAGTATTCTTGTCTGGAGAATTCCATGGACAGATC
TGCTGTCCGTTGGGTTGCAAAGAGTTGGACACAACTGAGTGACTAACACTTTTACTTTCACTTAATTTTG
CAAAGGAGAGAACTTGATTAACTGAGTGTAATTGCAGGTGGCTTTTTTTTTTTTTTAAATAAATGACTAT
TTGCTCCTTTCCTTCTCCTTCAGAGCTCAGGCTTTTCTTGGGACTCAACTCATAAAGCTTCAGAATCACC
TTTGTCTATTCCTGTTTCCCAGAGTTAGATCACTTTCTTGAGTGTTCTAATGCTAATGAGTGTAGCATTG
TTAGGAATTTTTTTTCTCCATCTTCCCATCTACCTCAAGCTGTTCCCCAATTATCCACACTGCTTTGAAT
CACGTTTTCTCTCCAAGTAAAAGAAGATAGCAAATATCTAAAGTATTTTGAAAACTAAGTTCCAGCCATT
GTTTTAGGTGCATTAATCCTCACACAGCCCTAATACACATATTAGTATTATTATCCCATTTATAGTTGGG
CAAAGCAGTGCAAGTATTTGAACTAAGGCACTCTAGCTCCAGAAATCATGCATTAAGTAATAAGTTATTG
TTAAATGAAATATTTCCTGCCACATGAGTGGGCAAGAAATGTCACAGCTATCTGCAATTCAGGCCCTTCA
AATGTGGATTTCTGAGCCACGCTGACAAGCAGTGCCACTCTCTACACAAGGAACTTAAGGATGTCACAGT
CATCAGCGATTTCAGCTCTCCAGTGTGAGTGGGAAGGCTCAAAGGGAATTTAAGAAACAAAACTATCTTT
GTAAATATGCA

>BTNL2|hg19
TCTATCAGTGCTTTAAGTCAGAAAACAAACCAAGCAGACAGTAGAATGACAGACAGACAGTTCAGCCACC
AGGATATGAGAGTTGACTTTTTTTTTTTTTTCCTTTTGCTGGAGAGAACTCTACATTTTAAGACCCATTG
CTAAGGGATTCCATGAAATAATTATTATCTTTCTGGCGAGTTCACCTGACAACTCTCGACACAGATTTCT
TACCAGGTCCCCTGAATAGAAACTGGCACTGATCACCTGAATTTCTCTGAATTCATAGCAAGACTGATGT
CCTCTCACAATAATCCTTTTCCTTTTTCCTGATTTTCATTTATCTCCTTTATTGCTTTAGTCGCCCTTCT
TTCCAAGACTCCATACTCCCAGTAATGAAGCCTAAGACCTAAATAGGAATTTTTTTTCACAGTTATATTC
TCATCCTACACTTTTAGGATTATTTTAATGTGAGTAGTGTATAACTAAAGAAGCTTGCCCTACTTTATAG
CAAACCTAGAATTTAAATCTCAGGCATCCTAAGCTCAGGGTTCCTTTTGTTATCTACTAGCTGAGAAAGT
CATGAATGTGTATCATACAAGACAGCAGCATCTGGGGGAGAAAGGGTCTGGAGTGAGTTAGATTTTGAAC

AGAAGCCCTTGGAGGCGGACAGTGAACAGGTTTCTGCTGCCCTCTCGTGGTCATATTCATGAATTACACC
ATTTCCTTGTATTCTTCCGCCTTCTCTGAATCCTCACTTTTCCTGCTTTCCTTAGTGTTATGAGCAGTTT
GTCATGCCCTTCTCTTTCAAATACCAGCATGTCCCTACACAAAATGAACAACATAATCACAGCATAATTG
ACGGCACAGTTCTTATTTCAAAACTGGTGTCCCCTTGAGAAAGCATACGTAAGGGTTTTTTGTGAAGAGA
TGCATACAATTTTAAGCAGAGTTTATTTTAAGTGGTCAGTGGGTACTAATGTGTCTGATCTCTAACACAA
TGCAAAGACAAGTCCTAAAGTCCTGGGAAAGTCCCAGCACTTTACATACCATCAAGATGATACAGAATGT
GTGTAACAAATAGCTTTTGATACCGCAAAATTGAGTGTGAAAAAATAAAGTTCTGTTTTCCTATTACCGA
TCCGAATTTTTGTGTCAAGCCCTATCATGTCTTAGAGCAAAGGGGTCAATCTAGTAATTTCCAGAATACT
CCATGACTTTCTTGACCATTAATAATTTAAAAGCAGCCCTCTCTGCCCAGGCTTATTTCTAAGTCCCCAG
GCAGGAGGTGGTGCAGGACTGCTGGAGAAGGCAGTCCTCATATTTACATGATGACTCCAACTGTATGTCA
TGGTCACACTGCCCCTGGTTGTCCCTGTCCTTCCCGAATGGGTGACGATGACTGTTATATCCATACCATC
ACTCTCCTCTTCCTGTATCATCCATGCCAACATTCGAGGCAGTGATCTTTCGTTCTAAGAGTTGAATCTT
CCAGGGTCCACCTCTTCTTGCTTCGAGTAGCTTTCCCTTTGCTGCTGCTCTTTGGGGCTGTTGTTGCAGA
GACACGCCTATGGCATCACTGCCATTCTGGGAACCAGAGGTAAAGTGGGGCAGCGTCCCCTCCACCCTCA
TCGAGAGAGAGAAATGGGGTAAAGGAGTTTTCACCAAGGTTTAATTACTTTTTTTCTCCTCTTCAAACCC
ATCATTCACCATGAGAAAGGTCTTTATCTGCACTAAAGATTGCAGGGGAAAAATTCTTCATTAAGAATAG
CAATCTCAAGTTAGCTAGCCCAAATGTGGGCAGACCTTATCAGGGGAGAGGGAGAAACTTCAGGAAAAAA
ATTCGTTTAAATGCTTTCACATAGTAATGTTGCATATGCTTACATTTTATATTGAAATGAATATTTACAG
TTGAATTGTCTTGGAGAACTTTTTAATTCTTGAGAAGCCATACCTCAGGGATACTGTGGGTGTGGTTCCA
GACCACCACAATAAAGCGAATATGGCAACAAAGTGAGTCACACAAAGTTTTTGGCTTCCCAGAACTTATA
AAAATTAACATTTATATTCTGCTGTAGTGTATTCAGTGTGCAATAGCATTATGTCTAAAAAGCTATATAT
CTTAATTAAAAATACTTTATTACTAAAAAATGCTAACTATCACCTGTGCCTTCTGTGAGTGGTAGGAGTA
CCATCAAGGCTCACTGACCACAGATCACCACCCAAATATAATAATAATTAAAAAGAAATACTGGAAGAAT
TGCCAAGCTGTGACATGGAGACATGAAGTGAGCAAACGCTGTTGGGAAAATGGCGCCATTAGACTTGTTT
GGCTTATGGTTGCCACAAACCTTCAATTAGTTTAAAAAATGCAACACCTGGGAAGCACGATTAAAGCAAG
TACGCAGATCCCAGCGATACTTGCCCTCTGATTCAAGGAATGGACTTAAGGGGAACTTAAAGGAAAATCT
AAGTTAAACCAATGTGCGCGTACACGTGCGCGCACGCACACACACACAATGAATGTGATATAGTTCCCTC
ATTATAATTTTTGTGCCTAAACATAGTATTGAAAAGCTTTTTAAATTTCTTTGGTTCAGTGTCTGTAACA
ATTAAGCTCTTTATTACCCATCAAAGGGAGATAAAGAACCAACGTTAAAAGAGCAATAAACTAGCAGGGA
GAGACCTTGGCCTGGCGCTCTGTTCCTGGAGAAGGTAGCTCCTGTCTCTGGGCCTCGGGCTTCTCATCAT
TAGAAGAGGGGAAGGATACTTGATACTCAGTCTTGCGTCCAACTTTAAGCCCCGTGGAGACCTGACAATA
CGCGGGAGTCCTTAACGTGTGGGGAGGTAGTGGGGCTGTTTAGACAGGCGGCCAGCCTGAGGTGCTGTGA
GGATCTGCCAAGGCGCCCCAGGCCGGATGTGAGGTGGGGCCTGGCTTTCTGGGAAGAGATGAAAGTGTAA
ACGTTACCTTTTGGTCTGCAGGTCTGAAGAACTCATACTAAACCCAGTCAAAGTTCACTTGCCACCCGCG
TTTCACTTTTCAAAGGATTACAGCTAGGAGGTGAAGATGTGATTCTGACTGCAGTTTCATCTTCAGGGAT
TTGGCTTTTCCTTCAGTTTGGGGGAGGCGGCTTGACACCTCCTTCACCCACAGCCCAGGGGACCTGGGGG
GCCTCCCCCGCTTTGACTGTGCCTCCATTCTTGCTCCAGGCAGCACTTCAGGCTCTGACCTGTCGGTGGA
ACCTGCTCTGTGGACGAAGGGTACACCGGGGACCAGGTGAGGAGAAACCCCAGCAGCCCCCTGCCTTCAC
CCTGAGGGAAA

>ENSBTAG00000007618|hg19
TTTAGAAAATTCTAGTTTCTACTCAGGAAAGTGATGAAATAAAATTTATATTTTAAAGCAGGACAAGGAA
ATTTAAGCACAAAATTCTCTATTTGTCTGTTTGGGTCTCTTTCCTGCCCCTGTAATATGTACTGTGCTTC
TGCATTGTACATTAACAAGACCTCCTCAAAGGTGAGAATGCTGGCTTGACTATATGATAATTTTTTTCCT
TTCTTCTAATACTAGCAATGTAACTTCTTAAAAGAACAGCATTCTTTCCTGACTCTGTAAGGAATCGTGG
AGACTTGCTACTTGCTTTGTACATGCTTACCTGGACTAGCTCTGGTGAACTTTCATGTAAGATGTCAGTA
TGTCATTTTGATGACAATATTTTGTCTTAAAAATGTGTGCGGTGGTGCCTCCAATTAAAATAAATAAATT
TATATTAAAAAAAAAGCACATGGGGGAAAAAAAAATGTGTACGGTGTACTTTCAACTTCTAACAGGTGGG
AGAGTTCTCAGAGCTTCTGAATCTGTCTCTGAATTATAATCCTCAGTTGACTTGAATGAAATTCTCCCTT
TGTTTTTTTCACTTGATTGTTAATTGAATTTCTGTTGACAAAAATGAAGACTCCATTCAACTGTTTTCTA
GTAATTACTATTGAATGACATGTATGATGGAATCTGATTTTTTTTTTTCTTTTGTACATAACAAATTTTTC
TGCACCCTGGAACTTTGAGGTTTTGTGTTTTGTTTAGTTGTTTGTTTGTAACTCTAAAACATTACAAGAA
TTCATGTATAAATGACTCTTCTTCTCTAGTAGTCAGCTTTTTGTGAAGTACCCTTCCTTCTGATTCTTGC

CCCTTATCTTTTCATCTTGGGAAACATTCTGGGTGAATGCTGAAGTTCTGGGTGGATTTGCTCAACCTAA
ATTTCCTAATTTTTAAATCTTTTTGATTTTAAGTACCACATAATAACTCCCTTTTTCTAACCCAATATTT
TGATCTTCAGCAGTGTCTACGGGGTCACACAGAGTCAGACAAGACTGAAGCGACTTAGCAGCAGCAGCAG
CAGCAGCAGCAGTGTCTACTCAGAACTGACACCTCCATTTGTTTTCTATTTAAAGACAATTTTTCATTCC
CAAGAATGCTGATTGTTTCTCTAATACTTTATCTCCTATGAATGAATCTTCCTAATATGCTTCATATTTG
CATTAAAGATTTTTATATACACACATTATTCTTTAGCTTTTTAAGTTAGAGAAACCTGAAAATTAGAAAG
AACCAAAATTATAGACAGAAACACTTTTATTTGCTCCACTCAGAAATACATATATCTGAGTGGAAATATT
ATTATTTTGCTGTGATTTTTCTATTTTTTGCAAACAAGCAAGATCATACTGTTCACACTGTTTTAAAAAC
TTCTCTTTAAAATTAATAATGCCTTGTAGCTCTTTCCCTCTCTCTCCCTTTTCACCTTTCTCTCTTAAAC
ACACAAATGCTGGTATGTATTCAATGTAATTCCCTCTAAATATTCTTCACAGATTAATTGGCTCAAACAA
TTCTCTATAATTGGGAAAAAATGCCTAAATATGTATGTGAATTTTTAAAATTTACTTTTTTCTAACTGTTG
GAAATGTATATTAGTCTATCTACATTTTGAGATAGAATATATACACAAAGACGTATCATACATTTCAGAG
TAAACAAAAGAGGCAAATAATATTTTCAGATAGTTCCTTTTCTCCTTTAGTTTTCCCTTCCCGTTCTTTA
GTGTTTCAGACCTAAACCATAAGGTAGGTATCAGACCCCAAATAAGACAGGATAGTTTTATGGAGGAGTG
CTGCTTAGAGTGGCGTGAAAGAATTTGAGTGGAAGGAGATGGGCCTGTATGAGGAAGGGAGACAGCCCCA
AAGGAAATTCAGAATATATGGTCCTATATTCAAACAGTGTAATAATATGTGGCAAGAAAAAATTTTAAAT
GCACTGATACACACAATGTGGTTGAACCTCAAAAAAACATTTTGTTTAGCCAAAAAACCAAGCACAAAAG
GGTGTATACTGTATGACTCCGTTTTGGTGAAGTTTCTAAGAGTAGGTAAGATTAAGCTATCACAATTTAC
ATCAGAATACTGTTTTTTATTTTCCCCTTTGGAAAGGATTGATGGACAGTGGACATGAGGAAAATTTCAA
CAGACAAAAATGTTCCATTGTTCAATGAAGTGGTAGTTACATGAGTGTAAGCATTTGTGAAACCTCATTG
AAATGCATGTTCTAAAGCCATGCATTCCATTGTAGGCAAATTATATCTCAGTACATTTGATTTCTTAAAA
AAGGCAAATGTACCTATCTCCAGAGAATATTTATTTAGGATTTTGGTATTCATTTGTCCAGAATTACATA
TTAATATTTACTCAATTGCCTCTTTTGTCCCTGATTATCTGTAAATCTTATGTGACATGAAAAGAACATG
AATTACACTTGGAAAGACTGTGAAGTGGGAAGAAAACTCTGTGTGAGCATTTTGTATCTGGCACTCTATT
ACTGTGTTTTTACATCTATTCTATGTTGGGGAACCCTAAGGTTTTTTTGGAAACAGAGCCCTAATATTTT
CAGAGCACAGAGTCCTCCATTCCTGATGTCTTATTGTCCCCCATGCAGTTCTGCACATGGCTTCCAATGT
CAGCTATGGTGTGGTAGGAGGAAGTGTGCTGCTATTGGGTTTATCCTCTCAATCATCTAAATTTGTGAAT
CAAGAATTTGGATGACCAACTGACTTGGATATTATACAGGAAGATGATTCTTTCTCCCCAGCTAATGCAA
TTTCTGTCCTTTGCAGGTGGGAGCAGGGAGCCATAATGAACACTGTCGATCAAAGCAACTTTAGAGTTTC
TATCATTCTATGGCTCAGCCCAGTCCAAAGTCCTCTGGGATGAACTATTACTTCCACTTTCAAAGTTAGT
AGCAGTGCTGGAGGCAGGCTGAGACTTTCTCATTAAACCCATTCCAGAAGAGGCATTCAAATACCCATTC
TTCTTGGCAGCATTATTTGTCCTCCTTACTAAGAATGTTCTCCTAATTGAGGAATGGGTTAGAAACCTGC
CGTTTTCATCGCCTGCAGCCACACCCTTGGATGCTGACCACGAGAACAACCAGGTGCACATTGTGTCTGG
AGCCAGATCTTGTAGGTGCACTAAGGAGACCCGGAGCTTGGTCCAGAAACCCCCCTTGGTTCAACCTGGAT
GGTGAGAAAAACATGTGGGGATGTGCAGGTGTGTCTGTGGGACATGTGGGTCCTGAGAAAAGAAGGGCTT
GAGCTCTGGGC

>ENSBTAG00000063006|hg19
TATTTTTTACTTTTATTGAGGTAAAATTCACCTAATGTCAAGTTAACCACTGAAGTGTGCAATTCAGTGG
TGTTTGATACATTCATGATACTGAGTAACTATCACTTCCATCTAGTTCCAAAATAATTTTATCACCCTAA
AAGTACACTTCAAACTCATTAAGAAGTCACTTCTCATTCCCCCACTTCTTAGTCTTGGCAGCCACTGGTC
TGCCTTCTGTCCCTGATCCCATATGCGTGGAATTATACTCTCTGCCACATTTTTTGTTTTTGCCTTTGTT
CACTTGCATAAAGTTTTTGAAGTTCATTTATGTTGTTGCATGTGTCAGTACTTCATTCCTTTTTAAGGCC
AAGTTCATTGTCTAGGTATACCACATTTTGTTTATCCATAAATTGTTCATGGACGTTTGGCCTTTTTATG
CTTGTTTGATTATTGTGAATGGTATTGCTTTCGATTGCTACATTTTTCTTTAAATGCCTGTTTTCAGTTG
TAGGGGTGTATACTTAGCAATTACTGGGTCATATGGCATACTTTGTTTAGTTTATTGAAAATCTGCCAAA
CTGTTTCCTATAAAGATGGCACCATTTTACATTACAATCAGCGATGTATTAGAGTTCCAGTGTGTCTTCT
ATCTCAATAACACTTGTTACTTTTTATTTTTGAAAATTATAGTCATAGTAGTGCCTGGAAAGTGGTATCT
CATTAGGGTTTTGATTTGCATCTACCAGTGGACAAAAGAAATTGAGTATCTTTTCATATGCTAGTTAGTC
AACTGTACATCTTCCTTGGAGAAATGTCTATTCAAGACCTTTGCTCATTTTTTTTTAAAAGACCTTCGCT
CATTTTAAAACTGGATTCTTTTTTTTTTTTTTTCCAATCACTGAGCAGTCAGAACTTTATTTTCTTTATAT
ATATATTTGATCCTAGGCCCTTAAAATATGTAATTTGCAAATATTTTCTCCCATTCTATACCTTGTCTCT
TCACTATTCAATCTTTTGATGAACAGAAGTTTTCAAAGTTTTAAAAAATCAACTCCAATTTATCTGTTGT

TCTTTCTTGTGTTCCTTTTGCATTGGTATCATTTCTAAGAATTCATTCTCAAACCCAAGGTTATGATAAT
GTAACTTTATGTTTTCTTTTTTTTATGGTTTTAAGCACTTGGCATTAAGATTTGTTTCATTTGGAGTACA
TAATATGAATGTAGTAAAATATAATATAGTAAATAAAAGAGTTAATAATAAAATATAGTAAATAAAAGAA
TTAATAGTTGGAGGTTTTTATGTTAAATGACTAATTTGATTTTCACTACTGTGGTTGAAATAGGAAATAT
GAAGTGTTTAACTTTTTGAGTGTCTGACAAACTCTTTACATAGTCTTGCTATGTATCACACAATATTACA
TAGTACTGCATGGTATTTGAGGGTGCTACTTTATCTAAGGCTTCATTCTTTTACTGAAAGTCTCTTTGTC
TAATAATAAAATACACAGGCAAGTGCACATCCCATTTTTTCTGTTTTGACACATATTAGGTTCAATTATG
GATCATTGGAGTAGGTTGGTTAAGAATAACTAACAGTTTTTATATGCTTATATCTTTTTATCAGAGAGAC
CTGTTATTAGAAGAATTATATTACAATGAGGGGCTTCCCTGATAGCTCAGTTGGTAAAGAATTCACCTGC
AATGCAGGAGACCCCAGTTCGATTCCTAGGTGGGGAAGATGCCCTGGAGAAGGGATAGGTTACCCACTCC
AGTATTCTTGGGCTTCTCTTATGGCTCAGCTGGTAAAGAATCCACCTGCACTGCTGGAGACCTGGGTTCA
ATCCCTGGATTGGGAAGATCCCCTGGAGAAGGGAAAGGCTACTGACTCCAGTATTCTGGCCTGGAGAATT
ACATGGACTGTATAGGCCATGGGGTCACAAAGAGTTGGACGCGACTGAGAGACTTCCACTCACTCATATT
ACAATGAAAGTCAAAGCAAACGATAACAAATAGAGAGGGACCAAAACAGTATTAAATTTTATAGAAATAT
GCAGACAAAACACGTGTATACCTGTGGCGGATTCATTTTGATATTTGGCAAAACTAATACAATTATGTAA
AGTTTAAAAATAAAATAAAATTAAAAAAATAAAAATAAAAAATAACACACACACACAAAATTTTATGGAA
AATAGATATTATTTGTATGTAGTAAATAGGATCATAAAACAGAAGAGGAAGTCCAAATCCCATAAAAACC
TTTAACAATTTCTTGACATATCAATGTCAAAGGTAATGAAGAATTAAAGTGCATTGTGGAGTTGAAAATA
GGGTGTATATGGAAACAAGATTTATGATTCTTTTCCAGTATGAATTTTTCTGCCATATAGTTTTAATAAT
CTAAGAACTTGGTCATCAAAGTAACAAAAGATAACTAGTACTTCACTTTTATGAAAAGGGAATTTCTCTA
AAGAAAAAATTTTCTTAAATGTTACTTAGATAGTTTTAAAAATAATGGCTAGCTCTACCTCTTATAATCC
TAGGGAGAGTTTCACCTTTTAAAAAAGCTATTTTCAGGGATGGAGGATGTCCAGTTTTCTTTTAAATGGC
ATTACTTTTAAAAGTGAATTCATTACTAAATCTCATCCATCATTTGACATACTCTTCAACAAGAAGATGA
AAGACTGCTTTTAAAAATGATGCTGAAATGCCCAGAGATAATACAGAAAACAGAACAGATATTTGTATTG
GGTAAAATAGTTATTTATGATGTCAGACAAATGTTGGATAAAGCTCTGACTCTGAAATAAGCAACTGTGG
GGAATTCAGCAAGTAATGTATCTTTGCTCTAACTTCACTTTACCGCTCTGCATATGTGTAATTACTTATC
TGGAGCACTTCTGATGACCAAATGTAATGCTACCTCTAAAACAAGTATCATAGAGTCTAGTACCGGGAAA
GTACTTCAGTAAAAGTCAATTGTCGTAACGGTGATGATGATGGTGATGATAACAGTATTGTTACCATATC
ATTAAAAGGGCTTTGGGAAATCATTTTGTGGGATTTTAAGTTCAGTCATAATAAATTAAAATTTATTTTT
CTACTTCATCATTTGGTAAAGAGCAATGTGACCCCTGTATATTTTACAGAACACCACCTAGAAAATAAAT
ATGTTTCTTCACTCTAAAGTGGGTAATCTATTATATATAAAACAGGTGAAGAGATTTTGGAAGGTTTTTG
TGTTTGCTTCCATTTCTTAGTGATTTTTATATTACTTCTTACTTTTCAGATCAACTTAGGAAAGAACTTG
GTAAACTTTAA

>ENSBTAG00000069649|hg19
TTTCCTGCAGCATGATATAATCCTTAAAATATTAACATCTTTTAATGACAGTTTTCAGTGACTTTCTTAT
TTCAAAGATAATTTAATTCAGATCTGTATCAAAATTATGTATGTTGTAGTCTTGACATGGAGGCTTACTT
GCTGGCTCAGTGGTAAAGAGTTTGCCTGCCAATGCAGGAGACGCAGGTCCAGTCCCTGGGTCAGGAAGAT
ACCCTGGAGAAGGAAACGGCAACCCACTCCAGTATTTTTGCCTGGGAAATCCCATGGACAGAGGAACCTG
TTACAACTGTCCATGGGGTCACAAATGAGTTGGACACAACTTAGTGATTAAATAACAATGAAGCTTCACT
TAACTTCTTCTACAAGTGGATTGTGGACAGATGAATATTGACAGAGTAATGACATTTGCCCTGCTCTTCA
ATATCCTTTTTCTGGTCTTTAATATCCTTTTCGAGTACCTCTCCTGTAGTTCAGTTTCTGTCTGTTTTCA
TTGAATTTTGGAAAAGGAGGTTAACCCAATGCAAGATCGATGAATGTAATGACATTTATTTCAGACAGAG
GGATGACCACCCCCCACTTTATGATGTCATTTTCATTCTTATTCATTTGGAAACCAGGCATTTATAAAAG
TACAAGGACATCATCCCCTAATTGGTGAGATGTCCTTTGAAACTCATGGTTAAATGCTCAGAAGTGTGTA
TCAGTTAAGAACACAAAGTAAACCAAGGCTGTGTCTGAAAATTTTGTCCTCTTCCCTTCCTGTCATGGTT
ACTTTATTTTGTGAATTGTTTATTAGTTCCTTTGACTTTGTTAGTAAAGGTTAGGTGAAAAAAGGCAAAT
TTCATTAATGTATTTCCCTGTGGATCTAGACATGGTTTCATCTGTTATTGTACCTTTGTCATCAGTGCTC
AATCTCCATCTTTTTCAAAGTCAATATGCACACATTCTTGTATGATTTTGTCTCACCTTTCTGTCTTTAT
AATGTATGTTTGCTATATGGACTATTTTTTTCAGTTTTCCCTATTTATAATTCTGTGATTTGTCTTGTTG
TTTCAAAATCTGCAAGCATTTACTTCATGTCCAGTTAATGGTAGTGATCACTTATCTGGGAATTATTGTC
ATTTTCAGTGTGGCTGTTGTGTAATGCATTAATCTAAGTCACTCTCATATCCTGTAATTTATAACTTGCT
TCACTGATATTGATTATTTTATGTTTCTTGCATCCTGAATTTAAATCAAATGTGTATCATTTAGGAGAGG

GTCAGAACAGAAACCACATAAGACAAGTAGGTATGTTCACTGGTGACATAAATGTGAATAGAGACACCCA
GTAAATTCTGGAAAAAATTTTTCCAATCCTGCAATTAAATATTTGTATCTAGTTTATTGACAAAAGCTAC
TGTGACCTCTCGACATGTGTGCTAACATCCAAATAACTTATATCTCACAAACTGAAAATGAAAATCTAAC
TAAAGTCAAAAGTGATTATAAGCTTTTTTAAAAGGTAAATTCCAAAAACAACATTCCTGAAATTATCTCT
CAGTGTTCTTAAATGACTCAAATTATACCACTCTCTATTTCTGGAATATGTTGAGCAAAAGAGAGGGTTT
GGTCACTGAGAATGTGTGAGCCTCTTTGTATATGTGTGTGTTTGTGTGTGCATGTGTATGAGTATATGCT
TATGCTCCTGAGTGTATATGCATTTTCCATATGTGTTTTCCTGTTTTGTGTGAGTGTGAATAATGGAAAA
TATATTTATAACTTCAAAAGAGTCCATAAGATTAATTTATCTTTGTTTCTTTTCATATTTGCCTGGACTC
TTCATTTTATTTTTTAGATGATCATGGAAAAGTATTTAAAAATTTTTTCTAACATCATTTGCCCCAACAC
CCTTTAAATGACTGCTAATAATTCCCTGCCTTTCTTACCATGTATCAATCCACCATGCTCATCTTTCCCC
TCTCCCATCTCCAACCTTTTGGATATGGACTACCCCCATATTAACTCCTTTTATCTCTTTTCCCTAATTC
CATCCCTTCTCATTACTTGCTTTTTTTTCATGTATCTTTTCTTCTCTCTAAACATTAGGTCTTTTTTCTT
TTCCTCCTTTTCTGTCTCCAGAGAATTAACTAGCTTTAGTATATTTGTAGAGATAAGTGATATCAGGTTG
ATCACTACATTCTTTCAATTCTAAATACATTTTCCTACAAGTGACCACAGATGTAGAATCTCTGTGACAT
TTAAAACTCAAGGGATAATAGCTATGTGACTAACTCTGAGCCAAAACTTAAGTGAGCAGATAACTCCATA
TTTGCCGAGCAAATTTTGTCTACCTTCTGCTCTTTAAAAGACTAGAGTTTCAGATGTCATTCCTTGGCCA
ATGTGAGCTCTAAGCCTAACCTCACATTCACACTGACCACATAACCTAATTGTTCTGGGTATAGATGTGT
TCTACCAAATAGTGTATGTTGTTTTTAAAAATTTCTAAATTATTAGGTCTGCAAACTGAACATTTTCTCA
TTGTGTTTATGATAATGTCACCTGTTAACAAAAGGTCACCTAATACCGTATCACTCAATAATGAAATGTT
ATGTTAATGAATTGAGAAGCAACTTTCCTTAGAAATTTAAAAGAGTTTTTCATAAAACATTAAGTCAAGC
CTAAAGTTTTGCCTCAGGTATCCACTTGATATTTTCATTTACGTTTTGTTTCAGGGGAAATAGAACTTTC
CAAACTATCTCTTTACAGTTGTAGGAGGCCAGAAGTGGTTCATACTACAATAACCATATGTATAGTAATG
TCTGATTTTATGTTAAGAAAAGCACATGTTTCAAAGGTAAATTTCATAAGTATCATTTCAATGCTTTTAC
CCCATTAAGACACAACTTTTCCTGCTTAACACTGTAATCTTCTGGAAGAATAGTCCCTTGCCAAAATGGC
TCTTCATCCATACTGTGTATTAGGTTTTGAGTTTTTTTTTTTGCATGCAGATCAAAATGAATTTAAATAT
GAATTTTGTAATACAGTTTCTTTCATAGTGTTCCATTCCCATGATACTGACCTTAAAATTTCACTTTATT
TTTCTATAGAAGTTTGTGATCAAGATGGTAAGTATTCTGAAACTTCTCAAAAACCTCTCTAACATGACTG
ATTTCTTGAGGCCCCATCTCTTAATCCAGATGCTTTCCATTGTGTTGTGCTTGTCCCGGATGCCTGACTT
TGTGTCTCCCAGTTGAATTATAGGCTTCCCTAGACAGTCTCTCTATTCAGTCCTTCATTCTATTTTCCAA
TCTGTATCCTT

>ENSBTAG00000057211|hg19
ATTATTATTTCTGCCAACTGCAGTGTCTTTTCAATAATTAAACAAGATATTGTATCTTAAGAAAGAATCA
TGTATATTTCAAAATTTAGAACTATGTATGTAAATGAAAAGGGAACTATTGTATCAGAATAATTCCAAAA
AATTTTTCACAAATCACTTCAAATTTCAAAGTTTAACATCCTCCTTTTTTCCTTGATATTTTTCAATTAC
ATCTTGGTTTACATACTGGATAAATTTTAGGGACATATCCCTTAAAGGCATTTGGCAGGCTTCAAATGAA
AATAGCCACAGACCAGGTTTCTAAAGAGTGGTACTTTAACTATGCACATTTATATCATACTTATAGTACT
ATTTCATCAATGTGAGTATGTGTGTCTTTTGTTATGAAAAAGGTGGAAAGCCCAATTCAAAACCAAATAT
TGATTCAGTTTATCCCAGAAGAATTTCCACTTTACCTTGATCTACTTTTATATGGTAATTCTTTGGCAGA
AAAGGGTGAGAGAACTTAAAACAGCATTACCTCTCATGAGTTACATGGCCTTTGAAATGTTAGGTTCAAA
TTCTTAGAAAAGTGTCTTCATTTATAGTGAATGAATAAAGAACGGAAAGGAATCAAATTATTGTCTTTTC
TTTCTCACATTGTGCCTTTGGTTTGCATGGTTATTTCTTTCAATTTTTAGATAAAGGTGAACAGGATAAA
TGTAACTCCCATTAACATTTCACACTTTGTACCTTGTCATGTTTCATCTCTTTCTTTGGCTTTTCTTCAA
CTCTCAGTGATCATCTTCCTCCACTGCAATGTACATACCATATAGCTTTACATCATTTTTTAAAATCTCA
TTTTATGAGTACTTTAACTGAGGTCTGCCCTCTTCATGGGTTATCAATGCTCCATTACTTTTCCTTTGAT
TTGTCCCACCTGTGCCATTTAAACAACCATTTCCTATTTGTAAAATATATTCAAGTACGAAATTACCTGC
AAAGTTTTGCAATTTTTAACTTTCTATTCTGAAAGTTTTATCCAAGATGTTTCCTTCATTTTATTTAGAA
ATCCATTTACTGCCATTTAGTGTTTTATTAACCACTCTTTCATTTTACTTTGTAAAAGTAAGGTTCATTG
AGGAATGAGTTGAAATCAAAGTCTCATAAAATGCAGTTAATTCACTAAGCAGTTGAGAAAATTGTGAAAA
GAGGCACTCATGAACCTGACACTGATGTCCTACAAGAGCAGTAGCTCAGAATTGTGTAATTCAGTGTTTG
GTCATTTCATATGGCAAAGCTACCACTCTCAAACAGTTGAGTGAATGTATTTAACAGTACACAGGTCTCT
TATTAAAGATAAAAATCTGAGTAACAAAAGAGTATATACTGCCAAAAGGAATCATAAAAGCTTTATGAAG
TAATTACCAAGTATTTCTTAAGTTTTCTCAATTCAGAAAATGTATCCCTTTCCCCTTCTTCATTTGTTGA

ATACTATTCTTTTCTTAAAAGGAAATTTTCAAACACTGCATCCTGTGTGTATTGGTATAAGTGTATGTGG
TTTTTGAGTGTTTATTTGCATCCTGATAATTTAAATTTGCATACAATTTTCAACAAATCTGAATTTTCAT
GTTTCTTTCCAGTGAAATTTATATTGACTTTTCATTGTATTTTTTACAGAAAAAACTCCAAAAAAAGATG
GTAAAGTATCTTTGATATCCATCTACTCTCAAAACATTTTAAAAGGCCTGCTTTCCTGATGTCTTACATA
AATCCCAGCTTTCATCAAGAAGCCTTCATTGGTTTGCACTCACCAGGATCTCTATCATTCTAAGACAATA
TTTGTTTTATCAACTTCTCTTGTACTCCTTGCTTTTCTGGTCTTTCAAAGCCTGAATAAAACCTGAAATC
ATTCACTGATCAAGCGTAAACATGAGGGTCCTTCTTACCCTGGCTAAAGTCTAATAATCCCAGGTGTGCA
AATACTCACATTTTTTTTCCTGGAATTTTTTTATATCCAAAGAGAGTAATTTAAATTCATTCTTATTTA
TTTTTATCAACACAATTGTCAATTTAGCCATACTTCAGCTATTTTGGAAAGATTTTAAATTTTTACGAAT
GATTTAAAAAAAGCATATATCTCAAATCTGAATATTTTAGGAGAATGTCACCATAAATTGTTGTCCTGCA
ATGCCTCTATAAGTTATTTTTTGAAATAAAATACAAAATTATTTATTAAAATTTTAATATCTTATTTTTC
TAATGACATTTTTCAGTATACTTTTGTTTTCAGGGAAATCAAATTTATCTCTATAATTCATGCATCAAAG
GCTAGGTAGTTATTGCTACAGATACTGTTTTGAACAAATGGGTAATCTTTTTTCTCTAGTTCCTCATCTC
TTTTTAACTAGCTCTGTTGGCATGGGTGCATGTTAATGTTTTTCCTGATAAGATGATTGAGATAAGACGA
CTTCATTTTGACATGTGTGAATCTAGCAATTCACCACAAAGTTGTGCCTTCATTTTTAACATGATGTGAA
TATAGGTTCCCAAGAACCAAACACTGCATTTTCTCTAAAGCAGTTCAGTATTCTCTAACTTATTGTTTGT
AGGTTTTTTATACATCAAAGCTACAACAAATATGAGATGTGATTATTTATATTTAACAGCAACCAATAAA
TTTATTAAAGCTAACTAACCAAACAAGTAATCACCTGTAATTCTAAAAAATCAATAACTTATATAAGGAA
AACCATTAACAGTATCATTCTTGAAACTCTTCCTCAAGTCAGGAAGATCACTCCTTTCCCTTTTATTAAT
TTTTGATCAGTACTATCAGGACATAAGGAAGCTTTCAGCTACGTTGGTACCTGTGTGTGTGCCTGCACAT
TGTGTGTATGTATCAGTGTTGGTCTATATCCTGTAACCTTTACCATTGTATGTGAATATTTCACTAAATT
TGAATTCTTTTGTGCTTTTGTGGCATGATTTTTACAGTAGTTTTCTGTTTTTATTTTTCAGATGACTTTC
AGAAAAAAATTTGGTAAATATATCTGACAGCATTAATATTCCAAAAGACTTTAAAGTATCCATTCTCAGCA
ATTTGAATATTTTCTGTTGGCTTGCCATCTGTTGGCTTGCTTATCTTTTTCCTGGATGTCTGTTTTAACT
CCCCAAATTATTTGTTTAGGAACTTCATGCATATTAAATTCTCTCCACTTTTATTACCCTACAGCTGATT
CTTTCCCAATGCTTCTTTTTTCATTTCTTATGTTCCTTCCCTCTAAACATTGTCCCAGTCTATCATTCGG
AGTTTTGCTAG

>FBXO9|hg19
CGGACCGCCTGGGGCGCCGAGCCCCGTGCTCGGCTCTCCACCCCGGCCCGCGGTAGCCCAGGGGCCCTGT
GTCCCGCCTACCGCCGAGGAGCTCTCCAGCTCCACACGGCCGTCTAGGGCATCAGGATGGAGAGATGCAG
GCCGAGGATACACGTTCAAAGGACATCCCATTACAGATGATCATGGTGGACACGCTCTCCTGGCTTTAAA
AAACATATCTGTAATGCCACTGAAACGTGGTTAAAGATGGCAAATTGCATCATACTTAACCATAATAAAA
AATATTTAAAACAAAAACCAGACTGCGTGTTAGCATCCATTGGAACTTCAAGAGTTTTTATGTTGTGAGC
AACTTTAGTTTGGAAAACTGGATTGTTACTCAGGTGGAGAACACTCTAGGTAACGCTCTACAGATCGGTC
TCAGGGTGTAACTAGTTATTTGAAATTTAACACTGCTTAAACGCCATCACTTGTGTCCTTAGTTATATTG
CAAAGGTAAATCATCCCTATGAGGCCTGGCTCTGAACCGCTTTGAAAAAATAAATTGCATCGAGGTCCTC
GATTTAAATTCACATAATTGCCCCAAGCCAAGGGTTCTCACACTTTAGCTGGAGGCATCAGAATCACCCC
CAAGAGCCTGTGAAAACAGATTGCTGGGGCCTCTCCCCCAGAATTCTGGTGCTAATACTACTGGTCAGGA
GAAACACATTGCCCTAAACAACACAATTTAAATTAATAAAGCAGTAATCCTTTCTCTGAGTTTAGATCTC
CCAGCTCATACCAGTTTTCTCTCACTGTCTTTGTTTTTAAATTCTAAGAACTGAAAAAATTATTTCCAAA
AAGAATCTATGCAAAAGGCCCGAATCCCTCTTTTCCCTTGCTTCTGTTGAAGAAACAAGATACAAGACTG
CCAAGCTTCCTTAGACTTCCTGTCCCAAATGGGAAAGGGTCATCTGTCCACTTGGCAATTGTATAACAAT
TATCTACATTCATGTTTACCTACATAACACCCCAAACCATATGGCAAATGAGAATTGAAAGTCATTATCA
ATGAAAAGATATGGAGACAGGAAGCAGCCTAACAAATCATATAATCACCCCTATGAAAAGTGATTTTTGA
AGTATTTAAAATGATCAGTTTTAGTCAAACTCAGTTGATCAAGCAATTGGCAAATGGACTGTCCTGATCT
ACCTTTAACAAGGGTCCACTGGGCTAGTAAGTGGCTTAGGTCAGTAGTCCTAGCCTAGGGAAAATTATAA
CCATGTGAGTATTACATAATATGATGAAATAATACAACCATGTAAAAGGGTCCAGTGGATAGAGTGGATA
CTTCTCTAAAATTTTAGATGACAGCATTTTAATAAAGTAGATGCTCAATTTATTGTTACAAATACTAATT
ATTTTATTAAACTTTGTTGTTCATTTATTAGAATAGAAATAATGTCATGAGCATTCAACCAGTTTAATGA
AATAGACTAGTTCTGAAAATATTTCAACACCCTATTCCTCATTTGCAAAACCGATCTTTTGTATTCATAA
ATCACATATAGCATTTCCTTCTACTTTTAGACTACATCTAGTCTTTCCCCCGTTTTAATTTCAAAATATG
ATTTAGAAGAGGCCATTGGGAGGAAAATCATTATTAAAAGACCTAAGCTCTACAGTCTCCTTTAAAAACT

ACAAACCGGTAAATTTTTAGTCTCCCTTAAGTAAGAATGTACTTTTTACAGGTGGCTGACTTAGGCAAAT
GAAAACATACCCATGAGAACGATGGATCCTAATATTAAAAGATAGTACTTGGGGCTTCCATAATATAGCT
CACCATTCTGCACCCCTCCCTTTCTTTTGCCTTTGTTTTTCTGCAGAAAAATGTACTGCATTACTAAGAT
CTAAATAAACAGAATAAAATAACGGATAAGAGATTTAAACTCTTGTGAATTCGTGGAATCACGAGTAGGG
TCTTGAAAACCTGGTGACTATTAGTAACCCACACCTAGTAGAAACCTCAGAAACTCCATAAATGTTTCCT
TAAAGAAGGTAAGAAGCTCCAAGGATAGGTTTACACACAGCATGCCAAAGTCAGCATTTTAAACAACGTC
CAGTGTTGGAAAGTAATATACCGCCAGCCAAGCCGGAATGCCTTCTGCGGAAGTGAAACCAGGTGTTTGG
ACTTTAAGAGGCGGGGCCAGAACAGCGTGTGAGTACCGGCGCTCTACGTGGAACGGCTGACTACAAATCC
CAGTGTGCCACGGGAGCCGTCGCTGCAGTCTATCGGGGCCGTAGAGCCCCAGGGTCCTCCTCGCGTTGCA
TGATGGGGTTTGCAGTGCGACTTGCGTTGCAGGGGGTCGGGGGCTAAGTTTTCTCTCCTGCTTTTTCCTG
AGCGCCTCTTTTTCTCAGCCTCCAGAAAAAGGTGTTTTTGAGAAGCTTAGTAATTATGACAGCCTTACGA
TAGTGGAATTCCACTGTTTCCTTGTAGTCCTGGATTGGGATTCTAGCCACTGTCGCCGAGAACCGTGTAG
CGACCATCCCGAGCACCCAATTCGATACCTTAGGACCGCTTGCCTGAGGGTCTTAATTCGAAGTTTGAGC
ACCCCACTCCAGTACTCTTGCCTGGAAAATCCCAATGGATGGAGGAGCCTGATGGGTTACAGTCCATGGG
GTCGCTAAGAGTCGGACACGACTGAGCGACTTCACTTTCACGCATTGGAGAAGGAAATGGCAACCCACTC
CAGTGTTCTTGCCTTGAGAATCCCAGGGACGGGCGAGCCTGGTGGGCTGCCGCCTATGGGGTCGCATACC
GTCGGACACGACTGAAGCGACTTAGCAGCAGCAGCAGCAGCAAGTTTGCATAAGTTCACTGAACCGAACA
CTAGGTCGAGTTTGGCCTTTGGACACCCGATGACCTAACCCGGCCTGTGCCTCGCGCGCAAACCAAGTGA
ATGTCCCTCGCCCTCCACCGGGCAGCAAGGGGGCCGCGGAGCCGTGCGACGGCGGGTCGGCAGTGCGCAT
GCTCGCCGCGCCGGCGCAGGTTTCCGCAGCAGACTAGCAGCTCCGCTGCGGCGTCCGGGGTTTTCAGCAG
GGCCGGCGCTCCGGTTCGCGCAGAGTCCTTGGCCTCTCGGCGGGCCCCCGGCGTCCCTGCGCTCCGCGAG
TCCCCGGCCGCCGCCCAGCGCGCCACGATCTGGCCCTCTGCCCCGCGAAGATGGCTGCCGTACGCCGGGC
CCGCAGTTACTGCCGATGCCTGGTGCGCTTCTCCGACCGAGAACTCTGCTAAGCCCCGCTGCAGCAGACG
GCAGGAGTAGA

>TUBB|hg19
ATCCGTCATAATATACCTGTTTTTTTTTTTTTTTCCCTTAAAAATAAATACAGTCAAAAGAGGTTAAAAAT
CCTTACCTTGGCACCGATCTGATTGCCACACTGACCGGCCTGGATGTGCACGATTTCCCTCATTGTTAAA
ATTTATTCTAATCTTTTTGCTCGCCTCAAAGTGTATACGGGGCAAAAAAATATTATGGAATTAGTTTTTC
TCTGCTGCTAGTCGCAGGGTGGAAAGATGGAACGGGACCCGGAGGCTGAAGCAGCGAGGTCCGAACGCGG
CGGCAGGAAGGTTCTGAGAGGGAGAAAGGAGAGGGGAGGGCGCGGGAGGAAAGGCGGGCAGGGCGGGGCG
CGCGTGCGCGGGGGCCGGGAGGCGGGAGAACTCGCCGCGCGCCGCGGTGGAGAGACAAAGCCTCACTAGG
CTGGCCCTGATTAGTCCGTGCCGGTCCTGTATCACACGTCCATTGGTCCCTGCTGGACGAGCGCAGTCCT
GCCTGGGAGTTGTAGTTCTCTATTGTTGTCCACGCTGCAAAATGAAAGGACGAATGGGTGGGTACTGGGT
CTGGGGGTTTTGGTTTGGGTGTTTTGACTGAAAAACGGCCTGGCGCTAAGGTTCCTTCCTCTATGCCAGA
CTCTTCTGGTATCAAAAGAGAGTAGTTTTCGAGGGTACAGGAATTGGGAACCCTGGTGTGCCAGGCTTTG
GAAGGTGGAGTGGGGGCACGCCCTGAAAGCTCCCCGCTGCGGTAGCTCTTGGGAAGACAGGACTGCTTAG
CTCAGCGAAAACGGGGAGAAATGCGGCACCCTGATCTGCTCTCTAACAGCTGGTCCCTGGTGACCCAACT
GGCATGAACTCCAGTAAGCTGCGGCTCCCCCAGATACTTCGGGAAGGAGGAGGGCATTGCCTTCCTGCTA
TTTCCTTGGGAGCTTTCTAATGATAATATTTAGAAAGAAACAAAATTGCATCAAGGACTTTTCAGTATAA
AAACTAATTGTCTGGAAGGTATGTTCAAGAATAGAGAAGCAGATATGCCGGAAGTCTCCGTCCATGTGGG
GATCTGCGCCTTACAGCCGCATTTAACACACTGTTAAGAGGCGAACTGTGGGGTGAGATACCTGATAGGC
ACTTTGATGAAGTCAGTGTTTTTTCAATTCGCTGATTTCACTGAGAGGTGGTTGACTATTGCAACCTTTC
AAGAACCTCGGGATTAAAATAAAAGGGGTAGGGAGGGGGATGGAAAAAAATTGGATGAGCTCAAATCTG
AGTGGCATGTTTCTTCGCAGGGATTAATTCAACTTGAGAGATATTAAATGTCTACCAAACCAGGAGCTAG
GAATACAAAAATGATTAAGGATTAGCTCACTGTTCTCCAGGATCTTGCAATTTGGTGAGAAGTGTATAAA
CAAACACTAGAGTGTGGTAAGCACCATGATAAAGGACATGGGATATCAGGCTCCCTGAGTGTGTGCTCAG
TCATGTACGACTCTTTGCGACCCCATGGACTGCAGCCTGCCTGGCTTCTCTGTCCATGGGAGTTTCCAGT
CAAGAATACTGGAGCTGGTTGCCATTTCCTTCTACACGGAATCTTTCCGGCCCAGAGATCGAACCCGTGT
CTCCTGCATCTCCTGAATTGGCAAGAGGGCTGTTTACTTAAGAGGCACTTGGTTAGATGATAGCAAGGTG
TCAATTCACACTGGCTTCAACACACATTTCTCGTCTTCTAAATAAAAGAACATAAATTTCTCTAAGACTC
GTCTACCCAGTAAAGGACTTGCCTGGTGGCTCAGACCGGTAAAGCATCTGTCTACAATGCGGGAGACCCG
GGTTCAATCCCTGGGTTTGGAAGATCCCCTGGAGAAGGAAATGGCAATACACTCCAGTACTATTGCCTGG

AAAATCCCATGGACAAAGGAGCCTGATAGTCTACAGTCCATCGGGTTGCAAAGAGTCGGACAAGACTGTG
CGACTTCACTTTCACCCAGTTAAAACGTTCATGTTTGAAGGCACAATCACAGTGAAAGTAGTTATGTATT
TTGTGGTAGTGAAGAGAGTTCAGAATTCACGATTAAACTGTTTCAATTTGTCTCTAACAATAGCCGTAGG
AGCCTAGGCAAGCTAACTCCGCAGAACTCTTATTTCTGGTCAGAATGAAGTGAGAATACGAATGCAAAAG
GTCCTCCTAAATTATTCCTGTGAAATTGTCTTGTCTTAGTATGAAATTTAGCAAAATAAAAATTATCATT
CTGTAGCCCTTCAAAGACCTCGGTCAGCCTTTAATATGGTTAACAAAAGAGTTCTCTCCGTTTGCCTAGA
TATCTTTACTCTAGGAAAATGACCTCCAAAAGTGTCCATCAGGAATGGATTCTCAGGGAGGAAATGACAT
GACTTCTAGATGAGTTAAATCAAAAGGAAATGAACTTCCATTGTAGTGAGTCCTCAAGCGCCACGCCTAC
GGGCTATTCTCCAAACTGAAGTCTTCAGCCACGACTGCAGCCCGCAACCCTTATATTTCTCATGAGTCAG
CGGACACCATTTCTGGGAGGACCGAGAAGGCGCTCTGGCCGCACTACATGCTTAGGTCGACGACTGTGAT
AAGCCCCTCTATCCGCTGCTGGCATCCAGACTCTGTGGTCGCAACTTGGAAATATACGCGGGAAGTGGGT
GGTGAAACCGCAGTGGGTTGTCCCTTTGGAGCTGCCCAATCGAAGTGCGCCATTCAGTGCGCCTCCGCGG
CTTCCGATTACCGCCTCGCCCAACGGATCTCAGTCTCCCCGGAGAAGCCGCCCCTTTGAGCCGTAAGGGG
GCGGGTCGGGTCGGGTGGGGCTCCGGCTTTGCCTCCGCGTTGGATTGCTCCGTCTGCTTGCCGCAGGGCC
GAGAAAGACAAAGGCTTCAGCCAATCAGCTAATCGGCCCCTTCCCCGCTCAGGCACTGGCACCGCCCCTG
TCGCAGCCGTTACTGGTGGCGCGCGCGGGGACTCAAAGTAGGTGAGTTCTGGGGGGCCTGGCCCACGACT
CCCCGGATGCCATCTTGGTTCTCCCGGAGGGCGGGAGGGGCGTGCCTTGGGTACGACTGGGCGGAGGATT
CTTGGAAATTTGCTTCTCGGATTATAACCCTCATCTTCACTTGACGGCCCTCCCTGAGTTTAGGTATGAA
GACACTGGGAGAGGTTGGGCTCTGAGGGAACCCTTGGGATCGAGATCAAAGATGAAGGCCCATTGGATTG
GAGCCGAAGGTCATGGGCGCCAGCTGACTTCGCCATCATGAAACGATGTTAACACTTTCTCCGTGGAGGT
GTCCCACTTTC

>C23H6orf136|hg19
ATTTAGGAGGAGGAAGTAGCTGAGAATGATGTTAGAAAAGGGGTCAGAGGTGGTGAGAGGCTGGCAGGGA
AGGGGCTCAGACCTCACCTATTCTCTGTTTCCTGTTCCCCCTGCTTCAGTTCTTGGTTGCCTGTGGAAAT
ACCAGGCATGTATATGGGAAGGCTAGAGTAGGTAGTGAATATGGCCTATTTGATTTGAGGGTAAGGAGAT
GAGTAACTTACCTGTATTGGGACATGAGGAATTCAACCGCCCGTGGCGGCTATAGCTGCAGCTTTGGGCT
ACCAGCCCTGGGGGAGTCCCGCTGTAGAAGAAATGTGAAATGATGGGGAAGAGGCTACAGGATGAAAGAT
GAGTGGGTATGGAGCAATCAGGGGAGAAGAATAAAAGAACAGCACAGGAACAGAGAGGGGGTAGTTATCA
TTCCTATGGGGTTTAATACAAGTTGTGCCCCAGAAAAGGGAAGGGAGAGCACTGTATACCCCAAGGTTTT
CTGGAGCAGTCCTAATTTGCTGCTTTATTATTTCTAGTTGAGGCAATGTGCTAACCGTAAGGTTAAATGT
GATCGAAATCATGTTATAAAAAGATTCAGGAGAATTCCCTGGTGGTCCAGTGGTTAGGACTCTGTGCTTC
CCCTGCGGGGGGCACAGGTTCCATTCCTGGTTGGGACATTAAGATCCCACAAGCTACACAGTGCAGCCAA
AAACAAAAAATGGATTCACATAAAGGCAAAATTTTGCTGAACCAGGTGCTCTGATGTTTTGAGTTGGAAA
ACACGTTTACCTTAATTATGGGATATCTTAGCCAGAGGGGAAGAGGGAGTGGCAAGGCTGAATTTAGGAA
AAGGATTGTCTTGAGAAAGAGATCAAAGGATTTAGAGAAGAATAATCCAAGAAACAATTAGGGGATTGTT
ATCAGAAGGCAATTAATTGGCTCCTAGAGATGTTAGTAAATCTTAACATCTCAGCAAGGGCTGGCCTGGG
TAAACAGTAGGAGTTACTAGAGGGAGAGCTAAAGGTTTATGGAGAGAATGGAGGAATAGATACAGAAGCC
TACATGGAGGGATCCAGTGACTACATTAGAGGAGACAGAATCTGCAGGAGAAAATCCAGCAGTGAAGATG
GGGACAGGCAGACACCAGATCCTGCGAGGGGAGCTCTAAGCCTCCTTGCTGGAAGATCTGCAGATCTCAG
AGGTGAAGAAGAGGGCCAGAGGTCCTGAGATCCCAGGAAAATACAGGGTGACGCACAAACGAGGCTGAGA
CCTTAAGGTTTGTTTGTTTGTTTTTTCATTGAAAATGTCCATTTAAAAAACACAAAAGAGTTCACGCTTT
ATTCAGACAACACTGAGAGGGAGAAGAAAAAGGAGGAGTGAGTCAGAAAAGAGGGAGATCCTGCTCCCAA
GGATTTGGGGGAACACAGTAGGGGTACCTGAACTAGCACACATCCAGAGGGTAGGGAGGGAAAGGATTTC
TATAGCCTGGGTATGGAGCAGGGGAGGATTCTTGCTATGGAGGAAAGAAGAGAGGAAGGCCAGAGGAAGA
GTTCCCCCATCATCATCAAGACAATCTCACAAGACAGGAATCTATCAGTATATCTAGCTACCGGGGAGGA
ATGGATGTAAGAGGGTATTCCAGGAGCTAATAGAGCCTTGAAACTCAGGCTTAGCTGCTGAAACTTGGCT
AAGAGTGATCCTAGGGTGTTGGCTGGGATCTACTGCCAAGAAACAGACTCCTTCAGAGAACCTGGACCTA
GGGAGGTGGTAGTGTCTATAGAAGCGTGATGAGTCTAGAGTCCCACAGCAGACATCAGCTCAGGGGTCCC
GACAGATTAACACATACATGAAACGTGGAGATTAAGAGTTTGGCAATGTTGAGTGGATCCCGTGGGGAGC
TGGTAAGTCCCTGTCCTACCTGACTGGACTGCTATGAAGCAGAGTTCGTTCAGTGGCTGGCGTCAGCAGG
CTGATAGGGTAGCTGCAGCCGGTAGCACAAAGCAGTATTAGAACTACCAGCAGACAGTGTAGAGGACTAC
AGGAGTTATTCCATGGACAAGACTCTTTTACAAAGGTTTCCCTGGCAGAGGAGGCCTTTGGACTTTCCAT

GATGTTCTAGAATCTGACAGCAAGCAAGAGTGTGCTTCTCTGGATAATTAGTATGAGCTGCCCAACCAAG
AGGCCTGGCAGCAAATCGGGCTGTCTGTGGGAGTGTTCAGAGCCCAAGTTAGGAGAGAAGGGCTGCTTTC
TGTGGTAAGAAAAGCTATGAAGTGTTAGGCCACCTGGGACCCTGACCACCCAGTTGTCCAAGAAAGGGGT
AAAGGGAATCCTACGGGTTCTTGTCAAGTCCCCCTAGATGCTGGGGGATGCAGTGGGAGACTGGGTCTGG
GGTTCTCAGGGAGAAGGGTCTCCACCTTCGCCAGGTCTCCCAGGGAGGGGAGGGGGTTAGCAGTCTATCA
GTATCGACTCTCATCAGAGCGGGGAAGAGAACTGGGGAAGGATGTTATGGACAGGAGAGGGGGAGAGGGA
AAATGGAGGGGAGAGAAAAACAGAAAGAGAAGTCAGAGGTGAAGAGAAAGCTGGGGAGAGAAATAAAGGA
GAGGCCCAGTCCCTGCCTGCCGGGGTGGGCTCCCGCCACACAAGGCACTGAGCCGGGGCCAGTGGCTCCC
CAGGCTGTCAGAGACCCTGCCCCCTCCCAGCAAGTGTTAAACCACAATTTCCCGCCCCTCCCACCAGTGG
GTCTGCGTTCTTTCTAACCTTCCTCTAGGCACTAGCATCCCTAGGCTCCCCCGAAGTCCTATTCTGCTCC
TTAACTGTAATTTATTTCCAGACTATTTCCACAGGTATCTTCCAGTATATTCTCAAAACGTCTTAGGAAG
TCTCCAATCCTCCTGTCCCAATGCTTCATGTCTCTGATTTACACTGCCCTCTGCCATTTCTAACCGCCCT
GTCTGACAGTCCTGAGGCTCCCTAACTCTCCAGCTCCTGTTACCTGGTGCGGCGCCGCTGGGCAGGGCTG
CCCCCAGGGTCAGTAGCAAGCAGAAGGCGGGCGGTGGGGTGTGGGGGGCAGAGGCGCAGGGAACGCAGCA
GCTCCTGCTCTGGCACAAAGGGGCGGAGGGGGCCCCGTTCTGGTGAGTTTCCCAGGCTGCTGGAGCGAGG
GGGTGGGTGGGCTGGAGGAGTCGCACGGCGAGGGGCCCTGTTCAGGGGCCACGGAGGCTCCACAGCTACC
TTCAGAACTGGCGGAGGGAGAAAGCAGAGCAAGACAGAGAAAGAAGGAAAAGGACAGGGAAGGGAGTTGT
GAGAGCCAAGA

>ATAT1|hg19
CGCGACGTCACTTCCGCCCCTCCCACCGCAATCTGTATTTCCCCTGCCGCGCCTGCAGGTCCCCAGGGGA
CCGCCCCTCCCCGCGCTCCCCTCCTCCACCATCCGCCTGTATCGCAGCAGCTGGCCCCGGAGACCCCAAT
CTTCTTGCCAGACCAGCTGTCGCTTAGCAACAGCACTCAGCCCATCCTCGCCTCCCACGGCAGCGGGCGC
CACAGCAACCCATTTTTGGGGCCTAGTGTCTCATTCTCCAAACCTTTCAGCCCCTCAGTGAACAGCTGAC
AATAGTGAGAACGCTGTGCCTCCGGCATAGGGGGCCACAAGTGATCAAGAGAGAATGGTACAACTGCTCA
TTTCAGGTGCCTTCTGGTCCCAAGAGCCCAGAGGCAGGGTTGGTTCAAACCTGACCACCCCTCTCCGCAC
AGCAGCACCCACTGCCCACTCCCTCACCAACCCCAACCCCCACCACAGCATTCATTCATTTGAAATTCAG
CCCAGAGACAGGATACAGCTTTATTGTGAGAAAATGCAGCTGGTACAAGTCTGAAATAGTAACTGAATGT
GTAACCCATCTATTTACAACAGCAAGGACTGAGGTTTCCCATACAAATCTGTACTACCTTCCCTCAGCCT
CATGTGTATTCATCCCTTTCACCAACAATCATCTCTGGTGATTGAGCTCGCCACTGGCCACAAAGTAGGC
ACAACTCTTCAGGATGAAGTATTGTGTAGGAGACCTGCCTGTTCCCATCCCAGTCTGTACCTACAGAGCA
CTCTGGGGGCCCGCCTGCTCAGTGGAGGTGGCTTCAGTGGGGGCCGTGAGTGGCACCTTGGGCATTGACT
CAGGTGGAGGGCCACTTTCTTCTCGCAGATGGCCCTGGTAGAGCCGGCGAAGGCGGGACAGCTCTCTCTC
TGGTGGCTGTTTCCAGCTGTACCATGGATACCAGGGGTCACCAGAAACCAGAGTGGGGGCTGGTGGAGTA
GCACTCACAGCTCCATGAGAGGTACTGAAGTCAAGGTCCCGAGGTTCAACCTGGGGAATGTGGTAACTGA
GGAGACCTAGGGGGTTAAAAATAAAAGAGAAAGCCAGGGGAGAGGAAAGGGGTAATTCAGATTAACAAAG
ATAATAAGGAAAATAACCTGTAAAAGAAGGAGGGACCAATCAGAGCCACTGGGTACATTTATGCATGTGG
TGTGCCACCCAAAGGCAACTGGCCAAGGGGGCAAGTGTGGGCTGACGTGCTGCCTGGGCTCTTGCCTGCC
AAGCTGTGCAGTCCTGTGCCAGGTGAGGAAGCTGCATCTGCCCTAACAGAGGGCTACCTGTTCCTAACTT
GTGTAAAAGTGTCATGGAGCCCAGGGTCAAAAATTTATACAAAGCTCTTATGATCTAGCCATGGCACTGG
GACCAACAGTTACAAAATAAGAAGCAGAGAAAGAACAAAGGGACAGGGCAATGACAAGGTAAGAGATTCA
GGAGAGGAAACTAAAGGACAGTGGAATGACGAAAGGAGAGACTCTTGAGAAGAACATGTTGGGTGCTACT
TCTCAGACAGCTGGGAAGATTTATATCCACTCTCCAGCCCTAAGTCACTATCCTTATGCCTAAGAAAACC
ACCCTGTGCCCCTTCTCATGCTCACCATGATCCCTGGCTTTCTGGATAGCCTGGGTCAACTTCTTGTGTT
GCTTCATACAGACCCCTGTGGGGAGAAAAAAGGTAACTCACAGCTCATTTGATAAGGCACAGTAAATAAA
GAACATACCTGCTTCCTTATGGTGATTGAGGGCTAGTTATGGAAGACCAGCTCTACCTAATGACCCCATT
CCCCTGACCTCGCCATGAGCCTTGGTTTCCCCTTCTGCTCAGTGGCAAGAATGCTCTCTGAGAATCAGGA
TGAAGACTTAGGAGGGTCTGGGTAGTACAGAGAAGCCATTAAATGGTAAAGGCTTCAAGACAAGGAGCAG
AACACCATTCAAAAGGCAAGTGTGATGGGACTTCCCTGGTGGCCCAGTGGCTAAGACTCCCACTCCCAAT
GCGGGGGACCTGGGTTCGATCCCTGGTCAGGGAACTAGATCCCACATGCCACAACTAAGAGTCTGCATGC
TGCAACTATAGATCCCACGTGCCACAACTAGAAGTTCAAGGATACTGTGTGCCACATCTAAGACCTGGTG
CAACCCAATAAGTAAATAAAAATAAATATTAAAAAGAAAAGGAAAGTGTGGTTAAAGGGCTGACTTGAAC
AATCCACAGAGAAAGATTCAATAAAGGAAGAAAGGATTTCAGATCTGGGGAAGATTATCTGAGAAAGTGG

CAAAATAGTTCCTTCAGCCTTTTACAGCTTGACTATAAAAATCTTTTTAAAATTCCCTGGGAATTTCCTG
GGGGTCCAGTGGTTAGGGCTCCTCCTTGTTCTCACTGCTGAAGGCTTGGGTTTGACCTTTGGTCAGAGAA
CTAAAACCCCACAAGCCACACGTGCAGCCCAAAGAAAAAAAACCAACTCCTTGAAAAACCAAACTCCACT
GACCAGGCTGCTGCTGCCGCTGCTGCTAAGTCGCTTCAGTCATGTCCAACTCTGTGCAATCCCGTAGACG
GCAGCCCACCAGGCTCCCCCGTCCCTGGGATTCTCCAGGCAAGAACACTGGAGTGGGTTGCCATTTCCTT
CTCCAATGCAGGAAAAGTGAAAAGTGAAAGTGAAGTCGCTCAGTCGTGCCCGACTCTTAGCGACCCCATG
GACTGCCGCCTACCAGGCTCCTCCGTCCATGGGATTTTCCAGGCAAGTGTACTGGAGTGGGGTACCAGTG
CCTTCTCCGACTGACCAGGCTACCACCCCCTAAAAGGGTGGATTCTGATTATTCTTTGCAAATTTATCTT
GCTTTGGAACAGAGGCTGGCAAACTGCTTCTGTACAGGGCCATACACCATAGGCACTGTGGCATATATTT
TAGGCATATATTATCATCTGTCTCAGCTCAATTCTGCCATTGCAGACTGGAAGCAGTTATAAACAGTATG
GAAATAAATGGACATGAGAGTGAGCTGGATTTGGCCTGTGGTTGTAGTTAGGGAATAAACAGATGTAATC
TTGAATTGGTGAGTTTACACCAAATTTGGGAAATCTAACTGAAGTGTCAATTATATTAAAGAACAGTCAA
TTTATGAGAAAGCCTTCCAGAAAAGTGAAGAGACCCTAGAGAAAGGAAACAATCAGCACCATTTATCTTT
TAAGAGCTCCATTTAGATAGTCTGCTTATATCAGTACTCACCTGGATCTTTTGTCCATATGTAATTTATT
CAGTATATGAC

>MRPS18B|hg19
AATTGCGTTTTTGCTGGACCAGTCACTATGACTCTTAACTCTCATTCTTAGAGTCTGTGTTTTAAGCAAT
TTTGAAGTCGCTTTTGACCGTAATCCTTTCATTGCGCATGCGCCGGTCCGATTCTACCAACCCTTGGAGA
GAGATGTTCTGGAGGGCGGAAGTGGTGGAAGCAGAGGAAAGGGCGGTACTAGGCTGCTTGGTCACGCGCA
CGCGTTAGGGGGGGCACCCCCCTCCAAGGAAGGAGGCGGAAGAGGGTGCCCGGGGGAAGGGGCTGAGCGA
AGGTTAAGGCTGGATATCCAATCAGCTGGTGAGTGGTTTATCGGGTTGACCAATGAATTCACGCCTCGCG
CATACTGTCTCGCGAAGAGGGCGTTTCACTAGCACGTTTGGGCGCATTGGGCGGTGTCCTCGTATAAAAG
ACTCCGCCCGAGCGGGCAGCCGCCATTCTGGGGTTCGTTTAGAGGTAAGTGTTGCCATTTTGTCGGTTGG
TGGGTAACATTTTGCTCGGAGAGTTGGCCTGGGGAACGGGTCAAACGCGGGCTCAGTTGAGAGCCATGAC
TCGTATTAAGCGGCTGTGTTCGGGGTGGGACATGGAAGTGTTTGGGGACAACAAAAATGGTACTTTTGGG
TGTTGTAATAGCAGCCCGTACTGATTATCTTTCTCCCTTGTGTTCCTTTTATCCCAGGTTTGAATTTTCT
CGGAGAAAGACAGGCCGGCCACCAGGAGAAAAGAAACAAGCCGCAGCAACATCTAAGCCCTTGAAAGGAT
CCAGACAGAGGGGGGAAAGGGAAAAGAGCAGCCACCAGCCCAACCACTTGTGTCTTCTGCCCCTTCCCAC
CTATCTTGCCCACCCCACCAGCCCACGCTGCCTGGGACTTGAAATCTGTGGCCGAAGGACCGTCACCACA
TAACTTCAAAAATAATCAACCACCCACCCTTCCCAAACTCACCCAAGTCCACTCATCCAGCGTTTACTTT
TTTTAATCCACTCAGAACTTTTTTTCTACGACCCCCCTCCCTAAATGGAGTTGGGTGGGGGGGATATGAA
TACTGAGTTGGCCTTTATTTTTTCTAAAGAGATTTTTTGATCCAATGAGGCCCCCCAAATAATTGAGTTT
TGGGTCCTGGTTGGTTGTTTTATTTTTTTCTCCAAAATTTTAATCCCCTCCCCCCCTGAGCCCGAGGTGC
TGACGTCGCAAAAAAATTGGATAGTAAGTGTCGAATTTTCAAAAACCAGCCTTGCAACAAGAAATCAACG
TTTCCCGTTGTGAAACCAAAAATAATGAGAGGAAGAAATGAGACCATAGAACAAATAGAGATCTGGAGAA
GGACCGATCCGGTCACTTAATCTCCATTAAAAAGAGCCTTTGGGTCTAAAACAGTGTTCTCTCTCCCTTA
CCACCTACCTCTTCCCTGCTCTGCTGCAGGCAAGGAGAGGGTGGAGGGAGGGCCTGCGAGTTAGGATTTT
GATGGTTAACCCTTGGTAATCCAGCCAATTTCCAGACTGCCAAAGCTATAAGTGTTTGCTTTTTATAAGG
AGGAATGGTTACCTTCCTCTCGTTAGCAGTCTTCAAAAGGGTTAATGAGCTCACATACAAAAGATGGTGG
ATCTTGTGGCCCAGAAGTCCCACTTCTTTGTGTGTGAGACAAGCAGGGCGCTTTTCAATTTGCCAGATTA
ATAAGCAATCTGTACATAAAGATTGCAGGGTAAGGAGATGGGCGGAGAGAAGTGATAGTTGGGTATATTT
TTAAATGATTGTTAACTCCTCTCAATTTTAATGTAATATGCTTGGGGTATATTTGTGTGGTTAATTCAGG
TGGCAATCTAGGAATAAAGATTGCAAGACACGTAATTCAGGGCAAAATATAATCTTAGTGCCAATACATA
CAAGATTGATAGCTGGGGAGGTAAAGAATGTTTTGTAATTGTTGCAGATTGCATAGGTTATTTAAAAGCA
GAAAAAGTAACATTTTAACTTGATACTTTCTGGTCGGGGGAATACAGTTAGAGGGTGGAAAGGTTGGGGA
AATGTGGAATTCAGGCGTATTAATTTTACAAGCCATTTAGTGCAATTGAATGCTATCTAAAAGATTTGTG
TCGTTAAAAGGAATTTTTTTTTCTTCCCTGCTTCAATATGGCGACTTTCTTTGTCCTTTTGTCTTCCAAG
CGGCGTTGCAGGTTGTGTCTTGACCCATTTTTCTCCTGTCCTCCCACCTCAACCCTATTTGTTGAGAGGG
GTCCAAGTCCACCCCCCCCCTTTCCCTTTATAGGGAAGATGCGGTGGGGGGGGGCGCGGTGGGTGGTCGT
TAGTAGCAAGTTGAACCTCAATTGCAAATTCCGTTCCTCCCCGCAGAAAATTGTTCTTTTAAATATTTTA
ATTGCTTGTATTCTCACAAAACTCTTATTGATTCAAAATAGGTCAGACTATTCAGGATGGGGCTGCCTTT
TTCTTTTCTTTTTTACTAAAAAGGTGTTTTTAAATGTAAAGAGCTACCGGCGGAGCCTGGATAGCTTTAA

GTTTGAGTCCTCTTCTCCATTACAAAATGGCGTCCCAGCTTCTTTGTTTTTCCTTCCCCGTTTGCCATCC
AGAGCTTAGGGCCGCCCTAGAAACTTGGTCAAGGGGGAGGAGCCGCCCTGAAAGGTGATGTAATCAGCTT
TCTTCCTTTCTCCACCCTCGAAGCCCCGCCCCCGAGCAGATGCCTTCTGGATTCCTCATGCACGCTGCTG
CTAGACGGGAAAAGCCATTTTCCTTATGTTCTTTAACGTTTTGTAACCATTATCTTGAAAAAAAAAAAAA
AGCTTGTAGCAATTAGATCTTGAAGCTGTCTCCTTGGGCAGCTCTAGTTTTTGTATATAAGGATCCCGAG
CCTGTTGCTTTTACTTTCACCCTTTCTCACTCATCACTACTGCTTGGAAGTTTTCCCTGCTTTAGTTGCC
TGGGGTGGAAAACCAGTCAAACAGCTGTATTTTAGGACTGGAAGCTTTAGCGGGAGCGTGTGTGTGTGTG
AGTGTGTGTTGGGGGATCCTGGGGAAAGACACAAAGATGGTGAAAGTAACAGGAGTAAACAGCTGGGGCC
TGATGACCCTGAAATGTGCCGTTTGTGCTCAGGAATCAGTCCGTCCTTGGGCCTCCTGCGTTTGGTCGAT
TTGTATAATGGAAATCTGGGGGAGAGTGGGTACTTGAGGACTTACTTCTAAATGGCCCTTATTCCCGCCT
CTGGGTTTGGGGGTGAGCTCTTTGTGTTCAGTGCTGTGTCACAGTTCTCCAGTTGCTTAGCTCCTGTACA
CTGGTACTAAG

>ABCF1|hg19
CGCGCCGTCCAGTAGTGGGCGTGCCTGAGACCTGCTGGGGCGTGGCCTGCTATGACAGGGCGTGGCTGGT
GCCCGCCCACTCGGGTCGCGACAGCAGGTTTAAGACAGTCGAGGAACCCCAATGATTCTACGACGCCGCA
TGACTAAGTGACAGAACATAATCATTTCCAACTAGTATGAAAGAGTGAAGTCTTTTTTTTTTTTTTTAAT
AAAAGTGAAGTTGCTCAGTCGTGTGCAACTCTTGCGATTCCATGGACTGTAGCTTACCAGGCTCCTCCGT
CCATGGAATTTTCCAGGCAAGAGTACCGGAGTGGGTTGCCATTTCCTTCTCCATGAAGTCTTTAAAAAAA
AAACACAAGACAAAACTGAACGCTACCTTGTTACCAGTCCTTTCTGATTGGTAAGGAGCATAAAAGCAAA
GTCCACTAACTCAGGAAGAGGCTTTGTCGGTCTAGTGTTGAGATATGTCACAAAATCCCGAAGTGTAGAG
ATGTAAACGAGGAACTGTAGCCTAACAAACAGGAATCACAGTGCAGTGGGGCTTGGGTTCACTTTTTTTT
TGGTAACAGCTTTTCCAAGGTGTAACTGACTTAAGGTAAACTTTATATATTAATACAATAGATAACCAAT
AAACTGCAAAGTGAATGCTATAAGTTTTGACATGTTCAGTTCAGTTCAGTCACTCAGTGTGTCCGACTCT
TAGCGACCCATGAACCGCAGCACTCCAGGCCTCCCTGTCCATCACCAACTCCCAGAGTCCACCCAAACCC
ATGTCCATCGAGTAGGTGATGCCATCCAACCATCTCATCCTCTGTCGTCCCCTTCTCCTCCTGCCCTCAA
TCTTTCCCAGCATCAGGGTCTTTTCCAATGAGTCAGCTCTTTGCATCAGGTGGCCAAAGTATTGGAGTTT
CAGTTTCACCATCAGTCCTTCCAATGAACACCCAGGACTGATCTCCTTTAGGATGGACTGGTTGGATCTC
CTTGCAATTCAAGGGACTCCCAAGAGTCTTCTCCAACACCACAGTTCAAAAGCATCAATTCTTCGGTGCT
CAGCTTTCTTTATAGTCCAACTCTCACATCCATACATGACCACTGGAAAAACCATAGCCTTGACTAGACA
GACCTTTGTTTCCAAAGTAATGTCTCTGCTTTTTAATGTGCTGTCTAGGTTGGTCATAACTTTCCTTCCA
AGGAGTAAGCGTCTTTTAATTTCATGGCTTCAATCACCATCTGCAGTGATTTTGGAGCCCAGAAAAATAA
AGTCAGCCACTGTTTCCCCATCTATTTGCCATGAAGTGATGGGACCGTATGCCATGATCTTAAATCACCA
CCAAAATCAATATTTGGGGCACTTCGAATTCTACCACTAAATAGGTGTGGGACCAAAGTGCTCCCATAGC
TCTGCCAGCTCCCTTCTCTCCTTAAACTGCTACAGTCTTGCTTTTGTCCTCACCATTCCCTGGCAAGGTC
ACCACTCTTGCCAAGGTCAATGGATTGGGGCCAGTTCTCTGTCTTTATCCTACTCACTTCTCAGCATCAC
TTGATACAGATGGCCACTCCCTTCTCCTTTTGCTTTCTTCAGAGGCACTACTGTTTCCAAATTTCCTTTC
CTGGCTACTCCCTTTTCTCTTTTCCTGGCTTCTCCTCCTGAAGTCTAAATATTGCAGTGCCTCCAGGCTC
AACTGTCTTTTGTCTAGACTTATTACACATCCCTGTGTTGGGAAGATCCCCTTGAGGAGGAAAATGGCAA
CCCTCTCCAGTATTCATGCCTGAAAAATGCCATGGACAGAAGCACCTGGCAAGCTACAGTCCAAAGGGTC
ACAAAGAGTTGGATACAACTGAGCGACTAAGCACACACACATGCACATAACTTTAAATATCATCCAAAAC
TGATGAAACTCCTCAAATCTCTCTTTTTGGCTCAAATTTCTGCCCTAAACTCATGAATCCAACTGTTTAT
TGGACATCCTCTCTGAGATCTCTTATGCTGCTGCTGCTGCTGCTGCTAAGTCGCTTCAGTCGTGTCCGAC
TCTGTGCGACCCCATAGATGGCAGCCCACCAGAGATCTCTTATAGCTATCTCAAATTAATATGCACAAAA
CTGAGCTTTTACTTTTTTAATTTTTAAAAATAATTTTATTTATTTTGGCTGCACTGGCTCTTTGTTGCTG
TGTGAGGGCTTTCTCTAGTTGTGGCGAGTTGGGGCTACTCTTCCTGGTCGTGCACAGGTTTCTCATCGCA
ATGGCTTCTCTTGCTGTGCAGCACAGGCTCTGGGGCACACAGGCTTCTGTAGTATGGCTCACAGGCTCTA
AAGTGCAGGCTCAGTAGTTGTGGGGACTCGGTAGTTGTGGTGCATGGGCTTAGTTGCCCTGTCGCATGTG
GGATCCTCCTGGACTAGGGAGCAAACTCCTACATTGTCCAGCGGATTCTTAAGCACTGGACCACCAGGGA
AGTCCCTGAGCTCTTATGTTTATTCTGCAAGGCTGTATCTTTCCCAGGTTTCCCCATGTCAAGAAATCAC
ACTATCTTCCATCTAGTTACTGAGGCCAAAACGAAAAGGTCATCTTCTCTCCATTCCCGCTCTTCATCAG
CAAGCCATGTCTGCCTCAGGCGTCATTCATTCAAAAGTATACTGAGTGCACAGTATGTGCCAGGCACTCT
TTTAGGCCTTCGGGATATCACTGAATAAAATGAAGGTCTCTGCTTACATTTCTAGCAGAGGAGTTACTGC

TATGTTAAAATATACATCAAGGTAAAAGTATCAGGAAGGCAGGTTTCAGCACTAAATAGACTTCATGAG
GCCTCAGTGATGTGAAATTCAAGCAAATTCTTGAAGACAGTGAGAGCTCTGGCCACAAGGACTTCCAGAA
GAGCATTCTGGCAGAAGGAACAGCTAAGTGTGGTGCCTTGTAGAAGCAGTGTGCTTGCTGGAGAGGCACA
TGGAAAAGGAAAGCAGGCTGTGAACTGGAAAGGGGTTTTTAGATCATTGGGTTTCTACTCTTGACTAAAA
TGGAAATCATTACAAGGTTCTGAGCAGAGGAGTGACCTGACTTGACCTATATTTTAAAAGGATGACTTTG
GGTTCACACTGACGGAGGGGCATGAGATGGGAGAAATGGCAGAAGCAGAGTGACCTCTTGGAGGCGGTGA
AGATTCAAACCAGGGTGATAGTAGTGGAGGTAGTGAAAATGGCTGGTTTCTGAATATATTTGACAAAAAG
AGCCAGCAGGCTTTGTTAATGAACTAGACATCAGGTGTGACCAAAAAAAAAAAAAACAACTAAGGATGAC
CTCAAGACTTC

>PRR3|hg19
TCCTTCCTCGCCGCCGCAGCGCCCTCTCAGTGCCACTGGCTCTCACGTGCGGGTAGCCTACCCTGCATCA
TCCTCTCGCCTCGCTCTCACTGGGACGTGGCTGTATTTCCCCGTTGTCTTCCACCCGGGTCGCCCGGGCG
CAGCGGCCGTGCCCGCCCGCTGACGTCACCTTCCAGCCCCGCCCCCTCCAGGGTCCCGGGCCCTTGAGGC
GGGGGCTGCCCCGGGGGGAGTCAGTTGAGGCGGCGGGAGCTCGGCGGAGGGCGGGCCAGGTGACTGGTCC
GGGCCATGCCGAGGAAGAAGCCTTTCAGCGTGAAGCAGAAGAAAAAGCAGTTGCAGGACAAGCGGGAGCG
GAAGCGAGGTCAGTGCGGGAGCCGGAGGAGGGGGCGGGGCTCGGACTCCCGCACATCTGGAAGGAGGGGT
GTGCCCGCCTCACCTCTGGGAGGCGTGGGAGGGGGTGGCGGCCGCGTGCCGATGCGCGTGGGCGACCGCG
GAGGGGATTCCCTCACCCCAGCCCCCTACCTCTGGAAGGGGTGGGGAAGGATGGAGAGTTGGGGGAGGGG
AATCCCTCCAGCTCCAACGGGGCGCCATCCCCGCAGATCCCTGGAGGAGGAGTGCCTCCCCCCACGCACA
TCCGGGAGGGTCCTGGGCAGAGTAGGTGGGAGGGCCGGGTCTCAAAAGAAGTGCGGTAGTGTGGGGAGGA
GCCAGGCCGACCGGGGAGGGGGTCTTTTGGCCCCTGAGAACTGGCCAGCACTGGCGTCACCGGCTCCTCC
CCGCAGGGCTTCAAGATGGGCTGCGATCCAGTTCCAACAGCCGCAGCGGGAGCCGAGAGCGGCGGGAGGA
GCAAACCGACACCTCGGACGGGGAGTCTGTGACCCATCACATCCGCAGGCTCAACCAGCAACCTTCCCAA
GGGCTGGGCCCGCGAGGCTATGACCCAAATCGGTGAGAGTGGGCGGGGGGCTCTGGCCCAGGTTTCCCCG
CCTACCCGGAAGTCAGAGCGTTGGGGGAAAGCAGGCTGCTGCTGGTGGAGGCTTGAAACTGGAGATGGGG
GAGGGCTGGGATACCACGGTTCTCCACGGGCCGCTAGGGCACAAGGGAAGCTTGTGGAGCGGGCCACAAT
CTTCCCTTCCGCAGAACCTGGAGAGCGTTTGGCTTTTTCTGGACGAAACTTAGGGAAGGCCCGTTGTTGC
ACGATAGAGATTTGGGGGAGGGCCGACTGAGAAGCGAGACTTGAAGTCTGGAGAAAAGAGTTTGTGGCCA
AGGATTGCGATGGATGCTGTGGTCCCTTCCCTGTTAGATACCGGCTGCATTTTGAGCGAGACAGCCGGGA
GGAAGTGGAGAGGAGAAAGAGAGCTGCCCGGGAGCAAGTGCTACAGCCCGTCAGTGCTGAGCTGCTGGAG
CTGGACATCCGAGAGGTCTACCAGCCCGGCTCTGGTGAGTGAGAGCCCAGCGCCTGGATGCTTGGGACAG
GCAGTGGGCTGGGGAAAGGAGTTTGGAAGAGTAAGAGCCGACTGGGAGGAAGAAGGGGTCTGGGAGACAT
GCACGCTTTAGTCTTGAAACTATCCTGAAACTTATTTGGACTTTTTCTTGATTATTCAGTCCTGGACTTT
CCCCGACGTCCCCCTTGGAGCTATGAGATGTCTAAGGAGCAACTGATGAGCCAGGAGGAACGGAGCTTCC
AGGAGTATCTTGGGAAGATTCATGGGGCTTACACCTCTGAGAAACTCAGCTACTTTGAGCACAATCTGGA
GGTGAGACTAGAGTGGGGGCAGGAGTGGGGCATAGTCACCCATGGCCAAATTGGTTTGAGTCAGGCAGGA
GAGTGACTCTGGGCTCAAGTTCTCTTTCACTGATTTTGTCTTTTCAGACATGGAGGCAGCTGTGGCGAGT
GTTAGAGATGTCTGATATTGTTTTGCTGATTACTGATATCCGACATCCAGTGAGTCCTGGGACGAGGGTG
GGCAAGGGGCAGGAGGGAGGGAGGTCCTGGTGGGGTAAAGGGCAGAGGCAGTAGTTGGGAGACAGACAGG
TAATCCCTTCCTTTCCAGTCTCCTCTACCCTGCTGAAGTCTGCAGTTGGGAACTCGGTTTTTATTCATCA
GTGTCAGAGCCTGTCACTCCTCCTCTGGCCAACCACCCCGCCTGTTCTTTAACCCTTTGATGGAAAACTG
TTCCTGTTTCCTGGCACACCAGGGCTAATAGGAAGTCTCAGTGTGGAGCTCCAGGGAAGTCTGTGAAGTG
GGCTTCCCTGCCATATGAGTCTCTACAGAACTCAGCCTCACTTGGGATGCTAGGGTAGTGGTTAAGGCTC
TAGACAAGCTGCTGGACGGCTCTCCTGTGATCTGACTGTGCTGCTTCACTGTTGTTCTCCAGGGGGAGAA
AGGGAGGTCTGAAGTCTGTGTGGATATTTAGGTTGGAGATCATTAACATGTACACATACCCCCTACGTAG
TCGGAGACATACGTGTGCATTCACACACTCACTTAAACAGCTACGCATGCTCAGTGCAAACCTCATACGT
GCATGCCGGCACATAAGCTTGTGTACACATGCAGACAGAAGTGTGTGTAAAACTAGATATTGATTTTTAT
CCCAGCCCTCATCCCTGTGAGTGATTCTAGCATTTATCCCCCGTTGCTAGGTCCGCTTCCTTGTTTTTAG
CTATTTACCATCCCTGATCGGGCCCACAGGGAATGCCCAGAGACACACATACAGGTGATGCTCACCGCTG
CTTTGTCTCCCTAGTTCAGTTTGGCTGATCTGTCCTTGTTGCCCCAGAATCTGCTACTACCAGGTTCTTC
TCTCCAAGCTGTGCTTGGTGAGTAGGGCGCTGCAGGCAGAGAACTGTGGGTTGACCTTGCATCACTCCCT
CCTCTTATATCCTGTTCCAGGTTGTGAATTTCCCACCAGCACTTTACGAGTACGTGACTGGAGAGCTTGG

GCTGGCCCTGGTGCTTGTCCTGAACAAGGTGGATCTGGCCCCCCCAGCTCTCGTGGTTGCCTGGAAGCAT
TATTTCCATCAACACTATCCCCAACTCCACATTGTTCTTTTCACCTCTTTCCCTCGGGATCCCCGCACCC
CACAGGACCCTAGCAGCGGTGAGTGGGCAGTGAGGAGGGCAGTGTGGGAGATGTGGCGGGGGAGGGGGGG
CAGGGGACAAAAGGGAGGACCTGATCTGGGGTCTGAGGGTGGTGGGAGAGAGGGGGTACCTGAGCCTCAT
GGGGTGAGCTCATGTTTTTGCACCCTCTGATCTCAGTCTTGAAGAAGAGTCGGAGGCGGGGAAGAGGATG
GACTCGGGCTC

>RPP21|hg19
GCGAGCTCTCCACACGCGACCGTCCACAGGCCCCGCCCCCAGGATAGCCACGCCCCCGGCGTCCGCGCAA
GACCTCCTGGGAATTGTGGTTCCGGGAGCCATGTGCGCTCCTTTCTTTCTAGCCTTCCTTTCTCTGGCGC
CACCCTGTGGCCTCACAGACCCAACTATTTGCAAATCCCCTCCGCCCCTGCAGAGTCCAGGGGCCTCCCC
CTAGTCTTTCTCAAGCCAATAATTATATTAGGCAGAGACTGGAGAAGTTAGTTGAGCAGAAGTCTTAAAC
CCCTAAACATCGTCAGGACAGAAGACTCTTCTTTAGGCAGATAGGTTGGGAGATGGTTGCTGCTGCTAAG
TCGCTTCAGTCGTGTCCGACTCTGTGTGACCCCATAGACGGCAGCCCACCAGGCTTCCCCATCCCTGGGA
TTCTCCAGGCAAGAACACTGGAGTGGGTTGCCATTTCCTTCTCCAAAGCATGAAAGTGAAAAGTGAAAGT
GAAGTCGCTCAGTCGTGTTCAACTCTTCGTGACCCCATGGACTGCAGCCTAAGAGGCTCCTCCGTCCATG
GGATTTTCCAGGCAAGAATACTGGAGTGGGGTGCTATTGCCTTCTCCAATGGGAGATGGTTAGGTGCAGT
TTATACCCAGCTAGACTTTCTGCCCTATGCTTCTGAAGGCAAAGGCAGATGCCTGGAAGACCTACAGACA
GAAGCAGCAGGTCTCCCTTTTTTTTTAAAAAAAGGCACTGGTGCCAGTGGGAGTGATGCTGGAAAGGATC
ATGAGGGTAGTGGAGGGAGGGAAGAGGGCTCCTGGCCCTTAGAAAGCACAGCTTCCTCCCAATCTTTTCC
ACTTACCAGATGCAGTGAGCACAGAAACCTTTCAACAGCATCCCCTCCCCCTGAAGAAACATCCCCTGTA
GTACTTCACATCCCTCCCTTCGATCTCCGCCTCTCCTTTGCTGTTTGACCTCCTGCTTCTGCTTCATAAT
TAATACCACTCCTTCCTGAGGTTAGAAGTAGAGGGGCAGAATATGCATAAACCCTGAGATGGGGAGAGGG
TAGCGATGTCAGCAGAGGATGAGAATTCTCTCCTCTATCAACTACCGCCCCCCACACCTTGCCCCAATCA
GGACTCCAGTTTGAAAGAAAAGGCTGAAATTTCCTCTCAGATTACTTTCACAAGGGCCTCCAGACTCAAA
TAAGGCATATGTTTAAAAGGCAGATATGCCCAGGCCCACTCCAGACCTCAATCAATCACTGAGGGCAAGC
ACCAGGAATATGCTTTTTTTTTTTTTTTTTTGAGGCCACACTACAAGGCTTGTGGGATCTAAAGTTCCCC
CACCAGGGATCAAACCCAGGCCCCCAACAGTGAAAATGTGGAGTGCTGACACTGGACTGCCAGGGAACTC
CAAGGAACATGCATTTTTAACATTCAAGTTTCTAACACATCAACAGAGGTTACTGTCAAATTACCACAGG
ACACAAAGATTACTTCAGTGGATCCTTTGTTCCATATTATGTTTTACCAATCAAAGCATCTTGCCACAAA
GAACCACAGTCAAGGCACAAAGGTAAGAGGAGAGTCTGGACGAAGTCCTGTTGAGGTGGTAGGAGGAGCT
GAAATGCCCTCAATAAACTGCAGTTTCTTGTCCCAGTCTTCTGTGAACCCCTGATTAGCCAGTAGCAGAT
AATCCAGTCATTTTAGTTTGGTTTGGCATGCATTGCGTTACCTTTTCTGTAACATATGTACCCTATAGGT
TGTTTATTTTGCCCTAACATTAAAATGATTTACATTCTCTGATAGGAGAAATGGCAAAACATGAAGGATG
GCAACAAACAAACAAAAAACCGGCTCTTATGAACACTAGTTTCAGGCCAGATGCACACAGGCCAAAGGAA
GTTGCCTGTCTGGCTCTCCCCTAAACATGCTACCCTCTTCTACCCTGTCCCCAGGGTAAGGAAGACACCA
GTTTCTTTCTCCCTGTCTGGCTTGGATACCTTAGTCAGGTAACAAGGCTGACAACTTGGGAGCAACTACT
TGGAGCCATTCTTTAAGGCCAGGGCTTTGGATAGGAATGCTTTTCTCTTTCCTCTAATACCTGACTCTCC
TTTCCAAGTTCCCTTGGGGAAGCAGTGAGAGGGTAGACATGTCTTGCATGATCTCTTGCATCTTGCCCCT
CCAGATGAGCAAGGTAACAGCTCTTGGAAACAAACAACTACAAGAAAGCAGGCGACAAGCCAGGCCTGCT
GAAGATGGAGGCAGGGACCTGGGTTCTCTTTCTTCCTACTCCCTCCTCCTCACATTCCTACCTCTGGCCA
ATCCTACTCATAATGGAACCGAAGAAACCCTTCTCTCTTTCATTAGTCTCTAGGAATCGGGTCCTTTAGC
CCTCTTTCCTGACCTTCGTTCTAAACTTCTTGATGCCCAATCTCTGCCCTGAAAATAACTCCTCAACTTC
TAGACCCATGAGAACAACTGTCATTCACCTTTATTTCAATTAGAGGTACAAAGACCCCTGAGATCTTCCT
AAAAAAAAAACAAGTCTATTCTGGGTCAAAGAACCCCCTGAAAGGCTGTACAGGGGTTTCTGTTTCCTTC
TCTGGTTTGAGCAAGTCAGACCCTTCCACAGACTTGTTGTTTCTGGAAAAGTCTCTCCTTCCTTCCCAAC
CGGTCCTTGTGCAATAGCAAACCCGATGGACTTCAGGGGAGACCACCCCCTGAGGACCAGCTCTCCACCC
TGTCAGGAGGGAGACACTGCAGTGCTCTCGACCTAGTCCAGTCCTACCTACTCCTCCACATAAACCATTC
GGGAAACCAAGGTGTTCTCCCACATGGTTCAGAACCAGAACCAGGCAGACACGAAGACATTCCAGCAGGT
CACAGGAAGGAAGGCCTGTAGATCTTGACCCTGCCTCACCCTGATGATTCCCATGCCCCGACCTGTCACT
CCCAATCTGTTGGGGGCCTGATGGTGAGTGGTGCAGCATTCTTCCGACCCGCCCGGATGCCTGGATAGAA
GAGGGGCCAAAGTTTCTCAGTAAAAGTATCAGTGAAGGTGTAGATATGAGAGCGGTCTGTGACGTTGTAA
AAAGACAGTGTGCCGGCCTCATAGTCTAGGAATATGCCCACCCGCTTGGGTTTCACCTTGATGTGCAAAG

GGGTAAAAGGCGTGGTGGTGGCTGCGTACTTGTCCCCGTTCCATAGCCGCACCCGCCAGTAGCCAGTCTC
AGGAAGCGGGGTCAGTTCACCCTTTCGGCTCACGGAGTCCCGGCACACGCCCACTGCCCAGTGGGTCTTG
TCGCCCACCTC

>TRIM39|hg19
CGGCAGCGGGACGGGGTAACGGCGGGGTAGCCAAGCAACGCCGGACGCGCTGACGTCGCCGGGGCGGCAA
AACGTCCGCCCGGGCCTGAAACGGGCGGAGGCAGCTGTGGTTACGTGCGGGGGGCCGGTGACGCAGTCGC
GGGGCGCTAGGACGCGGCGTGCGGAGCCGGCGGGCAGGGGCAGGCCCCAGGGTCCCGGCGCGGCGGCGGG
ACAGCACCTACCTTCCCGGCTTCGGGTCCGGGAGTGCGGGGCGCGGAGCCGCCGGAGCGCTGCCTCCCTC
CTTCCTCCTCCCCCCGCCCCCGCCCCGCAGCGCGACACACAATACTCGCCGGAAGCGGAAGCCTCGCCGA
GAGGCTCGTGTCAGTGGAAGCTGGGGTGTAGGCGGGCGACAGCGGGAGGGTCTAGCGGCGCCTGCCGAAC
AAGAAGGCGGGGGGCCGGAGGTGAGCACCAGCTGGAGGAGTCAGGGGCAAGAAGAGACCCGGAGGGGCAG
GTGGCTGGAATGCGGTCGTCCAGGTGACCCGAGGAATCCGCTGGAGAAAGACAGCCGCGCATGCGCGTGC
CGGAGCGCTGAAGCCCATTGCCCTCAGGGCGCCTACGGGCGCCGGGGCAAGCGGTACTGTCCCGCCCAGG
GTCCGCGCCCGGACTCTGGCCTGGCTCGGTTGTTCCCAGGTGGCACTGGGAGCCGTCGAGGGCGGAGCAG
GGGCGGGTTGCGGACTTTGAGCTACACTTCTCCTCTCTCCTGGCCAGTCTCGGGAGAAGGTGGCTCGCCG
AGTCGGCGGCCAATAATGTGGCGGTCCTGCTGGTCTCAGCTGGCACGTCATTTCCGACTAGTTGAGTTTT
AGGCTTTTAACATTCAAGGATTAGCTCAGTCCTGCTTTGGGCTTCGGATATTCCTTACCTTGTCCCTTCT
AGGCGGGCTTTATGGAAATTACCTGCTGTCTCCGCTGGAGTGGGAGCCTCGAAAGAGCTGGTACTGCGAC
TCTGCTGCTCACCTTCGCATCCTCGGCTCCTTGCTCGGGGCCCGACTGCAGTGAGCGCGCCTTGAACTCG
GGTTCTGAATGCGAGAGAATCCTGTCTTTTTCCAGTTTGGGAGCTTTGACTTGAGTTTCTCCCCAAGAGT
AGATAGTGCTTGTATGGAGACTCCAGACTCCTGCGTTATTACCTCCTTACTTTCCTCTCCTGTTGCTGGA
AGCTTCGCAGGTCTGTGACTTTGGAGGACGAGGGTGAGTTGAGGCTGGAAGAGTCTGCTGATGGAGGTGT
GATGAGACTGGGAGGAGGTGCGGAGTTGATGGAGCTGCGGAGGGTGGGTGCTGGGTAAATGGGTGATGCG
GCTGGGTAGGAGTTAGTGCTCTAAGCGCAGACACGGCTGGTAGAAAAGGCAGCCCACCTCCCCTCTTCCC
CCAATATTTGGCATACAGAATGCTGACTGAAAGAAATAAGCAAATTTTGGTGTCCCCTTCGAGGGGAAAT
GGCCATATGGCCCAGCTTGTTCCTGTGTAAGTTGTGATGCTGTTTACAAACACTGTGCAGTGTTTAACCT
CAGAGCATCCTGGGAGCCAGGAACTTATTTACTTGCAGAAAATATCTAATATGGGAGGTATGAAGATTTA
GAGTCAGACATGCTAAGTACAGTTGATCTGCTTTTTCTTCACTGTTCTTTGCTTTTTGTTCACTTTTCTT
TTTTCAGTAGCCAGTGGTTGAAGATTAAATTCGATAAAATTACATTAACTCTTCTCCACCCCATTGTTAT
TTGATTGTTAGGAATTTTAGAAGCAGTACGTTTTCTATTTTACGGACTGCCTCAATTCGAATGTAGTGAA
AGCCACTAACTAGGTGGTCAGCCTCTTTAGCCTTGCTTTATGGGGAGAGTGGTGAGGTTTGGTTCTGGTT
TATGATTTGTAACTTTTAAGGTGTTGGCTAGCACTTTACTCTGGCTTTCTGTTTGTAAAAACAGATGTGG
TACTAAGGAAATTATCAGGAGCAAGGACATTGCCTAGGAGTATGAAGAATGTGTTGGCCTCGAGTAATAG
ATTTTTAAGGGGACTTCATGCCCAATAGGGCTTCCCTGGGGGCTCAGAGGTTAAAGCGTCTGCCTGCAAT
GCGGGAGACCCAGGTTGGATCCCTGGATTGGGAAGATCCCCTGGAGAAGGAAATGGCAACCCACTCCAGT
ATTCTTGCCTGGAGAATTCCATGGAGGGAGGAGCCTGGTAGGCTACAATCCACGGGTTGCAAAGAGTCGG
GCATGACTGAGCTACTTAACTTTCATGCCAAGTACGGAAGAAAAGGGAGGAAGAATAGGTAAAACTATAA
AATTATAGTTTAGAAGTTTATTATACTATAAAAATACAGTTTAGAGGGCATTATTGTCATAGGCCAGGGG
TAGATATTGCCCATACTGTCTTTGTGGTATGAAAATTGCATCAGATTATACTAAATGGTGAGCATTACTG
AAGTTTGAATTTCATATAAATTCTTCACTTGTTTTGAAAATTTATTCTCTTTTTTATTTTACCCCCAATC
ATTTAAAAATATAAACACATTTCTTAGCTTGTGGGCTGTACAAACACAGGTGGCAGACCATAGTTTGCTG
AGCCCTTACATAGAACAATAAAGAAGTACTCGGTATTTTTCAAGAGAGAATAATTGAGAAAGAGGGTGAT
CCCAGTGCTATGATCTCTTATTTTCATCTGTCTATATGATAACACACAAAATAATAAAGAAAATTTGAGA
TCTTAACACAAATTTGTATTTTTTATGACCTGTGAAACTTGATCACGTGAGAATTATGAATATACTAATA
AGATACAAGTACATTCTTTAAACAAGAAAAAGTATTCCTTGTCTAAAAAAGGGATTGTAGTTCAGGAGAG
GTGGAATAACATCGAGTTGTGGTGTTGATTTAGACCAAAGGATTTCCAGCACAGGAATATGCTGGAATAA
TAGTAGTAAAATATTATTACTATTATGCAAAAGTAATTACTATATAGTAAATATTACTATATTACTATAA
TTTATGCAAAAATAAATAATAGTAATGTGAAGGATAATTCAAAAATTTTAAATATACACTTTAATATTGT
TTTTATATCAGGGAACGATATATCTGCTATATCTTTTATATAGAGGATCTTGTGGAACTGCTGAGTCATA
TAGAGGAAAAGCAGGTATTAGCACTGCAGGTTGGAGAAGGAAATGGCAACCCACTCCAGTGTTCTTGCCT
AGAGAATCCCAGGGACAGGGGAGCCTGGTGGGCTGCCGTCTGTGGGGTCGCACAGAGTCGGACACGACTG
AAGCGACAGCA

>ENSBTAG00000069337|hg19
GAAAAATGGCAACCTACTCCAGTATTCTTGCCTGGAGAATCCCGTGGGTGGAGGAGCCTGGTGGGCTGCC
GTCTTTGGGGTCGCAGAGTCGAACACGACTGAAGCGACTTAGCAGCAGCAGCAGTGTAAACAAGAAAGAA
GTTGGCAGATCTCTGAACCAGGTCAGAAGTCATAATTCTGTAGTCTTGCTCAGAATAGATGTTCCTGGTT
TCCCACTCCAGTATTCTTGCCTGGAGAATCCCCCAGGATTTTGGCGGGCTACAGTCTATGCGCGGTCGCA
AGGAGTCGGACACAACTGAGCGACTGAACTAAACTGAACTGAACAGTCTATGCGGTCGTTCAGGAGGGAG
CGACTAACACTTTCGCTTTTACTTTTAAAGTCTTTGGAAGGGCAAAGGCACAGCCAGAAGCAAAAATTTG
GTATGGATGGTATTCAAGGTTTTAGATCATCTTTCTTTACAATTTAGAGTGTGTTTATTTATTTACAAAG
TATGTGCATATTCCATTATAGCAGTACATTATGTTGAGTATACGAATTTACAGAAACAAATCCAAAAAGA
TGAGATAAAATATTTTAAATAACTTAAAAAAAATTGATCACAAGCTTGATTAATGACAGAACTGAAAGAA
AATAACGTAGCACACACATTCATCTTTTATTCATATGGGGAAAGGGCTTCACTGAAGTGTTTGTTTAAAT
ATGGCTCATCCAGGAAAACCCCATAGGGACAGTATGCAAAACTCTCACCACCCACTAAGCTACAATTTTT
GCATTTTCCTACATGCAGGTTATTTGCAAAACCATTGTTCCCAGCCACAGGTCTCCTAGCACAGTTTCTC
AGCAGCTATTATTGCGCTCCAGACTCTAGAAGTGTACCAAGAAAGCCAGAAGGAAAGCCAGACTCATGAC
TTAATTACTGGGTGAAATGTTTCCAGACCAACAAGTAACAACTCGTGGAAAATGAAACAAATTCTTGACA
TATCTGAAACATATCTGGCAGGGTGAATTTTTTTAAGTCTTACCTATTTATGAGCAAGAGAGGAGGAGAA
ATGGGTTCTTTTTTTTTGAGAAATGGGTTCTAAATAAAGCTTTGCTACTAAGCTTCAGTTTTCTCACCTC
TAACATGGAATTTAAGCAGCTACTTCATACAGTCATCAAAAGAATTAAAGAAATAACGGCAGACCACAGA
GGAAATGCTCCAGCACAATGATAATTTTTAGGAAACAGTTTCTGTGCAGGCGTTAAGTGGTGTGAGTGCT
TTAATAGGAGTACAGTGTAAACATTTCCATATTTATACATTTCCTATCTGCCTGAATGGAAAGTCATCCA
GCTATGGCTGCTTGGCTGAGCCTTAGAATAAAGGGGAGTTTGTGCTCAGTGTCTTCCTTTTGCTCTTGTT
TTTGTGGTTTTTCCCTCTCCACAATCACTCCCTGGAGTTCAGTGTCTCCGAACATCTAACCACAGCCTTG
CTCCCTGCATAGCCCCTGTTAGGTGCAGGGTCACAGTAGGGATCAGCAGAGAGCAGACAGGGACTGCCGG
CTTGCATGTCCACTGGGTCCTCAGGAATGACCTCTGACCTCAGTAGGTGCTTCTCAATGGAGTCTAGATC
TGAGTGGGGTCAGAGTCAGTTCACAGTGAGAGGGACTTTATTCATCAGAGGAGGCGCCCATGTCAAGGGG
TAGAAGTGCAGAGGTTCTTCGGAAAAAAGAACAATGCTGTTAAAAACAGAAACGCTATGAATAATTTACA
GAAGAATGAATTCAATTGGCAGATAAACATGGAATTATGTTCAATATAACTTCATAAATTAATGTATAAA
CTAAAATGTATGTATTTTAACTGCCATTTATAGCAGTGTGACTAGACCTAGAAAATATTATATTTAGTGA
AATGTCAGACAAAAAATACTATTTGAGATTACTTATATTTGGAATTGAAAAATAATACAAATGAATGTACC
TACAAAACGGAAACAGACTCACAGACATAGAAATCAAATTTGGGATTACAAAGAGGAGAGAACAGAGGAG
GGACAAAGTAGGGATACGGGATTAGTAGATACAAACTAAGTAAAGTAGATAAGCACCAAGGATTTGTTGT
ACAGCACAGGAAATTATACGCATGATCTTTAATCTGTTATGGAATACAATTTTTCAGAATACTGAATCAC
TATGCTGTACACCTGAAGCCAACACAATGTTGTAAAGCAGCTGTACTTCAGTTTTTAAAAATTTGCACTT
CGCTCTCATCCCCCCACCCTGGGGCCATGGAAAAACTGTCTTCCATGAATCTGGTTCCAAAAAGGTTCAG
GACTACTGCCCTAGCTGGTAATGTGGGTAGGCCTTATCTGATCAGTTGAAGGCCTTAAGGGGAAGACAGG
TCTGAGGAAGAGTGAATTCTACTTTCAGACTGCCTTTGGACATAAGCTGCAATATCGTGTCTTCCTTAGG
TATCCAACATGCAGCTCTGCCTTGCAGATTTGGGCTTGCCAGCTAATTCCTTAAAGTAAATCAATCTTAC
CCTGTATATATTCACTTATGCAGAGGAGTGTAATTGTCTTTTCATTGTTTTATGTTGTGCGCCATGTCTC
CTATTAGACACACGCTCATTCCCTAGATCCTCACACAGTAAATTCTGGAGCTCTCTCTGACCCACTGTTG
CTTGTTCTCACCTCAGGATCACACTCTCTGCAGTATTTCTACTCAGTTGTGTCTGAACCGGGTCCAGGAG
TCCCTTCATTTATGGCTTTTGGCTTCGTGGACAACCAGCCCTTCATTCGCTATGACAGTGAAGAGATGAA
GGCAAAGTCTTGTGTTCATTGGTTGAGGGAGGAGCCAAGTTACTTTGATGATGAGACCAAGATCTTCACA
AGTCGGATGAAGATTTTCCACTTAAATCTGAGGAATGTACAGCAATATTACAACCAAACCAAGGAGGATG
GTCTGAACAGAGCGCTGGCACAAAAGCAAACAAGTAGGTGCCTCAGAGAAAGCAGAGGGAAGTGGTGCTT
GTCACTTCGTACCAATCCCCAGGGGTGAAAGGCTCTTGACAAGTGTTCCCATAGGGTCAGGCTGGGGCAG
AAGAACATGTACAAGTTATCACTTGAGAATGAAAGCTGAGCTGGTCTGGTCACAATGGTGTAGGGTCTGT
GTGACGAGAATTAATAATTAATAGATTAATTAAGCAGTTTTACTGGGTCATGGATATATGCTGAACATCT
TTTGAGTGCTAGAAATGGGGACCCAGAAGAGTATAAACTGATGTTTGTCTTCAAAGAGCTTATAATTCAT
ATGGGAAGGTA

>ENSBTAG00000002069|hg19
CTGGCGGGTCGGCGGAGACTTTATAACTGGATCCACGGAGACGCTGATTGGTTTTTCTAGGAACTGAACA

CCCAGTGGCAGTGAGAGCTGGGGCCGCATCATGAGTGTCTAGGCAGGAGGGCTCGACACAGGTTGTGAGA
GAGAAAGTGAAACTCGGGGAGAAGGGGAATCCCCAACCTGGAGTGTCCCAGCGCCAGACACCGCCCTTGA
CCTTGAGATCCTGAGCGGCTCGCCCACCAGGGACCTGGGACTTTGCCCTGATCCCTCCCCTCTTGCTAGG
TAGAATATTCAAACTACTGAATAATTGCACTCATCTCACATGCTAACAAAGTAATGCTCAAAATTCTCCA
AACCAGGCTTCAACAGTACTTGAATAGTGAACTTTCAGACTTTCAAGCCGGATTTAGAAAAGCCAGAGGA
GCCAGAGATCAAAGTGCCAACATCCCCTGAATTATCGAAAAAGCAAAAGAGAAAACATCTACTTCTGCTT
TAATGACTATGCCAAAGCCTTTGTCTGTGTGGATCATAACAAACTGAAAAATTCTTCAAGAGATGGGAAT
ACCAGACCATCTGACCTGCCTCCTGAGAAATTTGTATGCAGGTCAAGAAGCAAGAGTTAGAACTGGACAT
GGAAAAACAGACTGGTTCCAAATCTGGAAAGGAGTATGTCAAGGCTGTATATTGTCACCCTGATTATTTA
ACTTACATGCAGCGTACCTCATGAGAAATGCTGGACTGGATGAAGCACAAGCTGGAATCAAGATTGCTGG
GAGAAATATCAATAACCTCAGATATGCAGAGGACAACTACCCTTATGGCAGAAAGTGAACTAAAGAGCCT
CTTGATGAAAGTGAAAGAAGAGAGTTAAAAAGTTGGCTTAAAACTGAACATTCAGAAAACTAAGATCATG
GCATCTGGTTCCATCTCTTCATGTCAAATAGATGAGGTAACAATGGAAACAGTGAGAGATTAATTTTTTG
GCTCCAAAATCACTGCAGATGGTGATTGCAGCCATGAAATTAAAAGACACTTTCTCCTTGGAAGAAAAGT
TATGACCAACCTAGATAGCATATTCCAAAGCAGAGACATTACTTTGCCAACAAAGGGCCATCTAGTCAAG
GCTATGGTTTTTCCAGTAGTCATGTATGGATGTGAGAGTTGGACTATAAAGAAAGCTGAGCAACAAAGGA
CTGATGCTTTTGAACTGTGGTGTTGGAGAAGACTCTTGAGGGTCCCTTGGACTGCAAGGAGATCCAGCCA
GTCAATCTGAAAGGAAATTAGTCCGGAATACTCATTGGAAGGATTGATGCTGAAGCTGAAACTCCAAAAC
TTCGGCCACCTGGTGTGGAGAACTGACTCATTGGAAAAGACCCTGATGCTGGGCAAGATTGAAGGCAGGA
GGAGAAAAGGATGACAAAGGATAAGATGATTGGATGGCATCACCGACTTGATGGACATGAGTCTGAGCAA
GCTCCAGGAGATGGTGATGGACAGGGAAGCCTGACGTGCTGCAGTCCATGGGGTCACAAAGAGTCAGACA
CGAGTCATGATTGAACTGAAGTGAACTCCCCTCCTGCACGGAGAACTCTTTGTCACACTGTCTCCCAGCT
TTGGGGCTGTTTGCAATCTTGATTAAAATACCTACACAATCTTACAAATTAGTGAGGACCCCAAGGATAG
TATTTTGTGACCACTTCTATGCATATTTTCCACACTACGAATAATGCACTTTAAAGATTTTTTTTTAGTT
TATTTAGAATAATGTTAATAACACATTCATTTAATATGAAGAACTATTTCCCCAAATAAACAGGGTAGTA
AGAAGCATAGGGCTTTCTACATTTTTTTCCAATATGTTTCTTGTCTTTTTCACTAGAAGACCACTGGATT
TTTAGGTTTGCGTCTGCACTGAATCTGTTATTTTGATTGAAGTCTGCTGCTGCTGCTAAGTCGCTTCAGT
CCTGTCCGACTCTGTGTGACCCCATAGACAGAAGCCCACCAGGCTCCCCTGTCCCTGGGATTCTCCAGGC
AAGAACACTGGAGTGGGTTGCCATTTCCTTCTCCAATGCATGAAAGTGAAAAGTGAAAGTGAAGTCGCTC
AGTCGTGTCTGACTCTTAGCGACCCCATGGACTGCAGCCTACCAGGCTCCTCCGTCCATGGGATTTTCCA
GGCAAGAGTACTGGAGTGGGGTGGTGAAAAAGCCCACACACAGGTAGGAGTAGTATTTTTAAATAATGTT
TTCAGATAATTGTGGATGTTCATCTTGGATACAAAAGTAAAACTATACAAATTGTAGTTTCTCAAAGTTT
TCTTCCACAGTGGACCTGAAACAATATCATTGACTATTTTATATTCTGTTATTTTAAAATCCATAAGCCT
ATTTTGCTGATTGAACATCTGATGTAGTAATGTAAGATTTTTATAAAGTAATACATTGTTTCACTAAATT
ATACAGATCTTTCTGCATCATTCTTTCTTTCAATAAGTCTTTTTCCATGAAAAAGAGGGTAGTTCAGCTC
ACACAGAGTTTTCCCTTGGGCCGTAGGACTTTGAAATGCAGTAGAAGTGCTCGATGTGTACTTTCCATGT
CATCACAGAAGATACTTTTAAAATGTGTATTTAAGAATGATGATTAGGAAACATAAGCATTATTATTTAT
TTTATTTTAAATAATATATTTTATTGAAGGGTATTTGATTTATAATGTTAATTTCTGCTGTACAGCAAAG
TGATTCACTTATACGTGTGTATATATATATATATATTCTTTTTCATATTTTTTTCCATTTTGGTTTATTAAG
GACAGATAAGAATTTTTAATGTGTCATTCTGGATGTTTTACAGCAAACCTGATTATCAGTTTTCTTTTTT
TCTACAAGAAAATTTTGCTGAAAATAATTACAAGTGCAGCTTGCTACCATAGTCTCCATTACTGCTAAGC
TATACATCAAAGCAAATGTCAACATAGTGAGAAGTTAAATAATATCTGTGAAAGTAGTTTTCACCTCTGG
GATCTCATGAAAGGGTCTCAGTGACATCAAGGATTCCACAGACCACACTCTGAGAACTGCTGTTCTGAAT
GAATCAGGGCATCTCTCAACTGATCCCTGCCTTTGCCTCCTTTTCATTTTGGAAAATCCTTTCTCCTGAG
CTGGACTCCCTGCCTCCCAAAACTTAACTCAGGGTCCTGCTCCTGGGCTCCTCTAGGAGAGAACTCACCC
CCTAGAAAGTGATGCCAGAGAGTGTCCTCCATCTGGGAAGGGAGGTCAGGGGACAGGTGTTTCCCCTTGG
GAACTGGGAAC

>ENSBTAG00000051089|hg19
GGTAGTTGGAGGGCCTATGTGCTGACCGCGGTGACTGTCCTGACGGCCCAGGGGAGATTGTGCCTGGGAC
ACAGTAACAACTGCAGAGGAGTCGCTGCACAGCAGTGGAGCGAGCGGCCCGGACACTGGGGCTGGTCAGC
TTCCCTGCCTGGCGGCGGGGTGGACTTGCATGAGCTTCTAGGAAATCTCCCCACCAAGGATCCTGGGACC
CTGAATGCCCGTCCGTTTTTCAGGCTCTGTGGTTTCTGAATCCAGCACCAAAGAGGGAAACAGTCCAGGC

CCCTAAGCTCTTGGAATCTCAGTTTTCCCTTCTGTCAAATGGGGACAAAAATGCCTGCCTGATTTAAAGA
TAGTTTATGTAAAGCGCCTTACTCCTCGTAGACACCTCAGAAACGGTAATTAATTTTTGTAAATAGACCC
CTGAGACTATCAGTTTGCTGAGATCCTGCCTGATCCAGGAAGCAAATGTAGGCCAGGTTGGGTCAGGGAA
GTGCAGTTGCTCAGTAGTGTACAACTCTTTCTGATTCCATTGATTGTAGTCTACCAGGGAATCGAACCCA
GATCTCCCGCATTGCGGGCAGACGCTTTACCCTCTGAGCCTCCCGGGAAGCTTTAGGGTCATGGAGTCCT
ATTTAATGTTGAGCACCTGGCAAACGCCACTTATATGCCAAATGCCCTCCCGGTGGGGCGGAAATCGGGA
GGCGGGGAGGGTTTCACCCATTGCAGAGTCCTACAAAGGGGTCTCATGAGGCCTTAAGTTTCGCCTTCTT
CCACCCTGCAGAGCAGGTGCGCGTACCACGCGCCAAACTCAATACAGCAGCCGCTAAACCTAGGCTTTGC
ACCATTTCAGTTTCGGTTTCTCTGACCCTGCAGTCCCACCCTCCGCGACGGCCTGGGGATTTGCCCAGTG
ATCAGTGACGTAAGGGAGCTAAGGCTCCCATTGGGAGGCGGCGTTCAGCAGCGACCAATCCGGGTGCGGG
TCCGGGGGGAAATCTCGGTGGAGGGCTCCGGAAAGCCGGGAGATTACCGGGCGGGGACGCGCGGTCGCCC
TGGGTGCAGGCGGTGGAGCTCGACCCGGAACTGACCGCTGAGGGAAAGATCGACCCCAGCTCGGGATTCG
CGGCCCGGAGCTGGGGACCGGTGAGTAGGGGGTGCCCGGTGAGAAATGCTGCAGAGCAGCAGTCACACAC
GCGCCCCGGGGTCCGAGCGGCCGAACGTGGCCGCGCGGGCAGAATGGGGCCGGGCTGTCTGGCGCGCTCG
GAAGAAGGGTGAAGTTCGCGCCGTCCTCCCTGCCGGCCTAATCCTCCTGCTTGGCTTGGGCCTCCATTCG
CTCTCTCCTCGTGGGTCTTCCTCGCTTTCTCGTGTCCAGCTCGGGCACCCATCCATTCTTGTCTCCAGAT
GACGGTCTCGTTTCTTGGAGCCGTGTAAACTGCCAGTCTGGCCCCAGGACCAAAGTCATGGTGTTTTTTG
TTTTGTTTTGTTTTGTTTTTTAATTGGTCAGAATAAACCGGTTTAGCCACGTATACCTGGAGCTTTGCAT
TCACTCCTTCCTCTGGCCCATTAGTTCAGCATTTTCCCTGCTGCTTTTCACCCTTAGAAAAGTGAGTAAA
TTTATCTTTGTGGAGCAAAGGAGAAGCAAACACCTCCAAATTCCCAGTACATTATAGAGTTTGGCACTAC
GACGGTTTGTGCGCGCTTCCCGAGAATGATATTGGTTCATTCAACAACAAATATCAATGTCAGGGACCCT
GAGCTAAAAATCAAGATTCTTAGAGTTACCCCTGCCCCAAGTTTAAAAGAATCAACTGTTTAAAAAATTG
ACTTTATTTTGTACCTCAAATTGTAATTCCGTTTTTTTTTAATAGACTTTATTTTTTAGAACAGTTTTAG
ATTTATAGAAAAATTGGGAAGCTAGTACAGAGTTCCCAGTACCCCCAAACCTTTCCCCCATTCATTTATC
AATATCTTACATTAATATGGAACATTTCTTAATGATTAGTGAACCTGTACTGATACATTATTATTGGCCA
ACATACATACAGAATTCACATTTTCATAGTTTTTACCTAAGTACTTTTTTTGTCTCATCATTCTACCCAA
GTTACCATATTCTAATTAGTTCTCATTATGCTCCCCTTGGCTGTGGCAGTTTCTCAAACTTTTTTTATAA
TCTTGAGAGTTTGGAAAATACTCCTTATATATATTATATTATGTTATATTATAGGCTGCCCCTAAGACTG
GCTTACGGGTTCTTGGGAGGAAGACCACAGAGGTAGATTGCCATTTTCATCACTTCACATCAAGGGAACA
TGCTTTAGGACTTGATGCTGGTCTTGATTACCTGGCTGAGGCAGTGCTTGTCAGGCTTCTCCATTGTAAA
CTTTCTCCTCCCCCCTCCCCCACTCCTCCCTGCCATACTGTACTCTTTGGAGAGTAGTCAGACCCTGTGC
AGAGGAGTGGGGAGTGTGCTCCACCCTCCTTAAGGGCAACTCTGTACCTGTGTTATTTGCAATTCTTCTG
CATGAGAGATTTGTCTCTCTTGCCTTATTTATTAAGTTATTCATGTATTTTTATCAGTGTGGGCTCATGG
ATATTTATTTTATACTCAGGGTTATAATTCAGTACTACTTCATTTTGTTGCAACTTTAGCCACTGGGAGC
TCTTTTCGGTTAGCTCTTGTGCTCCTTTCTAGGATGTATTTTTATAGCGTATTTTGCTGTTGAGAACACT
TTTCACTTGTGTGACCTCACATGAAGTCAGCTTGGCAAACTTGACATTATTCTTTCTTGTTAAGCGGTAA
GGAAACTGAGGAGCAGAATGTGGCTTCTTAGATGTCACTTCCCCCAAAGTGGGGAGGGAGTGACAGGCTG
GGCTCAGACAGCTGCAGAAGACCCTGCGACTGCAGTGGAGAGCCCATAGTTGGAGTTGAGGCTTCTTGGG
GTGACTTCAGACTAGGCTCTGAATAGAAAAATACATGGAGAATGGGAAGAAATTAATTTGAAAAGCAAA
GGCTTATTGGCTGGCATCCAGAATGAAGGACTCTGCTTGCAATGCGGGAGACCGGGATTCGATCCCTGGG
TCAGGAAGATCCCTTGGAAAAGGAAATGGCAACCCACTCCAGTATTCTTGCTTAGATAATTCCATGGACA
GAGGAACCTGGTGGGCTACAGTCCATGGGGTAGCAAAGAATTGGACACTACTGAGCAAGTAACACTCACT
CACTTACTCAGAGTGAGTTCAAATTTGCCAGGTTGGTAGAAGTGCAGTGACCCTCCCCACCCCCCCACTC
CCAAACTATAT

>TRIM15|hg19
CAGCCGGCAGAAGGCGTGTCCGCAGGCGGCGGTCACTGCATCCTCCAGGGGCCCTTTGCACTTGGAACAG
GTAGCCCCTTTGTGGGAGGGGGTCGAGGGCATCACGGTGTCTTCCCCAGCCCGTCCTCTCAGGCCTCTGC
CTCAGTCCACATGAACCCTCTTTCCCTGAAATGGAATCACTTTACATGAGTCACAGAGGAAAGGAGTGGG
AGGCTGAGAGAGGGGAAGGCAGGGGTGTCTGGACAGAAAAGCCACCTGGCTCCACCTCTTGTGCTTCCCA
GAGTCCCGGCTACCACTTATCAGCTGAGTGGGTTCAAACTCCAGGGCATCTCGAATTACCCAGCATTCCC
CAACAGCGGCCCTGGCAGCCCGTTCTCCCCTCGTCCTAGTCCTTCCGGCCCCTGGGGTGGGGAACAGAGC
ACACCATGAGGTCACAGGGAGATGGGGTTTCCTGGAATCCTTCCCTTGACCACTGGCGGGTGGGGAGAGT

TGGGGAGGAGACCTTGCTTCCTGTCCGGCTGAGCAGGTGGGAGCCATAAACTTCAGGCCCTGAGGTATGC
ACAGGTGTGCACACAGCGGCAAAGTCCAGCTGGAGTGGGAGTGCTCACTTCCGGGAGATTGTTCTGGGGG
CTTTGCTCAATGCTGCAGGTGTGATGATCGCCCACCAGGACCCCTCCTCTGCACTTCCATTTTAGCTTTC
AACTGCAGCAAGGAGAAGGCAAAAAAGCATAATACAAGTGATCTGTGTTGATACAATTTAGAAAAGTCAA
TAAAAAAAAAAGATAAACAGGTTCAACCACATTTATCTAAGGCTTAGTATTTTCGTATTGTTACATCCAA
TCTGATAACAGCCCTTGAGAGCAGGTGATGATTATAGGAAATGTAAAGTGAAAGGTAAAGTGACCTTTCC
TGAGTCACAGAGCTAGAAAGCTGCAAGGCTGCAGTCTACTCAAGTCTCCTGGCTTCTAAGCTGCGTGCCC
TTCCTGAACTCCCAGCCAGGACAAGGGGATCAGACAGTCATGGGTCTTCATCCGCTCACATGCATCTGTG
TATAAAACCAAGGAAAACACCTCCCATAACCCCACTGGAGATCAGTCCTCCACCCTGTCTCACTCCCTTT
CCACTCCAGCTACTTTGTGTATCGACTTCCCTTCCCCCCACCTACACCCTCTGCCCCCAAGAGTGCAGTC
CCTGGAGCTGCAGTGTCCAGCACAAGAGCCTCTAAATGCCCGAGGCTGCTGGGCATTGAATGTGGCCGGT
CCCAACTGAAAAATGCCGTATGTGTAAAATACGCCCTGATTCCAGACTTTCTTGTTGTTTACTTGCTCAG
TCGTGTCCAACTCTTTGCAGCCCCACGGGCTGTAAGCCTCCAGGCTCCTCTGTCCATGGAATTCTCCAGG
CAAGAACACTAGAGTGGACTGCCATGCCCTTCTCCAGGGGATCTTCACGACCCAGGGATGGAACCCATGC
ATTGGCAGGTGGATTCTTTACCACTGAGCCACCAGCGAAGCCCCTTCAAAGACTTTAGCACCATATAAGA
AGAATGTAAAATGTCTATGACTAATTTTTTCTACCGATTACACACATTGGTACTATTTGGGGCTAAATAT
TAGTTTCCTGTCACCCTGCTTGTTTAATCTATATGCTGAGCACATCATGAGAACCGCTAGGCTGGATGAG
TTACAAGCAATGGAATCAAGATAGGCAGGAGAAACATCAACAACCTCAGATATGCGGATGACAACACTCT
AACGGCAGAAAGTGAAGGGGAACTAAAGAGTCTCTTGATGAGGATGAAGGAGAAGAGTGGAAGAGCTGGC
TTAAGACTAAATGTTAAAAAAAAGATCATGGCATCCAGCACCATTACTGCATGGCAGATGGAAAGGGAAA
AGGTGGAAGTAGTGAGTGACAGATGTCCTCTCGGGCTTCAAAATCACTGTGGATGGTGACTGCAGCCTTG
ACATCAGAAGATGACTGCTTCTTGGCAGGAACGTGATGACAAACCTAGTCAGTGTGTTGAAAAGCAGAGG
CATTACTCTGCAGACAAAGGTCTGTATAGCCAAAGCTATGGTCTTTCCAGTGGTCACGTACAGTTGTGAG
AGTTGGACCGTAATAAAGGCAGAACGCCAAAGAATTGATGCCTTTGAACTGTGGTGCTAGACAAGACTCC
TTTAAGTCCCTTGGACAGCAAGGAGATCAAACCAGTCAATCTTAACGGAGATCAACCCTGAATATTCACT
GAAAGAACTGATACTGAAGCGGAAGCTCTAGTATTTTGGTCATCTGACGTGAACAGGCAACTCATTAGAA
AAGTCCCTAGTGCTGGGAAAGATTGAGGGCAGGAGAAGAAGTTGTCAGAGGATGAGATGGCTGGATGGCA
TCACTGATGCAAAGAACATGAACTTGGGCAAACTCCAGGAGACGGTGAGGGACAGGGAGGCCTGGCGTGC
TGCAGTCTAAGAGTCGGACACGACTGAGTGACGAAACAACAACAATAATTAGTTTCACTTATAAAAAAAT
ATGGCTACTGGAACTTAAAATTGCATATGTGGCTCACATGGTATTTCTGTTGGACAGCATGGCTCTGAGA
TTTACAAATCTGAGTGAAAATTATGGCCTCTCATCTCCCTTTCTGTGTGCCTTGAGCAAGCTAAGTAAAC
TGGCTGAGCTCAGCTAATATCGTAACAGCACTGACGTCACAGAGCAGACTGGAGAAATAAATAAGATGGC
GTGTTTAAACCGCTTATGTCAGTATGAGGAATATGGTTATCGTAAATAAACTCTGGCTGGTGTTATTTTT
CTTTCTCCTCCCCCACGCAGAGTACATTCCAACCCCTTGCGCAGCCAAAGGCAGGCCGGTTGGAGCATCA
CTGGTGAAGTTGCTTGTGCCCTGATCCACCGCCCCTGCCCCGGGCACAACACCCTTATCTGAGTCCCCAC
ACTCCCCTGGGTTGGCTGATACCTTCAATCTCCCTGGAGACAGCTGGTCATACTGCAGGGAGGCTGGGTG
GCATCACTGCTCTGTGACCCAGTCTCCCTCCTGCTCCCAAAAGAACAGTCTCTTCTACCCATGGGTGGTT
AAGATACTTCTACCGTGAGCGTCTCTGGATGGACGGGACGAGGCAGCAACCTGCTGGTCATGGGCACCAG
CTCAGACAGTAACTGAGGTCATCTCTGGGAGGCTGTCACCCCTTCTTCTTCCCCTCCTCTGCTATTTCTC
TGTTTCTGCCACTGGAGGTGTGTGTCTGTTCCTTTGGGAGGGGGAAAGCAGAACCCTGGCTGCCAGGCGT
GCAAGTGGGTG

>TRIM40|hg19
CACCTCTGTCAGCATCTCTGTCAACCAGCATCTCTTTCGGGGTTATACAAATTCCACAATCAAAGGGACA
GAGAAGAAGAAAAGGACAGAGAAAAGTCCAGAGACCTTTTCCCTTCTTTTCAAACCACCTCCAAAGACCC
AAGGAAGAAAGTACTCTCCTGTCCCTATGTCCCTTAAAACACTGCGTTCAACTCTCATCCCCTCATCCCC
AAGCCTCTTGTCTAGTCCTCCTCCAGAAAAGCTCCTCTGGTATCCTACCTCAGTTTCTCCCGTGGCGATT
TCAGGTCGTGTCTCACGGTCCAGATCTGGAGAGATGGCCAGCAGGCGACTGGCTCTTTTTAAATCATGCG
AATTATATATGCTCACGTGTTAGTAAAAATGGGGGAAAGGTGATGAGGCCCATCCTCCCTACAGCTGCGT
GACTAACCGAGGGGTGAGATGGACTTTGGTTGGTGAAAGGTGTGGCTTGTTTGCACATAGGAAGGAGGAA
AGTCTTGGTGCCTCCGACGTGAATACGACACGAAGCCAGTCAGGACACAGAATGATCCCAGAGCCCAGCT
GATACCTCGGGATCCAGGTGTGGTCGGTGTCGGTGACACAGAACCATGGCATCTGCTCGTGATACCGGAA
TGCCCGGTGCCCACCCACTTCTAGTCAGGCTGGCCTCAGCACTCACCCTGGGGGAGCCCATGGGGAATAA

CACTAATAAGAGTTGCCAGTTGAGGCCTTTTTTTTTTTTTTTTTTTGCCACCCTGTGAGATTTGTGGGAT
CTTGGCTCCCCAACCAAGCACTGAACCCAGGTCCTTGGCAGTGAAAGTGTGGAGTCCTAACCACTAGACC
TCTAGGGAATTCCTGAGGCTTTGTTAAAAGGTCCAACATTCTTGTTCAAATCTCACTCCATACTATCCTT
CCTCAATCCCACTTGGCCACTGTCTGCTGTGGGGTTCTTATTAAAAAGTCACTTTCCCAGGCTTCCTAGG
TGGCTCAGTGGTAAAGATCTGCCTGCCAATGCAGGAGACATGGGTTCAGTCCCCAATCCAGGAGGATCCC
ACATGCCATGGAGCAACTAAGCCCGTGTGCCCCATCTATGAAGCCAGTGCTGTAGAGCCTGGGAGCTGCA
ACTAGAAAGCCCATGTGCTACAACTACTGAAGCCTGTGTGTCTAGAGCCTATGCTCCACAAGAGCAGCCA
CCACAGCGATAAGCCCTAGCACGGCAGCGAGAAGTAGCCCCCACTCAGAGCAACTAGGGTAAAGCCTGCA
GAGCAATGAAGACCCAGCACAGCCAAAAATAAATAAATAATAGATAAAAATTATTGTTTAAAAAAAGTTA
CTTTCACCATAGTCAGAGTTACTTGGCCTTTTAATCAAAGGCAATGGCTCCCTCAAATCTCAGGGTGACA
GATGGAATGAGCATCTTACACACTCCCTACCACCACCTCCTGAGCGTGATGCCCCAGAGCCAGATAAAAA
TACCTGTGTGTGGAGCTCTGGCTGTCCACTTTCTCCCTCTGCCTTGCTGTCCTCCACAGAGCCAATTCCC
CCGATTTGTTACAGATGAGCCACGGAGTCTTGTGAATGAAGCTCATGGGCTCTTCCCCACCCTACCTTGC
AATCCTGCTGTTGTCCTGGGTGATTTCAAGGACGATGTAGTTGACCATCAGGCCTGAGGCCCTTAAGCTC
CTTGACTTGCTCACATCCAGTGACGTTTCCTTTTGGGGACCACGTTGGTAACCATTCTCTGGTCCTTGTC
AGAACAGCCTTTACCACCCAAAGTCACAAATCCAGGCATTAGACTTGGCTTACATCTCTCTCCCAAGCTT
CTTACTACATCATCTGGTCATCAATTTCTAATTCTTGACCCCTCCACATTCCTAATTTGTCACGTCTCAT
GAGATAATATGAGAAAGGAAAATGAGGAGAGATTAAGTAACCTGCTGAGTCCATAGACTGGTAAACAGGG
AGTTGGGATTTAGATCTGGGTGTCTGTCATTTTAGCCCATGGGCTTCTTCACCACAAAATGTCATCTTTT
TACACTTTTCCTTGTCCTTACAAAAAGATCAATAAAGTCTTAATAAATGGAAAGATGAATTGTCAGATAG
ATTGATAATTATCTGACTTGGGAAGTTAGTGGTTGGGAGGATCCCTTGGAGAAGGGAATGGCAACCCATT
CCAGTATTCTTGCCTGGAGAATTCCATGGACAGAAGAGCCTGGAAGACTACAGTCCATAGGATTGCAAAG
AGTCAGACATGAGTTAGTGACTAAATGACAACAACAAAGATATCTATTTAAACAATTACATGTAAAGAAG
AAAAAAAATCTCTGGCTAGAATGAAAAAAATTTTAACTACCCCAAAGATCTGGATTATGGTCCTAATTCT
GAGATTCGGAGCTTCACTGCCAAGACAGCCACAGTCTCAGCCTCCTTCCCAGGAAAGCTGGGAATGGATT
CTGGATAGATAGATGAAAAATAGCACTTTGTGCCACTTTGGTTTACTGATCATATGGTAACTTCTTCTAC
AGTTATTCAGGTACTTTTCTTGATTTTCCTACAAGCTTATTGAGCAACTTGACGGTAGAGACACACTTAA
TGCCAACTTAAGAATAATTCAAATATAGAAGTCATAGAAGAATAGACAAATAAATTCAATCATATTTTAA
AAGATCTGAGTGTGTGGGGGGGCGGGAATTGCGAAAGAATGTCAAGAGACAAGTGACAAAATGAAAATGT
AAAACGAAAACATACTTAGAATTATCATCAAAATCAAAGGGTTACGACTTCTCTGGTGGTCCAGTGGTCG
AGAATCTGCCTTGCGATGGGATACGGGTTCCCTCCCTGGTTAGGTAACTAAAATCCCACATGTGGCAGGG
CAACTAAACCCATGAGCCACAGCTACCGAGCCCGTGCTCCACAACTAGAGAAAGCCTGCAAGCCCCAGTG
AAGACCCAGGGCAGCTAAAAATAAAATAAAATAAAAATGGTAGTTTATAAAAAAAAATCAAAGGATTAAT
TCCCTTAATGCATAGATAACTCACACACACAAAAATTAACAAGTATGAAACTGGACAGAGGAAATACACA
GACACTTCATAAGAAAAAAAGTCTCTAAAACATGTGAAAAGATGCTCAATCTCTCTCCCATGAAGAAAAG
GCACATTAAAATGGAGAACTGGGGTTGGAAAGGGACTCTTCTTTACGTTCCCATTGTACCTTTTAAATTG
GGAACCAAGTAAGAATATCAGAGTCAGACACGACTGAGCGACTGAACTGAACTGAAGAATATCACCTGGG
TTTCCCCTGTG

>PPP1R11|hg19
GTCCCCCTTTTCCCTTCCTGGTACTAAATGCGTCAAAGCGCTGGGTCGCCGGATTAGTTCCGCCGTAATG
TGACAGTGCGCAGGCGCCGCAAGGAGGCGACACAACTTTGGCGGAAGAGGCATCACTTTTGTACGGGAGC
AGGAGGCGGGGCGAGCAATGACTGCATGGTCTTGCAAAGCAGAGATTGAGCAGGAATCCGCGGTGGAGGT
TAGAGCTGGGCCTGTTCCTGAAGACTGGGTACCGCACCCGGTTCAGACGTCCCGTAGCTGAGTCCATTTC
ATTGCCATTCCTTGTGTGTGTGTGTGTGGCGGGGGGGGGAGCGGGGCGGGGGGGGGGGGTGAGGCGGGA
AAAAATAAGGGGCAATAGTAATGATCCTACCACAGCCTAACTTCGTTTCCTTATTGTTTTATCTGGGAAT
TATGCAGTCGGGACCTGGGAGCAGCTGGAAACACACTTGATTAAGAATTTTAATCCTCCCTCTGTGCCCG
TAGGGGTTGGAGCAGGGCACTTAATGCACCTTCAATATGGATTATTTCCTGTCACTGGCGCATGAAACAC
TCTCGAGTCTGCGTTTTAGCTCCAAGTCTACAACAACAAATGGGGAAGACCGGAAAAAATACTAACAAAA
ATATAAGACATTAGAATATTAACATAGATGTAAGACTCATCTGTGAAACTGAGTTTAGTCACATACTGCC
TACACTTGGTCTGGGGTGGAGATGGGAAAGATTAAACACAGAAATGGTCATCAGTTCAGTTTAGCCACTC
AGTTGAGTCCAACTGTTTGCGATCCCATGAATTGCAGCACACCAGGCTTCTCTGTTTACCACCAACTCCC
AGAGCTTACTCAGACTCATGTCCATCGAGGCGGTGATGCCATGCAACCATCTCATCCTCTGTCATCCCCT

TCTCCTCTTGCCTTCAATCTTTCCCAGCGTCAGGTCTTTTTAAATGAGTCAGTTCTTCGCATGAGGTGGC
CAAAGTATTGGAGTTTCAGCTTCAGCATCAGTCCTTCCAATGAATATTCAGGACTGATTTCATTTAGGAT
GGACTGGTTGGATCTCCTTGCAGTCCAACAGACTCTCAAGAGTCTTCTCCAACACCACAGTTCAAAAGCA
TCAATTCTTCAGCGCTCAGCTTTCTTCACAGTCCAACTCTCACATCCATACATGACTACTGGAAAAGCCA
TAGCTTTGACTAGATGGACCTTTGTTGACAAAGTAATGTCTCTGCTTTTTAATAAGCTGTCGAGGTTGGT
CATAACTTTTCTTCTAAGGAGCAAGTGTCTTTTAATTTCATGGCCACAGTCACCATCTGCAGTGATTTTG
GAGCCCCCCAAAATAGTCTGTCACTGTTTCCCTATCTATTTGCCATGAAGTGATGGGACCACATGCCATG
ATCTTAGTTTTCTGAATGTTGAGTTTTAAGCCAAATTTTTCACTCTCATTTTCATCAAGAAGCTCTTTAG
TTCCTCTTCACTTTCTGCCTTAAGGGTGGTGTCATCTGCATATCTGAGGTTTTTTATATTTCTGCTAGCA
ATCTTGATTCCAGCTTGTGCTTCATCCAGTCCAGTATTTCTCATGATGTACTCTGCATATAAGTTTTAAG
CAGAGTGACAATATACAGGCTTGACATACTCCTTTCCTGATTTGGAACCAGTCTGTTGTTCCATGTCCAG
TTCTAACTATTGCTTCTTAACTTGCATACAGATTTGTCAGGAGGCAGGTCAGGTGGTCTGGTATTCCCAT
CTCTTTAAGAATTTTCCACAGTTTGTTGTGATCCACACAGTCAAAGGCTTTGGGATAGCCAATAAAGCAG
ATGTTTTTTTCTGGAACTCTATTGCTTTTTTGATGATCCAGTGGATGTTGGCTCTTTGATCTCTGGTTTC
TCTGCTTTTTCTAAACCCAGCTTGAACATCTGGAAGCTCATAAATTACAATATAAGCCAGTTTGTATGAT
AAAAATAAGTGTATGTTTCTCAAGGAGCAAAAGAGTGGTAAAGAACAGGCAAGCAGACGAATGAGTTTTT
TAGGTAAAGAAAGCAGCATATAAATTACTTGTGTGTACTGAAGAAAATGCATTCCTGTATTTGCAGAGTA
CATCATGAGAAACTCTGAGCTAGAAGAAGCACAAGCTGCAATCAAGATTGCTGGGAGAAATATCAACAAC
CTCAGATATGCAGATGACACCACCGTTATGGCAGAAAGCAAAGAGGAACTAAAGAGCCTCTTGATGAAAG
TGAAAGAGGAGAGTGAAAAAGTTGGCTTAAAGCTCAACATTCAGAAAACCAAGATCATGGAAAATAGATG
GGGAAACAGTGGAAACAGTGTCAAGACTAATTTTTTGGGCTCCAAAATCACTGCAGATGGTGATTGCAAG
CCATGAAATTAAAAGACACTTATTCCTTGGAAGGAAAGTTATGACCAACCTAGATAGCATATTCAAAAGC
TGAGACATTACTTTGCCAACAAAGGTCCATCTAGTCAAGGCTATGGTTTTTCCAGTAGTCATGGATGGAT
GTGAGAATTGGACTGTGAAGAAAAGCTGAGCGCTGAAGAATTGATGCTTTTGAACTGTGGTGTTGGAGAA
GACTCTTGAGAGTCCCTTGGACTGCAAGGAGATCCAACCAGTCCATTCTAAAGGAGATCAGTCCTGGGTG
TTCATTGGAAGGACTGATGCTAAAGCTGAAACTCCAATACTTTGGCCACCTCATTCGAAGAGTTGACTCA
TTGGAAAAGACTCTGATGCTGGGGGGGATTGGGGGCAGGAGAAGGGGACAGAGGATGAGATGGCTGAATG
GCATCACCGGGTCGATGGACGTGAGTTTGAGTGAACTCCGGGAGTTGGTGATGGACAAGGAGGCCTGGTG
TGCTGCAATTCATGGGGTCGCAAAGAGTCGGACACAACTGAGCTACTGAACTGAACTGAAGGAGAGTTCC
AGATGATGACTGACAGGTAGACGTGGTTATGAGGCCCTGTAAAGTTTGCAAGAAACTTGGAGATTATCCA
GTCCGTAAAAGGCTTAAAGTAGGACAGGACATGAACAGATTTTATTATATTTCAGGAGTGGGGCACTAAA
GTCAATTACCAGAAATGTTCAGACACACAGTGGAAGAAACTAAGTTGCCCATCTGTCTAAGTTGCCATGG
ATAGTGTATCAGTTCAGTTCAGTTCCATTCAGTTGCTCAACTCCTGGAGCTTGCTCAAACTCATGAGAGC
TCCCAAAATATAAAGGGCCACACAACTGTAAGATAAATCATGTCTGTCATCTTCTCAGTCCAGTTCAGTC
GCTCAGTCGTG

>POLR1H|hg19
CAGAACCCGAGTCCTTCTCCCACGTATGCCTGTGCCTTTAAGACCAATGAGCCCACATATCTAGAATTCC
AAGCCGGGGTCCGGTACTTCGACTTGGGGGCTTGCTTACCTGGGACAGGCGAGAAGGGGAAGCCAACTCC
GCAGGCTAGAACGCCGGCTCTACGCGGCAGCGAAGTAGCGAGAGCAGGGCGGGCTAGAGAGCCCCGTCGT
TCGGCTTGTCGATTGGCTGTTACGTCATAATTAATCGCAAACTTGGTCCCCGGAGGGAGAGGAACCATTG
AAGGAAGGTTGTAGCGCTACCTTCAGGCTGGTTTTGTGACGTTGGATAAGAAAACAGGTGATTGAAGCTG
AGCCTCTGGTTGTTTTTCCCCTCATTACCCAGTTCTGGGAACTACAATTTCCTCTGTTCCTGAGTTGGTT
TAAAAAAAAAAAAAAAAGCGAAACATTCTGGGAGTTACAGTTCTAGCGCTCCTCGGACCTCGCGATGCAA
GCAAGGGCGCTCTGCAGAGCACCGCCTGGCTCGAGTGCTGGGCCCTTGGCGACGAAGAATTGATGCTTTT
GAACCGTGGTGTTGGAGAAGACTCTTGAGAGTTCCTTAACTGCAAGGAGATCAAACCAGTTAAGCCTAAA
GGAAATCAGTCCTGAATATTCACTGGAAGGACTGATGCTGAAGCTGAAACTCCAATACTTTGGCCATCTG
ATGCGAAGAACTGACTCATTTGAAAAGACCCTGTTGCTGGGAAAGATTGAAGGCAGGAGAAGGGAAGGAC
AGAGGATGAGATGGTTAGATGGCATCACCCACTCGATGGACGTGAGTTTGAGCAAGCTCCGGGAGTTGGT
GATGGACAGGGAGGCCTGGCATGCTGCAATACATGGAGTCGCAAAGAGTCGGACACGACTGAGCGACTGA
ACTGAGCTGGGCCTTTCGAAGGGGTGAGTCGGCCTCTTATGGGGCATGAAGTGGAAACGGTGAAGGCGGA
GGAGGAATGCCTAGTCACTAGGAAGGGATGAAGTAGGACACTTTAGGAAGGGGCTCAAGACTTTGGAGGT
AGTGGAACAGAAGAGATTGGGACTCCTTCCTCTTTAGATGCCTGTCAGATGAGGGAGGTTAGCATTAGTC

AAGGGAGCAGCCTTGTGACGAAGACTGTCGAAGTTTGCTTTCAGTTGCGGACCCCGTTCCACCTTGTCCC
CGGCCTCCCATCCGACCAGCTCTACATCATCATTGCATCCATTAGATGGAGAGTTGACATATCCCTGATC
TTTTATAAAACATGAGGACAAGTGTCTTAAAAGTCTTGCTGTTTTAATTCATCTGGAACCTACAATTTAA
AGTGTAGAATCTTGTCGTCCTAAAGGGACAGCTTCTAGATGAGTTTCCTAGTTCTCTCCCAGCCCATCCA
CCCCCTCTCAACCTCATATCTGTGCTAAGCTGCTTATGTATGAAAGTTCCAGAGCCATCACTATTTTCTC
CTGTTTGAAAGAGAACCAGCTGTGGTCTCTCCTGGAGAGGTTTTGATCCTCTACTCTCTGAAATAATGGA
AACAAACTAGTGAGAAGGTTAAAACTCTGACAAAGCTACTAGAAAGTGACAAGCCCCTTAATAATTTGTC
TTACAAGGAGAAGTCTGCTGAGCCCATGATGGTAGGCAAGTTTTTATTCTCTCTTCTCTGTGTGTCTGTC
TCCTTTAAAAATAGAAATTGAGATTGGTCCAACTTTCCCTTTCAGTATCAGTTCAGTCTGTCAGTCCTGT
CCAACTCTCTGCGACACCATGAACCACAGCAGGCCAGGCCTCCCTGTCCATCACCAACTCCCGGAGTCCA
CCCAAACTCATGTCCATTGAGTCGGTGATGCCATCCAACCATCTCGTCCTCTGTCATCCCCTTCTCCTCC
TGCCCTCAATCTTTCCCAACATCAGGGTCTTTTCAAATGAGTCAGCTCTTCGCATCAGGTGGCCAAAATA
TCGGAGTCTCAGCTTCAACATCAATCCTTCCAATGAACACGCAGGACTGATTTCCTTTAGGATGGACTGG
TTGGATCTCCTTGCAGTCCAAGGGACTCTCAAGAGTCTTCTCCAACACCACAGTTCAAAAGCATCAATTC
TTCGGCCCTCAGCTTTCTTTATAGTCCAACTCTCACATCCATACATGACTACTGGAAAAACCATAGCCTT
ATGTCTCTGCTTTTTACTATGCTATTTAGGTTGGTCATAACTTAACTTCCAAGGAGCAAGCCTCTTAATT
TCATGGCTGCATTGACAAAACTCAGTGAATATCCACAATGAGTTGAATACTGTCCTAATGCAGTAAAGTA
ACTAAGCATTATAAGGCATGTCTCTTTCCCAACAAAAGCTCAACAGTCTGTTCCCGTTCATATTTATTAA
GCACATATGCCAGTCTTTGAGCTGAAGTTGTGGCTGTCAAGCTAAAAACTGTGTCTCTGACCTTAAGGAA
TACAAATGAATAATTAGAATGCAGTGAAAGTACACTTACAGGACAAAATGTGAGAGCCCAGGAAAGGCAT
ATACAACATATATGCCTGTATACTTCAAGGAGGACTATTTATTAAAAAGTCCTACTTATTAAATAGGACT
TTAACTATGTATTAAATAGTTTATAAAAAAAACTATAAAAAAGTTTCAATAAAAAATATTCAGTATTATGG
AGAAATTAAATAGTTTATAAAAAACTATAAAAAAGTTTCAATAAAAAGATTCAGTATTATGGAGAATGTA
AGAGGATGATGCTCATATTCTGTGGTGAGGATAATCACCAGTTAACAAATCATTAATTTTGAGAACATGG
GATTACAGACTCTAATGTTAGGTACTGTGCTAGGTACTGTGTACACAGAAATAAAAGTAAGGTGGTCATA
TTTTTAAAAATCTCAAGTATATGTAGTCAGGCAAGTAAGAATATGCCCAAGAGGTTAGTGAGATCCAATA
TTCGCTAGCAGATCCTTTCTAATATGATAGATAGATGTCTATATTTGTAGATAAAAAAGTTATAGCTCCT
ACTCTATGAGTCTGAATTTCAGCATCAAAATGTTAAAAGTGATACATGGATACAGATATCACAGTGGAAA
CCAGGATTGATTTTCAAAAGAGTTGTAGAAGTAAAAAATATTATGTTTATAGGGAGGCAAAAATTGCTGT
TACATAGAGAAGACTGGGAAGATTTTATGGTCAAGTCTTGACCTGAGGAGGATGGACAACTGCTGTTTAT
TGAGAACTTACCATATATAAAGCATTGTGCCAGGATCTTTAATTGTCATTTGTTCTTTATAACTCCAAGA
GGCAAATTTTT

>MOG|hg19
GGCACTGAAGGTGTCTGAGGGGAACCATGTCAGCATATCTATGTTTTGGAAAGATCACTTGCACGGCAAC
TTGAGGATGTATTGAAGGAAGTAAGCCCAGAAAACTAGCCTTTCTGTTCTCAGAGTCTCCCCAGGGCAGG
AATAAAGAGAAATCTTACCTGCAGAGCTGGAAGTCAACTGGAGGAGGAGGAAGAGGAGGGAGGGGAGACA
GCTGGGCAGAGAGGAGCTCAATAAACTGGCCATCTCCACTGTCAGTGGGGACAGAGAGCCCCTGCAGCAG
GGTCAGGCCAAGGAGAGGCCCTTGGGGTGCATGTCCCCACCCCTGAAAAAGTCCTAGGGGAGGATCGGGG
AGGGCAGAGCTGCCCTGGCCCTGTCTCCTCCCCAGCAGAACCACAGCTGTGCTCCCAGGTGCAGCCCCAT
GTCATCCCTGCCAGGACTTACACAAAAGGGCTACAGAGCCAGGGGATGACCAGGGCTTGCCTTCCCCACC
CCCTCAGAGCTGGAGCAGAGGCTGCCTGGTACTGGGCCTTCCTCCCCCACTCCCTCCTGGCGGCCAGCCT
TCCCGGGGGCTTCTAGGATCTGACCTGTTGGGATGAATCACTCACTGGACCTCAGGCTGAAGCTTGTCCA
AGTGATCTGCAAGGTCTCTTCAATCCCTCACCTCCAGGCGCTAGCCAAGGGAGAGGGATCTGTGTTGGAT
TTTTTTAAAAAATGAAGAAGAAACATTTAGACTTAGTTTCCTATTTCTACCATCTACTGTCATCACCAGG
CCATTCATTCCTCTTGATATCTGACCTCCCTCTCTCTCACTGCAAATCTAAATGCCCTTCCTTATGTTCT
GGTGTGTTGCTTGGATTTAGTTAGCGTCTCCCTCCCAAAGAATATTAATACAGTTTGGAAAGTCACAAGT
TGAGTGTCACGAGTTGATTGCCACCTGTGCTTGGAGAGGCAGGTGTGAAGAAAGTATTATAAGATCTGCA
AAGTCCGTGACTGTCAATCAACACAGGCATTGAGGACAGACCAGCAACCAGGATGCATCCTCCAGCCGGC
GCATCACTCTCCAGTTTCCTCCGTGTCACAAGGAAAGTGAAATCTGGGATGGAACCTGCATGGAACAGAC
CATGGGGTCACAAAGAGTCGGACACTGAACAACCAGATAAACAAATAAGAATCCATTTTCCTTGCATTGG
ATGCTCAGAGTCCCAACCACTGGATGGCTAAGGAAGTTGCCGAGAGCTAGGATTTGAAGTCACTCTACCT
CTGAAGAGCTCACTTCCCTTACCTCCATGCCCTCGACTGCCTCACATCTTTGTAAAGGGGGTGGATTTCT

GGAGCTGGGGTGAGCAGTCCCTTGGATGACATGGTCTCTGGACAAGGGGAGAGAATGGGGAAACAAAGCC
GGTGGTGTTGCCATTTACAGAAATCACTATAGGAGCCTCTTTGTTTGCTGAGCATGGAGCACGGTGTGGT
CGTGTCTAACAACCCTGTGCTGTTGGTCAGGCTGGTCGTGACGGTCAGCAGTTAATTGTTGCTTTAATTC
TGTGTGGCACATGTTATGTGATCTTACAAATATTCTTCAGTTGTTTCTTGCTCTCCTCAGTAGTTTCCAT
TTGAGCCACTCCTCTCACCAGGTCTGATAACAGTCTGTGTACAGAGTCATGTATGCAAAGTGACATGCTA
TAAATATGTTAATGTATATGTTAAACAACACACTATCCATATGTGAGATAGTAAGCACTCAATAAATGGG
AGCTTACCTAGATGTTCCCTAAGAGCAGTACTATGTAAGTTTTTCTAGAGAATGTGTAAAATATAGATCA
TGCACTGTTCTGCTTCTTATGTTTACTTAATAATGCATAATCCCACATTAATATATAATATAACCCTCTT
GAACTTTTGAAAATGGATTCTTATTTGTTTATTTGGTTGTTCAGTGTCTGACTCTTGGCGACCCCATGGT
CTGTCCATGAAATTCTTCAGGCAAGAATACTGGAGTGGGTTGTCATGCCCTTCTCCAGGGCATCTTCCCA
ACCCAGGGATCGAGCCTATGTCTCCTGTATTTCAGGCAGATTCTTTATCATCTGAGCTACCAGCTACTAG
GTTGCATATAAATATATTCCATTAACTTAAATTAATAAATATTTAGCAGAAATGATGTGTGTCAATCCTT
ATATTAGGCTCTGATTGTAAAAGTTTCAGCAGGACAAAGAAAAAAGTTTGGTTCTTTTTTGGCCTGTACC
ATGAGGCTTGTGGGATCTTTGTTTCCTGATTAGGGTTGAACCCAGACCTCCCGCAGTGGAAGCCTGGAGT
CCATGCCAGTGGAAAGCCAGGGAATTTCCAAGAAGATTCTAGCTTTCTCTATGGTTTCACTCTTGATATG
TCACAGTGGCTTTGTTTTCGTTTTTACTATTTAATGTTGCATTTCTTCAGGCATCTGGGAACTTAGGTGG
GTAAATGCAGGCCTTCCCACATGCCTAGGGTCCTGTCCCTCTACTGGGCACAACACATGTGTATGCCTGA
CTCCACTTCTCCCCTGTGCCCAGGCTCCTGCTGGGGGGCACTGAGGGTGGCAGTGGTTCCTGGGGGGAGC
TGAAGACATCTGAAAACCAGTCCTGGGGAGATTAAGAAGCGAGATCAAGCTTGAATCTAGGCACCAAACC
CTCAGCACACAGTCTATTACCCCATTGGACTTCTCTTACAAAACACAAATTGAAAGAGAAGTGTTAAGAA
TTTCAAGATAGCGACATAGAGGGCAATTTCCTGGTGGCCCAGTGGATAAGACTCTATGCTCCCAATGCAG
GGGGCCTGGTTTGGTCCCTGTCAGGGAACTAGATCTTGCATGCTACAGCTAAAACCCAGAGCAGCCAAAA
AAAAAAAGAAAAAAAGATGGTGACACAGAGCATTAAAGCCCAAACAGTGTTCCCTCCTAACCATGGGGTC
GCGTGCAGCTGCGCTGGTTGTTCACTGCCTTCTATACAGGTGCAGTTGTTAGCTCAAAGAGCATGTGCAG
TTGCAGTTTTAATAAATTGCCCTGCACAAAGCTGTATACTCTGTACATGTGAATATACGATAGCTCAGCT
TTCTCCTCTGCAAGTGGAGGAGAATGACATCTGTCCCATTAGGACTGTGAACAGAATTACAGGAAGGAAC
CCACGGGAACTGTGAATGGTACACAGTAAACTCTCTCTAAGTGCCCTTATTTGCTCTCACTTGCAGTGAT
TTTTTTCTTCTGGAGCTCCTCCTAGTGGTACAAATATCAGGAGGAAATTGATATTTTCCGCTTTTGTGGT
CTTATTTTCAC

>ELOVL5|hg19
GCGGGCTCCTTCGCCACGCGGGCGCAGCGCGGGCCGGGCCGGCAGTCGCTCGTGCGCTCCGGGTTAAGAA
CCCAACAGCGGGGTCGCATCCCTGGGTCCCGGCTGCGCGCCGGCGCGGGTGTCATGGCCGAGCGGCGGCC
AGCCAAAGTTTTCCGGAGCCGCCCGCTTACCTCTTAGGCCGAGCGCAGCGGCGGCTCGGAGAAGCGGCTA
GGCGGAGGTGGCGGCGGAGCCCCGGGACCAGCCGAGCCAGGAGGAGGCTGGAGGGAGGTACCGGCCAGCA
GGAGCAGCCGCGATGAGAAGAGGAAGGCGGCGGGTATCTACAATCAGAACGTGGGCTGACCGTGACGTCA
CAATGGCGCGGCCAGCCAATCGCCGTCCGGAGGCCGAATCTAGCGCTATGATGTCATGCGACCCGGGAGC
CAATCGTGAAACAATAGAACTTTCGTGATTGTAAATTCCAGAATCCGATGCAGTGGAGAGCAGTGGTCCG
GGTTGGTCCCCTCTGGTTCACGTGTCTCTGGACAATTTTATCCGCTTTCTTCCTAATAAGCCTGCGTGGA
ACGCAGAGCCTGCGCAATAGCACCGGTGAAGTTCCCGATGCCCTGAACTCTGTTTCTCTCAGTGCCAGGC
CTGGCAGGGTTGTGTGGCTGCGTTTGGACGGCAGACGCCCACTGTGGTGCTGTCTCCTCGCGGGCTCTGG
AAGGGAGCCAGTTCTTGGTTTTAGATGAAAGGGTTAAATGCCTACGGACGTACTAGAGCTAACGAAATAG
GGCAGCAACAACAACAAAAAAAGCGCTGGAAACTGGTGCTGCAGCTTAACTCCCTGTTGGTAGATTTACC
GTGATTTTTCTTAGGGATTCTGATTTTCCCTTAGTGATCACTGCCAACTCCATTTCTCCTCAAGACTTTT
TGTAGGAAATAAGGTGAGGGAGTAAGTGACATCTTTTCAACTAGACAAATACAAGCACATAAGACAAAGT
GCTTTGTGTTTGGGGCATGAGTAGATCCTTGCTGTTTTTAATTGATATTTGGATAATTTAAGGCCTTCAT
CATCCTAGTATCCTATACTTTCTTCATGTCAGGTCTCAGAAATTAAACCTTTCTCCCAGTGCACTCTAAT
TGTATTTCTGTTACCCAAAGCCCATGGATCCGACATAGCCAACTATTTAATGCAGGCAAATTTATCTTAC
ATACTTTTTATCACGTGCCAGGTGTGTTCCAGGCACTGAGCCATTGCCCATTGCCTTAAGAATTAAGATG
TTGAAACTATACACAAATTAGATAATGTAAACATACACAAATAACTAAGATTTTTTAATATCTGATTGCC
ATCCTACTCCACAAACACTGACTGCAGTTTGCAATATTATGCTTGGTGTAAAAAACTGCAAGAATTCCCT
CCCCAAGTAAAATGATACAGTCAGGAGACAGCTACTGTACAGAACGGTAAACTGCTTTGACAGAAGCCAA
GATACATAAATTGATGAATCACTTTCTGTCAAGGGGCATGTGAAAAAGCATCTGTCAGAGGCATAGAAGA

GTTGAGCAAAGATGAATCCCTTACTCTTCATTCTTCTGCTGGAAGTTCAAGGCCAGAGCTGGTTCAACTC
TCTGTCACCCTATCCAAATCTATCTTTCCAGGTTTTAAAATTTTCAAATGTAACTTGCTATTCCAAAAAT
CCAAGTTTTATACCACACAGAATTGTTTTTATTGTGTTTACCTATCAATAAAATGTTTTAAACCCATACT
ATCAAAATATCTGTTGTTGATTAGTCGTTCAGTGTCCAACTCTTTGAGACCTCATGAACTGTAGCCTGCG
CAGCTCCTCTGTCCATGGAATTCTCCAGGCAAGAATACTGGAGTGGGTTGCCATTTCCTTCTCCAGGGGA
TCTTCCTGACCTAGGAATCGAACCCAGGACTCCTGTATTGCAGGCAGACACTTTATTATCTGAGCCACCA
AATCTGTATTTATTTTTAATTATGGACTTATATGGGCTTCCCAGGTGGTGCTAGTGGTAAAGAATCCACC
TGCCAATGCAGGAGACATAAGAGGCCTGAGTTTGATCTGTGAGTTGGAAAGATTCCCTTGGAGGAGGGCA
TGGCAACCCATTCCAGTATTATTGCCTGGAGAATCCCATGGACAGAGGAGCCTGATGTGCTTACAGTCCA
TAGGGTCACACCGAGTCAGACACGGCTGGAGAGACTTAGCACGCATGCACATGAACTTATATAACCATTG
TCATACATTAAATATTAAATATTTTTTCTCATTTTAAGACAAAAGTAAATATAAAGTTTATTTTATTCCT
ATCTCCCAAAGGATGTCTTTTCACATGCTCTTGACAGACATGCCTTAGTTACTATCTGTGTATCTTGGTT
TCCATTAGGGCATTTTTCACTCTGCATTGTAGCTTCCTGATTGTCACATCTTGTTCAGTCTGTGGGGTGC
TTGTTCCTCCACTTTCAAGACCCCTACTTTAGACTGTAAAGTCTATTGGAAGGGAGTTTCCTATGTGTTG
AATTTTGATCATTATTTATTCTTAGATATTACTACAGTCCTGATATATAATAAGAGCTTAGTAAATATGT
TTTTAATAAGTATGTGAGCTCTTATCCCTGCCTCACCATCATCACACTGGTACTTACTTCCATTTCCTGC
TCCTGAACTCCAAAACTCTGTCAGACAGATATGTCCCAAGACTGTTGACTCCTCTCAAACTTAAGCTAGT
AAGGTCGTGTTCTCATTCTCTCCCCATCCAAAGACTTAGGGCTCCCAATACATATATAATTCAGGGAGGA
ACTTCTGCCCTCACCTAACTCATCTTATGCCGTGCAGTGTAAACTGGCTACCATGACTTCAGTATGAGTT
ACTCCCTCTGGTTGCACCATCACATTCATGGATAAAGTTGTAAGAAATGGCTCTCCAACTTAACTGTGGC
TATATTTTTTTTCCCCCAAAATCACAAGAATGGCTAGGACTCCATGCCAGGGTTCAAGAAAAGTATGTTA
GTGAGTGTCAGAATCAACTTCCTGGCCAGGTTGGGTCCTACACCCTTTGGTGAAGTCATTCCAAACTTGA
CAACCAACCCTTCAAAAATGAACCTAAGTAGGTGCCATACAAAGCCCTGTAACTCTTGCTTGGTTCCATA
AAGCATCCATGGAGTACCCCCAAAAGGGATTGTTGAATTACCTTCATGTAATAGTTGACTATTTTACTCC
TTCTCCTCTTCCTCCTCCTGCTTTTCTCTATTTTTAAAATTCTGGCAACATTTTTCCTTCAGTGATCCCA
GACCTCCTCCA

>ENSBTAG00000009656|hg19
GGGTCACAAAGTGTCGGACACAACTGAGCGACAGAACTGACTGAATATGGAGAAAGGTGTGAGGGTCTCT
ACTATTTCTAGGCCAATCTTTCTTGATTTTCCCTCTGAATCCCTGTTCCCCAATTCTAATCTCTACATCC
CTCAACTGTGCACTCGCCCACAATGTCTTCACCTCCACAGGGGCTCATCATGGTGGTCAGGGCGAAGGCC
CCCAGAATCAGAGCTCTGTTCAGAACCATCCTCTTCTCAAGGTGGACTCAGCAGTGGCTGTTCTGAGTTG
CAGAGCAATGATGAGGGCTGAGTTAAGAAGGAACTTTGAAGACACTCAAACCATACTCTGCCAGGTCGGG
TTGCGGCCAATCAGAAAAATCCCCTTTGATGACTCCTCAGTTGCTAGATGAAGACTTTGTACTAAGAGGC
CTGAAAGGGACTGGAAAATCCCCTGGTTCTGATGCAGCCTCCATTCAGAGTGGACCAGAGAGATCTTCCT
ATGAAAGGAGGGAGGATATTAAATCAGAGTCTTTTCACATGTGTGTAAACGTGCTCCAACAGGATTTTCT
AGATTACAACTCCCCAGAACTCTCCCTTGAGTTGGTCCCCTCCCTGGGAGGCCCTGTCACCTCCCCTTTC
CCCTGCCTCCATGCTGTGCTGTGCTTAATCTCTGTCATGTCTGACTCTTTGTGATCCTGTGGACTATAGC
CCACCAGTCTCCTCTGTCCATGAGGATTCTCCAGACAAGAATACTGGAGTTGATTGTCATGCTCTCCTCT
GGGGAATCTTCCCAACCCAGGGATCAAACCCAGGTCTCCCATATTGCAGGCAGATTCTTTACAGCTGAGC
TACCATGGAAGACCAGGAATGCTGGAGTGGATAGCCTATGCCTTCCTCAGGGGATCTTCCCCACCCAGGA
ATCAAACCAGAGTCTCCTGCAATGCAGGTGGATTCTTTACGAGCTGAGCTACCAGGGAAGCTCAATGCAT
CCATATGTAGGTTAACTGTCTTTCTCTTTGCTTCCATAGTGCATATGGAATTCTCTAAATATTGAGATGA
CCCAGGGGAATTCTGTTAGTGGCAGCCAAGAAATACCTTTGCCTATCCTCCAGACCCAGCAGCACTGGTT
GGTCGGTCCTGGAAGTGCTTTCTGCCACCGTAGATGACACAGACTTAGTCTGAATGGAAGCCCCAGTGGT
ATAACATAAATGAAACAGACCAGAATAGCAGTGAAGGGGCTCCTAAAACAAAACTGCCTTCAGAACCACA
CCTTGAAAATTAGGCCAGGAATAAGTGCCAAGATCAAGAAGAGTGTTGTTCAGTCTCCAAATTGTGTCCA
ACTCTTGGCAATCTGTGCACACCAGGCTGCACACCAGGCTTCCTGTCCCTCACCATCTCCCAAAGTTTGT
CCAAGTTCATGTCTCTTGAACTCACTCACCCTACTCATCCACCCCCAACGCACTCCAGCTGCCATCAGTC
TCCTGATTAAAAAAAATCAGAGTTCACCTGAATTCCAGCCACACCCTAGGTGTTGTGACATGCAAGGGAT
CTCTTAATGTAGTCCAAACTGACACATCCATATCTATTGGATACTGATTCTTCAACTCTTAGAGTGATCC
AGACCTGTGGACATTATTTACCTGAGACTATCTTGTGCTTATTTCTTCCACAAATCAACACCAGAGAATT
AACATTACTCATTATGAGGGAGAACCTGAAGTAGTAGACATCTTTGAGACAGTGAAGCTTTGAATAAAAG

AAGCTTACTATGCCATGAAGATCAAGTTGCTATTGTTGTTCAGTCACTAAGTCGTGTCTGATTCTTTGTG
ACCCCATGAACTGCAGCACTGCAGGCTCCTCTGTCTTCTACTATCTCCTGGAGTTTGCTCAAATTCATGT
CCATTGAGTCAGTGATGCTATCTAGCCATCTCATCCTCTGCCCCGCCCTTTCTCCTTTTGCCTTCAATCT
TTGCCAGCATGAGGGTCTTTTCCAATGAATTGACTCTTCACATCAGGTGGCCAAAATATTGGAGCTTCAG
CTTCAGCATCAGTCCTTCCAATGAGTATTCAGGGTTGATTTCTTTAGAATTGGGTGACCACCAGCTAAAA
TAACATATATGAACAGGATCCAGAATTTCTTAACATAATAGCCAAAATGTCCAGGATGTAATAAAATGTC
ACTCTTGTCATACCAAGAATCAGGAAGAGAACCTTGAATGAAAAACAACATTCAATTGATACCAAGACAG
GTCAGATGTTGGAAGTGTCTGAGAGGAAATTTAAAACATCCATTACAAAATGCTTTGATCAGCAATTATA
AATTATCTAAAAAACAAATGAAAATAATAGAAAATTTAAGTAAAGAAATAAAAGTTATAAAAAAGAACCA
AGTAGAAATTACAGAACTAAAAAATAAATAAAATAATTGTCAAAAAGATTCAATAGTAGACTGGAGACAA
CAGAGAACAGAATCAGTGAACTTGAAGACAGATTAATAGAATTTACTCAGTTTGAGCCATAGAGACAAAA
CAGACTAAAAAATATGAACAGAGACTGCGACTTATGAGGTAATAGCAGAATACCTAACATTTTAATCATC
AGAGTCCAAGAGAAGGAAAAGGTGTAGTGTTGAAAAGTATTCAAAGAAATAATGGTGGAAAAATCCCTAA
ATTTGGATAAAGACATAAGAATCCTACAGATTCAAGAATCTAAGCAAAACTCAAATAGGTTATACCTAAA
GAAATTCATACCAAGAGATATGAATTATTAAACTCCTGAAATTAAATGGAAAAAAAGTCTTAAAATCTGT
CAGAGAAAAATGATACATTATAATAAGGGAACACCAAATTACATGACAGATGACCATGGATTTGTTACCT
GAAGCCACAATGGCCAGACAGATGTGACACAACATTTTTCAAGTGCTGAAAGTAAAAAAACTGTCAATCA
TGAATTATCCTGTATCTGCCAAGACTATCCTTCAGGAATTAAGGGGAAATAAAGACATTTCTAGATAAAA
GAAAACTAAGAAAATGTTGGTGGAAAATCTTTCCTTTTGGAGGGAAGTTCTCCAAACAGAAAGAAAATGA
TAACAGAAGAAAGTTTGGAATTTCAGAACAGAGGGAACAAGTGAATGGGTAGGAATGAATTCTGCCAACA
TCCAACAAGTTTGGGAGAGGACCCTACAATTTAGATGAGACCTGCAACCTCAGTTTGTGACTTCAATCTG
GTGAGACCTTAAGCAGAGAGTCTGGCAAACACTTCTGATCAAGAACACTGGATAGTATATGGGTGTTCTT
TTAAACTGCTG

>BOLA-DQA5|hg19
TCCGATTTCCTGAATGAGGAATGACTATAATTTTTAGACTTACTTAATATTGGGAACACTTTAGGAGTCT
CTACTATTTCTAGGCCAATCTCATTTTTCCTCTGATTCCCTGTTCCACATCTCTAGTCCCCACATCCTTC
AATTGTGCACTCACCCACGATGTCTTCACCTCCACTGGGGCTCATCATGGTGGTCAGGGCGAGGGTTCCC
AGAATCAGAGCTCTGTTCAGGACCATCCTCTACTCAGGGTAGTCTTGGCAGTTGCTGTTCTGAGTTGCAG
AGCAATAATGAGAGCTGATCTGAGAAGGAACTCTAAAGCACACAAACCACACTTGGCTGGGTCAGGTTTT
GGCCAATCAGAAAAATCCCCTATGTTGACACCTCAGTTGCCTGTTGAAGACTTTGTACTAAGGGGCTGAA
GGAACTGGGAAATCCCCTGGTTCTGATGCAGCCTCCACTCTGAGTGGATGTGAGGGATTTTTATATGAAA
GGAAGGAAGATGTTAAATCAGAGTCTTCTCACATGTGTGTATGACTGGAGATGACAGAGAACAGAATCCG
TGAACTTGTGAATAGATGAATAGAATTTACTCAATTTGAACAATAGACAGAAACTAGACCAAAAATATAA
ACAAAGATCCTGAATCTTATTAAATAATAACAAAAGATCTAACATTTTAATCATTAGAGAGAAAAAAGAG
TCTAGGGTTGACAAGTACTTCAAGAAATAATGGATGAAAAATTCCCAAATTTGAATAAAGACATAAAAAT
CTTACAGTTTCAAAGAATCTGAGAAAAATCCAAATAGGATAAACCAAAAGAATTTCACAACAAGAGACAC
GGATTATTAAGCTTGTGAAATGCAAGGGGAAAAATGTCTTAAAGCCAGCCAGGGAAAAGTGATACATGAT
GATAAAAGTAAAGTCACTCCGTCATGTCCAACTCTTTGCGATGCCATTGACTGTTGCCTGCCAGACTCCT
CCGTCCATGGGATTTTCCAGGCAAGAATACTGGTGTGGGTTACCATTTCCTTCTCCAGGGGATCTTCCCG
ACCCAGGGATCAAACTCAGGTCTCCCACATTGCAGGCAGACTCTTTACCACCTGAGCCGATAAGGAAGCA
TCAAATTACATGACAGAAGACTGTGGCTCAGTCACTTGAAGCCACAAAGGCCAGACACAAATGACACAAT
GTCTTTCAAGTGCTGAAAGTAAAAAATTGTCAATTATGGATTATATCTGGCAAAATTATCCTTCAGGAGT
TAAGGGGAAATAAAGACATTTCTAGACAAGGGAAAACTAAGAAAATGTTGGTAGCAATACAATGCTTAAA
GAATGGCTACAGGAGCTTTTTCATATGGAAAGGAAATGATAGTACAAGAAAGTCTGGAATATCAGAATGG
AGGGAAAAACTGAGTGGGTAGAAATGAATTCTGCCAACAGCTAGCAAGCTTGGGAGAGGATCTTTCCCCT
TTGATGTGAATTGCAATCTCAGATTGTGACTTCAGTCTGGTAAGACAAAGAGCCTGACATACACTTCTGA
CCAAGGATGCTGGATAATAAATATAAAATTTCTTTTCTTTTAAACTGCTGAGTTTGAAGTAATTTTTGGC
AGTGTCCTCCCGGCCGCCGCCCCTGACCTCAGACGTCGGGTAGCTCCTCTAGGCCGCTGCCCCTGACCTT
GGACGTCGTGTAGCTCCTCTCGGTCGTTCCTGCACCGTTGCAGCCTGGCACTCTCGGCCGCTGCCCCTGA
CCTCGGACGTGGGGTAACTCCTCTTGGCCACCCTCTCTCAGGCATGGGGTCCTCCTGGCTTCTGCCCCTG
ACCTCGGACGTGGGGCAGCCGCCCGCACTAAATACACTGGTTGCAGCTGCCTGGCTAAATGTAACCAGTA
ACACTGAAGAAGCTGAAGTTGAACGGTTCTATGAAGACCTACAAGACCTTTTAGAACTAACACCCAAAAA

AGATGTCCTTTTCATTATAGGGGACTGAAATGCAAAAGTAGGAAGTCAAGAAACACCTGGAGTAACAGGC
AAATTTGGCCTTGGAATATGGAATGAAGCAGGGAAAAGACTAATGGAGTTTTGCCAAGAAAATGCACTGG
TCATAGCAAACACCCTCTTTCAACAACACAAGAGAAGACTCTACACATGGACATCACCAGATGGTCAACA
CCGAAATCACATTGATTATATTCTTTGCAGCCAAAGATGAAGAAGCTCTATACAGTCAACTAAAACAAGA
CCAGGAGCTGACTGTGGCTCAGATCAAGAACTCCTTATTGCCAAATTCAGACTGAAATTGAAGAAAGTAA
GGAAAACCATTAGACCATTCAGGTATGACCTAATTCAAATCCCTTATGATTATACAGTGGAAGTGAGAAA
TAGATTTAAGGGACTAGATCTGATAGATAGAGTGCCTGATGAATTATGGACTGAGGTTCGTGACATTGTA
CAGGAGACAGGGATCAAGACCATCCCCATGGAAAAGAAATGCAAAAAAGCAAAATGGCTGTCTGGGGAGG
CCTTACAAATAGCTGTGAAAAGAAGAGAAGCCAAAAGCAAAGGAGAAAAGGAAAGATATAAGCATCTGAA
TGCAGAGTTCCAAACAATAGCAAGAAGAGGTAAGAAAGCCTTCCTCAGTGATCAATGCAAAGAAATAGAG
GAAAACAACAGAATGGGAAAGACTAGAGATCTCTTCAAGAAAATTAGAGATACCAAGGGAACATTTCATG
CAAAGATGGGCTTGATAAAGGACAGAAAGGGTATGGACCTAACAGAAACAGAAGATATTAAGAAGAGCTG
GCAAGAATACACAGAAGAACTGTACAAAAAATATCTTCATGACCCAGATAATCACAATGGTGTGATCACT
GACCTAGAGCCAGACATCCTGGAATGTGAAGTCAAGTGGACCTTAGAAAGCATCACTACGAACAAAGCTA
GTGGAGGTGATGGAATTCCAGTTGAGCTATTTCAAATCCTGAAAGATGATGCTGTGAAAGTGCTGCACTC
AATATGCCAGCAAATTTGGAAAACTCAGCAGTGGCCACAGGACTGGAAAAGGTCAATTTTCATTCCAATC
CCAAAGAAAGGCAATGCCAAAGAATGCTCAAACTACCGCACAATTGCACTCATCTCACAAGCTAGTAAAG
TAATGCTCAAAATTTTCCAAGCCAGGCTTCAGCAATATGTGAACTGTGAAATTCCAGTTGTTCAAGCTGG
TTTTAGAAAAGGCAGAGGAACCAGAGATCAAATTGCCAACATCCACTGGATCATTGAAAAAGCAAGAGAG
TTCCAGAAAAA

>ENSBTAG00000037605|hg19
TCTGGACCCTGATTTCCTGAAGGTGCAATGACGGTAACTTTTAGATTTGCTTAATATTGAGAATGCTTCC
AGGAATCTCTATTTCTAGGCCAATCCCCTCCCCCTCCACCCCCACCCCTCCAATTCCCTGCTCCCTAACT
TCAGTCTCCGTATCCTTCAACCGTGCACTCACCCACAATGTCTTCACTCCCAGAGGGACCCATCATGGTG
GTCAGGGCGAGGGCCCCCCAAATCAGGGCTCTGTTCAGGATCATCCTGTTCTCAAGGTGGTCTCAGCAGT
TGTTCTGAGTTGCAGAGCAATGATTGTGGGTTGAGTTGAGAGGGAATTCTAAAGATATCCAAGCCACACT
CTGTCAGGTTAGGTTTTGACCAATCAGAAAAATCCTCTATGATGACACCTCAGTTGCCAGATGAAGACTT
TGTACTAAGAGGCCTGGAAGGGACTGGGAAATCCCCTGGTTCTGATGCAGCCTCCACTCAGAGTGGACAA
GAGGGATCTGTCTATGAAAGGTGGGAGAGTACTAAATCAGAATCTTCTCATTTATGTGTGTATGCCTGCT
GGTGATGAGGGGTGGGGATTTCTGAACAGCCTCATGGATAGATGAAGATACAGCTAATTTGATGTCCAAC
AGGGCTATAGCTTCCAAAGAAATCTTCCCTGAGTTGTTCCCCCTCCCTGGCAGGTCCCATTCCCTCCCCT
TTCCATTGACTCTGTATGTAGGTTAACTGTCTTTCTCTTTGTTTCCATAGTGATATGGAATTCTCTAAAT
ACTGAATTGATCTAAGTGATTTCTGCCAAGTGGTAGCCAAGGAATACCTTTGCCTTTTCTACTTGTGGAA
ATCCAGTTGCGTAAATTATTCTTCTCATGCTCTCAAAAGCTCCAGTATTTGCACTCAGGGGAATAAAAAG
ACTTGTAAGGTCATATAGTGCTGCCTAAAGTAACTGCGTGATGTTTCATTTGAATTTTTCCCCTGCCTTG
AGTATTCTTTATGTTCTATATTTTGGTGAACCTACCTACATTTAAAGGAATTTGGGGATCATTGATTACA
TTAAAGGACATTCTTGTTGACTTGCTGTATAATTAAGTACATCAACTCAAATACTGAGGGTAGAATTCAG
GGAATTGTCCTGACTTAGAAAATTAGTTTTCTTCCTTTACTCTATATTTTAGAATTATTTATCCTGCCAT
ATCAAAACTCCAGATATTTGTGAGGGACTAAGGACGAAGCTAAAGTGAAGTGAGTGAAGTAAAGTCGCTT
AGTTGTGTCCGACTCTTTGCGATCCCCTGGACTGTAGCCTGCCAGGCTCCTCTGTCCATGGAATTCTCCA
GGCAAGAATACTGGAGTGGGTTGCCATTTCCTTCTCCAGGGGATCTTCCCAACCCAGGGATTGAACCCGG
GTCTCCTGCATTGCAGGCAGATGCTTTATCCTCTGAGCCACCAGGGAAGCCCATGACTTTAGACCTTTGA
CTTTAGACCAAGCTAAAGTCAAAGACAACTTTTAGAGGGGAAACTTATTTTTTCAAAATAATTTATGTTT
GTGTGTGTGTTATGAAAGCCACAGGTTAATGGGGGTTCCATTTGGAATCAATCACCCTGTCCCTGTTAGA
TGTTCCTCAGTTTCTCAGCACTGTCTTATCTCACAGTGACAAAGACTATAAAGAAGAGTGAGACAGAAAA
AGCCCTGGTGTCTAGAGCTGCTTGTAATTAGGCCTTGTTGTTGCCCGGGGCTGGCCTGCTACTCACAGCT
AGGTTTTGGTATTTTCTTGTTGAACATAAGCAATGTTGTACAACAGCATCAGACAGCCATTCTGTGACCA
GGATGGATCAAAACAGAAAACAAGGACCCTCAGAAATCATGACACAGACAAAATATGAACAACAGTGTCC
AAGCATGCTTCTGTCCTAGATAATATGAATGACCTCTGCTTCTTTACGAATTACAGCTTTAGCCTCAGTA
CCTTCTAGGTAAGAGTTACTTAGATGGCTGATTATAGAATTACCCCACTCCACCACCTATTACAATTCCT
GACAGAATCTAGTTGAGAACAAAGTCATTGTTTCTTAAATCATCCTCAAAATCACCAACACGAGCCTAAA
TCCTATAGTAGTTTAAGGGTCAGTTTCATGTGTTGCCAAGGCTATGGTCCTCAGTTATTCACTCAAATAC

TTATCTAGGTGTTGTTCTGAAGATATCCTGTACATGTGATTAAAGTCCATAATCAGTTGACATTAGGTAG
GAGAGGTTATTCTAGATAACCTGGGTGAGCCTGGACCAATGAGTTGAAAAGTCTCAAGAACAGAGCGAAA
TTTCCCTGAGGGAAAAGAGATTCCACTGTGGGCTACAGCATCAGCCTGTGCTGAGAGTTCCTGTGCTGAG
AGTTCCAGCCTGCCTTTCCTGACAATAGCCCACCTTATGGTCTTGCACTTCTCTAGTTCACCCTCAATTC
AGTTCAGTTCTCAGTCATGTCTGACTCTTTGCAACCCCACGGACTGCAGCTTGCCAGGCTTCCCTATCCA
TCACCACCTCCTGATGCTTGCTCAAACTCATGTCCACCAAGTCAGTGATGCCATCCAAACATATCATCCT
CTATCATCCCTTCTCCTCCTGCCTTCAATCTTTCCCAGCATCAGGGTCTTTTCCAATGAGTCGGTTCTTC
GTGTGAGTTAGTTTACCCTCACATTGTGTAAACTAATTCCTTGCAATAATTCTCTTAATAGATCTCTCCA
AAAAGTTCTGCTTCTTTGAATCCAACATCATCACACGAAGATACCCCATGATTCTCCATGGTGAGTTCAT
GAGCAATAAAACCAACTCGTTCATCCATGGATGTATTTCTGATGGACTTTGACTGAAGACATTAACAGCA
CTTCATTATATTGTTGTGAGGAACATACATATACTGCACCTGGAATGAAAAATTCCCAGGAGTTTTTAAC
TATACTTGTAATACATTATGGAGAAGGCAATGGCAAGCCACTCCAGTATTCTTGCCTAGAAAACCCCATG
AATGGAGGAGCCTGGTAGGCTGCAGTCCATGGGGTCGCTACGAGTCGGACGCGACTGAGTAACTTCACTT
TTACTTTTCACTTTCACGCAATGGAGAAGGCAATGGCAACCCACTCCAGTGTTCTTGCCTGGAGAATCCC
AGGGACGGCAGAGCCTGGTGGGCTGCGGTCTTTGGGGTCGCACAGAGTCGGACACAAGTGAAGTGACTTA
GCAGCAGCAGCAGCAGTAATACATTAATATTAATATCATCTCCATTTGACAAGGAGAACACTGAACTTCA
TAAAATTGAAA

>HLA-DRA|hg19
GTGGCACTCTCACTGCACTTGGGTAGAACTTCCGTCAAGCTTCTGTGAAACTGATGTCACATGTGGGGAA
ATAACACAACATATTCAGCTGCAGCGTGGGCTGTTCAGCCCTGGGCGAAGCAGCAACGGAACTGCTGATA
ATGGTCAGCAGCCAGAGACCCATACTTGCATATGCTTAGCCTTGTAGGCTTGTCTGGAGGCCAAGAGAAA
AAACCAAGAGCGATGATCAGACCCTTAATCACACTGCACAAGGAACCGGAGCTTCTGCTTTATACTGGTT
CCCACTCAGAGAAGGTGGAAGGATACCGTTTCGAGCAGGAAAGCCCGTGCAGTGCTGGGCAGGGAGATAA
GGGTGGAGGGGAGCTGTATGGCTGCCAATGACCCCTATACCCACTCAGCCTGTTCAGACTCCATATCTGA
AATCACAAAAATGGTCTCACCAAAGGTCCAGTACACAGCACAGAAGATCCTGGAGAAAAAGTGGGGGCCC
TAGAAATATGCTTTTCTTTTTTTCTTTTATGAAAGGTCTGTTTTGTGAAACATACATTGTTGTGAGTTGC
TCTGAGAAAGAGGCCTGGTTCTTTGGATCATATTCAAGTTCTGGAGGCATCTCTCACCCTTGATAGTCTG
ACTACATGCTCAGCAGGCCTTAAGGTGATGGCTCTGGGAAGTTGGAGCAAGTTTGGTGCAGAAGTCAGGG
TCCATAAACCCATAAAACTTGTCTCTCAGATTGGCTCTGATGCACTGGGTTCAGGCCAGGGCTGCAGGGG
GCTCTGGTACTTTCCTGACAACATTCTTTTTTGCCATTCCTCTTCTCTGTTTAGATTTATTGCTCCTCCG
TCCCTACCAGTCTTACTCTTCCCTAGGGAAAACTGCGCAAGGGACATGTGAGTCTATTTATTTAAAATTA
CATGCAATAGGAAAGACAGTCCAGGAAACAGGCCAGGTGTCCATTACAAGTGACAGGTGGAAAAAATAAA
GTACAGCCTCACCACGCAGTGCAGTGAAGCTGTTAAAAGTAATCATGAGGCTGCAGGGGGTGGGGAGGGG
GGTGGCGGGGGACAAGCAGAGAGGCCCTAGAATAAGGGACTTTTGGCCTGGGATTTGCAGGTACCAGTAA
CTGGAGCAACAGAAGCTATAACTGAAAGGCAGAAGCTGAAATTAGTTCCCAGAAGAAATAATGTCACTGG
ACCCTAGAGCCAAACACACAGCTTTCCATGGAGAGCTGTCTAATTGCACCAACCGTTCTTGGATTTCCCC
TCCCTCTTCTCCTCTGTTGAGGTAGCTGTTTCTTCATGATAAAAAGATAAGAATGGTTTCTCACACCTTA
TTTATCAGGGTCAGAGGAAAAAAAAAATCTAACCATCTGAGCAGCTGTAGACAGTGTTCTCCCCACATGG
ACATGATCCTCCTTGGTGATGTTTCCTGCTTTTATATCTTCTGATTATGTCAGCTACACACCAGTCATCA
TGGACCAGAAATTAATTTCAAGCTGATTTTATTTAGGCAAGAAGTGAGGACTGTGGGACTGCTGAGTTCT
TCCGAGAGCTGGTCACCTCCATCAGGGGATTATGGTTATCGTGCTGCTCACAGGGCCTGGTCAGCAGAGA
ACACTGTCAGAAAGTGACTGGTTACAACATGAGAGTGGCTCTAACGTCCTCCACAGTGGCCAGCAAGAGA
AAGACAGCGCTGAATATGCCTCACTCCAAAAATCTAGGTGAGGGCCAAAAGGGCACGTATGTTTGTGAAT
TTAGCTATACTTTATATTAAGATATATAAAGGTATTTATCCATTCAATGGTTAGCAAATGTTTTGCTCTT
ATGAATAATGTTATTATGAACTTGCAGCAGTTTTGTTAGGTGTGTATTTAAGAGTAGAATGCAGAATTAT
AATGCATGTACATATTTTCAATGTTACTATGTAATTTTGACTCTGAAATGACTGTAACAACTTATACTCC
TATCAGTTGTATATTGGCAGAAACATTGTTCCACACCCTTGCTTATGACTGCTATGTTTTAACATTTTTA
TTATTTTTTCAGGATGTTTTGGTTTGCATTCCTCTGATTAGTACTGAGGAGGTTGAATGACTTCTCATGT
TTATATATAATTTAGATGTGTCTGTAGAATGTTTCTTCTTGCCTTTGCCACTTTGTTTTTCTTGCACATT
ATAAATTTTACAATTGTCTATTTCTACAGAGCAAACCCGCTAGGAGCATTGATGAGGATCGTGTTGAAGA
GAATTGGTGTTTCAACAACACCCCATTTATCCAAAACCAAGCATCTTCTATCTCTCCATGTATTTGAGAT
TTCACTAGTCTCTGGCATCAGATAGGCTTCAAAATCACTGCAGATGGTGATTGCAGCCATGAAATTAAAA

GACACTTACTCCTTGGAAGGAAAGTTATGACCAACCTAGACAGCATATTAAAAAGCAGAGACATTACTTT
GTCAACAAAGGTCCGTCTAGTCAAGGCTATGGTTTTTCCAGTGGTCATGTATGGATGTGAGAGTTGGACT
GTGAAGAAAGCTGAGCGTCAAAGAATTGATGCTTTTGAACTGTGGTGTTGGAGAAAACTCTTGAGAGTCC
CTTGGACTGCAAGGAGATCCAACCAGTCCATCCTAAAGGAGATCAGTCCTGGGTGTTCATTGGAAGGACT
GATGTTGAAGCTGAAACTCCAATACTTTGGCCACCTGATGTGAAGAGTTGATTAATTGGAAAAGACCCTG
CTGCTGGGAAAGACTGACGGCAAGAGGAGAAGGGGACGACAGAGGATGAGATGGTTGGATGACATCACCG
ACTCAGTGGACGTGGGTTCAGGTGGACTCTGGGAGTTGGTGATGGACAGGGAGGCCTGGCATGCTGCGGT
TCATGGGAACACAAAGAGTCAGACATGACTGAGCAAGTGAACTGAACTGAACTGGCATCAGAACTTTGTA
GTTTTCCCTGTTGAGACATCATATATATTTTGGTTAAACTCATAACTATTTTATGCTTTTATTGCTATCG
GAATTACTTTTAAATTTTATATGCAGTGTTCTTCTGCTAGCATAGCAGATCACAATTGTTTTTTCTTTTT
ATATTGCCATCACTAAACTCATTTTAGACATTTTAATAACTTTCTTAGGGTTTTCTATGAAGGTGTATAA
AATGCTTCCCAGGTTGCAATGTTCATTATTTGTCAAAAAGAGTCTGAAATGAGGACTTCCTTAGCAGTCC
AGTGGTTAAGACTCCATACTTATACTTCAGCATCAAACCAGGTTTGATCCCTGGTTGGGGAACTAAGATC
CTGCATGCCAT

>ENSBTAG00000034945|hg19
TCGCCCATCACCAAGAAAATGTACTACTCACACAGACTGTTCACGTCTTCTAAGTTCTTCTTAGGGGGAT
GAAGGCATCCTGGAAGTTCTCACTGACGCTGCCACAGGCACCAGGAATCCATGCTCTTTGTCGTCCCTCC
AGGTGCCCCTCTGTGGGCTGCCTGCAGGTGTGTCCTGTGATACTTGCTCCAAGATGCCAGGGGAAGACCA
GACGTTAGAAACCTTGGGGTCCTGAAGGAAGCCTTAGGTTTTTTTTTTAAAAGGTTTCAGAGAACTAGAA
GAAAGATGGCCCCTGCTCAGGAAATTCTCAGCAGTGCGATTCTCCTCTATCTTGCCAACATATTCAAAAC
TGAAAGCCAAATAAGATAAGAAAAGAGAACTTTGACCTTGCATGAAATAAATCCATCTAATAATTGCACA
CTTCCAAGGAAAGGGTCAAAATCCAGAGATTTCTTTTTCTCTAAGAGTGTATTTATTCAGGCTGCTGCTG
CTGCTGCTGCTGCAAGTCGCTTCAGTCGTGTCCGACTCTGTATGACCCCATAGACGGCAGCCCATCAGGC
TCCCCCGTCTCTGGGATTCTCCAGGCAAGAACACTGGAGTGGGTTGCCATTTCCTTCTCCGATGCATGAA
AGTGAAAAGTGAAAGTGAAGTCATTCAGTCGTGTCCAACTCTTAGCGACCCCAGGGACTGCAGCCTACCA
GGCTCCTCCATCCATGGGATTTTCCAGGCAAGAGTACTGGAGTGGGGTGCCATTGCCTTCAAAACATAAA
TATTAGAACTTGACTGGCTGAGTTAAAATCTTGGTAAGGTCACATACAAGATCATGGTTTTGATCAAGTT
ACATAAGCTTTCTGTGTGCCTCAGTTTTCTCATCTGAAAATGGGTTTAATAATAGTACCTACCCTAAGCT
GTCATGAGGATTACATGAATTAATATATGTAAACCACTTAGAAGAGGATCAGTAACTTTTACGTTCTGCT
GTGTGGCTACATAATTGATTTGTGTTTTGAAGATCCTTCAAATCCTACAAGTTTTAGAATAATTGCCTTT
CTTGCCTAGAATGACAGACATTCTGAGTTAGAGTCTGAAAAAAATTTAAAAACTCTAAATTACATTTTTG
ATATTCTGGATATAAGATTGAACATCTCTGTATGGATTGTAAGAGATATAGTAACACGAAATAAACAAGT
ATAAACCAGACATGGGGCTTCCCTGATAGCTCAGCTGGTAAAGAATCTGCCTGCAATGCAGGGGACCTGG
GTTCGACCCCTGGGTTGGGAAGAGCCCCTGGAGAAGAGAAAGGCTACCCCTCCATTATTCTGGTTTGAAG
AATTTCGTGGACTCTCTAGTCCATATGTTCACAAAGAGTCAGACACCACTGAGCGAATTTCACTTTCCCT
TTCACAAACCAGACATAGCCTAACAAAGTTTTAAATATGGCAATATTAATTTTAAAATAAAAATAAAGTT
CAGAGAGAAATCATTCGGAGAAATGAGATGACCCCTTCAAATAGTGACATTCATCATGAGCACATAACTT
CCAAGGACTGCATGGTGCTAACAGCCTACAGCAGAATTAAAAAATGCAAAATCAGTTTGGAACTCAAAAT
AGTTTATAACCACAATTAATCAGAAATGAACAGAACCATTCCAGAACATATAAATGAAGTTAGAGAGGTT
TTACATACTATAACAACTATGCCACATAAAATCTTTTACTTTTTGACTTGGTGAGATTTAATAATTTCTT
TCAGTTTTTAATGAATAGAAATCCATTAGTAGAACGACCCAGTATGACACTCACCTAACTCCGTCTTCCA
CTGTGCTTAGAGTGCCAAGCCGCCCCCAGATCTATCTATAAAATTAATACCAAATTATCTGCTTCTCCCA
AGTATTCTCTTTGAAACTTGCTTAAGTATCTAATCTCACTAGAGATTTGGATTTGCATTTCCTAAATAGG
CAGGGGTTTGACATGAGTATGAATAATGCTATCATACTTTTGGAACACGATTAAAATGTATAAAGGCACT
GCTGCAGGAAAACCATGCAGTCATCATGGAGGCAAATACTCTATTGGAAGAAAGTATCAGAAAAAGGAGA
TTAAGTCACCCCATGATTCCTCCATTTCTACGTGACTAATGTCAGGACATGCATGTGTGCTCAGTCTTGA
AGTTGTGCCCAACTTTTGTGACCCTGTGGACTGTAGCCCCCAGGCTCTTCTGTCCATGGGATTTTCCAGG
CAAGAACATTGGAGTGGGTTGCCATTCCTCCTCCAGGGGACTTTCCCCACCCAGGGATCAAATCCAAGTC
TCCTGAGTCTCTTGCATCGGCAGGAGGATACTTTACCACTGAGCCACCTAGGAAGCCCATACTGCCAGAA
CACATTTGCTAAATCTCCTATCTTTCACTTTACTCCAAGTCAGATAACAAAGAGTGGGAGTAAACAACTA
CGGGATGTCGCCCAGCAGTATTTGTACCTTTTCCCCTCTCCAAGCCCTTGTGTTCAACCTGCAACCTTCA
CTTCTCTTATATAAGAGAAGTTTTATAAGAAAAAAGAAGCAACTTCTGATTCCTTGCTTGTCCAGAATTT

TCTTTTACACTTAATGCTAGTTTTGTTGTTTAGAAAATTCTAGTTTCTACTCAGGAAAGTGATGAAATAA
AATTTATATTTTAAAGCAGGACAAGGAAATTTAAGCACAAAATTCTCTATTTGTCTGTTTGGGTCTCTTT
CCTGCCCCTGTAATATGTACTGTGCTTCTGCATTGTACATTAACAAGACCTCCTCAAAGGTGAGAATGCT
GGCTTGACTATATGATAATTTTTTTCCTTTCTTCTAATACTAGCAATGTAACTTCTTAAAAGAACAGCAT
TCTTTCCTGACTCTGTAAGGAATCGTGGAGACTTGCTACTTGCTTTGTACATGCTTACCTGGACTAGCTC
TGGTGAACTTTCATGTAAGATGTCAGTATGTCATTTTGATGACAATATTTTGTCTTAAAAATGTGTGCGG
TGGTGCCTCCAATTAAAATAAATAAATTTATATTAAAAAAAAAGCACATGGGGGAAAAAAAAATGTGTAC
GGTGTACTTTCAACTTCTAACAGGTGGGAGAGTTCTCAGAGCTTCTGAATCTGTCTCTGAATTATAATCC
TCAGTTGACTTGAATGAAATTCTCCCTTTGTTTTTTTCACTTGATTGTTAATTGAATTTCTGTTGACAAA
AATGAAGACTCCATTCAACTGTTTTCTAGTAATTACTATTGAATGACATGTATGATGGAATCTGATTTTT
TTTTTCTTTTG

>GSTA3|hg19
GCTATTTCATTTTCATAGGTGTATCTACTCTGCAGAAAAAAAATACCCATTCCTTTTGTTTGATATAAGCA
AGTTTAGAAATAGTCATTACTGTAGAGCAGAGACTGGAACAGCATTTTAAATCAACTATACTTCAACAAA
AATAAAAGTAAATAATAGTCATTAAGTTAACCATAAAATCACAAAAATTTCTGACTACAAGTGACAAATT
TGGTCACAAAATAAACCATCCAACCAAGCCTAAAAAGAACAGATAAGAACTTCACCACTAACCTTATCTC
TTCCTGGATCTCCTCTCTGGGGTGCTTCAGGGGGATTACGTGAGGATTTATACCCCTTTCTTTTCTAATT
GAACGCTCTACCCCTTCAAAGGATTGGCCAATGAGCATTCGAGTTTGTACATACCTCTAGGGCTTCACTT
TACATCCATTGTTTGGATGTTAGATCAGGTTACTCAAGTAAGTTGAAAGAAAGATTCCACTGGCTCATGC
AACAAATATTTACTGAGTACCGCTCTGTGCCTGGTACTGTTTTAGTCCCAGGGTCTACGATGTTACATAT
GTCAGACTCAATTCCTGCATTGGTGGTACTGCTTACATTCTAGTTGGGGAAAACAGGCTGTATGAATATA
TAAGCAAATAAGTGCGAGGTGTTCAAAATAATAATGCCAACAATGTATCAGATGATTATAGCTTACGGAT
AAAAGAAATCTATGACAACAGTGTTGTAAGGGGTAGGACGGAGGAATTATAAGCAGTTATAAGGTACCTG
AGAAGTCTGTATGCAGATCAAGAAGCAACAGTTAGAAGTGGACATGGAACAACAGACTGGTTCCAAATAG
GAAAAGGAGTACGTCAAGGCTGTATATTGTCATCCTGCTTATTTAAACTTATGTGCAGAATACATCATGC
GAAATGCCAGGCCGGATGAAGCACAAGCTGGAATCAATATTGCCGGGAGAAATATCAATAACCTCAGATT
CACAGATGACACCACCTTTATGGCAGAAAGCGAAGAACTAAAGAGACTCTTGATGAAAGTGAAAGAAGAC
AGTGAAAAAGTTGGCTTAAAGCTCAACATTCAGAAAACTAAGATCATGGCATCCGGTCCCATCACTTCAT
GGCAAATAGATGGAGAAACAGTGGAAACAGTGACAGACTTTATTTTTGGGGGCTCCAAAATCACTGCAGA
TGGTGACTGTAGCCATGAAATTTAAAGACGCTTACTCCTTGGAAGAAAAGTTATGACCAACCTAGACAGC
ATATTAAAAAGCAGAGACATTACTTTGCCAACAAAGGTCCATCTCGTTAAGGCTATGGTTTTTCCAGTAG
TTATGTAAGGATGTGAGAGTTGGACTATAAAGAAAGCTGAGCGCTAAGAATTGATGCTTTTGAAGTGTGG
CGTTGGAGAAACACTGTTGAGAGTCCCTTGGACTGCAAGGAGATCCAACCAGTCCATCCTAAAGGAAATC
AGTCCTGAATATTCATTGGAAGGATTGATGCTGAAGCTGAAACTCCAATACTTTGGCCACCTGATTCGAA
GAACTGACTCATTGGAAAAGACCCTGATGCTAGGAAAGATTGAAGGCTGGAGAAGGGGACGACAGAGGAC
GAGATGGTTGGATGGCATCACTGATTCGATGGACATGAGTTTGAGTAAGCTTCAGGAGTTGGTGATGGAC
AGGGAAGCCTGGTGTGCTGCAGTCCATGGGGTCGCAAAGAGTCAGACACAACTGAGCAACTGAACTGAAC
TGATAAGGTACCTGCATCACCTGTGAAGTGATACAGTGTTGTTGGAAGAGGCCTTAGTGTTGTTGCATAT
AAATATTACAAACTTCAGAACAACCACTAAAACTAGAAAAATACAAACTATAATTGACATGCTAAGAAAG
GAGAAAAAATAGAATTATATAACATGCTCAATTAAAACAGGGCAGAAAAAGAGTAGAAGACAAAGCAAAA
ACAGTGGGGGGTGAAGAACCAAGGCAACAAATTGAAAAGAGTCACACAAAAAAGAAAAGAGTAGAGATTA
ATGCAACCACATCAATAATCACTTTAAATGTGAATTGCCGTGGTCTGTGCACAAGTTGGAAAAGAATCTC
TAGACACAGATTCTAGAGGCAGAATGAGAGGAGTATAGAGTTTATTAAAGCAGGAGATACTGTTAGAACA
GCGGGCTGGCTCAAGGAAGAGCTGACTCCTTTCATGGATTAGTAGCCAATTTTTATAGTCTCAAGACAGA
AAATTCCTGCTGGAAGGGTAGCATTAGGTGATTAGTTAGGGTGCTATAGGGTGGGTAATTGGGTGAGTAT
GTTTTCCTAATATGGGGTAAGGGAGCTGGCCAGTTTAGATCAGGGAGGCTCATGGCAATGGTTACTGTGG
GACTTGGTCAGTTCGGAGATGACCTTGGTGCAGGGCTCCACCTGTGTGGCCTCCATATAAGGCTTTACAC
CTGTGACCGTGATACAGAGCTCCATATGAATGTTGTAAACATTCATGAGTGGATTTTAAAACAAGACCCT
ATTTTTGGTTTTCTGTAAGAAACCCACTTTAAAGACACAGATTGATTAAACAGAGAGATTGATGGTGGGT
GTCAAGTGCTTTGAAGAAAATAAAATGATGAGAACATGAGAGATGAGATGGATGATGAGAAAGAAACATG
GGGTGCGGGGTTGGGCCACCGAGTTCATGATCAGAAGATTCCCTTAGTGGGAACGTTGAAGCTGATGGAA
CAAGGATCCCGCCACGGAATCTTCTGGGCAGACAATTTGGGTAAAGGAATAAAATGCACATACAACAAAG

TGGGAACACCAGCAAGTACGAGAGCCTGGGCTGGTAGCCATTTGCTGCAGAGGCTGGAATTCACCTTCTG
CTGTTGCATCCACAGGCCCTTCCATACCCACCCTCCTCAGGGACCCAGGCAGGAGTGTCACGGGTTCCTT
TGGGCACCCAGTCTGCCAAAGCCCTCTAAGCAGCTGATATTGGGTCCTGGGCTCATTAGGTGCTGCACCT
CCTCCTCTGACAGACACCAGAACCCCACACCGTTCAGCTCCTCCTTTTGTCCCAGAGGACACTGGAGCCC
CCACAAGTGCCCCGTTTACCCCCGGGGTGCCCTTTCACCCCATTGGCTCAGCTGCTGTGGGGTGTAGCCC
GTGAGCCTCATGGGCCCCCTCTGCCCTGTCTCCCTGCCTCACACGTCTGATTCCTTCCTGAACACAGCAT
GCTGACATCTTTGAGGACAACCGTCTGCTGCCTGACCTCCTGGCTCTTTTACCAGAGTCTGGCGGCTTGT
GAAGATGGGAT

>GSTA4|hg19
AGGGAGACAGACCTGGCTCCACTCGGAGTGACCAGAACCGCACAAAGGCGCCCAGCGAGCGGTCTGCAGC
CGGGGGACCCGAGGGCGCCGGTGGGCGAGGCTCCAGAATATTTCACCAATAGAGACGGCAATATTTAGCA
TGCTCCACCCATCTGCAAGGCATGCTGGATGATACGCAAACAACTCCAAATCAAGCCCGTTTTAACAGCA
ATTTTTGCAATTTGAGGGAATAATTTTTAAGTGTTAGTTAGGTTTGGACCTTAGTTTAAGATATATTTTA
GAGATCCGGCATTTAGAAAACAAAGAAAAAGTCGACATATCCTTAAAGACAGAAGTTTCTTGTAGTTTAT
ATACTGCGCGAACTACCATGGTGTTTCGGATGTGAGGGCGATCTGGTTGCGACATCTGTCACCCCATTGA
TCGCCAGGGTTGATTCGGCTGATCTGGCTGGCTAGGCGGGTGTCCCCTTCCTCCCTCACCGCTCCATGTG
CGTCCCTCCCGAAGCTGCGCGCTCGGTCGAAGAGGACGACCATCCCCGCTAGAGGAGGACCGGTCTTCGG
TCAAGGGTATACGAGTAGCTGCGCTCCCCTGCTAGAACCTCCAAACAAGCTCTCAAGGTCCATTTGTAGG
AGAACGTAGGGTAGTCAAGCTTCCAAGACTCCAGACACATCCAAATGAGGCGCTGCACGTGGCAGTCTGC
CTTTCTTTTGACTATTTTACAATTTTTCTATTAATATGTAACATTATTAGAACTTAATTTCACACTTTGT
GTGTGTAAAATGTATAATGAATGTTTATACATACTTACATATAATACGGAGAAGGCAATGGCTCCCCACT
CCAGTACTCTTGCCTGGAAGACTGCAGTCCATGGGGTCGCACAGAGTCGGACATGACTGAAGCGACTTAG
CAGCAGCAGCACATATAATACAATTAATACATAATTGTATATAGTTTATTCTTCATGTATTTTTAATTTA
TATATATATGTATTTCCTCTTTCCTCCTTTCTTCCTTGACTCAGTGAATCCTCTTGAGCCTCTCCTTCAT
TTCCTACACTTTCGACAAAACTGGTTTAATCCACAGCACCTTCTTGGATGGGAGGAAGTGGGGGCCTTGG
GGTAGAGGTGTCAGTGGGGCTACAACTATAATCTGGGTGGTAATTAAGCTAGAGCTTTAAAAAATATTTT
AAGACTATCAAAATTATTATAGAAAAATTCACACAAATTTTAAGGTTAAAAATGTATGCCTCCAAAAGGC
CTATTTCTTGTTTCCTGAGGCAGCCTCTCCTATTTCGCAGACTATAGATGTTGATTATTGTTTTCACCCA
GGAGGGGTTCTTGAAATTACCTGGCTTGAGAAGCCTGTGCTGCTGATCAATTACAGCAACTGACAAGATC
AGATTTTATTTTCTGTAGATTTCTCTGGCAGAAACAGGGAGAATGAACTGGGACTGTTCAGGGTGGGGAA
AGAGAAACCTGGAAATATCCGTGTGTATTATAGTATAGCATCATAAATTAGAAGAAGATAGATAAGAAAA
GGTAAGATATTAAGGGGGTGGATGGGAGGAAGAGAAGTCTAAACTGATGTCCTCTCCCTAAGCACACACA
CACTTTCTTTTCCATCCTCCTTATTTACTTTTCTGAACATTTATTGTCATATAATATACTATATATTTAT
TTTGTTATCTATCTGGAATTTTGATAGAATGTAAATTCCAACAGGTTAGGGAGTTTTCATATTTCTTAAA
ATGTACCTTTGTATACCCTGATCCTAGAATAATGCTTAGGGCAGAGAAGACATTCAGAGAACACTTGTGG
AAACAAACAATGAACGATGCTTTGGTTTCTAGCTCCATAGAGATTTGGGGGACATAATGAATGGGTTTGT
CTTTTATTCTCACAAGCCACACACTTGTAAATGTCACTAGACAGTTGAAAGGCCAGTGGGAGGTTAGGGT
TAGCAGATATAAGCTGTTACATATAGAATGGATAAACAACGAGGTCCTACAGTACAGCACAGAGAACTAT
ATTCAATGTCCTGTGATAAACCGTAAGGGAAAAGAATAGTAAATAAGAATTATGTATAACTGAAGCACTT
TGCTGTATAGCAGAAATTAACATAATACTATGAATCAACTATACTTCAACTAAAAAAATAAAATTCTATT
TTTTTTTTTTTAATTTAAAAAGAGGCCAGTGACAACTCAGAAGAGAGGACTGAGATGGATTAATCTGGCA
GTAAAACAAAGTATCCAGAGCAAGTGTGTAAAGTAAGAAGAAAAGCAAACAAGAACAGCTGGGCACCCCT
AGAACAGGAAGTCATTTTCAAACAGGAATAATAGTACATATTCAATTTCATCTTTCTAATTCTGACTTAA
CACAGACATTACTCAGTTTGACATAGGTAGGCAAGCCATATTTTTCAAACCTGCTGGAGGTGGTGATCTT
TATCAATTTTTTCCAACTCACCTTTCTGCAAAGATTCACTTTCATTGAACTATACAGGAAGAGCACTATT
ATCAACTTTGTTGATCAGTTAAATCTCTATATCTATTTGCTTCTTCTAATTGGTTGAACCATATCAAATT
GCTGGTATTTTACTATTTTCTTCTTTAAAAAAAAAAAAAGGACACTTCTATGTCCAACCTAATAGAAGTTA
CATCATCCTAGCAATCTTGTTTACCTGAAAGAGAAACTAAGGCAGTGTCATAAGACTCTCATTAAACATG
CTTGTGGAGAAATAAAAAAAAAATCCTGTTTAATTCTGCCACTCTATGAAAGTACCCTTAGGTCATTCTGC
TCCTTGGAAGCCCCTGAAATTATTTTATTCCTGGAGACCCTTGGAGTTGCTCTCAACCTTGGACAGTCTT
ATTCTCGGTGTCTTTCTACCTACTGGATATATATATTTAAATTCAGAGGCCATGGGAACTTGTCTCAGCC
TTTGACCCTTCCCCCCCACCCACTCAGTAGCAGGTAAAATTGCCTTAACAAAAATCAAGAGCTTTAAAAA

TCAGTTTAAGAGGAGCTTTTCTAAGTAAACATTCCAGAAAGAGAGTCACAATGGTTTAAATTCAGTCCCA
GCTGTTCTAACAGCAGGCTTTGATAGCAGGAAGGAAGACTGAGAGCTTGAATCTGTGGGGCATCTGAAGG
GCTGAGGGGAGATGGGGGGTCATTAGACGAACGGGGTGATGACACACTCAGGTTGGGGGTGTTCTCGTGG
GCTAGGGCACATCTGTGGATTCATGGGGTGATGACACACTCAGGTTGGGGGTGTTCTGATAGGCTAGGGC
ACATTTATGGA

>CILK1|hg19
GACCCTCGCGGCTCGGGTCCATCACCCCCAGTGACAGGTCACCCGGCGGAAGCCCCTCATCTCCGACCCC
ACTTACCGGCGCCGCGGCACAGGCGAGGGCCCGCCCCTGCGGGGAGCGCGGTTCCTCGGAGGGCGGGCAC
GGGGTCGGCCAGGGAGCTGAAGCCGGGGCAGGGCGACCCCCGCTAGGACGGGCCGAGGGGTCCGCGCAGA
GACGCCCGGGGGCGCCGGCGAGGGGCGCGGGCCGCCCGGCGCATGGTGAGCAGCGGGGATCCGGCCGCTC
TCCAGCCTCCGGCAGGAAAACAAACAAAAGGCTGAGAGGCTTGTGGTGGGGACGACCGGTCGCTTACCCT
GGGAAAAGTTACCCTGCAGCTCCCCTCGCTGGCCCCGCGCGTCCCTGCACCGCGGCCGGACCGCCTGGGG
CGCCGAGCCCCGTGCTCGGCTCTCCACCCCGGCCCGCGGTAGCCCAGGGGCCCTGTGTCCCGCCTACCGC
CGAGGAGCTCTCCAGCTCCACACGGCCGTCTAGGGCATCAGGATGGAGAGATGCAGGCCGAGGATACACG
TTCAAAGGACATCCCATTACAGATGATCATGGTGGACACGCTCTCCTGGCTTTAAAAAACATATCTGTAA
TGCCACTGAAACGTGGTTAAAGATGGCAAATTGCATCATACTTAACCATAATAAAAAATATTTAAAACAA
AAACCAGACTGCGTGTTAGCATCCATTGGAACTTCAAGAGTTTTTATGTTGTGAGCAACTTTAGTTTGGA
AAACTGGATTGTTACTCAGGTGGAGAACACTCTAGGTAACGCTCTACAGATCGGTCTCAGGGTGTAACTA
GTTATTTGAAATTTAACACTGCTTAAACGCCATCACTTGTGTCCTTAGTTATATTGCAAAGGTAAATCAT
CCCTATGAGGCCTGGCTCTGAACCGCTTTGAAAAAATAAATTGCATCGAGGTCCTCGATTTAAATTCACA
TAATTGCCCCAAGCCAAGGGTTCTCACACTTTAGCTGGAGGCATCAGAATCACCCCCAAGAGCCTGTGAA
AACAGATTGCTGGGGCCTCTCCCCCAGAATTCTGGTGCTAATACTACTGGTCAGGAGAAACACATTGCCC
TAAACAACACAATTTAAATTAATAAAGCAGTAATCCTTTCTCTGAGTTTAGATCTCCCAGCTCATACCAG
TTTTCTCTCACTGTCTTTGTTTTTAAATTCTAAGAACTGAAAAAATTATTTCCAAAAAGAATCTATGCAA
AAGGCCCGAATCCCTCTTTTCCCTTGCTTCTGTTGAAGAAACAAGATACAAGACTGCCAAGCTTCCTTAG
ACTTCCTGTCCCAAATGGGAAAGGGTCATCTGTCCACTTGGCAATTGTATAACAATTATCTACATTCATG
TTTACCTACATAACACCCCAAACCATATGGCAAATGAGAATTGAAAGTCATTATCAATGAAAAGATATGG
AGACAGGAAGCAGCCTAACAAATCATATAATCACCCCTATGAAAAGTGATTTTTGAAGTATTTAAAATGA
TCAGTTTTAGTCAAACTCAGTTGATCAAGCAATTGGCAAATGGACTGTCCTGATCTACCTTTAACAAGGG
TCCACTGGGCTAGTAAGTGGCTTAGGTCAGTAGTCCTAGCCTAGGGAAAATTATAACCATGTGAGTATTA
CATAATATGATGAAATAATACAACCATGTAAAAGGGTCCAGTGGATAGAGTGGATACTTCTCTAAAATTT
TAGATGACAGCATTTTAATAAAGTAGATGCTCAATTTATTGTTACAAATACTAATTATTTTATTAAACTT
TGTTGTTCATTTATTAGAATAGAAATAATGTCATGAGCATTCAACCAGTTTAATGAAATAGACTAGTTCT
GAAAATATTTCAACACCCTATTCCTCATTTGCAAAACCGATCTTTTGTATTCATAAATCACATATAGCAT
TTCCTTCTACTTTTAGACTACATCTAGTCTTTCCCCCGTTTTAATTTCAAAATATGATTTAGAAGAGGCC
ATTGGGAGGAAAATCATTATTAAAAGACCTAAGCTCTACAGTCTCCTTTAAAAACTACAAACCGGTAAAT
TTTTAGTCTCCCTTAAGTAAGAATGTACTTTTTACAGGTGGCTGACTTAGGCAAATGAAAACATACCCAT
GAGAACGATGGATCCTAATATTAAAAGATAGTACTTGGGGCTTCCATAATATAGCTCACCATTCTGCACC
CCTCCCTTTCTTTTGCCTTTGTTTTTCTGCAGAAAAATGTACTGCATTACTAAGATCTAAATAAACAGAA
TAAAATAACGGATAAGAGATTTAAACTCTTGTGAATTCGTGGAATCACGAGTAGGGTCTTGAAAACCTGG
TGACTATTAGTAACCCACACCTAGTAGAAACCTCAGAAACTCCATAAATGTTTCCTTAAAGAAGGTAAGA
AGCTCCAAGGATAGGTTTACACACAGCATGCCAAAGTCAGCATTTTAAACAACGTCCAGTGTTGGAAAGT
AATATACCGCCAGCCAAGCCGGAATGCCTTCTGCGGAAGTGAAACCAGGTGTTTGGACTTTAAGAGGCGG
GGCCAGAACAGCGTGTGAGTACCGGCGCTCTACGTGGAACGGCTGACTACAAATCCCAGTGTGCCACGGG
AGCCGTCGCTGCAGTCTATCGGGGCCGTAGAGCCCCAGGGTCCTCCTCGCGTTGCATGATGGGGTTTGCA
GTGCGACTTGCGTTGCAGGGGGTCGGGGGCTAAGTTTTCTCTCCTGCTTTTTCCTGAGCGCCTCTTTTTC
TCAGCCTCCAGAAAAAGGTGTTTTTGAGAAGCTTAGTAATTATGACAGCCTTACGATAGTGGAATTCCAC
TGTTTCCTTGTAGTCCTGGATTGGGATTCTAGCCACTGTCGCCGAGAACCGTGTAGCGACCATCCCGAGC
ACCCAATTCGATACCTTAGGACCGCTTGCCTGAGGGTCTTAATTCGAAGTTTGAGCACCCCACTCCAGTA
CTCTTGCCTGGAAAATCCCAATGGATGGAGGAGCCTGATGGGTTACAGTCCATGGGGTCGCTAAGAGTCG
GACACGACTGAGCGACTTCACTTTCACGCATTGGAGAAGGAAATGGCAACCCACTCCAGTGTTCTTGCCT
TGAGAATCCCAGGGACGGGCGAGCCTGGTGGGCTGCCGCCTATGGGGTCGCATACCGTCGGACACGACTG

AAGCGACTTAGCAGCAGCAGCAGCAGCAAGTTTGCATAAGTTCACTGAACCGAACACTAGGTCGAGTTTG
GCCTTTGGACA

>GCM1|hg19
TACATTTGTCTCTCCTTAGGATTTCTGGCTTTGTTACATTTTTTTAATATATAAATTGACTTACAATAT
TAGATTAGCCTCAAGTGTAAAACACATTGTCATCACGCAAAGACAAGCTACTATCTGTTACCATACAATT
ATTACAAAATTATTGACTATATTTTTAATGCTGTATATTATATCCCCATAATTTATTTATCTTACAACTG
AAACTTTATAATGGTTTCCTCACCTCTGACTTCTGGAGATGTAGACAAAACTGCTTCAGAAAAATCTCCT
CTCCGATTCAGGTCCCAGCAAGTTCTGGTCATTTTCTACGCAGAGACTGCTGTTCCCTAATCCAGGTCAA
GGTACACTATGCTAATGAGCTCTGGCAATAATCCAATCAAAATCTAGATGCTCTCAGCTTGGGATAGGTA
AGAGTGACCCGGGAGCCACTTACCCAGCCCCAATTCTTCGCAGAATGCCAGCAATGTGATGAATATTCTA
TGGTACGCTTATGAACATTCCCCTTGTTTATGTCAAAAAGGCGTTTTAACAGAAATGGACATTGCTAAGA
TTCTCATGAGGAAGTCCAGATGTTTTCTTTCTTGTAATTTCAAAGTTTGGGCCCAAGGCAGACTGAGTAA
CGGTAGAATTCTAATTCCTTCACCATTTACCTCGGCATGCGTACAAAAGAGAAAAGACAGACAACCAGCA
AGATATGTCATAGTGATGGAATTCATTGGAATAGATGTTTAGCAGCTGAACAACAATGACAAGATGTTGG
TTAGCCAACACCTCCCAAGCTACTAAAATCACTAAAATGCCATCCTGCCCCTAACAAGGTTGCCCCATGA
ATTCTGAGTGTTTTAATTGCAAAGAATAATATAATCCTTTCTTCAAAAGTGTCCTAAGCAGCCAGGGTGC
AGCCCACATTGGTTCAGCTCACCTGCTGTGTCCCAAGCAGTCTGCTAGTCTCTGCAGATAAGCAAGGAAG
CATTAAAGCCCTGAAGAAGTGTAGAAGTGGAAAAGATGAGCAGACACTGAGCTTAAGATAACAAGATGCA
TGGAAAAGAAGTAGTCTGGAAGGATGTAGAGGGGAAGGTACTAAGCTGGAGGGAGGAGACTCAGGGGAAT
CTTCTTCCACCAACAGGATGCCTGATCAGCCTTTTGAAGGACAAACCATTTGAAGGTGTCAAGGAATAAG
ACTTAGCCCTAGATAGTGGCGACACCACCGGCAAGGTAAAATGGTATGAGGAGTATGTGTGCTCAGCCTG
CTGAGAAGTCAGTACCTGGAGGAAGGTCCAGGTCATCAGAGGAAGGAATCAGTAAGCTGGAAGGCAAGAT
GCTGGGGCAGCCTCACCGGCTGTGTTATGCCCTGGGATACCTTTGGCAATGTTGGAAAGATATTTCATTA
GGCTTTCAGTTCATTAAGAAATTCCTCCCCCTCTGGACTTAAGACCCTGCATGGTTTTCTTATATTTTAT
TTGGTTAATCTTGGTGTGCTCTTTGTAGACCCTCTTTGGTCAGGATCTTTCTCCATCCCCCAAACAGCTG
TAGCATGACAAAGCCTGGCATTGTGCCAGCTGGCCAAAGAGAAATGTTCTGGGGTCTAGCTCCAGAACCA
GTAAGCAGGATGAGGAGTGGATGTAAAGCTGAGAGGAAATAAGGTGATAATTGCACATGCCATGATCATC
CAGTTGCTCAGGGTTCCTCGCTAGGGTGTCTTCTCCCCTCCATACAGCTACACCCCACCCAACCCTCAGG
AGCTCCCCAAATGCCACCTGTTATGACACCTTCCCACAACAGATCTACAACACAGGATTTCTCACTTCAG
TGGTATTCCAGAACTCTTCTGGGTTGTAAGCTGTCTTACATCACTTTAAACCAGCACTTTTTACCCTTAG
TAACAAAAAAAACCCCATAATCTCTCTTTTCTCCTATTTTTTGACAGAGCTATGCAGCATGGGGAATTTA
AATTTCCTGACCAGGGACTGAACTCATTCCCCCCACAGTAGAAGCTCAGTGTCTTAACCACTGTACCACC
AGGGAAGTCCCAAATTCATAATCTTAAAAAAACAAAATCACCTCATGTTCCCAAGTTCCTGGAGGCTCCC
ACAGGTTATATGTTGCCCATGTCACTTCAGACTGTTGTTTTCCTACATACATCACCAAATGCAGGTCTTA
CTTTGCACGATCATAATACTACTGTACATATTGTTGCTTTAACTGTTCATGAATGTTGGTTCTGGCTCCC
CACTAAGTTGTTAGCTCCCTCAAGGTGAAGCTGGTATCTTCTACTCTCTTCCTCTTTACAGAAAGATCTA
CTGGAATTCCAGCCTCAACAGCAGATCCATCCTTGTCCACAAAACCAGCCCTATTCTGACACAGGGCGAA
CTGAATGAATTGAGGTGCTTCGTCTCCACACTCGGTTGAGCTCTGACCATATGTCCCAGATGAGTGATGA
CATTTCTGCTTTCTTAAGGCCATACCCCACAGTCTTGCCCAAACTCTCCTAATTGGATCTCTGGCATTGT
TACCGATAACAACAGTAATCATGACAGCTTTCCTTGACGAAACACTTACTATATATCAGGGGCCATGCTA
AACACTGGTCCTTAGAGCATCCCTGGGAGGCAGATGTTATTCTGTAATCTTCACAGCTACCTCCTGAGAT
ACAGTATCATTAAGCTATATTGCTAGGAGCAAATAGGCACAGAAATGTTAAGTAACTTGATCTTTATAAG
ATTGTTAAGTAACCAATCTCTTAGCATCAGCAAACTGACCCTGGTCACAGAAGAAATCAATCAGAGAAGA
AGGACATTAAAAGACATTCCCTTCCTGACAGAGTAAGCCTGTCTGACACCCCAGCCCCTCCTGGCATCAA
GATTCCACAGCTTCTTGGGACTTCAATGGTGGTCCAGTGGTTAAGACTCCATGTTTCCAAGGCAGGGGAT
GAAAGTTCAATCCCTTGTAGGGGAACTCAGATCCCACATGCTCCATGGTGTGGCCAAAAAGTTTAAACAG
TTTTTTAAAAAAAGATTCCACTGCTTCCTTACCTCATCATTTTAAAGAACATTTCTTTCCTCCCCCAAGG
CTGTTTATCAGAATTTTCTGTCCCTTCTGGCATGGGACCCAGTCTGACTTATGCAGTTCTTCCCTGGAAT
TTTCCAAATCAGAGGTAGATGTATTGTTTTTTCATTTCCGATGCCAAATTGCAAGCCTGACCTCAGAGTT
GCTGTGGCCACATGTGTGGCCATATAACAGGTCTGAGAGATGAAACAACCAAGGCAGCAAGAAGCAGAAG
AGAGAGGCAGG
