# Supplementary material for: Major Histocompatibility Complex Genomic Regions as Candidates for Reproductive Success in Cattle: From GWAS to Post-GWAS Analysis
Source: Genes (Basel). 2026 Jul 30;17(8):895. doi: 10.3390/genes17080895 (PMC13511974; doi:10.3390/genes17080895)
Supplement: Supplementary file 1 [file genes-17-00895-s001.zip › Supplementary File S2.pdf]

Supplementary File S2: List of transcription factors identified using TFM-Explorer program.

HNF1A
Lhx3
Foxa2
NKX3-1
Foxq1
Sox2
Pou5f1
FOXF2
SOX9
Nkx3-2
NFKB1
NFE2L1
MafG
NR4A2
Foxd3
MEF2A
ARID3A
NF-kappaB
Stat3
MEF2A
FOXD1
HOXA5
GABPA
FOXA1
NFYA
TAL1
TCF3
