# Supplementary material for: Major Histocompatibility Complex Genomic Regions as Candidates for Reproductive Success in Cattle: From GWAS to Post-GWAS Analysis
Source: Genes (Basel). 2026 Jul 30;17(8):895. doi: 10.3390/genes17080895 (PMC13511974; doi:10.3390/genes17080895)
Supplement: Supplementary file 1 [file genes-17-00895-s001.zip › Supplementary Table S1.pdf]

Supplementary Table S1: MHC candidate genes identified in the literature with chromosome and positional data.

Author	Year	Journal	Type of study	Breed	Country	N° of animals	Trait	Gene	Identity	Chromosome	Starting Position	Final Position	Minus 250 bp	Plus 250 bp
Gao et al. [12]	2023	BMC Genomics	GWAS	Holstein	EUA	3.649.734	Early first calving	*BOLA-NC1*	ENSBTAG00000002069	23	28.523 .535	28.724.6 01	28.273.535	28.974.601
Melo et al. [11]	2017	Plos One	GWAS	Nelore	Brazil	2.925	Heifer rebreeding, number of calvings by 53 months	*BOLA-DQB*	ENSBTAG00000021077	23	25.607 .502	25.620.8 37	25.357.502	25.870.837
Melo et al. [11]	2017	Plos One	GWAS	Nelore	Brazil	2.925	Heifer rebreeding, number of calvings by 53 months	*BOLA-DQA1*	ENSBTAG00000037605	23	25.691 .708	25.695.2 96	25.441.708	25.945.296
Melo et al. [11]	2017	Plos One	GWAS	Nelore	Brazil	2.925	Heifer rebreeding, number of calvings by 53 months	*BOLA-DQA2*	ENSBTAG00000009656	23	25.583 .083	25.589.2 09	25.333.083	25.839.209
Melo et al. [11]	2017	Plos One	GWAS	Nelore	Brazil	2.925	Heifer rebreeding, number of calvings by 53 months	*BOLA-DRA*	ENSBTAG00000010645	23	25.838 .389	25.849.4 12	25.588.389	26.099.412
Melo et al. [11]	2017	Plos One	GWAS	Nelore	Brazil	2.925	Heifer rebreeding, number of calvings by 53 months	*HLA-DQA1*	-	23	25.426 .330	25.430.0 97	25.176.330	25.680.097
Peixoto et al. [13]	2023	American Dairy Science Association	Transcriptomic	Holstein	EUA	48	Elongation of bovine conceptuses	*BOLA-NC1*	ENSBTAG00000002069	23	28.523 .535	28.724.6 01	28.273.535	28.974.601
Peixoto et al. [13]	2023	American Dairy Science Association	Transcriptomic	Holstein	EUA	48	Elongation of bovine conceptuses	*BOLA-DQA1*	ENSBTAG00000037605	23	25.691 .708	25.695.2 96	25.441.708	25.945.296
Santos et al. [14]	2015	Veterinary Immunology and Immunopathology	Transcriptomic	Crossbred	Brazil	18	Fetal development	*BOLA-MICB*	-	-	-	-	-	-
Santos et al. [14]	2015	Veterinary Immunology and Immunopathology	Transcriptomic	Crossbred	Brazil	18	Fetal development	*BOLA-NC1*	ENSBTAG00000002069	23	28.523 .535	28.724.6 01	28.273.535	28.974.601
Santos et al. [14]	2015	Veterinary Immunology and Immunopathology	Transcriptomic	Crossbred	Brazil	18	Fetal development	*BOLA-NC3*	-	-	-	-	-	-

- refers to data not found in version ARS-UCD1.3 of the Ensembl database.
