# Supplementary material for: Major Histocompatibility Complex Genomic Regions as Candidates for Reproductive Success in Cattle: From GWAS to Post-GWAS Analysis
Source: Genes (Basel). 2026 Jul 30;17(8):895. doi: 10.3390/genes17080895 (PMC13511974; doi:10.3390/genes17080895)
Supplement: Supplementary file 1 [file genes-17-00895-s001.zip › Supplementary Table S2.pdf]

Supplementary Table S2: GWAS candidate genes and transcriptome candidate genes with stable IDs, gene descriptions, and genomic positions.

Table S2: 54 candidate genes from GWAS studies. Gene, stable identifier (gene stable ID), gene description, genomic position (chromosome, initial and final positions in base pairs (bp)), official gene name.

Gene stable ID	Gene description	Chromosome	Gene start (bp)	Gene end (bp)	Gene name	Type of gene
ENSBTAG00000011358	immediate early response 3 [Source:VGNC Symbol;Acc:VGNC:30039]	23	28274878	28286183	*IER3*	Protein Coding
ENSBTAG00000009960	flotillin 1 [Source:VGNC Symbol;Acc:VGNC:29036]	23	28286486	28296570	*FLOT1*	Protein Coding
ENSBTAG00000025526	mediator of DNA damage checkpoint 1 [Source:VGNC Symbol;Acc:VGNC:31328]	23	28304234	28317823	*MDC1*	Protein Coding
ENSBTAG00000006966	nurim [Source:VGNC Symbol;Acc:VGNC:49560]	23	28319981	28323011	*NRM*	Protein Coding
ENSBTAG00000039695	protein phosphatase 1 regulatory subunit 18 [Source:VGNC Symbol;Acc:VGNC:33233]	23	28323816	28333569	*PPP1R18*	Protein Coding
ENSBTAG00000006960	DEAH-box helicase 16 [Source:VGNC Symbol;Acc:VGNC:28049]	23	28336627	28348884	*DHX16*	Protein Coding
ENSBTAG00000006933	protein phosphatase 1 regulatory subunit 10 [Source:VGNC Symbol;Acc:VGNC:33221]	23	28373727	28389653	*PPP1R10*	Protein Coding
ENSBTAG00000025516	G protein nucleolar 1 (putative) [Source:VGNC Symbol;Acc:VGNC:57319]	23	28420981	28428768	*GNL1*	Protein Coding
ENSBTAG00000063412	-	23	28468108	28476740	-	
ENSBTAG00000038619	-	23	28493346	28505609	-	Protein Coding
ENSBTAG00000035744	tripartite motif containing 26 [Source:HGNC Symbol;Acc:HGNC:12962]	23	28777621	28787323	*TRIM26*	Protein Coding
ENSBTAG00000007530	tripartite motif containing 10 [Source:VGNC Symbol;Acc:VGNC:36312]	23	28816455	28825684	*TRIM10*	Protein Coding
ENSBTAG00000004490	tripartite motif containing 31 [Source:VGNC Symbol;Acc:VGNC:36327]	23	28854091	28880148	*TRIM31*	Protein Coding
ENSBTAG00000052499	ring finger protein 39 [Source:VGNC Symbol;Acc:VGNC:106899]	23	28904539	28909089	*RNF39*	Protein Coding
ENSBTAG00000058345	-	23	28912658	28917619	-	
ENSBTAG00000054588	-	23	28920606	28926650	-	Protein Coding
ENSBTAG00000017836	ZFP57 zinc finger protein [Source:VGNC Symbol;Acc:VGNC:37165]	23	28938683	28943320	*ZFP57*	Protein Coding
ENSBTAG00000021077	major histocompatibility complex, class II, DQ beta [Source:NCBI gene (formerly Entrezgene);Acc:282495]	23	25607502	25620837	*BOLA-DQB*	Protein Coding
ENSBTAG00000019588	-	23	25672686	25680159	-	Protein Coding
ENSBTAG00000013919	-	23	25723424	25735337	-	Protein Coding
ENSBTAG00000038397	-	23	25795993	25810143	-	Protein Coding
ENSBTAG00000015565	-	23	25827573	25828791	-	Protein Coding
ENSBTAG00000034939	butyrophilin like 2 [Source:VGNC Symbol;Acc:VGNC:107284]	23	25858687	25883362	*BTNL2*	Protein Coding
ENSBTAG00000007618	-	23	25899578	25907363	-	Protein Coding
ENSBTAG00000063006	-	23	25967336	25974876	-	Protein Coding
ENSBTAG00000069649	-	23	26012777	26024206	-	LncRNA

ENSBTAG00000057211	-	23	26085335	26114858	-	
ENSBTAG00000015083	F-box protein 9 [Source:VGNC Symbol;Acc:VGNC:28921]	23	25275775	25297258	*FBXO9*	Protein Coding
ENSBTAG00000006969	tubulin beta class I [Source:VGNC Symbol;Acc:VGNC:103058]	23	28298184	28301777	*TUBB*	Protein Coding
ENSBTAG00000006958	chromosome 23 C6orf136 homolog [Source:VGNC Symbol;Acc:VGNC:53961]	23	28349054	28353973	*C23H6orf136*	Protein Coding
ENSBTAG00000006941	alpha tubulin acetyltransferase 1 [Source:VGNC Symbol;Acc:VGNC:26236]	23	28355119	28366119	*ATAT1*	Protein Coding
ENSBTAG00000006936	mitochondrial ribosomal protein S18B [Source:VGNC Symbol;Acc:VGNC:50008]	23	28366052	28373341	*MRPS18B*	Protein Coding
ENSBTAG00000006927	ATP binding cassette subfamily F member 1 [Source:VGNC Symbol;Acc:VGNC:25480]	23	28394981	28408133	*ABCF1*	Protein Coding
ENSBTAG00000006914	proline rich 3 [Source:VGNC Symbol;Acc:VGNC:33396]	23	28415008	28421221	*PRR3*	Protein Coding
ENSBTAG00000050907	ribonuclease P/MRP subunit p21 [Source:VGNC Symbol;Acc:VGNC:106902]	23	28450680	28453965	*RPP21*	Protein Coding
ENSBTAG00000007643	tripartite motif containing 39 [Source:HGNC Symbol;Acc:HGNC:10065]	23	28455599	28467526	*TRIM39*	Protein Coding
ENSBTAG00000069337	-	23	28490859	28493960	-	
ENSBTAG00000002069	-	23	28523535	28724601	-	Protein Coding
ENSBTAG00000051089	-	23	28747018	28769136	-	Protein Coding
ENSBTAG00000007532	tripartite motif containing 15 [Source:VGNC Symbol;Acc:VGNC:36316]	23	28802214	28813725	*TRIM15*	Protein Coding
ENSBTAG00000037381	tripartite motif containing 40 [Source:VGNC Symbol;Acc:VGNC:54243]	23	28828093	28841508	*TRIM40*	Protein Coding
ENSBTAG00000032247	protein phosphatase 1 regulatory inhibitor subunit 11 [Source:VGNC Symbol;Acc:VGNC:33222]	23	28909903	28913349	*PPP1R11*	Protein Coding
ENSBTAG00000031792	RNA polymerase I subunit H [Source:VGNC Symbol;Acc:VGNC:37362]	23	28917109	28920646	*POLR1H*	Protein Coding
ENSBTAG00000017818	myelin oligodendrocyte glycoprotein [Source:VGNC Symbol;Acc:VGNC:57316]	23	28941968	28953585	*MOG*	Protein Coding
ENSBTAG00000003359	ELOVL fatty acid elongase 5 [Source:VGNC Symbol;Acc:VGNC:28451]	23	25400733	25474020	*ELOVL5*	Protein Coding
ENSBTAG00000009656	-	23	25583083	25589209	-	Protein Coding
ENSBTAG00000038128	major histocompatibility complex, class II, DQ alpha 5 [Source:NCBI gene (formerly Entrezgene);Acc:282494]	23	25636907	25643878	*BOLA-DQA5*	Protein Coding
ENSBTAG00000037605	-	23	25691708	25695296	-	Protein Coding
ENSBTAG00000010645	major histocompatibility complex, class II, DR alpha [Source:HGNC Symbol;Acc:HGNC:4947]	23	25838389	25849412	*HLA-DRA*	Protein Coding
ENSBTAG00000034945	-	23	25885521	25894260	-	Protein Coding
ENSBTAG00000032642	glutathione S-transferase, alpha 3 [Source:NCBI gene (formerly Entrezgene);Acc:768055]	23	25182280	25204322	*GSTA3*	Protein Coding
ENSBTAG00000004288	glutathione S-transferase alpha 4 [Source:VGNC Symbol;Acc:VGNC:53979]	23	25209831	25224178	*GSTA4*	Protein Coding
ENSBTAG00000015590	ciliosis associated kinase 1 [Source:VGNC Symbol;Acc:VGNC:30028]	23	25229377	25272669	*CILK1*	Protein Coding
ENSBTAG00000008148	glial cells missing transcription factor 1 [Source:VGNC Symbol;Acc:VGNC:29291]	23	25311576	25332914	*GCM1*	Protein Coding

- refers to data not found in version ARS-UCD1.3 of the Ensembl database.

46 candidate genes from transcriptomic studies. Gene, stable identifier (gene stable ID), gene description, genomic position (chromosome, initial and final positions in base pairs (bp)), official gene name.

Gene stable ID	Gene description	Chromosome/scaffold name	Gene start (bp)	Gene end (bp)	Gene name	Type of gene
ENSBTAG00000011358	immediate early response 3 [Source:VGNC Symbol;Acc:VGNC:30039]	23	28274878	28286183	*IER3*	Protein Coding
ENSBTAG00000009960	flotillin 1 [Source:VGNC Symbol;Acc:VGNC:29036]	23	28286486	28296570	*FLOT1*	Protein Coding
ENSBTAG00000025526	mediator of DNA damage checkpoint 1 [Source:VGNC Symbol;Acc:VGNC:31328]	23	28304234	28317823	*MDC1*	Protein Coding
ENSBTAG00000006966	nurim [Source:VGNC Symbol;Acc:VGNC:49560]	23	28319981	28323011	*NRM*	Protein Coding
ENSBTAG00000039695	protein phosphatase 1 regulatory subunit 18 [Source:VGNC Symbol;Acc:VGNC:33233]	23	28323816	28333569	*PPP1R18*	Protein Coding
ENSBTAG00000006960	DEAH-box helicase 16 [Source:VGNC Symbol;Acc:VGNC:28049]	23	28336627	28348884	*DHX16*	Protein Coding
ENSBTAG00000006933	protein phosphatase 1 regulatory subunit 10 [Source:VGNC Symbol;Acc:VGNC:33221]	23	28373727	28389653	*PPP1R10*	Protein Coding
ENSBTAG00000025516	G protein nucleolar 1 (putative) [Source:VGNC Symbol;Acc:VGNC:57319]	23	28420981	28428768	*GNL1*	Protein Coding
ENSBTAG00000063412	-	23	28468108	28476740	-	
ENSBTAG00000038619	-	23	28493346	28505609	-	Protein Coding
ENSBTAG00000035744	tripartite motif containing 26 [Source:HGNC Symbol;Acc:HGNC:12962]	23	28777621	28787323	*TRIM26*	Protein Coding
ENSBTAG00000007530	tripartite motif containing 10 [Source:VGNC Symbol;Acc:VGNC:36312]	23	28816455	28825684	*TRIM10*	Protein Coding
ENSBTAG00000004490	tripartite motif containing 31 [Source:VGNC Symbol;Acc:VGNC:36327]	23	28854091	28880148	*TRIM31*	Protein Coding
ENSBTAG00000052499	ring finger protein 39 [Source:VGNC Symbol;Acc:VGNC:106899]	23	28904539	28909089	*RNF39*	Protein Coding
ENSBTAG00000058345	-	23	28912658	28917619	-	Protein Coding
ENSBTAG00000054588	-	23	28920606	28926650	-	Protein Coding
ENSBTAG00000017836	ZFP57 zinc finger protein [Source:VGNC Symbol;Acc:VGNC:37165]	23	28938683	28943320	*ZFP57*	Protein Coding
ENSBTAG00000021077	major histocompatibility complex, class II, DQ beta [Source:NCBI gene (formerly Entrezgene);Acc:282495]	23	25607502	25620837	*BOLA-DQB*	Protein Coding
ENSBTAG00000019588	-	23	25672686	25680159	-	Protein Coding
ENSBTAG00000013919	-	23	25723424	25735337	-	Protein Coding
ENSBTAG00000038397	-	23	25795993	25810143	-	Protein Coding
ENSBTAG00000015565	-	23	25827573	25828791	-	Protein Coding
ENSBTAG00000034939	butyrophilin like 2 [Source:VGNC Symbol;Acc:VGNC:107284]	23	25858687	25883362	*BTNL2*	Protein Coding
ENSBTAG00000007618	-	23	25899578	25907363	-	Protein Coding
ENSBTAG00000006969	-	23	28298184	28301777	*TUBB*	Protein Coding
ENSBTAG00000006958	chromosome 23 C6orf136 homolog [Source:VGNC Symbol;Acc:VGNC:53961]	23	28349054	28353973	*C23H6orf136*	Protein Coding

ENSBTAG00000006941	alpha tubulin acetyltransferase 1 [Source:VGNC Symbol;Acc:VGNC:26236]	23	28355119	28366119	*ATAT1*	Protein Coding
ENSBTAG00000006936	mitochondrial ribosomal protein S18B [Source:VGNC Symbol;Acc:VGNC:50008]	23	28366052	28373341	*MRPS18B*	Protein Coding
ENSBTAG00000006927	ATP binding cassette subfamily F member 1 [Source:VGNC Symbol;Acc:VGNC:25480]	23	28394981	28408133	*ABCF1*	Protein Coding
ENSBTAG00000006914	proline rich 3 [Source:VGNC Symbol;Acc:VGNC:33396]	23	28415008	28421221	*PRR3*	Protein Coding
ENSBTAG00000050907	ribonuclease P/MRP subunit p21 [Source:VGNC Symbol;Acc:VGNC:106902]	23	28450680	28453965	*RPP21*	Protein Coding
ENSBTAG00000007643	tripartite motif containing 39 [Source:HGNC Symbol;Acc:HGNC:10065]	23	28455599	28467526	*TRIM39*	Protein Coding
ENSBTAG00000069337	-	23	28490859	28493960	-	
ENSBTAG00000002069	-	23	28523535	28724601	-	Protein Coding
ENSBTAG00000051089	-	23	28747018	28769136	-	Protein Coding
ENSBTAG00000007532	tripartite motif containing 15 [Source:VGNC Symbol;Acc:VGNC:36316]	23	28802214	28813725	*TRIM15*	Protein Coding
ENSBTAG00000037381	tripartite motif containing 40 [Source:VGNC Symbol;Acc:VGNC:54243]	23	28828093	28841508	*TRIM40*	Protein Coding
ENSBTAG00000032247	protein phosphatase 1 regulatory inhibitor subunit 11 [Source:VGNC Symbol;Acc:VGNC:33222]	23	28909903	28913349	*PPP1R11*	Protein Coding
ENSBTAG00000031792	RNA polymerase I subunit H [Source:VGNC Symbol;Acc:VGNC:37362]	23	28917109	28920646	*POLR1H*	Protein Coding
ENSBTAG00000017818	myelin oligodendrocyte glycoprotein [Source:VGNC Symbol;Acc:VGNC:57316]	23	28941968	28953585	*MOG*	Protein Coding
ENSBTAG00000003359	ELOVL fatty acid elongase 5 [Source:VGNC Symbol;Acc:VGNC:28451]	23	25400733	25474020	*ELOVL5*	Protein Coding
ENSBTAG00000009656	-	23	25583083	25589209	-	Protein Coding
ENSBTAG00000038128	major histocompatibility complex, class II, DQ alpha 5 [Source:NCBI gene (formerly Entrezgene);Acc:282494]	23	25636907	25643878	*BOLA-DQA5*	Protein Coding
ENSBTAG00000037605	-	23	25691708	25695296	-	Protein Coding
ENSBTAG00000010645	major histocompatibility complex, class II, DR alpha [Source:HGNC Symbol;Acc:HGNC:4947]	23	25838389	25849412	*-HLA-DRA*	Protein Coding
ENSBTAG00000034945	-	23	25885521	25894260	-	Protein Coding

- refers to data not found in version ARS-UCD1.3 of the Ensembl database.
