# Supplementary material for: Major Histocompatibility Complex Genomic Regions as Candidates for Reproductive Success in Cattle: From GWAS to Post-GWAS Analysis
Source: Genes (Basel). 2026 Jul 30;17(8):895. doi: 10.3390/genes17080895 (PMC13511974; doi:10.3390/genes17080895)
Supplement: Supplementary file 1 [file genes-17-00895-s001.zip › Supplementary Table S3.pdf]

Supplementary Table S3: Gene ontology terms and enriched genes for GWAS and transcriptome candidates.

Annotation Cluster 1	Main Term, Enrichment Score: 8.37	Genes enriched	p-value	Benjamini
**GOTERM_BP_DIRECT**	peptide antigen assembly with MHC class II protein complex	9	7.2×10^{-16}	7.5×10^{-14}
**GOTERM_BP_DIRECT**	antigen processing and presentation of exogenous peptide antigen via MHC class II	9	2.2×10^{-15}	1.1×10^{-13}
**GOTERM_MF_DIRECT**	MHC class II protein complex binding	9	3.2×10^{-15}	1.3×10^{-13}
**GOTERM_CC_DIRECT**	MHC class II protein complex	9	4.2×10^{-15}	2.4×10^{-13}
**UP_KW_CELLULAR_COMPONENT**	MHC II	8	4.8×10^{-15}	8.2×10^{-14}
**INTERPRO**	MHC/Immunoglobulin	9	7.8×10^{-15}	4.0×10^{-13}
**GOTERM_BP_DIRECT**	positive regulation of T cell activation	9	8.3×10^{-15}	2.9×10^{-13}
**INTERPRO**	MHC_II_a/b_N	8	2.7×10^{-14}	6.9×10^{-13}
**INTERPRO**	Ig_C1-set	11	6.0×10^{-14}	1.2×10^{-12}
**GOTERM_MF_DIRECT**	peptide antigen binding	9	6.6×10^{-14}	1.4×10^{-12}
**INTERPRO**	Ig/MHC_CS	10	1.9×10^{-13}	2.5×10^{-12}
**KEGG_PATHWAY**	Allograft rejection	8	2.6×10^{-13}	8.2×10^{-12}
**KEGG_PATHWAY**	Type I diabetes mellitus	8	3.5×10^{-13}	8.2×10^{-12}
**KEGG_PATHWAY**	Graft-versus-host disease	8	6.7×10^{-13}	1.1×10^{-11}
**INTERPRO**	MHC_I/II-like_Ag-recog	10	9.4×10^{-13}	9.6×10^{-12}
**KEGG_PATHWAY**	Autoimmune thyroid disease	8	2.1×10^{-12}	2.5×10^{-11}
**SMART**	IGc1	11	4.8×10^{-12}	5.3×10^{-11}
**KEGG_PATHWAY**	Viral myocarditis	8	8.1×10^{-12}	7.5×10^{-11}
**KEGG_PATHWAY**	Antigen processing and presentation	8	9.6×10^{-12}	7.5×10^{-11}
**GOTERM_BP_DIRECT**	positive regulation of immune response	7	1.2×10^{-11}	3.1×10^{-10}
**INTERPRO**	Ig-like_dom	15	1.3×10^{-11}	1.1×10^{-10}
**UP_SEQ_FEATURE**	DOMAIN:Ig-like	15	1.3×10^{-11}	3.3×10^{-10}
**KEGG_PATHWAY**	Phagosome	9	1.6×10^{-11}	1.1×10^{-10}
**INTERPRO**	Ig-like_dom_sf	15	6.5×10^{-11}	5.1×10^{-10}
**GOTERM_BP_DIRECT**	antigen processing and presentation of peptide or polysaccharide antigen via MHC class II	6	2.7×10^{-10}	5.6×10^{-9}
**GOTERM_BP_DIRECT**	immunoglobulin production involved in immunoglobulin-mediated immune response	6	4.3×10^{-10}	7.3×10^{-9}
**GOTERM_CC_DIRECT**	late endosome membrane	8	6.9×10^{-10}	2.0×10^{-8}
**KEGG_PATHWAY**	Asthma	6	1.1×10^{-9}	6.6×10^{-9}
**KEGG_PATHWAY**	Cell adhesion molecules	8	1.3×10^{-9}	6.6×10^{-9}

**INTERPRO**	Ig-like_fold	15	2.2×10^{-9}	1.5×10^{-8}
**UP_KW_BIOLOGICAL_PROCESS**	Adaptive immunity	8	7.4×10^{-9}	8.9×10^{-8}
**KEGG_PATHWAY**	Intestinal immune network for IgA production	6	9.9×10^{-9}	4.7×10^{-8}
**GOTERM_BP_DIRECT**	adaptive immune response	8	1.2×10^{-8}	1.8×10^{-7}
**KEGG_PATHWAY**	Epstein-Barr virus infection	8	1.3×10^{-8}	5.4×10^{-8}
**KEGG_PATHWAY**	Human T-cell leukemia virus 1 infection	8	1.4×10^{-8}	5.4×10^{-8}
**KEGG_PATHWAY**	Herpes simplex virus 1 infection	9	2.9×10^{-8}	9.7×10^{-8}
**KEGG_PATHWAY**	Inflammatory bowel disease	6	2.9×10^{-8}	9.7×10^{-8}
**KEGG_PATHWAY**	Leishmaniasis	6	5.7×10^{-8}	1.8×10^{-7}
**GOTERM_BP_DIRECT**	antigen processing and presentation	5	6.0×10^{-8}	7.7×10^{-7}
**GOTERM_CC_DIRECT**	lysosomal membrane	8	1.1×10^{-7}	2.1×10^{-6}
**KEGG_PATHWAY**	Th1 and Th2 cell differentiation	6	1.6×10^{-7}	4.8×10^{-7}
**KEGG_PATHWAY**	Rheumatoid arthritis	6	2.3×10^{-7}	6.3×10^{-7}
**UP_KW_BIOLOGICAL_PROCESS**	Immunity	9	2.3×10^{-7}	1.4×10^{-6}
**KEGG_PATHWAY**	Staphylococcus aureus infection	6	2.4×10^{-7}	6.3×10^{-7}
**KEGG_PATHWAY**	Hematopoietic cell lineage	6	3.0×10^{-7}	7.5×10^{-7}
**KEGG_PATHWAY**	Th17 cell differentiation	6	3.6×10^{-7}	8.6×10^{-7}
**KEGG_PATHWAY**	Toxoplasmosis	6	4.1×10^{-7}	9.3×10^{-7}
**INTERPRO**	MHC_II_a_N	4	1.4×10^{-6}	7.7×10^{-6}
**INTERPRO**	MHC_II_b_N	4	1.8×10^{-6}	9.3×10^{-6}
**GOTERM_BP_DIRECT**	immune response	8	1.9×10^{-6}	2.2×10^{-5}
**KEGG_PATHWAY**	Systemic lupus erythematosus	6	4.6×10^{-6}	9.8×10^{-6}
**KEGG_PATHWAY**	Influenza A	6	5.5×10^{-6}	1.1×10^{-5}
**SMART**	MHC_II_alpha	4	6.8×10^{-6}	2.5×10^{-5}
**KEGG_PATHWAY**	Tuberculosis	6	7.5×10^{-6}	1.5×10^{-5}
**SMART**	MHC_II_beta	4	8.6×10^{-6}	2.7×10^{-5}
**UP_SEQ_FEATURE**	DOMAIN:MHC class II beta chain N-terminal	3	4.7×10^{-5}	4.8×10^{-4}
**GOTERM_BP_DIRECT**	positive regulation of T cell mediated cytotoxicity	3	5.6×10^{-3}	4.1×10^{-2}
**UP_KW_DOMAIN**	Transmembrane helix	14	1.1×10^{-2}	5.4×10^{-2}
**UP_KW_DOMAIN**	Signal	14	1.3×10^{-2}	5.4×10^{-2}
**UP_KW_PTM**	Glycoprotein	16	3.3×10^{-2}	2.3×10^{-1}
**UP_KW_CELLULAR_COMPONENT**	Membrane	18	1.3×10^{-1}	1.0

**UP_KW_DOMAIN**	Transmembrane	15	2.8×10^{-1}	7.3×10^{-1}
**GOTERM_CC_DIRECT**	membrane	11	4.7×10^{-1}	1.0
**UP_SEQ_FEATURE**	TRANSMEM:Helical	14	6.9×10^{-1}	1.0
**Annotation Cluster 2**	**Main Term, Enrichment Score: 6.85**	**Genes enriched**	**p-value**	**Benjamini**
**INTERPRO**	TRIM/RBCC	10	3.0×10^{-15}	3.1×10^{-13}
**INTERPRO**	Butyrophylin_SPRY	10	1.4×10^{-14}	4.8×10^{-13}
**INTERPRO**	SPRY_dom	10	1.4×10^{-13}	2.3×10^{-12}
**INTERPRO**	B30.2/SPRY	10	1.6×10^{-13}	2.4×10^{-12}
**UP_SEQ_FEATURE**	DOMAIN:B30.2/SPRY	10	1.7×10^{-13}	8.7×10^{-12}
**INTERPRO**	B30.2/SPRY_sf	10	2.7×10^{-13}	3.1×10^{-12}
**INTERPRO**	PRY	8	1.4×10^{-12}	1.3×10^{-11}
**SMART**	SPRY	10	4.7×10^{-12}	5.3×10^{-11}
**SMART**	PRY	8	4.5×10^{-11}	3.3×10^{-10}
**INTERPRO**	ConA-like_dom_sf	10	2.7×10^{-10}	1.9×10^{-9}
**UP_SEQ_FEATURE**	DOMAIN:B box-type	7	3.9×10^{-9}	6.6×10^{-8}
**INTERPRO**	Znf_B-box	7	4.5×10^{-9}	2.8×10^{-8}
**INTERPRO**	Znf_RING_CS	7	5.5×10^{-7}	3.3×10^{-6}
**UP_SEQ_FEATURE**	DOMAIN:RING-type	8	8.5×10^{-7}	1.1×10^{-5}
**INTERPRO**	Znf_RING	8	9.9×10^{-7}	5.6×10^{-6}
**SMART**	BBOX	6	1.2×10^{-6}	6.4×10^{-6}
**SMART**	RING	8	6.0×10^{-6}	2.5×10^{-5}
**GOTERM_BP_DIRECT**	protein ubiquitination	7	1.6×10^{-5}	1.6×10^{-4}
**INTERPRO**	Znf_RING/FYVE/PHD	8	1.6×10^{-5}	7.6×10^{-5}
**GOTERM_MF_DIRECT**	ubiquitin protein ligase activity	7	2.7×10^{-5}	3.6×10^{-4}
**GOTERM_BP_DIRECT**	negative regulation of viral entry into host cell	3	4.8×10^{-4}	3.8×10^{-3}
**GOTERM_MF_DIRECT**	zinc ion binding	8	4.9×10^{-4}	4.0×10^{-3}
**INTERPRO**	Znf_C3HC4_RING-type	3	3.0×10^{-3}	1.3×10^{-2}
**UP_KW_LIGAND**	Zinc	13	3.2×10^{-3}	1.9×10^{-2}
**GOTERM_BP_DIRECT**	innate immune response	4	1.9×10^{-2}	1.3×10^{-1}
**UP_KW_LIGAND**	Metal-binding	12	1.5×10^{-1}	4.5×10^{-1}
**GOTERM_CC_DIRECT**	cytoplasm	11	1.9×10^{-1}	1.0
**GOTERM_MF_DIRECT**	protein binding	7	2.5×10^{-1}	1.0

**UP_KW_DOMAIN**	Coiled coil	5	6.1×10^{-1}	9.8×10^{-1}
**Annotation Cluster 3**	**Main Term, Enrichment Score: 2.57**	**Genes enriched**	**p-value**	**Benjamini**
**INTERPRO**	IgSF_BTN/MOG	4	5.6×10^{-6}	2.7×10^{-5}
**GOTERM_MF_DIRECT**	signaling receptor binding	6	3.5×10^{-5}	3.6×10^{-4}
**GOTERM_BP_DIRECT**	T cell receptor signaling pathway	4	1.7×10^{-4}	1.6×10^{-3}
**GOTERM_BP_DIRECT**	regulation of cytokine production	4	3.9×10^{-4}	3.3×10^{-3}
**GOTERM_CC_DIRECT**	external side of plasma membrane	6	4.8×10^{-4}	6.9×10^{-3}
**INTERPRO**	BTN3A2-like_Ig-C	3	5.1×10^{-4}	2.2×10^{-3}
**INTERPRO**	Ig_V-set	4	3.1×10^{-2}	1.2×10^{-1}
**SMART**	IGv	4	3.7×10^{-2}	1.0×10^{-1}
**INTERPRO**	Ig_sub	4	6.8×10^{-2}	2.4×10^{-1}
**SMART**	IG	4	2.2×10^{-1}	4.7×10^{-1}
**UP_KW_PTM**	Disulfide bond	7	9.6×10^{-1}	9.8×10^{-1}

Terms highlighted in blue refers to the most significant terms in the Top 1 Cluster ($p \leq 0.05$).

Gene ontology terms and enriched genes from the 46 candidate transcriptomic genes.

**Annotation Cluster 1**	**Main Term, Enrichment Score: 8.42**	**Genes enriched**	**p-value**	**Benjamini**
**GOTERM_BP_DIRECT**	peptide antigen assembly with MHC class II protein complex	9	7.2×10^{-16}	7.5×10^{-14}
**GOTERM_BP_DIRECT**	antigen processing and presentation of exogenous peptide antigen via MHC class II	9	2.2×10^{-15}	1.1×10^{-13}
**GOTERM_MF_DIRECT**	MHC class II protein complex binding	9	2.5×10^{-15}	1.0×10^{-13}
**GOTERM_CC_DIRECT**	MHC class II protein complex	9	4.2×10^{-15}	2.4×10^{-13}
**UP_KW_CELLULAR_COMPONENT**	MHC II	8	4.8×10^{-15}	8.2×10^{-14}
**INTERPRO**	MHC/Immunoglobulin	9	5.2×10^{-15}	5.2×10^{-13}
**GOTERM_BP_DIRECT**	positive regulation of T cell activation	9	8.3×10^{-15}	2.9×10^{-13}
**INTERPRO**	MHC_II_a/b_N	8	1.9×10^{-14}	9.7×10^{-13}
**INTERPRO**	Ig_C1-set	11	3.6×10^{-14}	1.2×10^{-12}

**GOTERM_MF_DIRECT**	peptide antigen binding	9	5.3×10^{-14}	1.1×10^{-12}
**INTERPRO**	Ig/MHC_CS	10	1.2×10^{-13}	3.1×10^{-12}
**KEGG_PATHWAY**	Allograft rejection	8	2.6×10^{-13}	8.2×10^{-12}
**KEGG_PATHWAY**	Type I diabetes mellitus	8	3.5×10^{-13}	8.2×10^{-12}
**INTERPRO**	MHC_I/II-like_Ag-recog	10	6.0×10^{-13}	1.2×10^{-11}
**KEGG_PATHWAY**	Graft-versus-host disease	8	6.7×10^{-13}	1.1×10^{-11}
**KEGG_PATHWAY**	Autoimmune thyroid disease	8	2.1×10^{-12}	2.5×10^{-11}
**SMART**	IGc1	11	2.4×10^{-12}	5.2×10^{-11}
**UP_SEQ_FEATURE**	DOMAIN:Ig-like	15	6.1×10^{-12}	3.1×10^{-10}
**INTERPRO**	Ig-like_dom	15	6.2×10^{-12}	1.0×10^{-10}
**KEGG_PATHWAY**	Viral myocarditis	8	8.1×10^{-12}	7.5×10^{-11}
**KEGG_PATHWAY**	Antigen processing and presentation	8	9.6×10^{-12}	7.5×10^{-11}
**GOTERM_BP_DIRECT**	positive regulation of immune response	7	1.2×10^{-11}	3.1×10^{-10}
**KEGG_PATHWAY**	Phagosome	9	1.6×10^{-11}	1.1×10^{-10}
**INTERPRO**	Ig-like_dom_sf	15	3.2×10^{-11}	4.0×10^{-10}
**GOTERM_BP_DIRECT**	antigen processing and presentation of peptide or polysaccharide antigen via MHC class II	6	2.7×10^{-10}	5.6×10^{-9}
**GOTERM_BP_DIRECT**	immunoglobulin production involved in immunoglobulin-mediated immune response	6	4.3×10^{-10}	7.3×10^{-9}
**GOTERM_CC_DIRECT**	late endosome membrane	8	6.9×10^{-10}	2.0×10^{-8}
**INTERPRO**	Ig-like_fold	15	1.1×10^{-9}	8.4×10^{-9}
**KEGG_PATHWAY**	Asthma	6	1.1×10^{-9}	6.6×10^{-9}
**KEGG_PATHWAY**	Cell adhesion molecules	8	1.3×10^{-9}	6.6×10^{-9}
**UP_KW_BIOLOGICAL_PROCESS**	Adaptive immunity	8	7.4×10^{-9}	8.9×10^{-8}
**KEGG_PATHWAY**	Intestinal immune network for IgA production	6	9.9×10^{-9}	4.7×10^{-8}
**GOTERM_BP_DIRECT**	adaptive immune response	8	1.2×10^{-8}	1.8×10^{-7}
**KEGG_PATHWAY**	Epstein-Barr virus infection	8	1.3×10^{-8}	5.4×10^{-8}
**KEGG_PATHWAY**	Human T-cell leukemia virus 1 infection	8	1.4×10^{-8}	5.4×10^{-8}
**KEGG_PATHWAY**	Herpes simplex virus 1 infection	9	2.9×10^{-8}	9.7×10^{-8}
**KEGG_PATHWAY**	Inflammatory bowel disease	6	2.9×10^{-8}	9.7×10^{-8}
**KEGG_PATHWAY**	Leishmaniasis	6	5.7×10^{-8}	1.8×10^{-7}
**GOTERM_BP_DIRECT**	antigen processing and presentation	5	6.0×10^{-8}	7.7×10^{-7}
**GOTERM_CC_DIRECT**	lysosomal membrane	8	1.1×10^{-7}	2.1×10^{-6}
**KEGG_PATHWAY**	Th1 and Th2 cell differentiation	6	1.6×10^{-7}	4.8×10^{-7}
**KEGG_PATHWAY**	Rheumatoid arthritis	6	2.3×10^{-7}	6.3×10^{-7}

**UP_KW_BIOLOGICAL_PROCESS**	Immunity	9	2.3×10^{-7}	1.4×10^{-6}
**KEGG_PATHWAY**	Staphylococcus aureus infection	6	2.4×10^{-7}	6.3×10^{-7}
**KEGG_PATHWAY**	Hematopoietic cell lineage	6	3.0×10^{-7}	7.5×10^{-7}
**KEGG_PATHWAY**	Th17 cell differentiation	6	3.6×10^{-7}	8.6×10^{-7}
**KEGG_PATHWAY**	Toxoplasmosis	6	4.1×10^{-7}	9.3×10^{-7}
**INTERPRO**	MHC_II_a_N	4	1.2×10^{-6}	6.6×10^{-6}
**INTERPRO**	MHC_II_b_N	4	1.6×10^{-6}	8.0×10^{-6}
**GOTERM_BP_DIRECT**	immune response	8	1.9×10^{-6}	2.2×10^{-5}
**KEGG_PATHWAY**	Systemic lupus erythematosus	6	4.6×10^{-6}	9.8×10^{-6}
**KEGG_PATHWAY**	Influenza A	6	5.5×10^{-6}	1.1×10^{-5}
**SMART**	MHC_II_alpha	4	5.6×10^{-6}	2.1×10^{-5}
**SMART**	MHC_II_beta	4	7.1×10^{-6}	2.2×10^{-5}
**KEGG_PATHWAY**	Tuberculosis	6	7.5×10^{-6}	1.5×10^{-5}
**UP_SEQ_FEATURE**	DOMAIN:MHC class II beta chain N-terminal	3	4.2×10^{-5}	4.3×10^{-4}
**GOTERM_BP_DIRECT**	positive regulation of T cell mediated cytotoxicity	3	5.6×10^{-3}	4.1×10^{-2}
**UP_KW_DOMAIN**	Transmembrane helix	14	6.9×10^{-3}	3.5×10^{-2}
**UP_KW_DOMAIN**	Signal	14	8.7×10^{-3}	3.5×10^{-2}
**UP_KW_PTM**	Glycoprotein	14	7.4×10^{-2}	5.2×10^{-1}
**UP_KW_CELLULAR_COMPONENT**	Membrane	18	1.3×10^{-1}	1.0
**UP_KW_DOMAIN**	Transmembrane	15	2.1×10^{-1}	5.6×10^{-1}
**GOTERM_CC_DIRECT**	membrane	11	4.7×10^{-1}	1.0
**UP_SEQ_FEATURE**	TRANSMEM:Helical	14	6.2×10^{-1}	1.0
**Annotation Cluster 2**	**Enrichment Score: 5.7**	**Genes enriched**	**p-value**	**Benjamini**
**INTERPRO**	TRIM/RBCC	8	2.0×10^{-11}	2.9×10^{-10}
**INTERPRO**	Butyrophylin_SPRY	8	6.4×10^{-11}	7.2×10^{-10}
**INTERPRO**	SPRY_dom	8	3.6×10^{-10}	3.7×10^{-9}
**INTERPRO**	B30.2/SPRY	8	4.2×10^{-10}	3.8×10^{-9}
**UP_SEQ_FEATURE**	DOMAIN:B30.2/SPRY	8	4.3×10^{-10}	1.1×10^{-8}
**INTERPRO**	B30.2/SPRY_sf	8	6.2×10^{-10}	5.2×10^{-9}
**UP_SEQ_FEATURE**	DOMAIN:B box-type	7	2.9×10^{-9}	4.9×10^{-8}
**INTERPRO**	Znf_B-box	7	3.3×10^{-9}	2.4×10^{-8}
**SMART**	SPRY	8	5.3×10^{-9}	5.8×10^{-8}
**INTERPRO**	PRY	6	1.2×10^{-8}	8.1×10^{-8}

**INTERPRO**	ConA-like_dom_sf	8	1.2×10^{-7}	7.8×10^{-7}
**SMART**	PRY	6	1.4×10^{-7}	1.0×10^{-6}
**INTERPRO**	Znf_RING_CS	7	4.1×10^{-7}	2.4×10^{-6}
**UP_SEQ_FEATURE**	DOMAIN:RING-type	8	6.0×10^{-7}	7.7×10^{-6}
**INTERPRO**	Znf_RING	8	7.1×10^{-7}	4.0×10^{-6}
**SMART**	BBOX	6	8.4×10^{-7}	4.6×10^{-6}
**SMART**	RING	8	3.8×10^{-6}	1.7×10^{-5}
**INTERPRO**	Znf_RING/FYVE/PHD	8	1.2×10^{-5}	5.5×10^{-5}
**GOTERM_BP_DIRECT**	protein ubiquitination	7	1.6×10^{-5}	1.6×10^{-4}
**GOTERM_MF_DIRECT**	ubiquitin protein ligase activity	7	2.3×10^{-5}	3.2×10^{-4}
**GOTERM_MF_DIRECT**	zinc ion binding	8	4.1×10^{-4}	3.4×10^{-3}
**GOTERM_BP_DIRECT**	negative regulation of viral entry into host cell	3	4.8×10^{-4}	3.8×10^{-3}
**INTERPRO**	Znf_C3HC4_RING-type	3	2.7×10^{-3}	1.1×10^{-2}
**UP_KW_LIGAND**	Zinc	13	3.2×10^{-3}	1.9×10^{-2}
**GOTERM_BP_DIRECT**	innate immune response	4	1.9×10^{-2}	1.3×10^{-1}
**UP_KW_LIGAND**	Metal-binding	12	1.5×10^{-1}	4.5×10^{-1}
**GOTERM_CC_DIRECT**	cytoplasm	11	1.9×10^{-1}	1.0
**GOTERM_MF_DIRECT**	protein binding	6	4.0×10^{-1}	1.0
**UP_KW_DOMAIN**	Coiled coil	4	7.9×10^{-1}	1.0
**Annotation Cluster 3**	**Enrichment Score: 2.61**	**Genes enriched**	**p-value**	**Benjamini**
**INTERPRO**	IgSF_BTN/MOG	4	4.9×10^{-6}	2.4×10^{-5}
**GOTERM_MF_DIRECT**	signaling receptor binding	6	3.1×10^{-5}	3.2×10^{-4}
**GOTERM_BP_DIRECT**	T cell receptor signaling pathway	4	1.7×10^{-4}	1.6×10^{-3}
**GOTERM_BP_DIRECT**	regulation of cytokine production	4	3.9×10^{-4}	3.3×10^{-3}
**INTERPRO**	BTN3A2-like_Ig-C	3	4.6×10^{-4}	2.0×10^{-3}
**GOTERM_CC_DIRECT**	external side of plasma membrane	6	4.8×10^{-4}	6.9×10^{-3}
**INTERPRO**	Ig_V-set	4	2.7×10^{-2}	1.1×10^{-1}
**SMART**	IGv	4	3.1×10^{-2}	8.6×10^{-2}
**INTERPRO**	Ig_sub	4	6.1×10^{-2}	2.2×10^{-1}
**SMART**	IG	4	1.9×10^{-1}	4.2×10^{-1}
**UP_KW_PTM**	Disulfide bond	5	9.9×10^{-1}	9.9×10^{-1}

Terms highlighted in blue refers to the most significant terms in the Top 1 Cluster ($p \leq 0.05$).
