# Supplementary material for: Major Histocompatibility Complex Genomic Regions as Candidates for Reproductive Success in Cattle: From GWAS to Post-GWAS Analysis
Source: Genes (Basel). 2026 Jul 30;17(8):895. doi: 10.3390/genes17080895 (PMC13511974; doi:10.3390/genes17080895)
Supplement: Supplementary file 1 [file genes-17-00895-s001.zip › Supplementary Table S4.pdf]

Supplementary Table S4: Identification of gene ontology terms, *p*-values, cluster frequencies, and transcription factors for GWAS and transcriptome candidates.

Table S4: Gene ontology identification (GO ID), gene ontology description, p-values, corrected p-values, frequency of clusters and total frequency of transcription factors (TF) identified from the 54 candidate GWAS genes.

GO ID	GO Description	p-value	Corrected p-value	Cluster frequency	Total frequency	Transcription Factors
6355	Regulation of transcription, DNA-dependent	1,02E-11	3,08E-09	14/14 100.0%	716/8356 8.5%	*FOXA1, MEF2A, NFYA, STAT3, HNF1A, POU5F1, NFKB1, NR4A2, LHX3, MAFG, HOXA5, GABPA, NFE2L1, FOXA2*
51252	Regulation of RNA metabolic process	1,48E-11	3,08E-09	14/14 100.0%	735/8356 8.7%	*FOXA1, MEF2A, NFYA, STAT3, HNF1A, POU5F1, NFKB1, NR4A2, LHX3, MAFG, HOXA5, GABPA, NFE2L1, FOXA2*
45449	Regulation of transcription	8,10E-10	1,12E-07	14/14 100.0%	976/8356 11.6%	*FOXA1, MEF2A, NFYA, STAT3, HNF1A, POU5F1, NFKB1, NR4A2, LHX3, MAFG, HOXA5, GABPA, NFE2L1, FOXA2*
10556	Regulation of macromolecule biosynthetic process	2,46E-09	2,56E-07	14/14 100.0%	1056/8356 12.6%	*FOXA1, MEF2A, NFYA, STAT3, HNF1A, POU5F1, NFKB1, NR4A2, LHX3, MAFG, HOXA5, GABPA, NFE2L1, FOXA2*
31326	Regulation of cellular biosynthetic process	4,15E-09	2,73E-07	14/14 100.0%	1096/8356 13.1%	*FOXA1, MEF2A, NFYA, STAT3, HNF1A, POU5F1, NFKB1, NR4A2, LHX3, MAFG, HOXA5, GABPA, NFE2L1, FOXA2*
10468	Regulation of gene expression	4,54E-09	2,73E-07	14/14 100.0%	1103/8356 13.2%	*FOXA1, MEF2A, NFYA, STAT3, HNF1A, POU5F1, NFKB1, NR4A2, LHX3, MAFG, HOXA5, GABPA, NFE2L1, FOXA2*
9889	Regulation of biosynthetic process	4,60E-09	2,73E-07	14/14 100.0%	1104/8356 13.2%	*FOXA1, MEF2A, NFYA, STAT3, HNF1A, POU5F1, NFKB1, NR4A2, LHX3, MAFG, HOXA5, GABPA, NFE2L1, FOXA2*
19219	Regulation of nucleobase, nucleoside, nucleotide and nucleic acid metabolic process	5,56E-09	2,80E-07	14/14 100.0%	1119/8356 13.3%	*FOXA1, MEF2A, NFYA, STAT3, HNF1A, POU5F1, NFKB1, NR4A2, LHX3, MAFG, HOXA5, GABPA, NFE2L1, FOXA2*
51171	Regulation of nitrogen compound metabolic process	6,07E-09	2,80E-07	14/14 100.0%	1126/8356 13.4%	*FOXA1, MEF2A, NFYA, STAT3, HNF1A, POU5F1, NFKB1, NR4A2, LHX3, MAFG, HOXA5, GABPA, NFE2L1, FOXA2*
60255	Regulation of macromolecule metabolic process	2,83E-08	1,18E-06	14/14 100.0%	1256/8356 15.0%	*FOXA1, MEF2A, NFYA, STAT3, HNF1A, POU5F1, NFKB1, NR4A2, LHX3, MAFG, HOXA5, GABPA, NFE2L1, FOXA2*
80090	Regulation of primary metabolic process	4,79E-08	1,81E-06	14/14 100.0%	1304/8356 15.6%	*FOXA1, MEF2A, NFYA, STAT3, HNF1A, POU5F1, NFKB1, NR4A2, LHX3, MAFG, HOXA5, GABPA, NFE2L1, FOXA2*
31323	Regulation of cellular metabolic process	7,88E-08	2,73E-06	14/14 100.0%	1351/8356 16.1%	*FOXA1, MEF2A, NFYA, STAT3, HNF1A, POU5F1, NFKB1, NR4A2, LHX3, MAFG, HOXA5, GABPA, NFE2L1, FOXA2*
19222	Regulation of metabolic process	1,95E-07	6,25E-06	14/14 100.0%	1441/8356 17.2%	*FOXA1, MEF2A, NFYA, STAT3, HNF1A, POU5F1, NFKB1, NR4A2, LHX3, MAFG, HOXA5, GABPA, NFE2L1, FOXA2*
6357	Regulation of transcription from RNA polymerase II promoter	7,60E-06	2,26E-04	8/14 57.1%	230/8356 2.7%	*FOXA1, NR4A2, NFYA, STAT3, HNF1A, HOXA5, GABPA, NFKB1*
50794	Regulation of cellular process	2,47E-04	6,86E-03	14/14 100.0%	2396/8356 28.6%	*FOXA1, MEF2A, NFYA, STAT3, HNF1A, POU5F1, NFKB1, NR4A2, LHX3, MAFG, HOXA5, GABPA, NFE2L1, FOXA2*
45944	Positive regulation of transcription from RNA polymerase II promoter	4,49E-04	1,17E-02	6/14 42.8%	136/8356 1.6%	*FOXA1, NR4A2, NFYA, HNF1A, HOXA5, NFKB1*
50789	Regulation of biological process	6,98E-04	1,71E-02	14/14 100.0%	2580/8356 30.8%	*FOXA1, MEF2A, NFYA, STAT3, HNF1A, POU5F1, NFKB1, NR4A2, LHX3, MAFG, HOXA5, GABPA, NFE2L1, FOXA2*
45893	Positive regulation of transcription, DNA-dependent	1,48E-03	3,42E-02	6/14 42.8%	166/8356 1.9%	*FOXA1, NR4A2, NFYA, HNF1A, HOXA5, NFKB1*
51254	Positive regulation of RNA metabolic process	1,59E-03	3,48E-02	6/14 42.8%	168/8356 2.0%	*FOXA1, NR4A2, NFYA, HNF1A, HOXA5, NFKB1*
65007	Biological regulation	2,30E-03	4,79E-02	14/14 100.0%	2809/8356 33.6%	*FOXA1, MEF2A, NFYA, STAT3, HNF1A, POU5F1, NFKB1, NR4A2, LHX3, MAFG, HOXA5, GABPA, NFE2L1, FOXA2*
45941	Positive regulation of transcription	3,20E-03	6,33E-02	6/14 42.8%	189/8356 2.2%	*FOXA1, NR4A2, NFYA, HNF1A, HOXA5, NFKB1*
10628	Positive regulation of gene expression	4,47E-03	8,44E-02	6/14 42.8%	200/8356 2.3%	*FOXA1, NR4A2, NFYA, HNF1A, HOXA5, NFKB1*
10557	Positive regulation of macromolecule biosynthetic process	8,26E-03	1,49E-01	6/14 42.8%	222/8356 2.6%	*FOXA1, NR4A2, NFYA, HNF1A, HOXA5, NFKB1*
45935	Positive regulation of nucleobase, nucleoside, nucleotide and nucleic acid metabolic process	9,18E-03	1,59E-01	6/14 42.8%	226/8356 2.7%	*FOXA1, NR4A2, NFYA, HNF1A, HOXA5, NFKB1*

51173	Positive regulation of nitrogen compound metabolic process	1,10E-02	1,83E-01	6/14 42.8%	233/8356 2.7%	*FOXA1, NR4A2, NFYA, HNF1A, HOXA5, NFKB1*
31328	Positive regulation of cellular biosynthetic process	1,31E-02	2,09E-01	6/14 42.8%	240/8356 2.8%	*FOXA1, NR4A2, NFYA, HNF1A, HOXA5, NFKB1*
9891	Positive regulation of biosynthetic process	1,44E-02	2,22E-01	6/14 42.8%	244/8356 2.9%	*FOXA1, NR4A2, NFYA, HNF1A, HOXA5, NFKB1*
10604	Positive regulation of macromolecule metabolic process	5,28E-02	7,84E-01	6/14 42.8%	305/8356 3.6%	*FOXA1, NR4A2, NFYA, HNF1A, HOXA5, NFKB1*
31325	Positive regulation of cellular metabolic process	6,36E-02	8,73E-01	6/14 42.8%	315/8356 3.7%	*FOXA1, NR4A2, NFYA, HNF1A, HOXA5, NFKB1*
42593	Glucose homeostasis	6,50E-02	8,73E-01	3/14 21.4%	23/8356 0.2%	*FOXA1, STAT3, HNF1A*
33500	Carbohydrate homeostasis	6,50E-02	8,73E-01	3/14 21.4%	23/8356 0.2%	*FOXA1, STAT3, HNF1A*
9893	Positive regulation of metabolic process	9,23E-02	1,20E+00	6/14 42.8%	336/8356 4.0%	*FOXA1, NR4A2, NFYA, HNF1A, HOXA5, NFKB1*
48469	Cell maturation	1,33E-01	1,68E+00	3/14 21.4%	29/8356 0.3%	*FOXA1, NR4A2, HOXA5*
21700	Developmental maturation	3,05E-01	3,73E+00	3/14 21.4%	38/8356 0.4%	*FOXA1, NR4A2, HOXA5*
7275	Multicellular organismal development	3,48E-01	4,14E+00	8/14 57.1%	922/8356 11.0%	*FOXA1, MEF2A, NR4A2, MAFG, STAT3, HNF1A, HOXA5, GABPA*
2070	Epithelial cell maturation	3,90E-01	4,50E+00	2/14 14.2%	6/8356 0.0%	*FOXA1, HOXA5*
32502	Developmental process	6,61E-01	7,43E+00	8/14 57.1%	1006/8356 12.0%	*FOXA1, MEF2A, NR4A2, MAFG, STAT3, HNF1A, HOXA5, GABPA*
2065	Columnar/cuboidal epithelial cell differentiation	7,26E-01	7,95E+00	2/14 14.2%	8/8356 0.0%	*FOXA1, HOXA5*
43009	Chordate embryonic development	1,06E+00	1,13E+01	4/14 28.5%	158/8356 1.8%	*MAFG, HNF1A, HOXA5, GABPA*
9792	Embryonic development ending in birth or egg hatching	1,09E+00	1,13E+01	4/14 28.5%	159/8356 1.9%	*MAFG, HNF1A, HOXA5, GABPA*
2064	Epithelial cell development	2,70E+00	2,74E+01	2/14 14.2%	15/8356 0.1%	*FOXA1, HOXA5*
32501	Multicellular organismal process	3,04E+00	2,95E+01	8/14 57.1%	1243/8356 14.8%	*FOXA1, MEF2A, NR4A2, MAFG, STAT3, HNF1A, HOXA5, GABPA*
51239	Regulation of multicellular organismal process	3,05E+00	2,95E+01	5/14 35.7%	390/8356 4.6%	*FOXA1, NR4A2, MAFG, HOXA5, NFKB1*
9887	Organ morphogenesis	3,30E+00	3,12E+01	4/14 28.5%	212/8356 2.5%	*FOXA1, STAT3, HNF1A, HOXA5*
9653	Anatomical structure morphogenesis	3,51E+00	3,24E+01	5/14 35.7%	402/8356 4.8%	*FOXA1, NR4A2, STAT3, HNF1A, HOXA5*
48522	Positive regulation of cellular process	4,24E+00	3,84E+01	6/14 42.8%	664/8356 7.9%	*FOXA1, NR4A2, NFYA, HNF1A, HOXA5, NFKB1*
9790	Embryonic development	4,58E+00	4,05E+01	4/14 28.5%	231/8356 2.7%	*MAFG, HNF1A, HOXA5, GABPA*
48731	System development	6,34E+00	5,49E+01	6/14 42.8%	715/8356 8.5%	*FOXA1, MEF2A, NR4A2, STAT3, HNF1A, HOXA5*
7399	Nervous system development	6,66E+00	5,58E+01	4/14 28.5%	255/8356 3.0%	*FOXA1, MEF2A, NR4A2, STAT3*
30182	Neuron differentiation	6,71E+00	5,58E+01	3/14 21.4%	107/8356 1.2%	*FOXA1, NR4A2, STAT3*
1701	In utero embryonic development	6,89E+00	5,62E+01	3/14 21.4%	108/8356 1.2%	*MAFG, HNF1A, GABPA*
48729	Tissue morphogenesis	7,86E+00	6,17E+01	3/14 21.4%	113/8356 1.3%	*FOXA1, HNF1A, HOXA5*

45892	Negative regulation of transcription, DNA-dependent	7,86E+00	6,17E+01	3/14 21.4%	113/8356 1.3%	*FOXA1, GABPA, NFKB1*
51253	Negative regulation of RNA metabolic process	8,49E+00	6,52E+01	3/14 21.4%	116/8356 1.3%	*FOXA1, GABPA, NFKB1*
48518	Positive regulation of biological process	8,62E+00	6,52E+01	6/14 42.8%	757/8356 9.0%	*FOXA1, NR4A2, NFYA, HNF1A, HOXA5, NFKB1*
35270	Endocrine system development	8,93E+00	6,64E+01	2/14 14.2%	27/8356 0.3%	*HNF1A, HOXA5*
48856	Anatomical structure development	1,13E+01	8,28E+01	6/14 42.8%	797/8356 9.5%	*FOXA1, MEF2A, NR4A2, STAT3, HNF1A, HOXA5*
16481	Negative regulation of transcription	1,37E+01	9,28E+01	3/14 21.4%	137/8356 1.6%	*FOXA1, GABPA, NFKB1*
30154	Cell differentiation	1,49E+01	9,28E+01	5/14 35.7%	551/8356 6.5%	*FOXA1, MEF2A, NR4A2, STAT3, HOXA5*
48699	Generation of neurons	1,56E+01	9,28E+01	3/14 21.4%	143/8356 1.7%	*FOXA1, NR4A2, STAT3*
45083	Negative regulation of interleukin-12 biosynthetic process	1,68E+01	9,28E+01	1/14 7.1%	1/8356 0.0%	*NFKB1*
60480	Lung goblet cell differentiation	1,68E+01	9,28E+01	1/14 7.1%	1/8356 0.0%	*HOXA5*
60481	Lobar bronchus epithelium development	1,68E+01	9,28E+01	1/14 7.1%	1/8356 0.0%	*HOXA5*
60482	Lobar bronchus development	1,68E+01	9,28E+01	1/14 7.1%	1/8356 0.0%	*HOXA5*
60484	Lung-associated mesenchyme development	1,68E+01	9,28E+01	1/14 7.1%	1/8356 0.0%	*HOXA5*
60535	Trachea cartilage morphogenesis	1,68E+01	9,28E+01	1/14 7.1%	1/8356 0.0%	*HOXA5*
60536	Cartilage morphogenesis	1,68E+01	9,28E+01	1/14 7.1%	1/8356 0.0%	*HOXA5*
60574	Intestinal epithelial cell maturation	1,68E+01	9,28E+01	1/14 7.1%	1/8356 0.0%	*HOXA5*
60576	Intestinal epithelial cell development	1,68E+01	9,28E+01	1/14 7.1%	1/8356 0.0%	*HOXA5*
48341	Paraxial mesoderm formation	1,68E+01	9,28E+01	1/14 7.1%	1/8356 0.0%	*HNF1A*
60738	Epithelial-mesenchymal signaling involved in prostate gland development	1,68E+01	9,28E+01	1/14 7.1%	1/8356 0.0%	*FOXA1*
60764	Cell-cell signaling involved in mammary gland development	1,68E+01	9,28E+01	1/14 7.1%	1/8356 0.0%	*HOXA5*
15721	Bile acid and bile salt transport	1,68E+01	9,28E+01	1/14 7.1%	1/8356 0.0%	*HNF1A*
6699	Bile acid biosynthetic process	1,68E+01	9,28E+01	1/14 7.1%	1/8356 0.0%	*HNF1A*
48754	Branching morphogenesis of a tube	1,68E+01	9,28E+01	2/14 14.2%	37/8356 0.4%	*FOXA1, HOXA5*
48869	Cellular developmental process	1,70E+01	9,28E+01	5/14 35.7%	568/8356 6.7%	*FOXA1, MEF2A, NR4A2, STAT3, HOXA5*
7610	Behavior	1,72E+01	9,28E+01	3/14 21.4%	148/8356 1.7%	*NR4A2, MAFG, STAT3*
45934	Negative regulation of nucleobase, nucleoside, nucleotide and nucleic acid metabolic process	1,92E+01	1,02E+02	3/14 21.4%	154/8356 1.8%	*FOXA1, GABPA, NFKB1*

48871	Multicellular organismal homeostasis	1,96E+01	1,02E+02	2/14 14.2%	40/8356 0.4%	*STAT3, HNF1A*
51172	Negative regulation of nitrogen compound metabolic process	1,96E+01	1,02E+02	3/14 21.4%	155/8356 1.8%	*FOXA1, GABPA, NFKB1*
22008	Neurogenesis	2,11E+01	1,08E+02	3/14 21.4%	159/8356 1.9%	*FOXA1, NR4A2, STAT3*
30534	Adult behavior	2,16E+01	1,10E+02	2/14 14.2%	42/8356 0.5%	*NR4A2, MAFG*
10629	Negative regulation of gene expression	2,19E+01	1,10E+02	3/14 21.4%	161/8356 1.9%	*FOXA1, GABPA, NFKB1*
10558	Negative regulation of macromolecule biosynthetic process	2,43E+01	1,20E+02	3/14 21.4%	167/8356 1.9%	*FOXA1, GABPA, NFKB1*
31327	Negative regulation of cellular biosynthetic process	2,68E+01	1,31E+02	3/14 21.4%	173/8356 2.0%	*FOXA1, GABPA, NFKB1*
9890	Negative regulation of biosynthetic process	2,82E+01	1,36E+02	3/14 21.4%	176/8356 2.1%	*FOXA1, GABPA, NFKB1*
10551	Regulation of gene-specific transcription from RNA polymerase II promoter	2,93E+01	1,37E+02	2/14 14.2%	49/8356 0.5%	*HNF1A, NFKB1*
30323	Respiratory tube development	2,93E+01	1,37E+02	2/14 14.2%	49/8356 0.5%	*FOXA1, HOXA5*
30324	Lung development	2,93E+01	1,37E+02	2/14 14.2%	49/8356 0.5%	*FOXA1, HOXA5*
48608	Reproductive structure development	3,05E+01	1,39E+02	2/14 14.2%	50/8356 0.5%	*FOXA1, HNF1A*
1763	Morphogenesis of a branching structure	3,05E+01	1,39E+02	2/14 14.2%	50/8356 0.5%	*FOXA1, HOXA5*
60541	Respiratory system development	3,30E+01	1,41E+02	2/14 14.2%	52/8356 0.6%	*FOXA1, HOXA5*
60433	Bronchus development	3,35E+01	1,41E+02	1/14 7.1%	2/8356 0.0%	*HOXA5*
2066	Columnar/cuboidal epithelial cell development	3,35E+01	1,41E+02	1/14 7.1%	2/8356 0.0%	*HOXA5*
60435	Bronchiole development	3,35E+01	1,41E+02	1/14 7.1%	2/8356 0.0%	*HOXA5*
2069	Columnar/cuboidal epithelial cell maturation	3,35E+01	1,41E+02	1/14 7.1%	2/8356 0.0%	*HOXA5*
60684	Epithelial-mesenchymal cell signaling	3,35E+01	1,41E+02	1/14 7.1%	2/8356 0.0%	*FOXA1*
60741	Prostate gland stromal morphogenesis	3,35E+01	1,41E+02	1/14 7.1%	2/8356 0.0%	*FOXA1*
60743	Epithelial cell maturation involved in prostate gland development	3,35E+01	1,41E+02	1/14 7.1%	2/8356 0.0%	*FOXA1*
10033	Response to organic substance	3,93E+01	1,64E+02	3/14 21.4%	198/8356 2.3%	*NR4A2, STAT3, HNF1A*
30855	Epithelial cell differentiation	4,22E+01	1,74E+02	2/14 14.2%	59/8356 0.7%	*FOXA1, HOXA5*
7154	Cell communication	4,34E+01	1,77E+02	3/14 21.4%	205/8356 2.4%	*FOXA1, NR4A2, HOXA5*
48468	Cell development	4,52E+01	1,80E+02	3/14 21.4%	208/8356 2.4%	*FOXA1, NR4A2, HOXA5*
9719	Response to endogenous stimulus	4,80E+01	1,80E+02	2/14 14.2%	63/8356 0.7%	*NR4A2, STAT3*

8206	Bile acid metabolic process	5,02E+01	1,80E+02	1/14 7.1%	3/8356 0.0%	*HNF1A*
60439	Trachea morphogenesis	5,02E+01	1,80E+02	1/14 7.1%	3/8356 0.0%	*HOXA5*
60534	Trachea cartilage development	5,02E+01	1,80E+02	1/14 7.1%	3/8356 0.0%	*HOXA5*
60575	Intestinal epithelial cell differentiation	5,02E+01	1,80E+02	1/14 7.1%	3/8356 0.0%	*HOXA5*
48339	Paraxial mesoderm development	5,02E+01	1,80E+02	1/14 7.1%	3/8356 0.0%	*HNF1A*
48340	Paraxial mesoderm morphogenesis	5,02E+01	1,80E+02	1/14 7.1%	3/8356 0.0%	*HNF1A*
60638	Mesenchymal-epithelial cell signaling	5,02E+01	1,80E+02	1/14 7.1%	3/8356 0.0%	*HOXA5*
31018	Endocrine pancreas development	5,02E+01	1,80E+02	1/14 7.1%	3/8356 0.0%	*HNF1A*
21952	Central nervous system projection neuron axonogenesis	5,02E+01	1,80E+02	1/14 7.1%	3/8356 0.0%	*NR4A2*
48665	Neuron fate specification	5,02E+01	1,80E+02	1/14 7.1%	3/8356 0.0%	*FOXA1*
45604	Regulation of epidermal cell differentiation	5,02E+01	1,80E+02	1/14 7.1%	3/8356 0.0%	*MAFG*
60397	JAK-STAT cascade involved in growth hormone signaling pathway	5,02E+01	1,80E+02	1/14 7.1%	3/8356 0.0%	*STAT3*
31324	Negative regulation of cellular metabolic process	5,09E+01	1,80E+02	3/14 21.4%	217/8356 2.5%	*FOXA1, GABPA, NFKB1*
32583	Regulation of gene-specific transcription	5,11E+01	1,80E+02	2/14 14.2%	65/8356 0.7%	*HNF1A, NFKB1*
48878	Chemical homeostasis	5,56E+01	1,94E+02	3/14 21.4%	224/8356 2.6%	*FOXA1, STAT3, HNF1A*
3	Reproduction	5,70E+01	1,98E+02	3/14 21.4%	226/8356 2.7%	*FOXA1, STAT3, HNF1A*
10605	Negative regulation of macromolecule metabolic process	5,77E+01	1,98E+02	3/14 21.4%	227/8356 2.7%	*FOXA1, GABPA, NFKB1*
48732	Gland development	6,40E+01	2,11E+02	2/14 14.2%	73/8356 0.8%	*FOXA1, HOXA5*
45075	Regulation of interleukin-12 biosynthetic process	6,69E+01	2,11E+02	1/14 7.1%	4/8356 0.0%	*NFKB1*
60479	Lung cell differentiation	6,69E+01	2,11E+02	1/14 7.1%	4/8356 0.0%	*HOXA5*
42053	Regulation of dopamine metabolic process	6,69E+01	2,11E+02	1/14 7.1%	4/8356 0.0%	*NR4A2*
60487	Lung epithelial cell differentiation	6,69E+01	2,11E+02	1/14 7.1%	4/8356 0.0%	*HOXA5*
42069	Regulation of catecholamine metabolic process	6,69E+01	2,11E+02	1/14 7.1%	4/8356 0.0%	*NR4A2*
60528	Secretory columnal luminar epithelial cell differentiation involved in prostate glandular acinus development	6,69E+01	2,11E+02	1/14 7.1%	4/8356 0.0%	*FOXA1*
21955	Central nervous system neuron axonogenesis	6,69E+01	2,11E+02	1/14 7.1%	4/8356 0.0%	*NR4A2*
33238	Regulation of cellular amine metabolic process	6,69E+01	2,11E+02	1/14 7.1%	4/8356 0.0%	*NR4A2*

33599	Regulation of mammary gland epithelial cell proliferation	6,69E+01	2,11E+02	1/14 7.1%	4/8356 0.0%	*HOXA5*
3016	Respiratory system process	6,69E+01	2,11E+02	1/14 7.1%	4/8356 0.0%	*HOXA5*
9892	Negative regulation of metabolic process	7,38E+01	2,31E+02	3/14 21.4%	248/8356 2.9%	*FOXA1, GABPA, NFKB1*
122	Negative regulation of transcription from RNA polymerase II promoter	7,46E+01	2,32E+02	2/14 14.2%	79/8356 0.9%	*FOXA1, GABPA*
2009	Morphogenesis of an epithelium	8,21E+01	2,41E+02	2/14 14.2%	83/8356 0.9%	*FOXA1, HOXA5*
60416	Response to growth hormone stimulus	8,35E+01	2,41E+02	1/14 7.1%	5/8356 0.0%	*STAT3*
60428	Lung epithelium development	8,35E+01	2,41E+02	1/14 7.1%	5/8356 0.0%	*HOXA5*
2067	Glandular epithelial cell differentiation	8,35E+01	2,41E+02	1/14 7.1%	5/8356 0.0%	*FOXA1*
60438	Trachea development	8,35E+01	2,41E+02	1/14 7.1%	5/8356 0.0%	*HOXA5*
42416	Dopamine biosynthetic process	8,35E+01	2,41E+02	1/14 7.1%	5/8356 0.0%	*NR4A2*
42551	Neuron maturation	8,35E+01	2,41E+02	1/14 7.1%	5/8356 0.0%	*NR4A2*
45666	Positive regulation of neuron differentiation	8,35E+01	2,41E+02	1/14 7.1%	5/8356 0.0%	*FOXA1*
71378	Cellular response to growth hormone stimulus	8,35E+01	2,41E+02	1/14 7.1%	5/8356 0.0%	*STAT3*
60396	Growth hormone receptor signaling pathway	8,35E+01	2,41E+02	1/14 7.1%	5/8356 0.0%	*STAT3*
9888	Tissue development	9,42E+01	2,70E+02	3/14 21.4%	271/8356 3.2%	*FOXA1, HNF1A, HOXA5*
50793	Regulation of developmental process	9,61E+01	2,74E+02	3/14 21.4%	273/8356 3.2%	*FOXA1, MAFG, HOXA5*
60644	Mammary gland epithelial cell differentiation	1,00E+02	2,80E+02	1/14 7.1%	6/8356 0.0%	*HOXA5*
42755	Eating behavior	1,00E+02	2,80E+02	1/14 7.1%	6/8356 0.0%	*STAT3*
1975	Response to amphetamine	1,00E+02	2,80E+02	1/14 7.1%	6/8356 0.0%	*NR4A2*
3006	Reproductive developmental process	1,02E+02	2,84E+02	2/14 14.2%	93/8356 1.1%	*FOXA1, HNF1A*
35239	Tube morphogenesis	1,04E+02	2,87E+02	2/14 14.2%	94/8356 1.1%	*FOXA1, HOXA5*
10243	Response to organic nitrogen	1,17E+02	3,09E+02	1/14 7.1%	7/8356 0.0%	*NR4A2*
60441	Epithelial tube branching involved in lung morphogenesis	1,17E+02	3,09E+02	1/14 7.1%	7/8356 0.0%	*HOXA5*
60525	Prostate glandular acinus development	1,17E+02	3,09E+02	1/14 7.1%	7/8356 0.0%	*FOXA1*
30878	Thyroid gland development	1,17E+02	3,09E+02	1/14 7.1%	7/8356 0.0%	*HOXA5*
43576	Regulation of respiratory gaseous exchange	1,17E+02	3,09E+02	1/14 7.1%	7/8356 0.0%	*NR4A2*

14075	Response to amine stimulus	1,17E+02	3,09E+02	1/14 7.1%	7/8356 0.0%	*NR4A2*
48523	Negative regulation of cellular process	1,27E+02	3,36E+02	4/14 28.5%	575/8356 6.8%	*FOXA1, NR4A2, GABPA, NFKB1*
7267	Cell-cell signaling	1,31E+02	3,42E+02	2/14 14.2%	106/8356 1.2%	*FOXA1, HOXA5*
21954	Central nervous system neuron development	1,33E+02	3,42E+02	1/14 7.1%	8/8356 0.0%	*NR4A2*
45682	Regulation of epidermis development	1,33E+02	3,42E+02	1/14 7.1%	8/8356 0.0%	*MAFG*
6783	Heme biosynthetic process	1,33E+02	3,42E+02	1/14 7.1%	8/8356 0.0%	*HNF1A*
16477	Cell migration	1,39E+02	3,54E+02	2/14 14.2%	109/8356 1.3%	*NR4A2, HOXA5*
48513	Organ development	1,43E+02	3,64E+02	4/14 28.5%	595/8356 7.1%	*FOXA1, STAT3, HNF1A, HOXA5*
42036	Negative regulation of cytokine biosynthetic process	1,50E+02	3,65E+02	1/14 7.1%	9/8356 0.1%	*NFKB1*
60749	Mammary gland alveolus development	1,50E+02	3,65E+02	1/14 7.1%	9/8356 0.1%	*HOXA5*
15718	Monocarboxylic acid transport	1,50E+02	3,65E+02	1/14 7.1%	9/8356 0.1%	*HNF1A*
45453	Bone resorption	1,50E+02	3,65E+02	1/14 7.1%	9/8356 0.1%	*HNF1A*
35264	Multicellular organism growth	1,50E+02	3,65E+02	1/14 7.1%	9/8356 0.1%	*HOXA5*
15908	Fatty acid transport	1,50E+02	3,65E+02	1/14 7.1%	9/8356 0.1%	*HNF1A*
43691	Reverse cholesterol transport	1,50E+02	3,65E+02	1/14 7.1%	9/8356 0.1%	*HNF1A*
7259	JAK-STAT cascade	1,66E+02	3,87E+02	1/14 7.1%	10/8356 0.1%	*STAT3*
33014	Tetrapyrrole biosynthetic process	1,66E+02	3,87E+02	1/14 7.1%	10/8356 0.1%	*HNF1A*
31016	Pancreas development	1,66E+02	3,87E+02	1/14 7.1%	10/8356 0.1%	*HNF1A*
60742	Epithelial cell differentiation involved in prostate gland development	1,66E+02	3,87E+02	1/14 7.1%	10/8356 0.1%	*FOXA1*
42417	Dopamine metabolic process	1,66E+02	3,87E+02	1/14 7.1%	10/8356 0.1%	*NR4A2*
42423	Catecholamine biosynthetic process	1,66E+02	3,87E+02	1/14 7.1%	10/8356 0.1%	*NR4A2*
6779	Porphyrin biosynthetic process	1,66E+02	3,87E+02	1/14 7.1%	10/8356 0.1%	*HNF1A*
32655	Regulation of interleukin-12 production	1,66E+02	3,87E+02	1/14 7.1%	10/8356 0.1%	*NFKB1*
51674	Localization of cell	1,69E+02	3,88E+02	2/14 14.2%	121/8356 1.4%	*NR4A2, HOXA5*
48870	Cell motility	1,69E+02	3,88E+02	2/14 14.2%	121/8356 1.4%	*NR4A2, HOXA5*
48598	Embryonic morphogenesis	1,72E+02	3,92E+02	2/14 14.2%	122/8356 1.4%	*HNF1A, HOXA5*

60429	Epithelium development	1,74E+02	3,96E+02	2/14 14.2%	123/8356 1.4%	*FOXA1, HOXA5*
6338	Chromatin remodeling	1,83E+02	4,05E+02	1/14 7.1%	11/8356 0.1%	*HNF1A*
48663	Neuron fate commitment	1,83E+02	4,05E+02	1/14 7.1%	11/8356 0.1%	*FOXA1*
1659	Temperature homeostasis	1,83E+02	4,05E+02	1/14 7.1%	11/8356 0.1%	*STAT3*
46849	Bone remodeling	1,83E+02	4,05E+02	1/14 7.1%	11/8356 0.1%	*HNF1A*
60395	SMAD protein signal transduction	1,83E+02	4,05E+02	1/14 7.1%	11/8356 0.1%	*HNF1A*
7167	Enzyme linked receptor protein signaling pathway	1,85E+02	4,07E+02	2/14 14.2%	127/8356 1.5%	*STAT3, HNF1A*
42592	Homeostatic process	1,95E+02	4,21E+02	3/14 21.4%	355/8356 4.2%	*FOXA1, STAT3, HNF1A*
60425	Lung morphogenesis	1,99E+02	4,21E+02	1/14 7.1%	12/8356 0.1%	*HOXA5*
60740	Prostate gland epithelium morphogenesis	1,99E+02	4,21E+02	1/14 7.1%	12/8356 0.1%	*FOXA1*
7585	Respiratory gaseous exchange	1,99E+02	4,21E+02	1/14 7.1%	12/8356 0.1%	*HOXA5*
34284	Response to monosaccharide stimulus	1,99E+02	4,21E+02	1/14 7.1%	12/8356 0.1%	*HNF1A*
9743	Response to carbohydrate stimulus	1,99E+02	4,21E+02	1/14 7.1%	12/8356 0.1%	*HNF1A*
9746	Response to hexose stimulus	1,99E+02	4,21E+02	1/14 7.1%	12/8356 0.1%	*HNF1A*
9749	Response to glucose stimulus	1,99E+02	4,21E+02	1/14 7.1%	12/8356 0.1%	*HNF1A*
48519	Negative regulation of biological process	2,09E+02	4,39E+02	4/14 28.5%	665/8356 7.9%	*FOXA1, NR4A2, GABPA, NFKB1*
35295	Tube development	2,11E+02	4,40E+02	2/14 14.2%	136/8356 1.6%	*FOXA1, HOXA5*
60512	Prostate gland morphogenesis	2,16E+02	4,42E+02	1/14 7.1%	13/8356 0.1%	*FOXA1*
42168	Heme metabolic process	2,16E+02	4,42E+02	1/14 7.1%	13/8356 0.1%	*HNF1A*
1708	Cell fate specification	2,16E+02	4,42E+02	1/14 7.1%	13/8356 0.1%	*FOXA1*
1754	Eye photoreceptor cell differentiation	2,16E+02	4,42E+02	1/14 7.1%	13/8356 0.1%	*STAT3*
48565	Digestive tract development	2,32E+02	4,69E+02	1/14 7.1%	14/8356 0.1%	*HOXA5*
61008	Hepaticobiliary system development	2,32E+02	4,69E+02	1/14 7.1%	14/8356 0.1%	*HNF1A*
1889	Liver development	2,32E+02	4,69E+02	1/14 7.1%	14/8356 0.1%	*HNF1A*
6928	Cellular component movement	2,34E+02	4,71E+02	2/14 14.2%	144/8356 1.7%	*NR4A2, HOXA5*
46530	Photoreceptor cell differentiation	2,49E+02	4,95E+02	1/14 7.1%	15/8356 0.1%	*STAT3*

7631	Feeding behavior	2,49E+02	4,95E+02	1/14 7.1%	15/8356 0.1%	*STAT3*

Lines highlighted in green refer to biological processes related to reproduction.

Gene ontology identification (GO ID), gene ontology description, p-values, corrected p-values, frequency of clusters and total frequency of transcription factors (TF) identified from the 46 candidate transcriptomic genes.

GO ID	GO Description	p-value	Corrected p-value	Cluster frequency	Total frequency	Transcription Factors
6355	Regulation of transcription, DNA-dependent	7,24E-14	2,05E-11	16/16 100.0%	716/8356 8.5%	*MEF2A, NFYA, STAT3, HNF1A, FOXO3, ELK1, NFKB1, POU5F1, NR4A2, LHX3, FEV, MAFG, ELF5, GABPA, NFE2L1, FOXA2*
51252	Regulation of RNA metabolic process	1,11E-13	2,05E-11	16/16 100.0%	735/8356 8.7%	*MEF2A, NFYA, STAT3, HNF1A, FOXO3, ELK1, NFKB1, POU5F1, NR4A2, LHX3, FEV, MAFG, ELF5, GABPA, NFE2L1, FOXA2*
45449	Regulation of transcription	1,08E-11	1,33E-09	16/16 100.0%	976/8356 11.6%	*MEF2A, NFYA, STAT3, HNF1A, FOXO3, ELK1, NFKB1, POU5F1, NR4A2, LHX3, FEV, MAFG, ELF5, GABPA, NFE2L1, FOXA2*
10556	Regulation of macromolecule biosynthetic process	3,83E-11	3,55E-09	16/16 100.0%	1056/8356 12.6%	*MEF2A, NFYA, STAT3, HNF1A, FOXO3, ELK1, NFKB1, POU5F1, NR4A2, LHX3, FEV, MAFG, ELF5, GABPA, NFE2L1, FOXA2*
31326	Regulation of cellular biosynthetic process	6,97E-11	4,16E-09	16/16 100.0%	1096/8356 13.1%	*MEF2A, NFYA, STAT3, HNF1A, FOXO3, ELK1, NFKB1, POU5F1, NR4A2, LHX3, FEV, MAFG, ELF5, GABPA, NFE2L1, FOXA2*
10468	Regulation of gene expression	7,73E-11	4,16E-09	16/16 100.0%	1103/8356 13.2%	*MEF2A, NFYA, STAT3, HNF1A, FOXO3, ELK1, NFKB1, POU5F1, NR4A2, LHX3, FEV, MAFG, ELF5, GABPA, NFE2L1, FOXA2*
9889	Regulation of biosynthetic process	7,84E-11	4,16E-09	16/16 100.0%	1104/8356 13.2%	*MEF2A, NFYA, STAT3, HNF1A, FOXO3, ELK1, NFKB1, POU5F1, NR4A2, LHX3, FEV, MAFG, ELF5, GABPA, NFE2L1, FOXA2*
19219	Regulation of nucleobase, nucleoside, nucleotide and nucleic acid metabolic process	9,74E-11	4,44E-09	16/16 100.0%	1119/8356 13.3%	*MEF2A, NFYA, STAT3, HNF1A, FOXO3, ELK1, NFKB1, POU5F1, NR4A2, LHX3, FEV, MAFG, ELF5, GABPA, NFE2L1, FOXA2*
51171	Regulation of nitrogen compound metabolic process	1,08E-10	4,44E-09	16/16 100.0%	1126/8356 13.4%	*MEF2A, NFYA, STAT3, HNF1A, FOXO3, ELK1, NFKB1, POU5F1, NR4A2, LHX3, FEV, MAFG, ELF5, GABPA, NFE2L1, FOXA2*
60255	Regulation of macromolecule metabolic process	6,26E-10	2,32E-08	16/16 100.0%	1256/8356 15.0%	*MEF2A, NFYA, STAT3, HNF1A, FOXO3, ELK1, NFKB1, POU5F1, NR4A2, LHX3, FEV, MAFG, ELF5, GABPA, NFE2L1, FOXA2*
80090	Regulation of primary metabolic process	1,14E-09	3,86E-08	16/16 100.0%	1304/8356 15.6%	*MEF2A, NFYA, STAT3, HNF1A, FOXO3, ELK1, NFKB1, POU5F1, NR4A2, LHX3, FEV, MAFG, ELF5, GABPA, NFE2L1, FOXA2*
31323	Regulation of cellular metabolic process	2,02E-09	6,25E-08	16/16 100.0%	1351/8356 16.1%	*MEF2A, NFYA, STAT3, HNF1A, FOXO3, ELK1, NFKB1, POU5F1, NR4A2, LHX3, FEV, MAFG, ELF5, GABPA, NFE2L1, FOXA2*
19222	Regulation of metabolic process	5,71E-09	1,63E-07	16/16 100.0%	1441/8356 17.2%	*MEF2A, NFYA, STAT3, HNF1A, FOXO3, ELK1, NFKB1, POU5F1, NR4A2, LHX3, FEV, MAFG, ELF5, GABPA, NFE2L1, FOXA2*
50794	Regulation of cellular process	2,01E-05	5,34E-04	16/16 100.0%	2396/8356 28.6%	*MEF2A, NFYA, STAT3, HNF1A, FOXO3, ELK1, NFKB1, POU5F1, NR4A2, LHX3, FEV, MAFG, ELF5, GABPA, NFE2L1, FOXA2*
50789	Regulation of biological process	6,61E-05	1,63E-03	16/16 100.0%	2580/8356 30.8%	*MEF2A, NFYA, STAT3, HNF1A, FOXO3, ELK1, NFKB1, POU5F1, NR4A2, LHX3, FEV, MAFG, ELF5, GABPA, NFE2L1, FOXA2*
65007	Biological regulation	2,59E-04	5,99E-03	16/16 100.0%	2809/8356 33.6%	*MEF2A, NFYA, STAT3, HNF1A, FOXO3, ELK1, NFKB1, POU5F1, NR4A2, LHX3, FEV, MAFG, ELF5, GABPA, NFE2L1, FOXA2*
6357	Regulation of transcription from RNA polymerase II promoter	1,01E-03	2,21E-02	7/16 43.7%	230/8356 2.7%	*NR4A2, NFYA, STAT3, HNF1A, FOXO3, GABPA, NFKB1*
45944	Positive regulation of transcription from RNA polymerase II promoter	4,01E-02	8,27E-01	5/16 31.2%	136/8356 1.6%	*NR4A2, NFYA, HNF1A, FOXO3, NFKB1*
42593	Glucose homeostasis	9,97E-02	1,85E+00	3/16 18.7%	23/8356 0.2%	*STAT3, HNF1A, FOXO3*
33500	Carbohydrate homeostasis	9,97E-02	1,85E+00	3/16 18.7%	23/8356 0.2%	*STAT3, HNF1A, FOXO3*
45893	Positive regulation of transcription, DNA-dependent	1,07E-01	1,88E+00	5/16 31.2%	166/8356 1.9%	*NR4A2, NFYA, HNF1A, FOXO3, NFKB1*
51254	Positive regulation of RNA metabolic process	1,13E-01	1,91E+00	5/16 31.2%	168/8356 2.0%	*NR4A2, NFYA, HNF1A, FOXO3, NFKB1*

45941	Positive regulation of transcription	2,00E-01	3,23E+00	5/16 31.2%	189/8356 2.2%	*NR4A2, NFYA, HNF1A, FOXO3, NFKB1*
10628	Positive regulation of gene expression	2,63E-01	4,07E+00	5/16 31.2%	200/8356 2.3%	*NR4A2, NFYA, HNF1A, FOXO3, NFKB1*
10557	Positive regulation of macromolecule biosynthetic process	4,35E-01	6,46E+00	5/16 31.2%	222/8356 2.6%	*NR4A2, NFYA, HNF1A, FOXO3, NFKB1*
45935	Positive regulation of nucleobase, nucleoside, nucleotide and nucleic acid metabolic process	4,74E-01	6,76E+00	5/16 31.2%	226/8356 2.7%	*NR4A2, NFYA, HNF1A, FOXO3, NFKB1*
51173	Positive regulation of nitrogen compound metabolic process	5,49E-01	7,54E+00	5/16 31.2%	233/8356 2.7%	*NR4A2, NFYA, HNF1A, FOXO3, NFKB1*
31328	Positive regulation of cellular biosynthetic process	6,32E-01	8,37E+00	5/16 31.2%	240/8356 2.8%	*NR4A2, NFYA, HNF1A, FOXO3, NFKB1*
9891	Positive regulation of biosynthetic process	6,84E-01	8,75E+00	5/16 31.2%	244/8356 2.9%	*NR4A2, NFYA, HNF1A, FOXO3, NFKB1*
7275	Multicellular organismal development	1,22E+00	1,51E+01	8/16 50.0%	922/8356 11.0%	*MEF2A, NR4A2, MAFG, ELF5, STAT3, HNF1A, FOXO3, GABPA*
10604	Positive regulation of macromolecule metabolic process	1,97E+00	2,35E+01	5/16 31.2%	305/8356 3.6%	*NR4A2, NFYA, HNF1A, FOXO3, NFKB1*
32502	Developmental process	2,27E+00	2,57E+01	8/16 50.0%	1006/8356 12.0%	*MEF2A, NR4A2, MAFG, ELF5, STAT3, HNF1A, FOXO3, GABPA*
31325	Positive regulation of cellular metabolic process	2,29E+00	2,57E+01	5/16 31.2%	315/8356 3.7%	*NR4A2, NFYA, HNF1A, FOXO3, NFKB1*
9893	Positive regulation of metabolic process	3,09E+00	3,37E+01	5/16 31.2%	336/8356 4.0%	*NR4A2, NFYA, HNF1A, FOXO3, NFKB1*
32501	Multicellular organismal process	9,88E+00	1,05E+02	8/16 50.0%	1243/8356 14.8%	*MEF2A, NR4A2, MAFG, ELF5, STAT3, HNF1A, FOXO3, GABPA*
1701	In utero embryonic development	1,04E+01	1,07E+02	3/16 18.7%	108/8356 1.2%	*MAFG, HNF1A, GABPA*
48469	Cell maturation	1,35E+01	1,36E+02	2/16 12.5%	29/8356 0.3%	*NR4A2, FOXO3*
48731	System development	1,46E+01	1,42E+02	6/16 37.5%	715/8356 8.5%	*MEF2A, NR4A2, ELF5, STAT3, HNF1A, FOXO3*
1544	Initiation of primordial ovarian follicle growth	1,91E+01	1,61E+02	1/16 6.2%	1/8356 0.0%	*FOXO3*
1556	Oocyte maturation	1,91E+01	1,61E+02	1/16 6.2%	1/8356 0.0%	*FOXO3*
45083	Negative regulation of interleukin-12 biosynthetic process	1,91E+01	1,61E+02	1/16 6.2%	1/8356 0.0%	*NFKB1*
6699	Bile acid biosynthetic process	1,91E+01	1,61E+02	1/16 6.2%	1/8356 0.0%	*HNF1A*
48341	Paraxial mesoderm formation	1,91E+01	1,61E+02	1/16 6.2%	1/8356 0.0%	*HNF1A*
15721	Bile acid and bile salt transport	1,91E+01	1,61E+02	1/16 6.2%	1/8356 0.0%	*HNF1A*
21700	Developmental maturation	2,32E+01	1,91E+02	2/16 12.5%	38/8356 0.4%	*NR4A2, FOXO3*
48856	Anatomical structure development	2,56E+01	1,99E+02	6/16 37.5%	797/8356 9.5%	*MEF2A, NR4A2, ELF5, STAT3, HNF1A, FOXO3*
48871	Multicellular organismal homeostasis	2,57E+01	1,99E+02	2/16 12.5%	40/8356 0.4%	*STAT3, HNF1A*
7610	Behavior	2,57E+01	1,99E+02	3/16 18.7%	148/8356 1.7%	*NR4A2, MAFG, STAT3*
30534	Adult behavior	2,83E+01	2,14E+02	2/16 12.5%	42/8356 0.5%	*NR4A2, MAFG*

30154	Cell differentiation	2,91E+01	2,16E+02	5/16 31.2%	551/8356 6.5%	*MEF2A, NR4A2, ELF5, STAT3, FOXO3*
43009	Chordate embryonic development	3,10E+01	2,25E+02	3/16 18.7%	158/8356 1.8%	*MAFG, HNF1A, GABPA*
9792	Embryonic development ending in birth or egg hatching	3,16E+01	2,25E+02	3/16 18.7%	159/8356 1.9%	*MAFG, HNF1A, GABPA*
48869	Cellular developmental process	3,32E+01	2,32E+02	5/16 31.2%	568/8356 6.7%	*MEF2A, NR4A2, ELF5, STAT3, FOXO3*
1547	Antral ovarian follicle growth	3,83E+01	2,50E+02	1/16 6.2%	2/8356 0.0%	*FOXO3*
9994	Oocyte differentiation	3,83E+01	2,50E+02	1/16 6.2%	2/8356 0.0%	*FOXO3*
48599	Oocyte development	3,83E+01	2,50E+02	1/16 6.2%	2/8356 0.0%	*FOXO3*
10551	Regulation of gene-specific transcription from RNA polymerase II promoter	3,84E+01	2,50E+02	2/16 12.5%	49/8356 0.5%	*HNF1A, NFKB1*
48608	Reproductive structure development	3,99E+01	2,55E+02	2/16 12.5%	50/8356 0.5%	*HNF1A, FOXO3*
51239	Regulation of multicellular organismal process	5,44E+01	3,13E+02	4/16 25.0%	390/8356 4.6%	*NR4A2, MAFG, FOXO3, NFKB1*
1542	Ovulation from ovarian follicle	5,73E+01	3,13E+02	1/16 6.2%	3/8356 0.0%	*FOXO3*
30728	Ovulation	5,73E+01	3,13E+02	1/16 6.2%	3/8356 0.0%	*FOXO3*
8206	Bile acid metabolic process	5,73E+01	3,13E+02	1/16 6.2%	3/8356 0.0%	*HNF1A*
45604	Regulation of epidermal cell differentiation	5,73E+01	3,13E+02	1/16 6.2%	3/8356 0.0%	*MAFG*
48339	Paraxial mesoderm development	5,73E+01	3,13E+02	1/16 6.2%	3/8356 0.0%	*HNF1A*
48340	Paraxial mesoderm morphogenesis	5,73E+01	3,13E+02	1/16 6.2%	3/8356 0.0%	*HNF1A*
31018	Endocrine pancreas development	5,73E+01	3,13E+02	1/16 6.2%	3/8356 0.0%	*HNF1A*
21952	Central nervous system projection neuron axonogenesis	5,73E+01	3,13E+02	1/16 6.2%	3/8356 0.0%	*NR4A2*
60397	JAK-STAT cascade involved in growth hormone signaling pathway	5,73E+01	3,13E+02	1/16 6.2%	3/8356 0.0%	*STAT3*
10033	Response to organic substance	5,84E+01	3,14E+02	3/16 18.7%	198/8356 2.3%	*NR4A2, STAT3, HNF1A*
16265	Death	6,26E+01	3,28E+02	3/16 18.7%	203/8356 2.4%	*MEF2A, NR4A2, FOXO3*
9719	Response to endogenous stimulus	6,27E+01	3,28E+02	2/16 12.5%	63/8356 0.7%	*NR4A2, STAT3*
48522	Positive regulation of cellular process	6,51E+01	3,35E+02	5/16 31.2%	664/8356 7.9%	*NR4A2, NFYA, HNF1A, FOXO3, NFKB1*
32583	Regulation of gene-specific transcription	6,66E+01	3,39E+02	2/16 12.5%	65/8356 0.7%	*HNF1A, NFKB1*
45075	Regulation of interleukin-12 biosynthetic process	7,64E+01	3,63E+02	1/16 6.2%	4/8356 0.0%	*NFKB1*
42053	Regulation of dopamine metabolic process	7,64E+01	3,63E+02	1/16 6.2%	4/8356 0.0%	*NR4A2*

42069	Regulation of catecholamine metabolic process	7,64E+01	3,63E+02	1/16 6.2%	4/8356 0.0%	*NR4A2*
21955	Central nervous system neuron axonogenesis	7,64E+01	3,63E+02	1/16 6.2%	4/8356 0.0%	*NR4A2*
33238	Regulation of cellular amine metabolic process	7,64E+01	3,63E+02	1/16 6.2%	4/8356 0.0%	*NR4A2*
48878	Chemical homeostasis	8,22E+01	3,86E+02	3/16 18.7%	224/8356 2.6%	*STAT3, HNF1A, FOXO3*
3	Reproduction	8,43E+01	3,91E+02	3/16 18.7%	226/8356 2.7%	*STAT3, HNF1A, FOXO3*
9790	Embryonic development	8,95E+01	4,07E+02	3/16 18.7%	231/8356 2.7%	*MAFG, HNF1A, GABPA*
60416	Response to growth hormone stimulus	9,54E+01	4,07E+02	1/16 6.2%	5/8356 0.0%	*STAT3*
42551	Neuron maturation	9,54E+01	4,07E+02	1/16 6.2%	5/8356 0.0%	*NR4A2*
45648	Positive regulation of erythrocyte differentiation	9,54E+01	4,07E+02	1/16 6.2%	5/8356 0.0%	*FOXO3*
71378	Cellular response to growth hormone stimulus	9,54E+01	4,07E+02	1/16 6.2%	5/8356 0.0%	*STAT3*
42416	Dopamine biosynthetic process	9,54E+01	4,07E+02	1/16 6.2%	5/8356 0.0%	*NR4A2*
60396	Growth hormone receptor signaling pathway	9,54E+01	4,07E+02	1/16 6.2%	5/8356 0.0%	*STAT3*
48518	Positive regulation of biological process	1,13E+02	4,61E+02	5/16 31.2%	757/8356 9.0%	*NR4A2, NFYA, HNF1A, FOXO3, NFKB1*
60644	Mammary gland epithelial cell differentiation	1,14E+02	4,61E+02	1/16 6.2%	6/8356 0.0%	*ELF5*
42755	Eating behavior	1,14E+02	4,61E+02	1/16 6.2%	6/8356 0.0%	*STAT3*
48477	Oogenesis	1,14E+02	4,61E+02	1/16 6.2%	6/8356 0.0%	*FOXO3*
1975	Response to amphetamine	1,14E+02	4,61E+02	1/16 6.2%	6/8356 0.0%	*NR4A2*
7399	Nervous system development	1,17E+02	4,67E+02	3/16 18.7%	255/8356 3.0%	*MEF2A, NR4A2, STAT3*

Lines highlighted in green refer to biological processes related to reproduction.
